# Indole-based perenosins as highly potent HCl transporters and potential anti-cancer agents

**DOI:** 10.1038/s41598-017-09645-9

**Published:** 2017-08-24

**Authors:** Laura A. Jowett, Ethan N. W. Howe, Vanessa Soto-Cerrato, Wim Van Rossom, Ricardo Pérez-Tomás, Philip A. Gale

**Affiliations:** 1 School of Chemistry (F11), The University of Sydney, 2006 Sydney, NSW Australia; 2 Department of Pathology and Experimental Therapeutics, Cancer Cell Biology Research Group, University of Barcelona, Barcelona, Spain; 3 Chemistry, University of Southampton, Highfield, Southampton, SO17 1BJ UK

## Abstract

Prodigiosin is one of the most potent anion transporters in lipid bilayer membranes reported to date. Inspired by the structure of this natural product, we have recently designed and synthesised a new class of H^+^/Cl^−^ cotransporters named ‘perenosins’. Here we report a new library of indole-based perenosins and their anion transport properties. The new transporters demonstrated superior transmembrane transport efficiency when compared to other indole-based transporters, due to favourable encapsulating effects from the substituents on the perenosin backbone. Anion transport assays were used to determine the mechanism of chloride transport revealing that the compounds function as ‘strict’ HCl cotransporters. Cell viability studies showed that some compounds specifically trigger late-onset cell death after 72 h with a unique correlation to the position of alkyl chains on the perenosins. Further investigations of cell death mechanism showed a mixture of cell cycle arrest and apoptosis was responsible for the observed decrease in cell viability.

## Introduction

**Figure 1:**
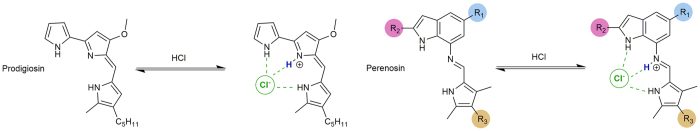
Schematic of prodigiosin (left) and perenosin (right) showing the chemical structures of these H^+^/Cl^−^ cotransporters as free receptors and their respective HCl complexes. Three modification positions for the perenosins reported in this work are highlighted R_1_, R_2_ and R_3_.

Synthetic transmembrane anion transporters are an area of intense current interest due to their potential application in the treatment of diseases^1–3^. The potential for anti-cancer activity^4^, and to replace the anion transport process in patients with genetically impaired ion channels^5^, has led to a significant rise in the number of small molecule anion transporters developed, such as indole-based receptors^6^, the *ortho*-phenylenediamine-based bisureas^7^, as well as many others^8–12^. However, the natural product prodigiosin (Fig. 1) remains a benchmark for new anion transporters due to its potent anion transport properties in lipid bilayer membranes^13–16^. Prodigiosin has been shown to bind H^+^/Cl^−^ through a combination of hydrogen bonds and electrostatic interactions^17^, allowing it to facilitate H^+^/Cl^−^ symport through membranes resulting in pH dissipation in both model liposomal and cell assays^18–21^. In cells, the dysregulation of ion homeostasis through chloride influx, which can be facilitated by anion transporters^22^, can induce cell shrinkage and leads to the apoptotic cell death pathway^23–26^. Another consequence of the dysregulation of intracellular chloride concentrations is disruption of autophagy, through the interruption of lysosomal enzyme activity caused by altering the lysosomal pH^27^. The disruption of autophagy by small molecule anion transporters results in the suppression of cell proliferation which is more pronounced in tumour cells^28^. A second family of recognised H^+^/Cl^−^ cotransporters, the tambjamines^29–31^, have also been shown to induce lysosomal dysfunction^29^ and autophagy blockage^32^ as well as cancer cell death^3^.

The encouraging antitumor activity displayed by members of the prodiginines family^33^ has promoted the development of new synthetic derivatives such as obatoclax^34^. Recently, we have developed a new class of anion transporters, the “perenosins”, with structures inspired by that of prodigiosin (Fig. 1)^35^. The incorporation of an imine linker in the structural design of perenosins is beneficial for further pharmacokinetic studies, especially the metabolic breakdown pathway after administration.

**Figure 2:**
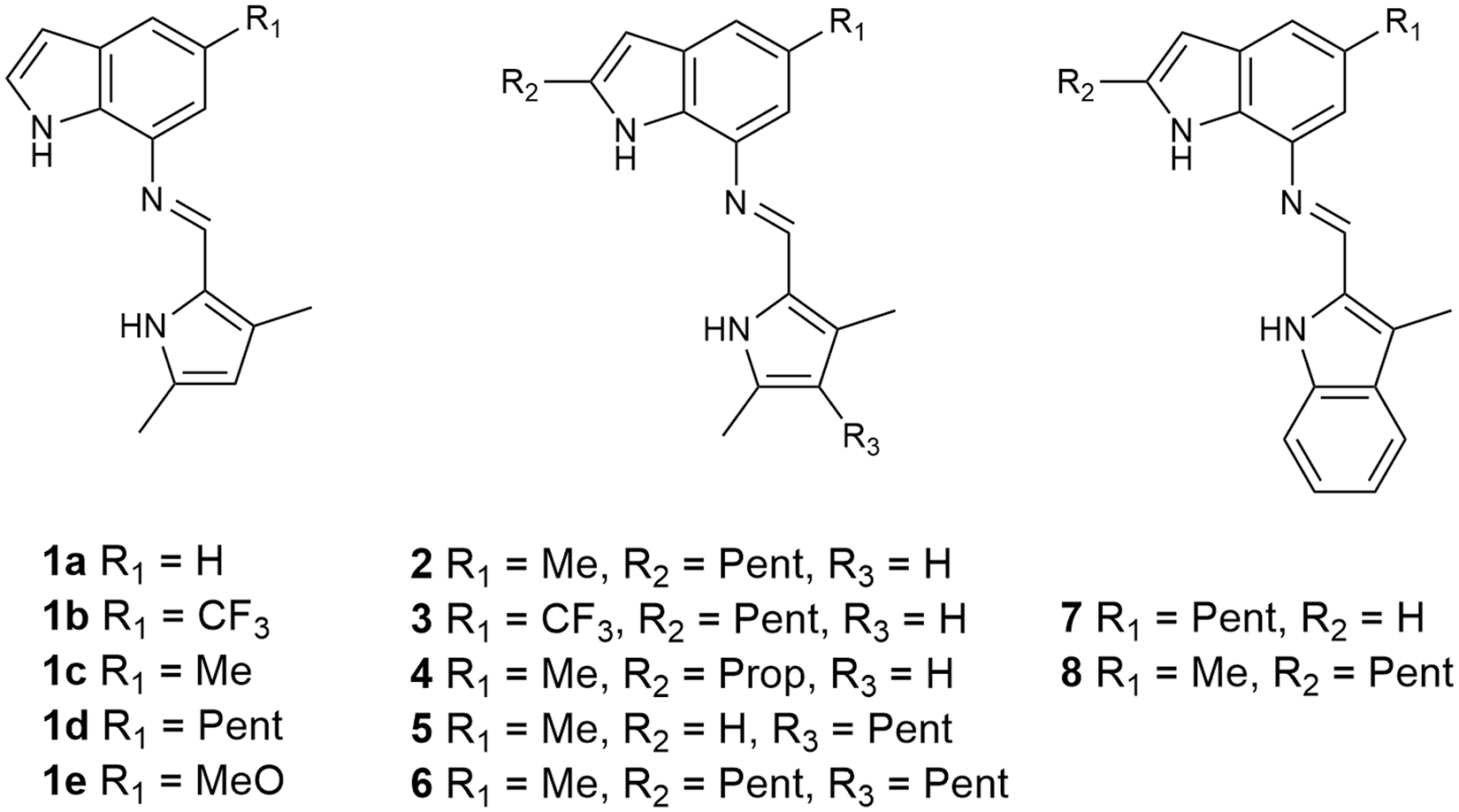
Structures of the perenosins reported in this work; **1a**–**e** from the first generation are used for comparison, **2**–**6** are new analogues based on the same indole-pyrrole scaffold of **1**, and **7**–**8** are based on a modified bis-indole scaffold.

Perenosins consist of a dimethylpyrrole unit linked through an imine moiety to an indole, benzimidazole or indazole heterocyclic functional group, which are the basis of other anion receptor systems^36, 37^. Perenosins function as H^+^/Cl^−^ cotransporters in a similar fashion to prodigiosin, as protonation of the imine nitrogen leads to binding of the chloride anion. Liposome-based anion transport studies showed that the indole derivatives such as compound **1d** (Fig. 2) performed as one of the more effective transporters, with superior cytotoxic properties among the initial compounds studied.

Encouraged by the outstanding transmembrane transport and cytotoxic properties of compound **1d**, we have developed a new family of perenosins **2**–**6** based on the indole-pyrrole scaffold of **1a**–**e**, and bis-indole perenosins **7**–**8**. Here, we report detailed studies of the chloride anion binding, p*K*_*a*_ in aqueous and lipid bilayer environments and transmembrane transport capabilities (and mechanisms) of the indole-based perenosins. We have also performed in-depth cell studies of the cytotoxic properties of these compounds (**1a**–**e** and **2**–**8**), and have comprehensively elucidated the transport mechanism in liposomes as well as the cell death pathway induced by perenosins for the first time.

## Results and Discussion

### Indole-Based Perenosins

#### Design and synthesis

The pyrrole-indole derivatives of the first generation of perenosins featured modifications at one site, R_1_ (Fig. 1). Adapting the reported stepwise synthetic approach allowed incorporation of a variety of substituents at the R_2_ and R_3_ positions, as highlighted in Fig. 1 (see Supplementary Information for full synthetic schemes). Briefly, non-commercial indoles were synthesised from a 2-nitroaniline starting material (with the respective R_1_ substituent) *via* iodination, followed by a Sonogashira coupling with an alkyne (corresponding to the R_2_ substituent), and finally a base assisted cyclisation step. Manipulation of the pyrrole through the Knorr pyrrole synthesis afforded the pentyl-substituted moiety at the R_3_ position. Hydrogenation of the nitroindole and subsequent condensation with the pyrrole aldehyde gave the final products in good yields.

The new family of perenosins reported here expands the library of perenosin derivatives. Introducing alkyl chains of different lengths at R_2_ for compounds **2** (pentyl) and **4** (propyl) allowed investigation into the effects of encapsulation of the binding site on the transport properties of the perenosins. Another advantage of using the R_2_ position for alkylation was that other substituents, such as a CF_3_ group (compound **3**) which has been shown to enhance transport capabilities^38, 39^, could be positioned at R_1_ whilst retaining the alkyl chain in the structure. R_3_ was alkylated allowing investigation into the effects of optimal lipophilicity^40–42^, showing the differences between having a pentyl chain on the pyrrole rather than indole (compound **5**) or on both (compound **6**). As indole-NH groups are more acidic and hence are better hydrogen-bond donors than the pyrrole-NH groups^43, 44^, we have also developed two bis-indole derivatives (**7** and **8**) as an alternative perenosin scaffold to potentially improve anion binding and its effect on transmembrane transport.

**Figure 3:**
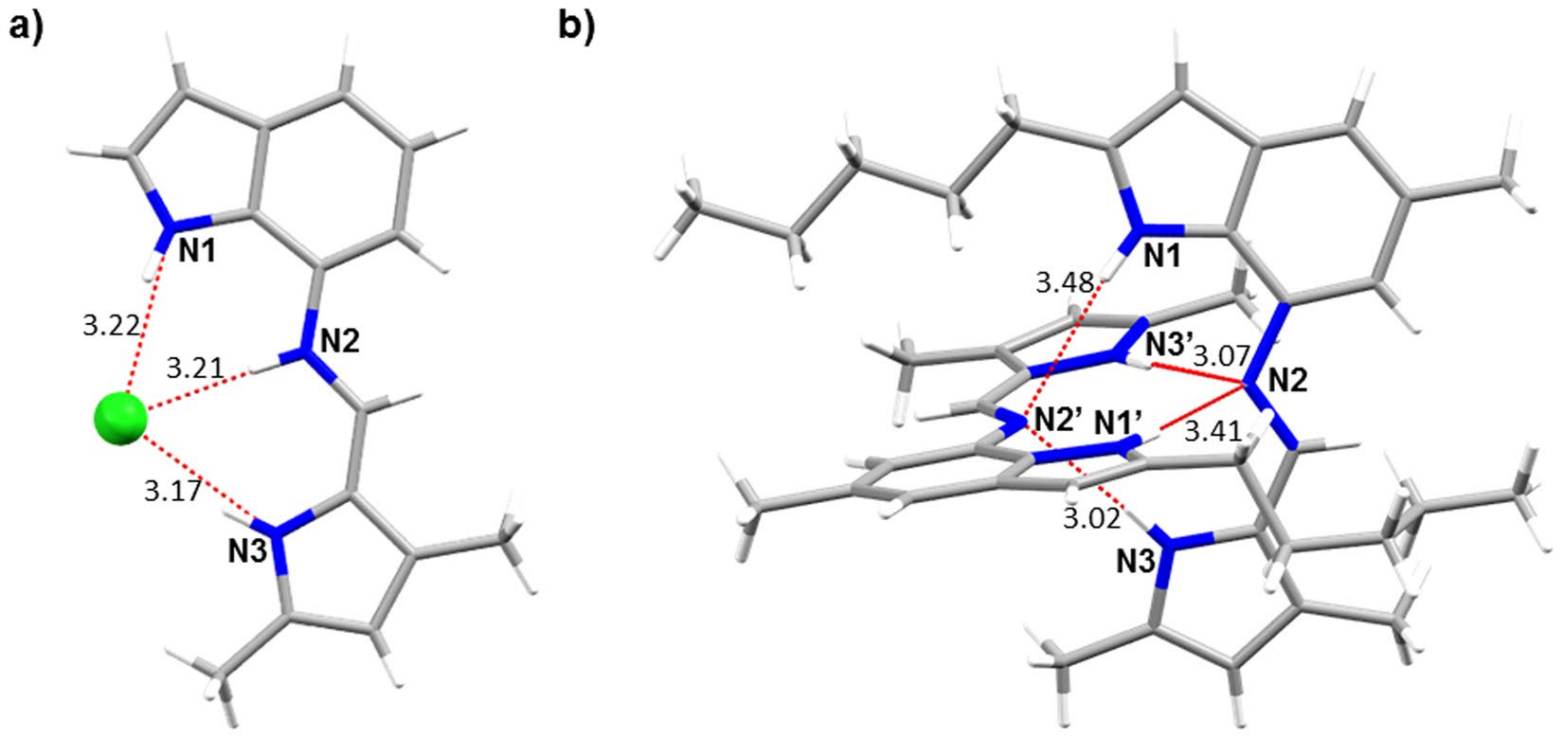
Single-crystal X-ray structures of (**a**) **1a**·HCl complex (CCDC: 1441636)^35^ with N1···Cl, N2···Cl and N3···Cl distances displayed as red dashed lines with measurements (Å) in black; and (**b**) **2** with N1···N2′, N3···N2′, N1′···N2 and N3′···N2 distances displayed as red dashed lines, with measurements (Å) in black.

Previously, we reported the X-ray crystal structure of the HCl complex of the unsubstituted analogue (R_1_ = H) of **1a** ^35^ (Fig. 3a), here we present the X-ray structure of free perenosin **2** (Fig. 3b), see the Supplementary Information for the single crystal X-ray structures of compounds **1d** and **4**. The structure of compound **2** reveals the formation of an orthogonal ‘narcissistic’ dimer^45–47^ in the solid state *via* intermolecular hydrogen bonds between the imine nitrogen acceptor and two NH donors from pyrrole and indole.

#### Anion Binding Studies

**Figure 4:**
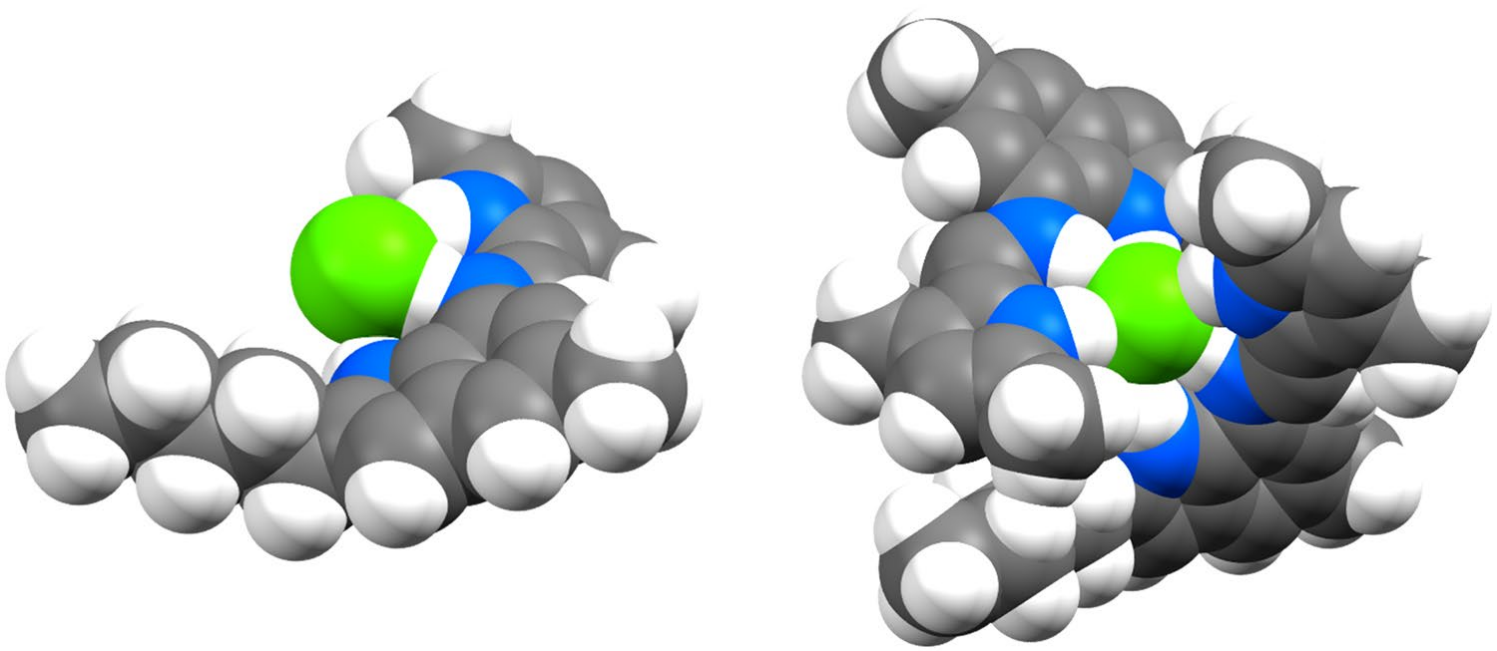
Theoretical (PM6^51^) optimised structures performed using Gaussian09^52^ for compound **2** in both the 1:1 and 2:1 binding mode; shown in space-filling models.

Proton NMR titrations were performed in DMSO-*d*_6_/0.5% H_2_O, to assess the ability of compounds **1d** and **2**–**8** to bind chloride, added as the tetrabutylammonium (TBA) salt, in solution. Free perenosins showed weak binding of the chloride anion. However, in the presence of one equivalent of HPF_6_, strong binding to chloride was observed through downfield shifts of the pyrrole-NH, indole-NH and the protonated imine-NH^+^. All three resonances were fitted globally to the 1:1 or 2:1 (sandwich) binding models using Bindfit^48^. Apart from **1d**; the data of perenosins **2**–**6** are more complex and did not fit well to the 1:1 model, however, it can be fitted to the 2:1 binding model (see Supplementary Information) with an improvement in the quality of fit (cov_fit_) by >6 times (see Table 1). The curve fitting analysis demonstrated that a 2:1 sandwich-binding mode for perenosins **1d** and **2**–**6** is plausible, with the protonated receptors showing a 2:1 binding mode in the presence of small amounts of chloride, and a 1:1 complex at higher chloride concentrations. The 1:1 and 2:1 binding modes are supported by the PM6 optimised structures of perenosin **2**, demonstrating an octahedral 2:1 host:guest complex (Fig. 4).

**Table 1:** Overview of association constants for the complexation between protonated perenosins (**1d** and **2**–**8**) and Cl^−^ (as TBA salt) in DMSO-*d*_6_/0.5% H_2_O at 298 K.

Perenosin	Association constants (M^−1^) of Cl^−^ in the presence of HPF_6_ (1 equiv.)					p*K*_a (aq)_	p*K*_a (mem)_
	1:1 (*K*_a_)	cov_fit_^**a**^	2:1	β_21_^b^	cov_fit_^**a**^		
**1d**	605	3.3 × 10^−3^	*K*_11_: 1750 *K*_21_: 190	3.33 × 10^5^	1.5 × 10^−3^	6.35	5.42
**2** ^**c**^	2450	6.2 × 10^−3^	*K*_11_: 2500 *K*_21_: 473	1.18 × 10^6^	1.0 × 10^−3^	6.08	5.85
**3** ^**c**^	3440	6.4 × 10^−2^	*K*_11_: 190 *K*_21_: 2070	3.93 × 10^5^	2.6 × 10^−3^	n.d.^e^	n.d.^e^
**4** ^**c**^	3440	1.1 × 10^−2^	*K*_11_: 1530 *K*_21_: 306	4.68 × 10^5^	1.2 × 10^−3^	6.78	6.12
**5** ^**c**^	2610	5.2 × 10^−3^	*K*_11_: 331 *K*_21_: 14	4.63 × 10^3^	8.6 × 10^−4^	6.44	5.81
**6** ^**c**^	78300	8.8 × 10^−2^	*K*_11_: 1090 *K*_21_: 158	1.72 × 10^5^	7.5 × 10^−3^	n.d.^e^	n.d.^e^
**7** ^**d**^	157	1.0 × 10^−2^	*K*_11_: 72 *K*_21_: 507	3.65 × 10^4^	8.2 × 10^−4^	5.51	5.07
**8** ^**d**^	142	4.0 × 10^−3^	*K*_11_: 184 *K*_21_: 193	3.55 × 10^4^	1.2 × 10^−3^	6.28	5.62

^a^The covariance of the fit (cov_fit_) is calculated by dividing the (co) variance of the residual (experimental data − calculated data) with the covariance of the experimental data^50^. ^b^The overall association constant (β_21_) for the 2:1 host:guest complex; calculated by multiplying *K*_11_ and *K*_21_. ^c^Poor fitting to the 1:1 binding model, the derived *K*_a_ is reported here for completeness and comparison of the cov_fit_ between 1:1 and 2:1 fitting. ^d^Partial protonation of bis-indole perenosins **7** (~20%) and **8** (~10%), the derived association constants should be viewed with caution. ^e^p*K*_a_ not determined due to precipitation from solution.

By comparing the derived association constants from the 2:1 fitting analysis for perenosins **1d** and **2**–**6**, **2** emerged to be the best receptor for binding to chloride, with the largest *K*_11_ (and *K*_21_ for most cases but smaller than **3**, to be discussed later), as well as the largest overall binding constant β_21_. The superior chloride binding of **2** suggests that the encapsulating effect of the binding site by pentyl chain at the R_2_ position can enhance the binding affinity for chloride. This hypothesis is further proven by the decrease in binding for **4** (with a shorter propyl chain, hence less encapsulating) and more significant decrease for **5** (absence of an alkyl chain at the R_2_ position); whilst compound **6** exhibits improved binding compared to **5** when the pentyl chain is re-introduced at the R_2_ position. Interestingly, only compound **3** exhibits strong positive cooperativity^49^, where *K*_21_ is much greater than *K*_11_^50^, hence a preferential 2:1 (perenosin:chloride) complex formation occurs (see speciation ratio plots in Supplementary Information).

Addition of one mole equivalent of HPF_6_ to bis-indole perenosins **7** and **8** resulted in partial protonation of the imine nitrogen, approximately 20% and 10% respectively. Subsequent titration of **7** and **8** in the presence of HPF_6_ (1 mole equiv.) with tetrabutylammonium chloride induced a downfield shift of only one indole-NH (NH1 or NH1′ shown in Fig. 3), and resulted in weaker binding to chloride compared to the indole-pyrrole perenosins. Although the titration isotherms of **7** and **8** can be fitted to both 1:1 and 2:1 binding modes, the derived association constants must be viewed with caution.

It must be noted that the formation of 2:1 and 1:1 host:guest complexes during the titration binding studies resulted in a degree of uncertainties in the derived binding constants due to the complexity of the data. We acknowledge that more in-depth studies (such as solvent effects and protonation equilibrium in the same solvent environment) are required to gain better understanding of the 2:1 sandwich binding and the encapsulation effects. However, the focus of this work is on the transport and biological activities, hence it is more suitable to further explore the binding properties in a separate work.

#### p*K*_a_ Studies

The p*K*_a_ at which protonation of the imine (N2, see Fig. 3) occurs gives an indication of the optimum pH for the binding of chloride anions. This is an important consideration in anti-cancer drug development as the external pH environment of cancer cells is mildly acidic (pH 6.7)^53^ compared to that of healthy cells (pH 7.2–7.5)^54^. This can be exploited to selectively target cancer cells and potentially reduce the cytotoxic effects of perenosins when used as therapeutics for anti-cancer treatment. The apparent p*K*_a_ values, shown in Table 1, for compounds **1d** and **2**–**8** were determined both in aqueous solution (p*K*_a (aq)_) and in the presence of a 1-palmitoyl-2-oleoyl-*sn*-glycero-3-phosphocholine (POPC) lipid bilayer membrane (p*K*_a (mem)_). UV spectrophotometric titrations were conducted by varying the pH condition from neutral to acidic with the titration of either HPF_6_ or HCl. While the neutral perenosin has an absorbance maxima of ~380 nm for **1d** and **2**–**6** and ~330 nm for **7**–**8**, protonation resulted in a red shift to ~420 nm and ~400 nm respectively.

The apparent p*K*_a_ values were derived from nonlinear curve fitting of the absorbance plotted against the pH; global fitting of both wavelengths corresponding to the neutral and protonated species were performed whenever feasible, see Supplementary Information for details. The p*K*_a_ values obtained for **1d** and **2**–**8** are lower in the presence of a lipid bilayer, indicating that the lipid bilayer stabilises the neutral form of the compound to some extent^55, 56^.

### Transmembrane Transport Studies

#### Cl^−^/NO_3_^−^ antiport at pH 7.2

The chloride transport properties of the new perenosins **2**–**8** were compared to **1d** using the chloride/nitrate exchange assay monitored by a chloride ion selective electrode (ISE). Typically, unilamellar vesicles were prepared from POPC, containing an intravesicular NaCl solution (489 mM with 5 mM phosphate buffer at pH 7.2) suspended in a NaNO_3_ solution (489 mM with 5 mM phosphate buffer at pH 7.2). All perenosins were added as a DMSO solution and the rate of chloride efflux was monitored by the chloride ISE. At the end of the experiment (300 s), detergent was added to lyse the vesicles and obtain the 100% chloride efflux reading. Dose response studies of **1d** and **2**–**8** were performed to allow Hill analysis, giving the EC_50_ values and Hill coefficients as shown in Table 2.

**Table 2:** EC_50_ values shown for the Cl^−^/NO_3_^−^ antiport assay, the NMDG-Cl assay alone, with gramicidin (Gra) and with oleic acid (OA), with the chloride selectivity (S_G_) and the factor of enhancement (F_OA_) included.

Perenosin	clog P^a^	Cl^−^/NO_3_^−^ at pH 7.2 (by ISE)		NMDG-Cl (by HPTS)				
		EC_50_ (mol%)^b^	n^c^	EC_50_ (mol%)^d^	EC_50 (Gra)_ (mol%)^e^	EC_50 (OA)_ (mol%)^f^	S_G_^g^	F_OA_^h^
**1d**	5.1	0.1220	1.2	0.01073	0.00656	0.00925	1.6	1.2
**2**	4.9	0.0043	1.3	0.00062	0.00037	0.00085	1.7	0.7
**3**	5.7	n.d.^i^	n.d.^i^	0.01151	0.00879	0.04912	1.3	0.2
**4**	4.4	0.0111	1.1	0.00069	0.00038	0.00008	1.8	8.6
**5**	5.3	0.0227	1.3	0.00231	0.00095	0.00273	2.4	0.9
**6**	6.9	0.0852	0.3	0.00249	0.00211	0.00208	1.2	1.2
**7**	6.1	>10^j^	n/a^j^	n.d.^j^	n.d.^j^	n.d.^j^	n/a^j^	n/a^j^
**8**	6.2	>10^j^	n/a^j^	n.d.^j^	n.d.^j^	n.d.^j^	n/a^j^	n/a^j^

^a^average clog P values calculated using VCCLab^62^. ^b^EC_50_ at 270 s, shown as carrier: lipid molar percent. ^c^Hill coefficient reveals the stoichiometry of the complex mediating transport. ^d^EC_50_ from the NMDG-Cl assay, measuring H^+^/Cl^−^ symport. ^e^EC_50_ in the presence of Gra, showing the total H^+^/Cl^−^ symport possible with no rate limiting H^+^/OH^−^ transport. Gra concentration has been optimised to 0.1 mol%. ^f^EC_50_ in the presence of OA, showing changes in transport due to acceleration of the natural proton transport facilitated by fatty acids. OA concentration has been optimised to 2 mol%. ^g^S_G_ > 1 indicates Cl^−^ selectivity and is calculated by dividing EC_50_ in absence of Gra by EC_50 (Gra)_. ^h^Factor of enhancement in the overall H^+^/Cl^−^ cotransport rate is calculated by dividing EC_50_ in absence of Gra or OA by EC_50 (OA)_. F_OA_ > 1 suggests the receptor can assist the flip-flop of OA^−^ increasing pH dissipation. ^i^n.d. EC_50_ not determined by ISE due to poor solubility. ^j^Weak transport ability where EC_50_ for Cl^−^/NO_3_^−^ by ISE is larger than 10 mol%, hence NMDG-Cl by HPTS assay not determined (n.d.).

Perenosin **1d**, bearing a pentyl group at R_1_ position, achieved moderate transport efficiency with an EC_50_ of 0.122 mol%. On the other hand compound **2**, with pentyl substitution at R_2_ position, has a remarkable EC_50_ value of 0.0043 mol% being the most effective transporter of the entire series, and is comparable to the natural product and synthetic analogues of tambjamines^39^. However, introduction of the pentyl group at the R_3_ position in compound **5** decreased the transport capabilities as compared to compound **2** (but the compound still performed better than **1d**), with an EC_50_ value of 0.0227 mol%. While perenosins **1d**, **2** and **5** all possess a pentyl chain at R_1_, R_2_ or R_3_ positions with very similar log P values (4.9–5.3), the respective transport efficacies are significantly different. This outcome highlights the beneficial effect of strategically positioning the pentyl chain to improve the mobility within the lipid bilayer and more importantly, enhancing the encapsulation of the binding site. Compound **3** achieved a maximum chloride efflux of 35% (after 300 s) at a high loading of 10 mol%, so an EC_50_ value could not be determined; this is likely due to poor solubility in aqueous solution as was observed in the p*K*_a_ studies. Compound **4** bearing a shorter propyl group at R_2_ position resulted in a slightly poorer EC_50_ of 0.0111 mol% c.f. **2**, which can be attributed to the lower lipophilicity and lesser encapsulating effect by the shorter alkyl chain. The most lipophilic compound **6**, with two pentyl groups at the R_2_ and R_3_ positions, resulted in a decrease of transport capabilities with an EC_50_ of 0.0852 mol% and this is presumably due to the compound’s unfavourably high lipophilicity and poor solubility. Finally, compounds **7**–**8** gave weak transport activity at pH 7.2 and Hill analysis could not be used to calculate EC_50_, potentially due to a number of factors; (1) the p*K*_a_ values being two pH units lower than the pH condition of the experiment for compound **7**, (2) weak chloride binding and (3) unfavourably high lipophilicity. Overall, the new perenosins bearing the indole-pyrrole scaffold achieved better transport efficiencies than **1d**, the best perenosin transporter previously reported.

#### pH dependent Cl^−^/NO_3_^−^ antiport

**Figure 5:**
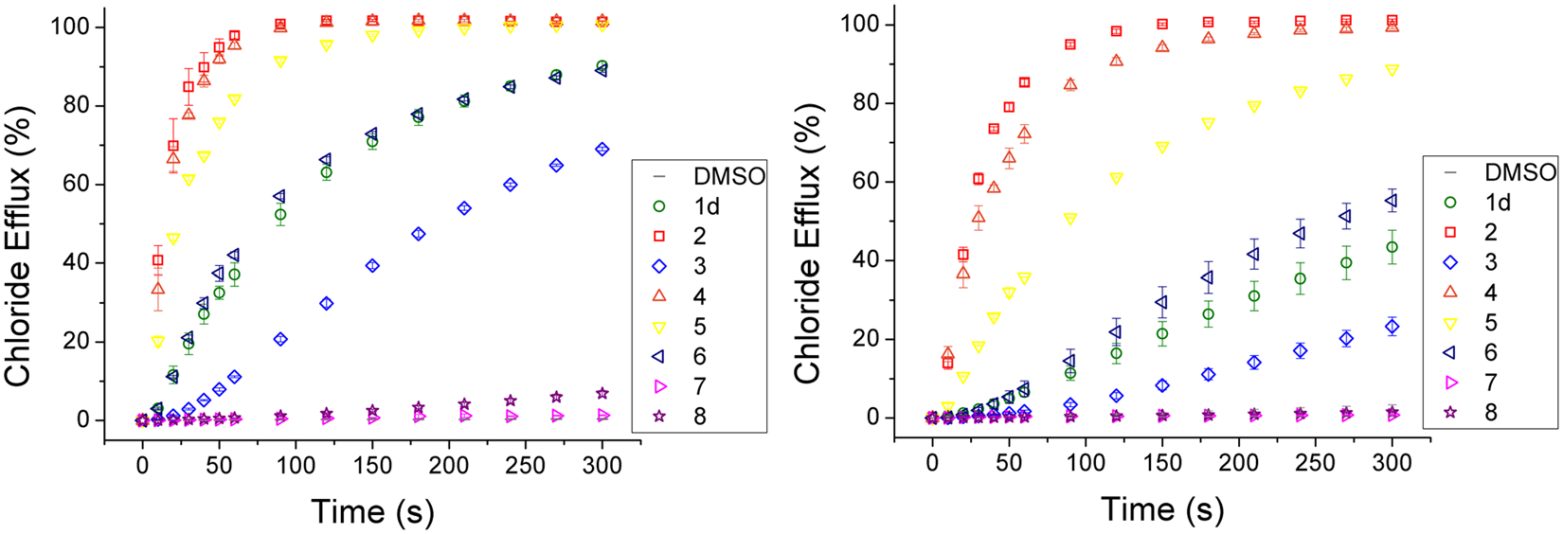
Chloride efflux achieved from compounds **1d** and **2**–**8** at 0.1 mol% loading from unilamellar vesicles loaded with NaCl (489 mM) suspended in NaNO_3_ (489 mM) buffered to pH 6.5 (left) and pH 7.2 (right) with sodium phosphate salts (5 mM). All data are the average of three repeats, performed using the same batch of vesicles, with error bars corresponding to the SD.

**Figure 6:**
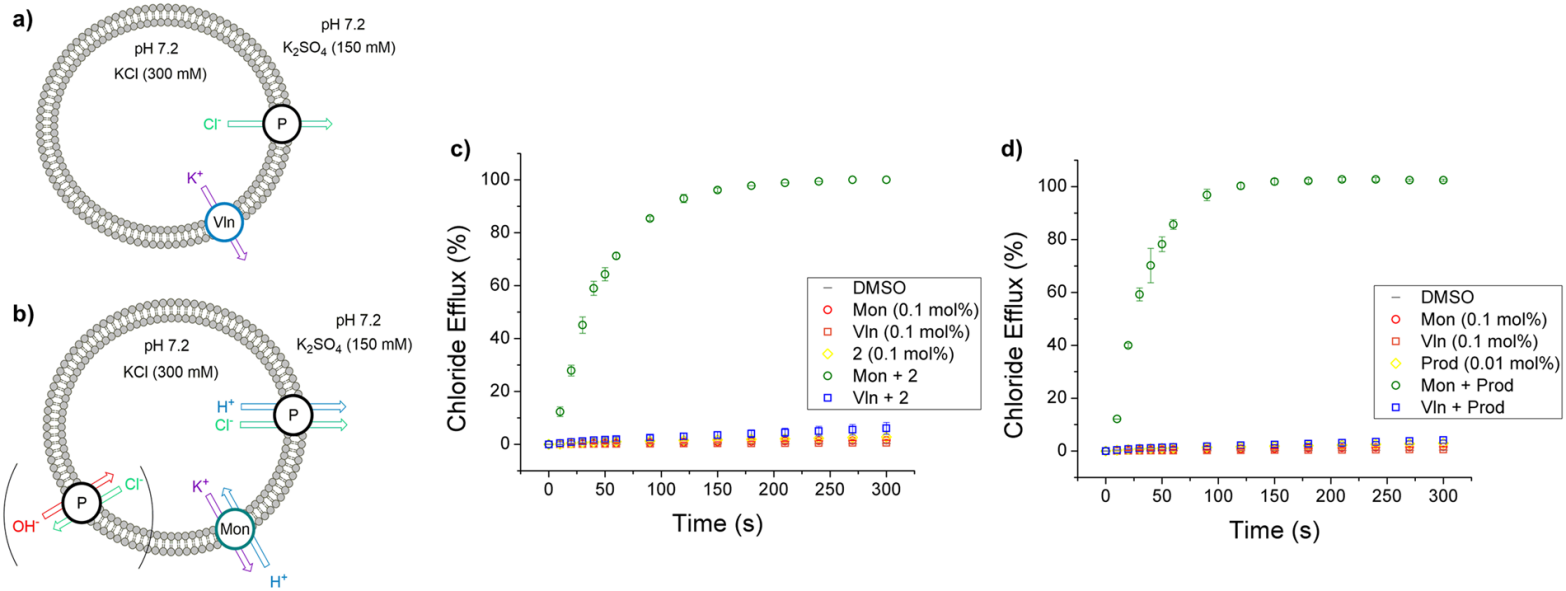
Schematic of the cationophore coupled KCl efflux assays measured by chloride ISE, from unilamellar POPC vesicles loaded with KCl (300 mM) and suspended in K_2_SO_4_ (150 mM), buffered to pH 7.2 with potassium phosphate salts (5 mM). (**a**) Overall KCl efflux induced from a combination of electrogenic K^+^ transport facilitated by valinomycin (Vln) (0.1 mol%) and electrogenic Cl^−^ transport facilitated by the transporter (T). (**b**) Overall KCl efflux induced from electroneutral transport processes; K^+^/H^+^ antiport by monensin (Mon) (0.1 mol%), and Cl^−^/H^+^ symport and Cl^−^/H^+^ antiport facilitated by transporter (T). (**c**) Chloride efflux facilitated by compound **2** (0.1 mol%). (**d**) Chloride efflux facilitated by prodigiosin (0.01 mol%). All data are the average of three repeats, performed using the same batch of vesicles, with error bars corresponding to the SD.

The external pH of cancer cells (pH 6.7^53^) is slightly acidic compared to healthy cells (pH 7.2–7.5^54^). Therefore, for the perenosins to show a degree of cytotoxic selectivity towards cancer cells over healthy cells it is desirable for cellular HCl cotransport to be more effective in the mildly acidic cancerous cell environment. To investigate the effect of pH on the chloride transport capabilities unilamellar vesicles were loaded with NaCl (489 mM) and suspended in NaNO_3_ (489 mM), and the internal and external solutions were buffered to pH 4.5, 6.5, 7.2 and 10 using various buffers (more detail can be found in the Supplementary Information). Compounds **1d** and **2**–**8** were then tested in a single point screen at 0.1 mol% loading (Fig. 5). At pH 4.5 the perenosins exist predominantly as the active protonated species and so all compounds show moderate to high chloride transport properties, whereas, there is no chloride transport observed at pH 10 (see Supplementary Information for results). For all compounds, except **7**, an increase in chloride transport is observed at pH 6.5 compared to pH 7.2, suggesting that these perenosins could potentially be more selective toward cancer cells (Fig. 6).

#### H^+^/Cl^−^ symport mechanism studies

The previously reported perenosins **1a**–**e** were shown to function in the same way as prodigiosin and facilitate chloride transport when protonated through H^+^/Cl^−^ symport (see Fig. 1), as demonstrated using a HPTS liposome-based assay^20, 35^. Herein, we employed two recently reported complementary cationophore coupled assays^19^ to unambiguously confirm H^+^/Cl^−^ symport facilitated by perenosins is a strictly inseparable process. Briefly, unilamellar vesicles were prepared with an internal solution of KCl (300 mM) and an external solution of K_2_SO_4_ (150 mM), buffered to pH 7.2 with potassium phosphate salts (5 mM). The naturally occurring cationophores valinomycin and monensin were either added alone, or in combination with a perenosin and the resulting chloride efflux, driven by a large chloride concentration gradient, was monitored using a chloride ISE. Valinomycin is selective for K^+^ and facilitates electrogenic transport which results in a net flow of charge across a lipid membrane, however, this does not directly affect the transmembrane pH gradient^57^. In contrast, monensin functions as an electroneutral transporter. Deprotonation of a carboxylic acid group occurs upon metal complexation allowing M^+^/H^+^ antiport (exchange) which results in an intra-vesicular pH change (illustrated in Fig. 6)^58^.

Compounds **1d** and **2**–**6** were tested in the cationophore coupled assays and, in all cases, chloride efflux was observed when coupled to monensin and only a very small amount, <5%, of chloride efflux occurred in the presence of valinomycin. The K^+^/H^+^ antiport facilitated by monensin is coupled to the H^+^/Cl^−^ cotransport facilitated by the receptors resulting in the overall KCl efflux observed. Therefore, the perenosins function as strict inseparable H^+^/Cl^−^ cotransporters and display the same mechanism as prodigiosin, requiring protonation of the free imine-N2 to facilitate anion transport. The protonated perenosins cannot back-diffuse into the liposome resulting in the ‘strict’ HCl co-transport.

#### Selectivity mechanism studies

The selectivity for Cl^−^>H^+^/OH^−^ transport of the perenosins was quantified using a modified NMDG-Cl assay^18^. Unilamellar vesicles loaded with HPTS (1 mM) and *N*-methyl-D-glucamine chloride (NMDG-Cl) (100 mM), buffered to pH 7 with HEPES (10 mM) were prepared. Free base NMDG (0.5 M) was added increasing the extravesicular solution to pH 8, and the dissipation of this gradient following the addition of receptors was monitored. Dissipation of pH gradient indicates H^+^/Cl^−^ symport (or the functionally equivalent OH^−^/Cl^−^ exchange) mediated by perenosins. Gramicidin D (Gra) is a proton channel added to facilitate H^+^ transport. The addition of oleic acid (OA), a naturally occurring fatty acid found in cell membranes, allowed the investigation of the enhancement in H^+^/Cl^−^ symport, in the presence of oleic acid. Anion transporters can assist the fatty acid flip-flop mechanism by taking on the role of a flippase for fatty acid anions via binding to the anionic carboxylate head group, masking the charge and allowing rapid passage of the head group through the lipid bilayer. This in turn accelerates the natural proton transport mediated by fatty acids in biological membranes^59^.

For all cases EC_50_ values were obtained from dose-response experiments and subsequent Hill analysis (see Supplementary Information). Chloride selectivity was then quantified to give a selectivity factor (S_G_), shown in Table 2, using the ratio of the EC_50_ values. From the previous cationophore coupled assays, it was evident that the capability of perenosins to facilitate electrogenic chloride transport is negligible, therefore an absence in selectivity for Cl^−^>H^+^/OH^−^ was expected. The selectivity for chloride (S_G_) is calculated by dividing EC_50_ in absence of Gra by EC_50 (Gra)_, and the S_G_ factors obtained for compounds **1d** and **2**–**6** are all marginally greater than one which indicates a minor increase in the rate of transport in the presence of Gra, attributed to a small degree of Cl^−^>H^+^/OH^−^ selectivity. Factor of enhancement (F_OA_) in the overall H^+^/Cl^−^ cotransport rate facilitated by OA is calculated by dividing EC_50_ in absence of Gra or OA by EC_50 (OA)_, a value greater than one suggests the receptor can assist the flip-flop of OA^−^ increasing pH dissipation. F_OA_ values for the overall H^+^/Cl^−^ cotransport rate were calculated for compounds **1d** and **2**–**6** in the presence of OA and are also shown in Table 2. Interestingly, compound **4** has a F_OA_ value of 8.6, indicating a significant increase in the overall transport rate in the presence of fatty acids. This suggests that the receptor coupled fatty acid flip-flop is enhancing the overall rate of H^+^/Cl^−^ cotransport (or OH^−^/Cl^−^ exchange). This acceleration in transport rate also suggests that the rate limiting step in the NMDG-Cl assay for compound **4** is the back-diffusion of the neutral transporter across the lipid bilayer, due to the lower log P value. In the presence of fatty acids, an alternative pathway that involves the diffusion of the fatty acid complex and free fatty acids could replace this step. Conversely, compounds **2**, **3** and **5** have F_OA_ values lower than one, indicating slower H^+^/Cl^−^ symport rate in the presence of fatty acids. This may be likely due to the competitive binding of the receptors to the carboxylate head group so inhibiting chloride binding and transport.

#### Other mechanistic studies

To further probe other aspects of transmembrane transport mechanism of perenosins, a variety of studies were employed. A Cl^−^/NO_3_^−^ antiport assay using an ISE method, as described previously in Cl^−^/NO_3_^−^ antiport at pH 7.2, was performed in POPC:cholesterol (7:3) vesicles; the addition of cholesterol decreases the fluidity and permeability of the membrane^60^ and this resulted in a lower percentage efflux facilitated by perenosins **1d** and **2**–**8** compared to the same assay done in POPC vesicles. Sulfate is a highly hydrophilic, strongly hydrated anion that is difficult to transport over lipid bilayers^61^; using the Cl^−^/NO_3_^−^ antiport assay and changing the external solution to Na_2_SO_4_ from NaNO_3_ showed no Cl^−^ efflux upon addition of **1d** and **2**–**8**, indicating that sulfate transport is not facilitated by the perenosins. Using the same conditions for sulfate transport, and adding a bicarbonate pulse at 120 s to give a final external bicarbonate concentration of 40 mM, allowed observation of potential Cl^−^/HCO_3_^−^ antiport elicited by **1d** and **2**–**8**; no Cl^−^ efflux was detected, presumably due to the basicity of bicarbonate anion preventing protonation of the imine-N2. Finally, preincorporation of **1d** and **2**–**6** into POPC vesicles followed by performing a similar NMDG-Cl assay, and subsequent dilution of the resulting lipid solution, investigated how perenosins behave in a lipid membrane environment. The H^+^/Cl^−^ cotransport rates at different lipid concentrations remained unchanged, indicating that the perenosins remained in the lipid bilayer over the course of the transport experiments rather than partitioning into the aqueous phase. See Supplementary Information for more details of results and experimental details of these assays.

### *In Vitro* Studies

#### Cell viability studies

Preliminary cell viability studies on the first generation perenosins conducted at the University of Southampton suggested cytotoxic selectivity for cancerous cells (MDA-MB-231 and MCF-7) over non-cancerous cells (MCF-10A)^35^. Herein, we carried out further *in vitro* studies at the University of Barcelona for the full library of perenosins. Single dose screening for cell viability using the methylthiazoletetrazolium (MTT) colorimetric assay over five different cell lines (cancer cells MCF-7, MDA-MB-231, A549 and SW620, and non-cancerous human cells MCF-10A) was conducted. After exposure to the perenosins (10 µM) for 24 h, interestingly, all perenosins exhibited selective cell viability decrease in human breast adenocarcinoma cell line (MDA-MB-231) over the other cell lines (see Supplementary Information).

Encouraged by these single dose screening results, dose response MTT assays were performed for the first time with compounds **2**–**8** and repeated for compounds **1a**–**e**, on MDA-MB-231 cells to obtain IC_50_ values (inhibitory concentration of 50% of cell population) systematically at 24 h and 72 h incubation periods, see Table 3. Among compounds **1a**–**1e**, **1d** (being the best transporter in liposome studies) is the most potent after 24 h exposure time. Additionally, perenosin **1d** remains the most potent compound after 72 h exposure time, and there is a slight decrease in cell viability by a factor of 1.2–1.7 for all the first generation of perenosins. Interestingly, compounds **2**–**8** exhibited an apparent structure-activity relationship for the onset of cytotoxic effect. There is negligible effect in cell viability (IC_50_ > 100 µM) after 24 h incubation time for perenosins with alkyl substituents at R_2_ (**2**–**4**, **6** and **8**). However, there is a significant late onset of affecting the cell viability after 72 h, with **6** being the most potent perenosin (IC_50_ (72 h): 4.2 µM) in MDA-MB-231 cells. While perenosin **5**, with pentyl substituent at R_3_, has similar cell viability effects as **1d**, bis-indole perenosin **7** with pentyl substituent at R_1_ has a moderate degree of late onset by a factor of 7.

**Table 3:** IC_50_ values (µM, with error from three repeats in brackets) of first and second generations of perenosins **1a–e** and **2**–**8** on the human breast adenocarcinoma (MDA-MCB-231) cell line with 24 h and 72 h exposure time.

Perenosin	IC_50_ (24 h)	IC_50_ (72 h)	F_A_^a^	Perenosin	IC_50_ (24 h)	IC_50_ (72 h)	F_A_^a^
**1a**	38.3 (1.6)	24.4 (0.5)	1.6	**2**	>100^b^	11.3 (0.8)	n/a^b^
**1b**	32.2 (1.9)	22.1 (2.7)	1.5	**3**	>100^b^	9.5 (0.1)	n/a^b^
**1c**	34.4 (1.7)	20.4 (0.3)	1.7	**4**	>100^b^	9.2 (2.2)	n/a^b^
**1d**	25.2 (4.0)	16.6 (0.7)	1.5	**5**	20.3 (0.5)	14.0 (0.3)	1.5
**1e**	38.4 (8.5)	32.7 (3.0)	1.2	**6**	>100^b^	4.2 (1.6)	n/a^b^
				**7**	73.8 (6.5)	11.0 (2.1)	6.7
				**8**	>100^b^	38.0 (1.9)	n/a^b^

^a^Factor of activity (F_A_) enhancement between 24 h and 72 h exposure time, IC_50_ (24 h) divided IC_50_ (72 h). ^b^Effect of cell viability is too weak (>100 µM) for IC_50_ dose response determination, hence factor of activity enhancement not applicable (n/a). Differences in IC_50_ values were observed between results of experiments reported previously from the University of Southampton and those reported here from the University of Barcelona, with a systematic error of ~20 µM. Whilst reason for these differences is unclear, it is important to note that the IC_50_ values reported here display the same trend as those previously reported^35^.

#### Cell-death mechanism studies

The specific late onset of affecting cell viability induced by perenosins is interesting, and to the best of our knowledge this phenomenon has not been observed for other anion transporters. Furthermore, this is governed by the unique structure-activity relationship displayed when bearing alkyl substituents at the R_2_ position (**2**–**4**, **6** and **8**). However, the apparent potency (IC_50_) of these perenosins from the MTT assay does not correlate well with the anion transport efficacy from liposome-based studies. To investigate this further, Annexin-V was used as a marker with flow cytometry analysis to evaluate apoptosis in MDA-MB-231 cells after treatments with **2** (best transporter) and **6** (most potent) at their respective IC_50_ values. It is known that synthetic transporters can disrupt ion homeostasis in cells and induce cell death via an apoptotic pathway^3, 22, 32, 63, 64^.

**Figure 7:**
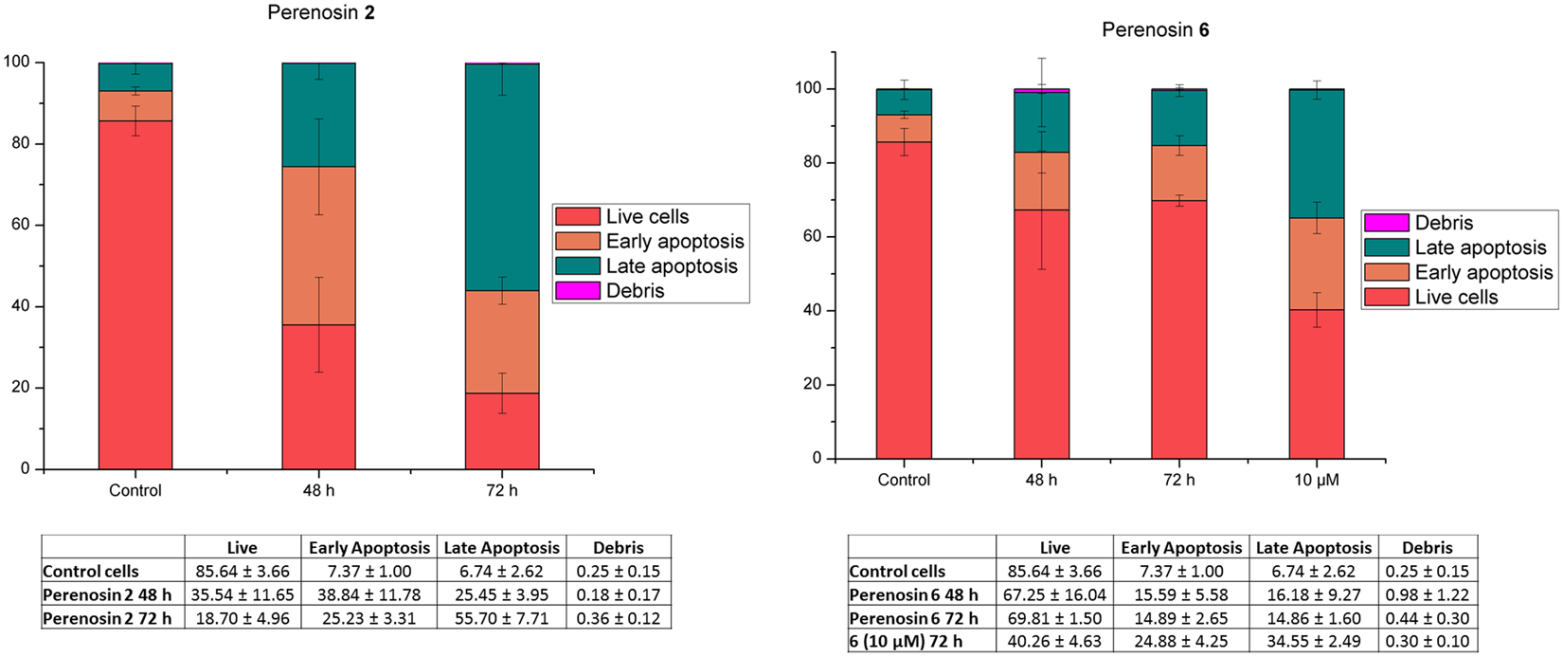
Results of Annexin-V flow cytometric assay on MDA-MB-231 cells, showing the distributions of cells present in the early and late apoptotic populations when treated with IC_50_ values of perenosins **2** (left) (11 µM) and **6** (right) (4 µM).

As shown in Fig. 7 (see Supplementary Information for details), perenosin **2** clearly induces cell death by apoptosis, with a higher population of early apoptotic cells after 48 h exposure time and then transitioning to greater proportion of cells in late apoptosis after 72 h. Unexpectedly, the most potent perenosin **6** (from MTT cell viability assay) resulted in slight increase in apoptotic cells at the IC_50_ value after 48 h and 72 h. To confirm **6** induces apoptosis in cells, a higher concentration dose of **6** (10 µM) was used and demonstrated an increase in both early and late apoptotic cell populations. From this Annexin-V assay, we conclude that the best transporter **2** is more cytotoxic in inducing cell apoptosis, in contrast to the most potent compound **6** from the MTT assay. Therefore, the IC_50_ value of compound **6** obtained from MTT cell viability studies may be due to a combination of cell cycle arrest and apoptosis. This also suggests that other cellular effects can be induced by **6** such as DNA intercalation which disturbs the normal cell cycle, and not solely disruption of ion homeostasis. It should be mentioned that perenosins hydrolyse steadily (as shown in our preliminary investigation in aqueous buffer (see Supplementary Information)), due to the presence of the imine moiety.

## Conclusions

The design of anion transporters often involves having a suitable alkyl chain to achieve optimal log P; however, we have also demonstrated the importance of positioning the alkyl chains to significantly enhance the anion binding and transport properties of the perenosins. While perenosin **1d** emerged as the best compound from the original report (EC_50_ (ISE) 0.122 mol% and (HPTS) 0.0107 mol%), compound **2** shows a remarkable enhancement in the transmembrane anion transport (EC_50_ (ISE) 0.0043 mol% and (HPTS) 0.00062 mol%). This was achieved by changing the pentyl chain from position R_1_ to R_2_, to introduce the encapsulation/dehydration effects of the anion binding site by an alkyl chain. By using the cationophore-coupled ISE and gramicidin-coupled HPTS assays, the perenosins have been confirmed to function predominantly by the electroneutral mechanism of strictly inseparable H^+^/Cl^−^ symport, in a similar fashion to prodigiosin. Additionally, the Cl^−^>H^+^/OH^−^ selectivity as well as their transport capabilities in the presence of fatty acids have been investigated.

*In vitro* studies of our full library of perenosins demonstrated a decrease in cell viability of the human breast adenocarcinoma (MDA-MB-231) cell line over other non-cancerous and cancer cells. More interestingly, the new perenosins (**2**–**4**, **6** and **8**) bearing an alkyl substituent at the R_2_ position serendipitously exhibited a distinctive late onset of cytotoxicity effect observed between 24 h and 72 h from the cell viability studies using MTT assays. Additionally, the cell death mechanism was evaluated using Annexin-V assays to confirm the apoptotic pathway as well as possible cell cycle arrest mechanism. The excellent anion transport efficacy in liposomes and cytotoxicity observed towards human breast adenocarcinoma cells demonstrated that the perenosins are potentially suitable candidates as anticancer therapeutics.

## Electronic supplementary material

Supplementary information
