# Supplementary material for: Indole-based perenosins as highly potent HCl transporters and potential anti-cancer agents
Source: Sci Rep. 2017 Aug 24;7:9397. doi: 10.1038/s41598-017-09645-9 (PMC5570892; doi:10.1038/s41598-017-09645-9)
Supplement: Supplementary file 1 — Supplementary information [file 41598_2017_9645_MOESM1_ESM.pdf]

Supplementary Information

Indole-based perenosins as highly potent HCl transporters and potential anti-cancer agents

Laura. A. Jowett,[1] Ethan. N. W. Howe,[1] Vanessa Soto-Cerrato[2], Wim Van Rossom,[3] Ricardo Pérez-Tomás[2], and Philip. A. Gale[1,*]

[1] School of Chemistry (F11), The University of Sydney, NSW 2006, Australia

[2] Department of Pathology and Experimental Therapeutics, Cancer Cell Biology Research Group, University of Barcelona, Barcelona, Spain

[3] Chemistry, University of Southampton, Highfield, Southampton, UK SO17 1BJ.

* E-mail: philip.gale@sydney.edu.au

Contents

S1. General ... 2

S2. Overview of Anionophores ... 3

S3. Synthesis ... 3

S4. HRMS, ^{1}H NMR and ^{13}C NMR Spectra ... 8

S5. X-ray Crystallography ... 28

S6. pK_a Studies ... 32

S6.1 pK_a Studies in Aqueous Solution ... 32

S6.2 pK_a Studies in Vesicles ... 35

S7. Stability Studies ... 40

S7.1 Stability in Aqueous Solution ... 40

S7.2 Stability in Vesicles ... 47

S8. ^{1}H NMR Titration Binding Studies ... 51

S8.1. Equipment and Sample Preparation ... 51

S8.2. Titration Procedure ... 51

S8.3. Titration Data Fitting ... 51

S.8.4 ^{1}HNMR Titration Spectra and Fitted Binding Isotherms ... 52

S9. Dilution Studies ... 68

S10. Anion Transport Studies ... 72

S10.1. General Procedure for the ISE Assay ... 72

S10.2. Cl^-/NO_3^- ISE Assay - pH Tests ... 73

S10.3. Cl^-/NO_3^- ISE Assay - Hill Analysis ... 75

S10.4. Cl^-/NO_3^- ISE Assay - POPC:Cholesterol (7:3) Vesicles ... 78

S10.5. Cl^-/SO_4^{2-} ISE Assay ... 79

S10.6. Cl^-/HCO_3^- ISE Assay ... 80

S10.7 KCl/K_2SO_4 ISE Assay - Coupling to Monensin or Valinomycin ... 80

S10.8. General Procedure for the HPTS Assay ... 82

S10.9. HPTS Assay - NMDG-Cl ... 83

S10.10. HPTS Assay - TBAOH ... 89

S10.11. HPTS Assay - Fatty Acids ... 90

S10.12. Preincorporated Leaching Assay ... 93

S11. Cell Studies ... 98

S11.1 Cell Lines and Culture Conditions ... 98

S11.2 Cell Viability Assays ... 98

S11.3 Apoptosis Evaluation through Annexin-V Assay ... 102

References ... 103

S1. General

All chemicals and solvents used were purchased from commercial sources (Alfa Aesar, Fisher Scientific, Matrix Scientific and Sigma Aldrich). Commercially available reagent grade chemicals were used without further purification unless otherwise specified. All deuterated solvents for NMR were purchased from Cambridge Isotope Laboratories.

NMR experiments were conducted using a 5mm ID NMR sample tube. ^{1}H and ^{13}C NMR spectra were recorded on Bruker Avance AVII400 and AVIIIHD400 FT-NMR spectrometers with 5mm BBFO z-gradient probes, operating at a frequency of 400 MHz for ^{1}H NMR and 101 MHz for ^{13}C NMR respectively. NMR spectra were recorded at 298 K and samples were dissolved in the stated solvents and chemical shifts were referenced internally to residual solvent resonances; CD_3CN, ^{1}H = 1.94 ppm; $CDCl_3$, ^{1}H = 7.26 ppm; $(CD_3)_2SO$, ^{1}H = 2.50 ppm; $CDCl_3$, ^{13}C = 77.16 ppm; $(CD_3)_2SO$, ^{13}C = 39.52 ppm. Signals are recorded in chemical shift (δ in ppm from residual solvent resonances referenced to tetramethylsilane, TMS), multiplicity, coupling constants (*J* in Hz) and relative integral in that order. Multiplicity abbreviations used are: s, singlet; d, doublet; t, triplet; q, quartet; quin, quintet; m, multiplet; br s, broad singlet; br d, broad doublet; dd, doublet of doublets; ddd, doublet of doublets of doublets; td, triplet of doublets. Uncertainties in chemical shifts at 298 K are typically ± 0.01 ppm for ^{1}H and ± 0.05 ppm for ^{13}C. Coupling constants (*J*) have an uncertainty of ± 0.1 ppm for ^{1}H-^{1}H coupling. NMR data were processed using ACD labs software.

For low resolution mass spectrometry, samples were analysed using a Waters (Manchester, UK) TQD mass spectrometer equipped with a triple quadrupole analyser. Samples were introduced to the mass spectrometer via an Acquity H-Class quaternary solvent manager (with TUV detector at 254 nm, sample and column manager). Ultra-performance liquid chromatography was undertaken via a Waters BEH C18 column (50 mm × 2.1 mm 1.7 µm). Mobile phase: gradient 20% acetonitrile (0.2% formic acid) to 100% acetonitrile (0.2% formic acid) in five minutes at a flow rate of 0.6 mL/min. Low resolution mass spectra were recorded using positive/negative ion electrospray ionization.

For high resolution mass spectrometry, samples were analysed using a MaXis (Bruker Daltonics, Bremen, Germany) mass spectrometer equipped with a Time of Flight (TOF) analyser. Samples were introduced to the mass spectrometer via a Dionex Ultimate 3000 autosampler and an uHPLC pump. Ultra performance liquid chromatography was undertaken via a Waters UPLC BEH C18 (50 mm × 2.1 mm 1.7 µm) column. Mobile phase: gradient 20% acetonitrile (0.2% formic acid) to 100% acetonitrile (0.2% formic acid) in five minutes at a flow rate of 0.6 mL/min. High resolution mass spectra were recorded using positive/negative ion electrospray ionization.

UV-Vis spectra were recorded on an Agilent Cary 100 UV-Vis spectrophotometer, equipped with a temperature-controlled multicell holder and a temperature control unit.

S2. Overview of Anionophores

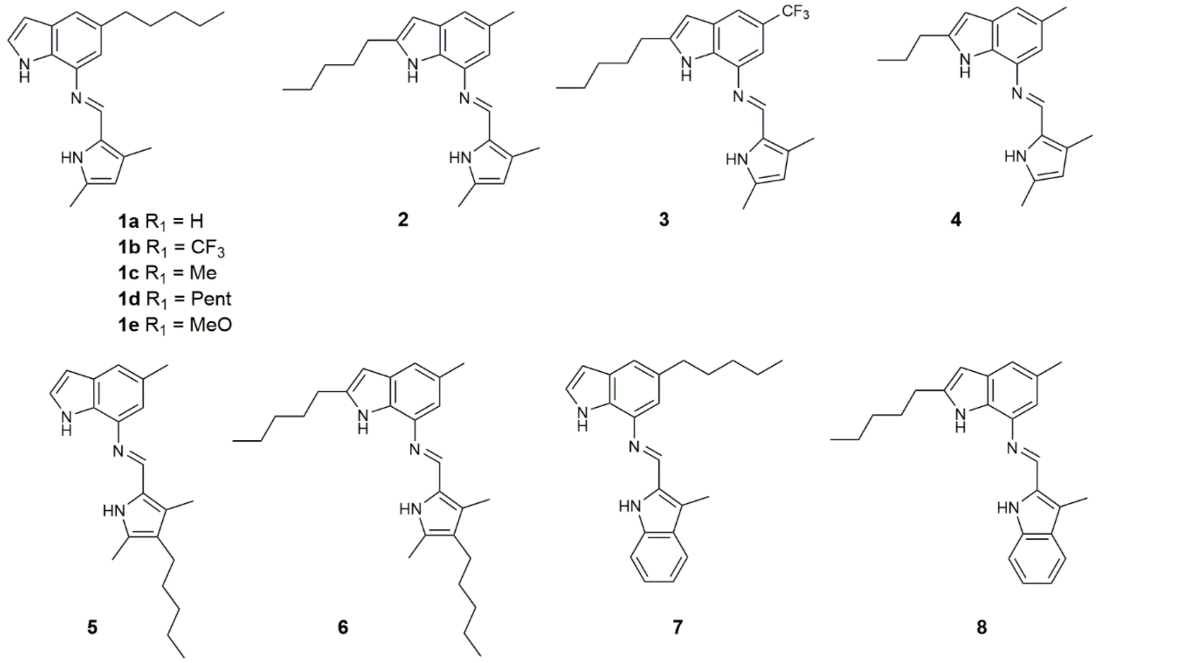

S3. Synthesis

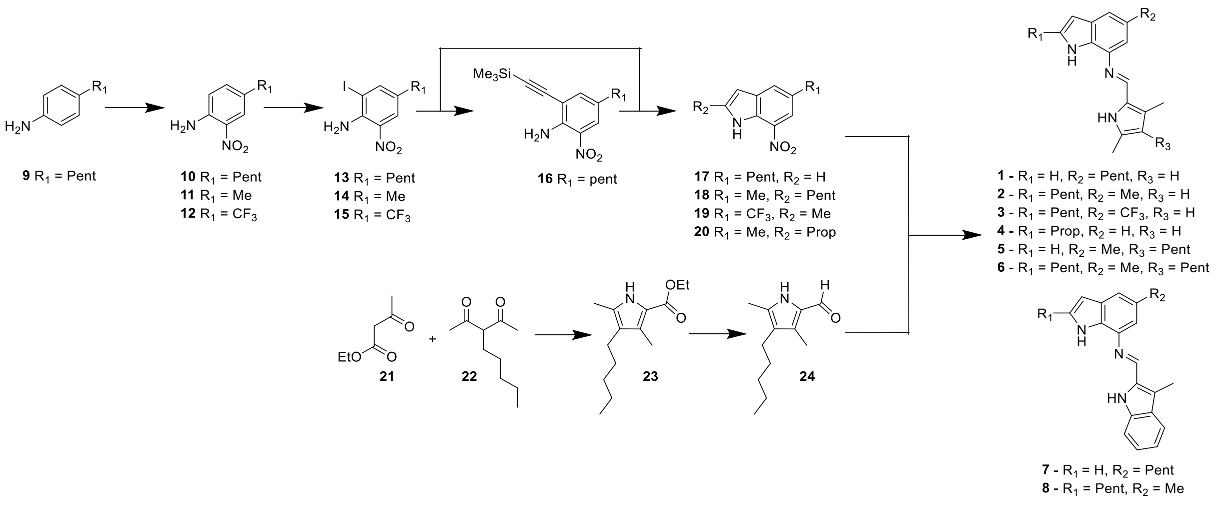

Figure S1. Overview of the synthesis scheme for anionophores **1-8**.

Compounds **11**, **12**, **21** and **22** were commercially available.

Compounds **1a-e**[1], **9**[1], **10**[1], **13**[1], **14**[1], **15**[2], **16**[1], **17**[1], **18**[3], **23**[4] and **24**[5,6] were synthesised and characterised using previously reported procedures.

Synthesis of **2**

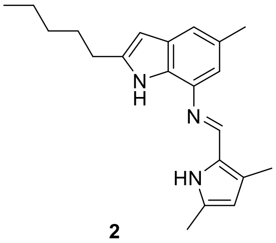

2

2-pentyl-5-methyl-7-nitro-1*H*-indole (0.367 g, 1.49 mmol) was dissolved in ethanol (30 mL) and Pd/C (0.150 g, 10 mol%) was added. The reaction mixture was degassed with N_2, placed under a hydrogen atmosphere and stirred vigorously in the dark at room temperature for 5 hrs. After this time the reaction was filtered through a glass microfibre filter to separate the catalyst. To the orange filtrate, magnesium sulfate (0.25 g, 2.1 mmol) and 3,5-dimethylpyrrole-2-carboxyaldehyde (0.185 g, 1.50 mmol) were added and left to stir at room temperature in the dark overnight. The reaction mixture was filtered and run through an SCX-2 column using the catch and release method (eluent: methanol, wash out: NH_3 7N in MeOH), then the solvent removed. Purification by column chromatography (silica, eluent: hexane-ethyl acetate-triethylamine, 80-20-3) and recrystallization from hexane gave a yellow solid (0.308 g, 64 %). **^{1}H NMR** (400 MHz, DMSO-*d*$_6$) δ ppm 10.90 (br s, 1 H) 10.42 (s, 1 H) 8.42 (s, 1 H) 6.96 (s, 1 H) 6.63 (s, 1 H) 6.03 (d, *J*=1.83 Hz, 1 H) 5.78 (s, 1 H) 2.71 (t, *J*=7.52 Hz, 2 H) 2.35 (s, 3 H) 2.26 (s, 3 H) 2.21 (s, 3 H) 1.68 (quin, *J*=7.21 Hz, 2 H) 1.28 - 1.36 (m, 4 H) 0.88 (t, *J*=6.80 Hz, 3 H); **^{13}C NMR** (101 MHz, DMSO-*d*$_6$) δ ppm 146.29, 140.28, 136.45, 133.09, 129.62, 129.40, 127.64, 126.76, 126.07, 115.43, 110.20, 109.00, 98.02, 30.99, 28.76, 27.62, 21.93, 21.38, 13.95. 12.92. 10.79; **HRMS** (ESI$^+$) calcd for $C_{21}H_{27}N_3$ [M+H]$^+$: 322.2278, found *m/z* 322.2285; **MS** (ESI$^+$) m/z 332.3 [M + H]$^+$

Synthesis of **3**

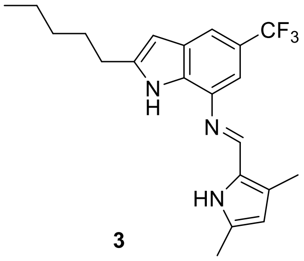

3

2-pentyl-5-trifluoromethyl-7-nitro-1*H*-indole (0.080 g, 0.27 mmol) was dissolved in ethanol (30 mL) and Pd/C (0.048 g, 10 mol%) was added. The reaction mixture was degassed with N_2, placed under a hydrogen atmosphere and stirred vigorously in the dark at room temperature for 5 hrs. After this time the reaction was filtered through a glass microfibre filter to separate the catalyst. To the orange filtrate, magnesium sulfate (0.25 g, 2.1 mmol) and 3,5-dimethylpyrrole-2-carboxyaldehyde (0.119 g, 0.97 mmol) were added and left to stir at room temperature in the dark overnight. The reaction mixture was filtered and run through an SCX-2 column using the catch and release method (eluent: methanol, wash out: NH_3 7N in MeOH), then the solvent removed. Purification by column chromatography (silica, eluent: hexane-ethyl acetate-triethylamine, 80-20-3) and recrystallization from hexane gave an orange solid (0.057 g, 57 %). **^{1}H NMR** (400 MHz, DMSO-*d*$_6$) δ ppm 11.06 (br s, 1 H) 11.01 (br s, 1 H) 8.51 (s, 1 H) 7.55 (s, 1 H) 7.07 (s, 1 H) 6.31 (s, 1 H) 5.82 (s, 1 H) 2.77 (t, *J*=7.58 Hz, 2 H) 2.28 (s, 3 H) 2.25 (s, 3 H) 1.65 - 1.77 (m, 2 H) 1.31 - 1.36 (m, 4 H) 0.86 - 0.91 (m, 3 H); **^{13}C NMR** (101 MHz, DMSO-*d*$_6$) δ ppm 148.06, 142.47, 137.15, 133.90, 132.52, 128.34, 127.41, 126.48, 122.44, 110.40, 110.34, 103.76, 103.72, 99.50, 30.75, 28.42, 27.32, 21.72, 13.75, 12.76, 10.65; **HRMS** (ESI$^+$) calcd for $C_{21}H_{25}F_3N_3$ [M+H]$^+$: 376.1995, found *m/z* 376.2001; **MS** (ESI$^+$) m/z 376.4 [M + H]$^+$

Synthesis of **4**

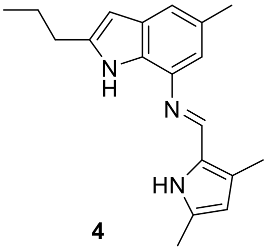

4

2-propyl-5-methyl-7-nitro-1*H*-indole (0.813 g, 3.72 mmol) was dissolved in ethanol (60 mL) and Pd/C (0.050 g, 10 mol%) was added. The reaction mixture was degassed with N_2, placed under a hydrogen atmosphere and stirred vigorously in the dark at room temperature for 5 hrs. After this time the reaction was filtered through a glass microfibre filter to separate the catalyst. To the pale orange filtrate, magnesium sulfate (0.25 g, 2.1 mmol) and 3,5-dimethylpyrrole-2-carboxyaldehyde (0.504 g, 4.09 mmol) were added and left to stir at room temperature in the dark overnight. The reaction mixture was filtered

and run through an SCX-2 column using the catch and release method (eluent: methanol, wash out: NH_3 7N in MeOH), then the solvent removed. Purification by column chromatography (silica, eluent: hexane-ethyl acetate-triethylamine, 80-20-3) and recrystallization from hexane gave a yellow solid (0.356 g, 33 %). **^{1}H NMR** (400 MHz, DMSO-*d*$_6$) δ ppm 10.89 (br s, 1 H) 10.41 (br s, 1 H) 8.42 (s, 1 H) 6.96 (s, 1 H) 6.64 (s, 1 H) 6.03 (s, 1 H) 5.78 (s, 1 H) 2.68 (t, J=7.52 Hz, 2 H) 2.35 (s, 3 H) 2.25 (s, 3 H) 2.21 (s, 2 H) 1.64 - 1.74 (m, 2 H) 0.93 (t, *J*=7.34 Hz, 3 H); **^{13}C NMR** (101 MHz, DMSO-*d*$_6$) δ ppm 146.26, 140.07, 136.44, 133.11, 129.67, 129.40, 127.65, 126.78, 126.08, 115.45, 110.21, 108.98, 98.13, 29.73, 22.34, 21.38, 13.76, 12.93, 10.78; **HRMS** (ESI$^+$) calcd for $C_{19}H_{24}N_3$ [M+H]$^+$: 294.1965, found *m/z* 294.1971; **MS** (ESI$^+$) m/z 294.3 [M + H]$^+$

Synthesis of **5**

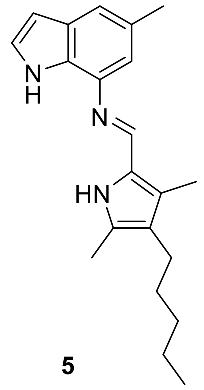

5

5-methyl-7-nitro-1*H*-indole (0.334 g, 1.90 mmol) was dissolved in ethanol (20 mL) and Pd/C (0.053 g, 10 mol%) was added. The reaction mixture was degassed with N_2, placed under a hydrogen atmosphere and stirred vigorously in the dark at room temperature for 5 hrs. After this time the reaction was filtered through a glass microfibre filter to separate the catalyst. To the orange filtrate, magnesium sulfate (0.25 g, 2.1 mmol) and 3,5-dimethyl-4-pentyl-pyrrole-2-carboxyaldehyde (0.104 g, 0.54 mmol) were added and left to stir at room temperature in the dark overnight. The reaction mixture was filtered and run through an SCX-2 column using the catch and release method (eluent: methanol, wash out: NH_3 7N in MeOH), then the solvent removed. Purification by column chromatography (silica, eluent: hexane-ethyl acetate-triethylamine, 80-20-3) and recrystallization from methanol gave a yellow solid (0.115 g, 66 %). **^{1}H NMR** (400 MHz, DMSO-*d*$_6$) δ ppm 10.82 (br s, 1 H) 10.73 (br s, 1 H) 8.45 (s, 1 H) 7.25 (t, *J*=2.69 Hz, 1 H) 7.08 (s, 1 H) 6.73 (d, *J*=1.20 Hz, 1 H) 6.32 (dd, *J*=3.00, 2.02 Hz, 1 H) 2.37 (s, 3 H) 2.33 (t, *J*=7.40 Hz, 2 H) 2.21 (s, 3 H) 2.17 (s, 3 H) 1.41 (quin, *J*=7.24 Hz, 2 H) 1.23 - 1.34 (m, 4 H) 0.85 - 0.90 (m, 3 H); **^{13}C NMR** (101 MHz, DMSO-*d*$_6$) δ ppm 146.36, 137.13, 130.33, 129.92, 128.65, 127.98, 125.80, 124.83, 124.68, 120.91, 116.02, 109.60, 100.60, 31.00, 30.15, 23.40, 22.02, 21.38, 13.98, 11.14, 9.03; **HRMS** (ESI$^+$) calcd for $C_{21}H_{28}N_3$ [M+H]$^+$: 322.2278, found *m/z* 322.2282; **MS** (ESI$^+$) m/z 322.4 [M + H]$^+$

Synthesis of **6**

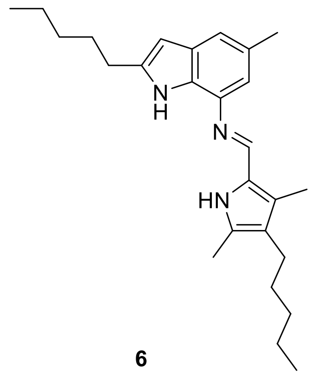

6

2-pentyl-5-methyl-7-nitro-1*H*-indole (0.503 g, 2.03 mmol) was dissolved in ethanol (35 mL) and Pd/C (0.036 g, 10 mol%) was added. The reaction mixture was degassed with N_2, placed under a hydrogen atmosphere and stirred vigorously in the dark at room temperature for 5 hrs. After this time the reaction was filtered through a glass microfibre filter to separate the catalyst. To the orange filtrate, magnesium sulfate (0.25 g, 2.1 mmol) and 3,5-dimethyl-4-pentyl-pyrrole-2-carboxyaldehyde (0.550 g, 2.85 mmol) were added and left to stir at room temperature in the dark overnight. The reaction mixture was filtered and run through an SCX-2 column using the catch and release method (eluent: methanol, wash out: NH_3 7N in MeOH), then the solvent removed. Purification by column chromatography (silica, eluent: hexane-ethyl acetate-triethylamine, 80-20-3) and recrystallization from hexane gave a brown/yellow, oily solid (0.204 g, 26 %). **^{1}H NMR** (500 MHz, DMSO-*d*$_6$) δ ppm 10.75 (br s, 1 H) 10.40 (br s, 1 H) 8.41 (s, 1 H) 6.95 (s, 1 H) 6.62 (s, 1 H) 6.02 (s, 1 H) 2.70 (t, *J*=7.56 Hz, 2 H) 2.31 - 2.37 (m, 5 H) 2.22 (s, 3 H) 2.17 (s, 3 H) 1.64 - 1.72 (m, 2 H) 1.37 - 1.45 (m, 2 H) 1.22 - 1.32 (m, 8 H) 0.87 (t, *J*=7.05 Hz, 6 H); **^{13}C NMR** (101 MHz, DMSO-*d*$_6$) δ ppm 140.24, 136.66, 131.99, 130.18, 129.38, 128.34, 127.65, 125.72, 124.57, 120.84, 115.28, 108.99, 98.05, 30.99, 30.15, 28.75, 27.61, 23.40, 22.03,

21.92, 21.37, 13.99, 13.94, 11.88, 9.03; **HRMS** (ESI+) calcd for $C_{26}H_{38}N_3$ $[M+H]^+$: 392.3060, found *m/z* 392.3057; **MS** (ESI+) m/z 392.5 $[M + H]^+$

Synthesis of **7**

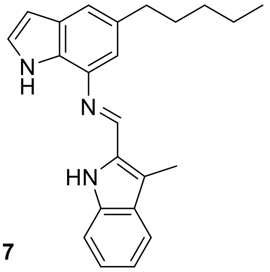

7

5-pentyl-7-nitro-1*H*-indole (0.796 g, 3.43 mmol) was dissolved in ethanol (30 mL) and Pd/C (0.118 g, 10 mol%) was added. The reaction mixture was degassed with N_2, placed under a hydrogen atmosphere and stirred vigorously in the dark at room temperature for 5 hrs. After this time the reaction was filtered through a glass microfibre filter to separate the catalyst. To the orange filtrate, magnesium sulfate (0.25 g, 2.1 mmol) and 3-methylindole-2-carboxyaldehyde (0.294 g, 1.70 mmol) were added and left to stir at room temperature in the dark overnight. The reaction mixture was filtered and run through an SCX-2 column using the catch and release method (eluent: methanol, wash out: NH_3 7N in MeOH), then the solvent removed. Purification by column chromatography (silica, eluent: hexane-ethyl acetate-triethylamine, 80-20-3) and recrystallization from methanol gave a yellow solid (0.329 g, 79 %). **^{1}H NMR** (400 MHz, DMSO-d_6) δ ppm 11.19 (s, 1 H) 10.98 (s, 1 H) 8.78 (s, 1 H) 7.63 (d, *J*=7.95 Hz, 1 H) 7.44 (d, *J*=8.19 Hz, 1 H) 7.36 (t, *J*=2.69, 1.16 Hz, 1 H) 7.25 (td, J=7.58, 1 H) 7.22 (s, 1 H) 7.00-7.08 (m, 2 H) 6.40 (dd, *J*=2.93, 1.96 Hz, 1 H) 2.68 (t, *J*=7.64 Hz, 2 H) 2.55 (s, 3 H) 1.60-1.72 (m, 2 H) 1.27 - 1.40 (m, 4 H) 0.85 - 0.90 (m, 3 H); **^{13}C NMR** (101 MHz, DMSO-d_6) δ ppm 147.28, 137.10, 135.32, 133.48, 132.90, 130.56, 128.89, 128.57, 125.27, 124.48, 119.77, 118.93, 117.26, 116.93, 111.54, 109.35, 101.03, 35.62, 31.55, 31.08, 22.07, 14.08, 8.41; **HRMS** (ESI+) calcd for $C_{23}H_{26}N_3$ $[M+H]^+$: 344.2121, found *m/z* 344.2130; **MS** (ESI+) m/z 344.4 $[M + H]^+$

Synthesis of **8**

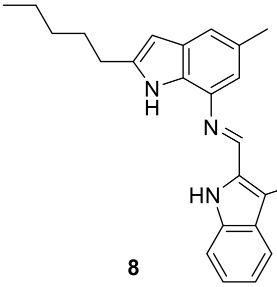

8

2-pentyl-5-methyl-7-nitro-1*H*-indole (0.785 g, 3.19 mmol) was dissolved in ethanol (30 mL) and Pd/C (0.070 g, 10 mol%) was added. The reaction mixture was degassed with N_2, placed under a hydrogen atmosphere and stirred vigorously in the dark at room temperature for 5 hrs. After this time the reaction was filtered through a glass microfibre filter to separate the catalyst. To the orange filtrate, magnesium sulfate (0.25 g, 2.1 mmol) and 3-methylindole-2-carboxyaldehyde (0.296 g, 1.71 mmol) were added and left to stir at room temperature in the dark overnight. The reaction mixture was filtered and run through an SCX-2 column using the catch and release method (eluent: methanol, wash out: NH_3 7N in MeOH), then the solvent removed. Purification by column chromatography (silica, eluent: hexane-ethyl acetate-triethylamine, 80-20-3) and recrystallization from methanol gave a yellow solid (0.321 g, 53 %). **^{1}H NMR** (400 MHz, DMSO-d_6) δ ppm 11.11 (s, 1 H) 10.63 (s, 1 H) 8.91 (s, 1 H) 7.63 (d, *J*=7.83 Hz, 1 H) 7.46 (d, *J*=8.19 Hz, 1 H) 7.24 (ddd, *J*=8.19, 7.03, 1.16 Hz, 1 H) 7.07 (s, 1 H) 7.05 (ddd, *J*=8.20, 7.00, 1.00 Hz, 1 H) 6.89 (d, *J*=0.86 Hz, 1 H) 6.08 (d, *J*=2.08 Hz, 1 H) 2.74 (t, *J*=7.60 Hz, 2 H) 2.54 (s, 3 H) 2.39 (s, 3 H) 1.71 (quin, *J*=7.40 Hz, 2 H) 1.30 - 1.40 (m, 4 H) 0.86 - 0.89 (m, 3 H); **^{13}C NMR** (101 MHz, DMSO-d_6) δ ppm 147.39, 140.87, 137.25, 135.05, 133.04, 130.37, 129.91, 128.76, 127.95, 124.68, 119.96, 119.15, 117.20, 117.12, 111.79, 109.29, 98.37, 31.23, 28.94, 27.90, 22.15, 21.55, 14.17, 8.55; **HRMS** (ESI+) calcd for $C_{24}H_{28}N_3$ $[M+H]^+$: 358.2278, found *m/z* 358.2275; **MS** (ESI+) m/z 358.4 $[M + H]^+$

Synthesis of **19**

2-nitro-4-trifluoromethyl-6-iodo aniline (**15**) (0.365 g, 1.10 mmol), heptyne (0.2 mL, 1.5 mmol), $Pd(PPh_3)_2Cl_2$ (0.02 g, 10 mol%) and copper iodide (0.01 g, 20 mol%) were dissolved in anhydrous

DMF (5 mL). The reaction was cooled to 0 ˚C and diethylamine (0.15 mL, 1.5 mmol) was added causing the reaction mixture to turn a dark red to brown colour. The reaction was allowed to warm to room temperature and left to stir for 1.5 hrs after which complete consumption of starting material was observed. Sodium hydroxide pellets (0.787 g, 10 mmol) were added and the reaction mixture was heated to 140 ˚C for 3 hrs. After cooling, water (50 mL) was added and this was washed with DCM (3 x 100 mL). The organic phase was washed with water (3 x 100 ml), dried over $MgSO_4$ and solvent removed under vacuum. Purification by column chromatography (silica, eluent: hexane-ethyl acetate, 4-1) gave a yellow solid (**19**) (0.139 g, 46 %). **^{1}H NMR** (400 MHz, DMSO-d_6) δ ppm 12.12 (br s, 1 H) 8.33 (s, 1 H) 8.19 (d, *J*=0.98 Hz, 1 H) 6.62 (s, 1 H) 2.86 (t, *J*=7.64 Hz, 2 H) 1.67 - 1.76 (m, 2 H) 1.31 - 1.37 (m, 4 H) 0.87 - 0.91 (m, 3 H);); **^{13}C NMR** (101 MHz, DMSO-d_6) δ ppm 147.18, 133.06, 131.69, 129.63, 125.65, 123.57, 119.25, 113.52, 101.26, 30.91, 28.33, 27.18, 21.87, 13.91; **HRMS** (ESI$^+$) calcd for $C_{14}H_{14}F_3N_2O_2$ [M-H]$^-$: 299.1013, found *m/z* 299.1007; **MS** (ESI$^-$) 299.8 [M – H]$^-$

Synthesis of **20**

6-iodo-4-methyl-2nitroanlinine (**14**) (2.001 g, 7.19 mmol), $Pd(PPh_3)_2Cl_2$ (0.505 g, 10 mol%), copper iodide (0.260 g, 20 mol%) and pentyne (1.1 mL, 10.7 mmol) were stirred under N_2 and anhydrous DMF (20 mL) was added. The mixture was cooled to 0 ˚C and diethylamine (1.1 mL, 10.7 mmol) was added dropwise. The reaction was allowed to proceed under N_2 at room temperature for 3 hrs, after this time complete consumption of the starting material was seen so sodium hydroxide pellets (1.44 g, 35.9 mmol) were added and the reaction mixture was heated to 140 ˚C and left overnight. After cooling, water (50 mL) was added and the aqueous phase was washed with DCM (3 x 100 mL), then the organic phase was washed with water (5 x 100 mL), dried using magnesium sulfate and solvent removed under vacuum. Purification by column chromatorgraphy (silica, eluent: hexane-ethyl acetate, 9-1) gave a solid yellow (**20**) (0.813 g, 52 %). **^{1}H NMR** (400 MHz, DMSO-d_6) δ ppm 11.52 (br s, 1 H) 7.82 (s, 1 H) 7.73 (s, 1 H) 6.34 (s, 1 H) 2.78 (t, *J*=7.52 Hz, 2 H) 2.44 (s, 3 H) 1.71 (sxt, *J*=7.38 Hz, 2 H) 0.94 (t, *J*=7.34 Hz, 3 H;); **^{13}C NMR** (101 MHz, DMSO-d_6) δ ppm 144.63, 133.12, 131.66, 127.98, 127.92, 127.26, 117.90, 98.79, 29.45, 22.25, 20.61, 13.92; **HRMS** (ESI$^+$) calcd for $C_{12}H_{15}N_2O_2$ [M+H]$^+$: 219.1128, found *m/z* 219.1131; **MS** (ESI$^+$) m/z 219.1 [M + H]$^+$

S4. HRMS, ^{1}H NMR and ^{13}C NMR Spectra

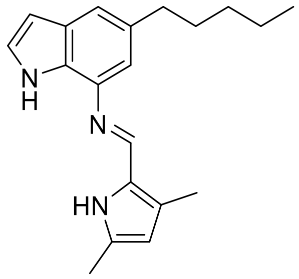

Chemical Formula: $C_{20}H_{25}N_3$
Molecular Weight: 307.44

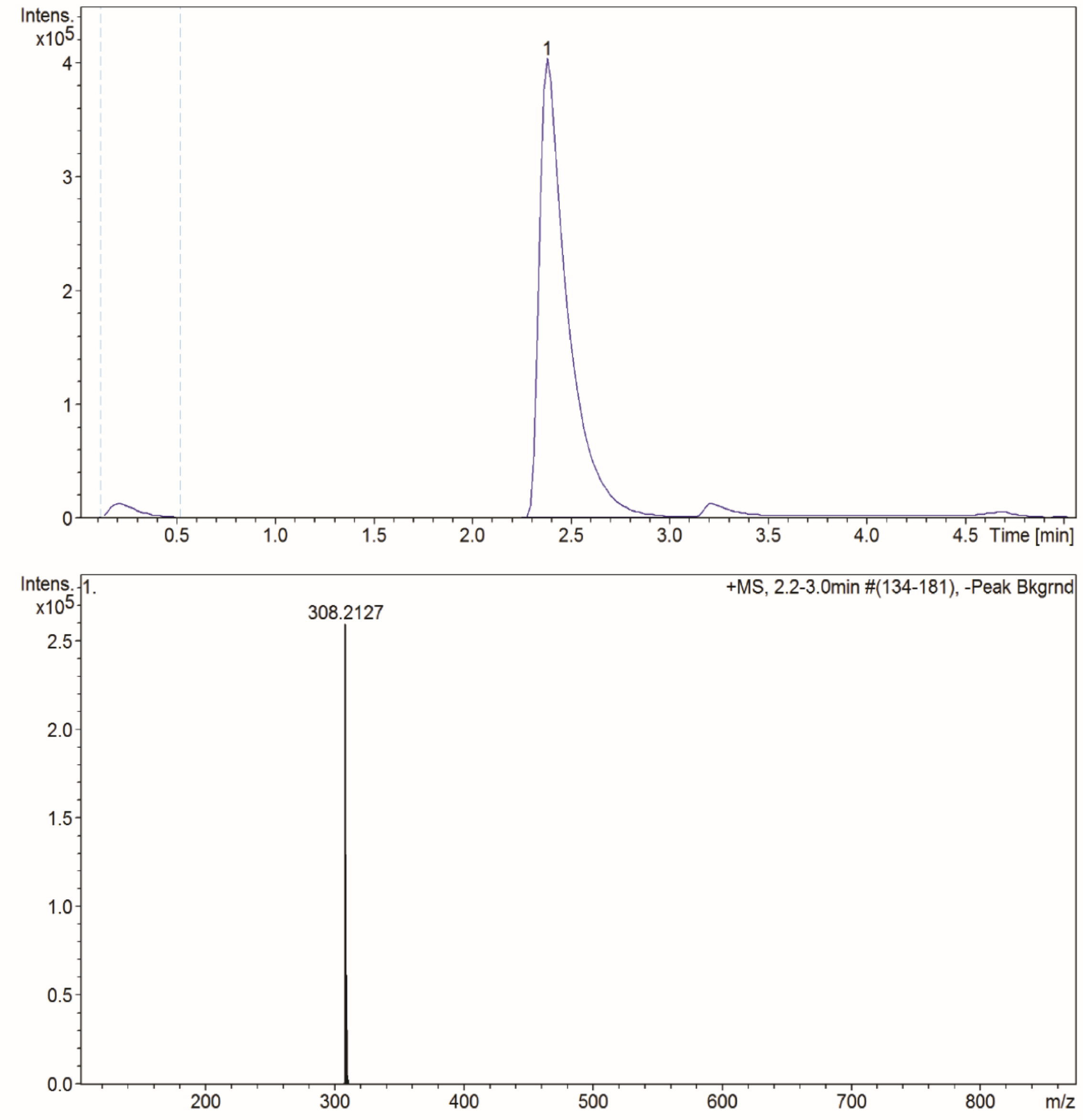

Figure S2. UPLC chromatogram and HRMS (ESI) spectrum of compound **1d**.

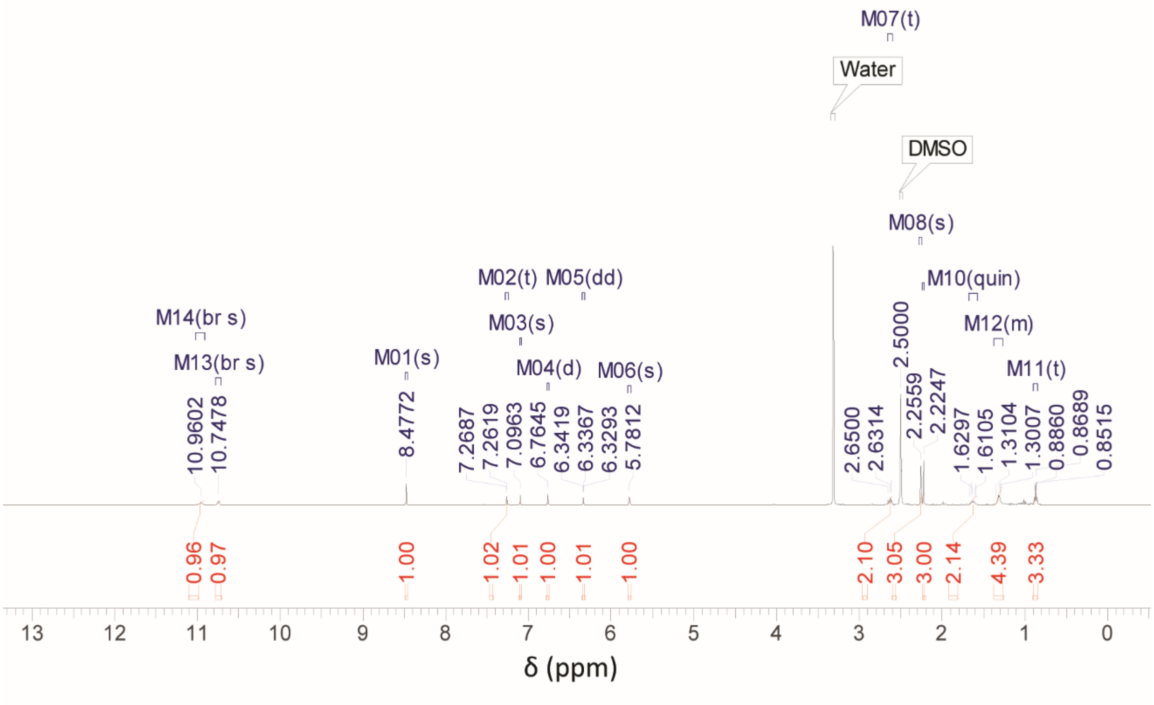

Figure S3. ^{1}H NMR (400 MHz) spectrum of compound **1d** in $(CD_3)_2SO$ at 298 K.

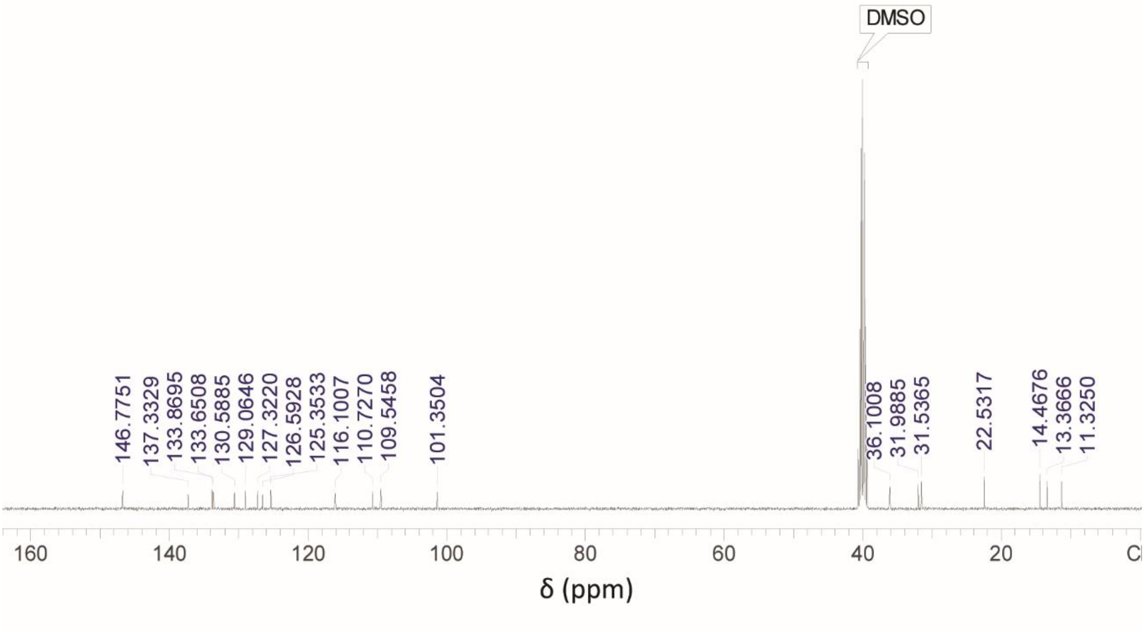

Figure S4. ^{13}C NMR (101 MHz) spectrum of compound **1d** in $(CD_3)_2SO$ at 298 K.

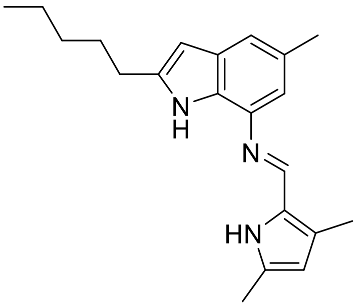

Chemical Formula: $C_{21}H_{27}N_3$
Molecular Weight: 321.47

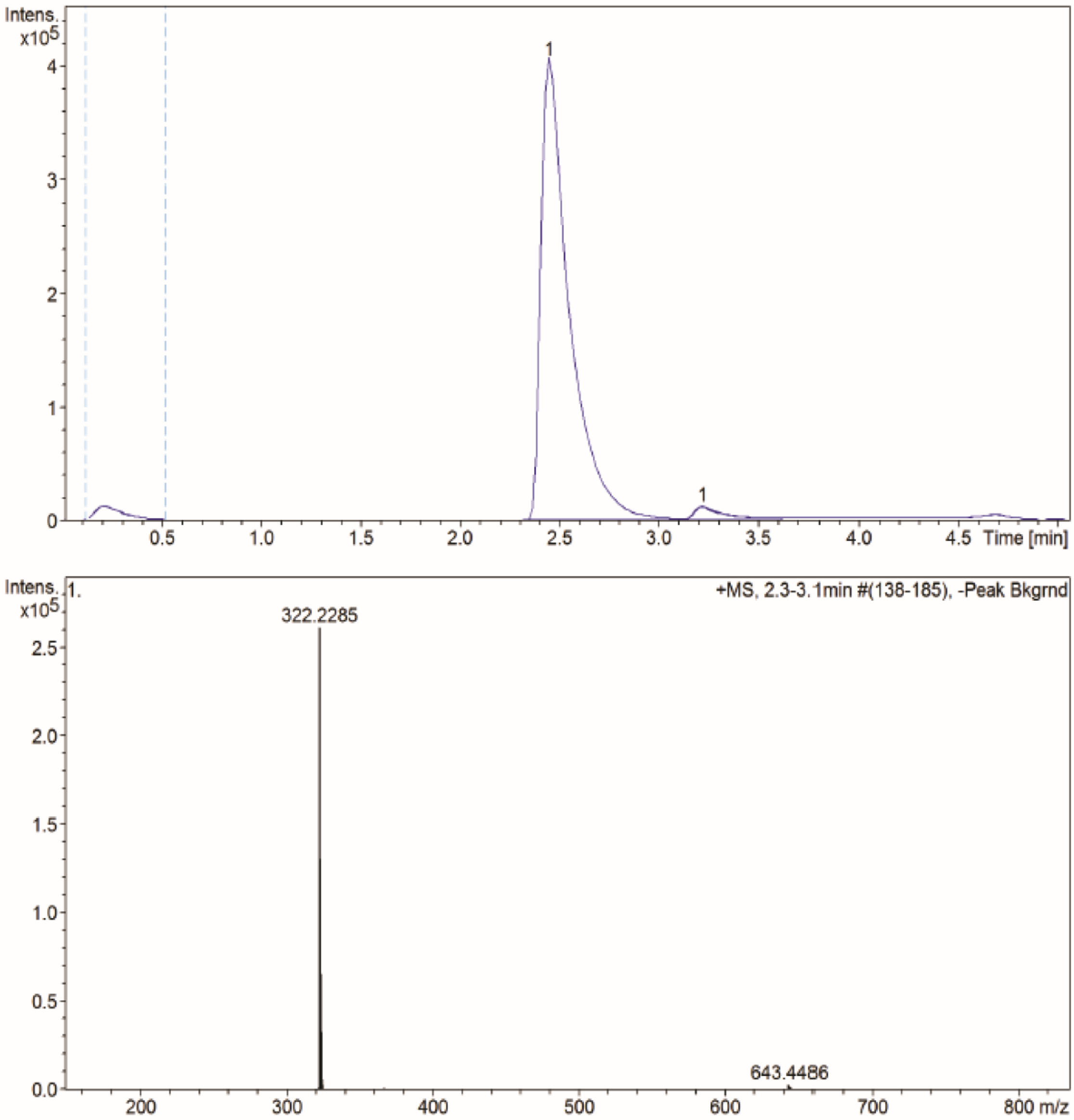

Figure S5. UPLC chromatogram and HRMS (ESI) spectrum of compound **2**.

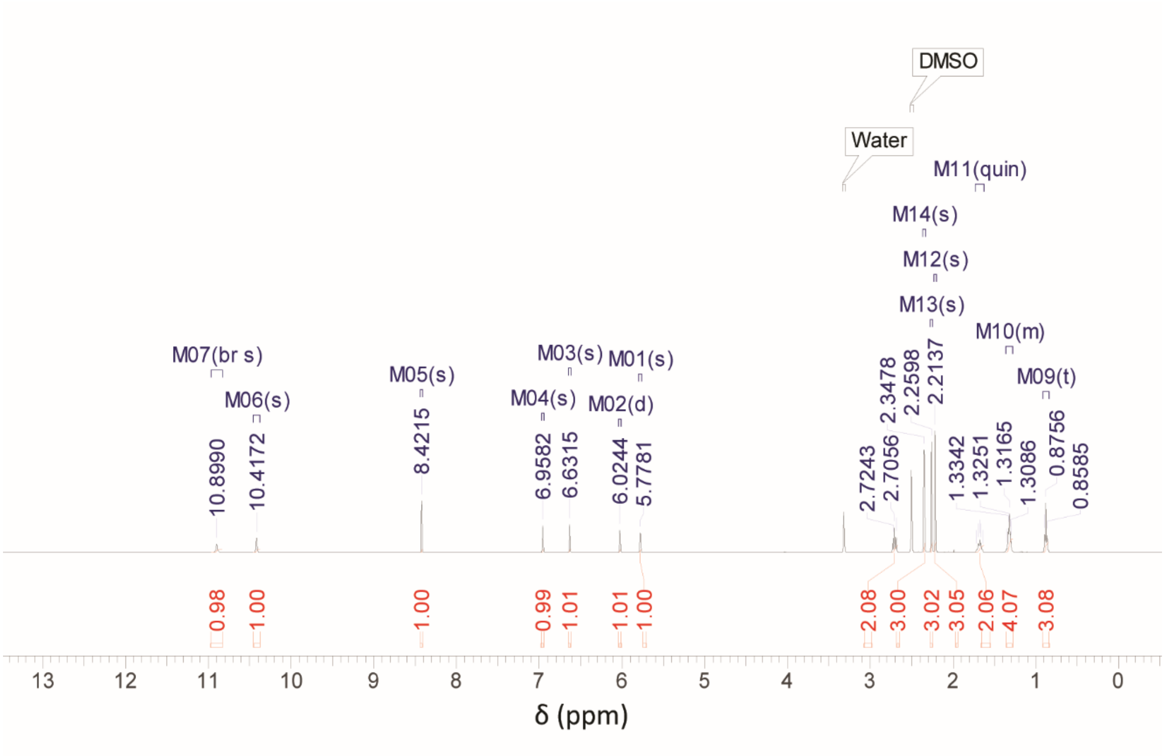

Figure S6. ^{1}H NMR (400 MHz) spectrum of compound **2** in $(CD_3)_2SO$ at 298 K.

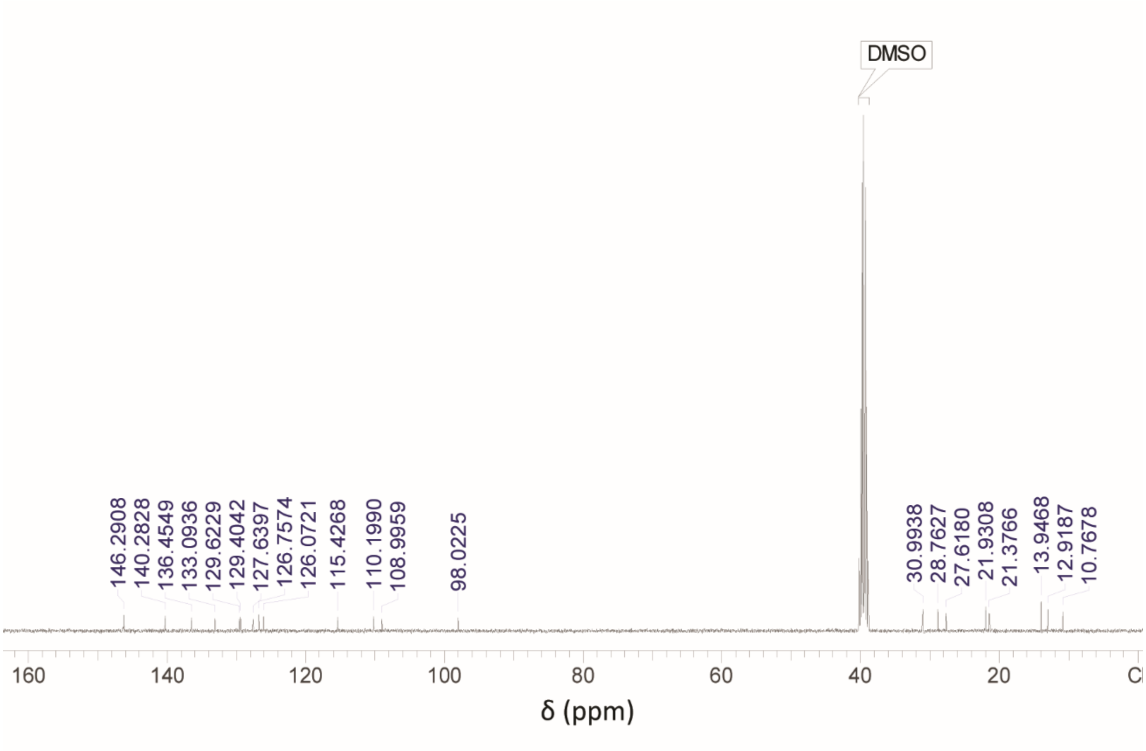

Figure S7. ^{13}C NMR (101 MHz) spectrum of compound **2** in $(CD_3)_2SO$ at 298 K.

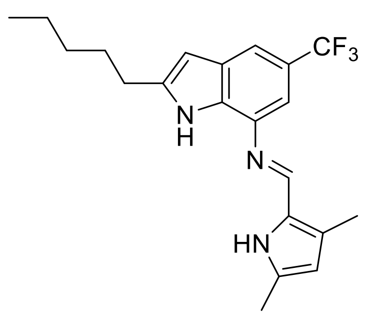

Chemical Formula: $C_{21}H_{24}F_3N_3$
Molecular Weight: 375.44

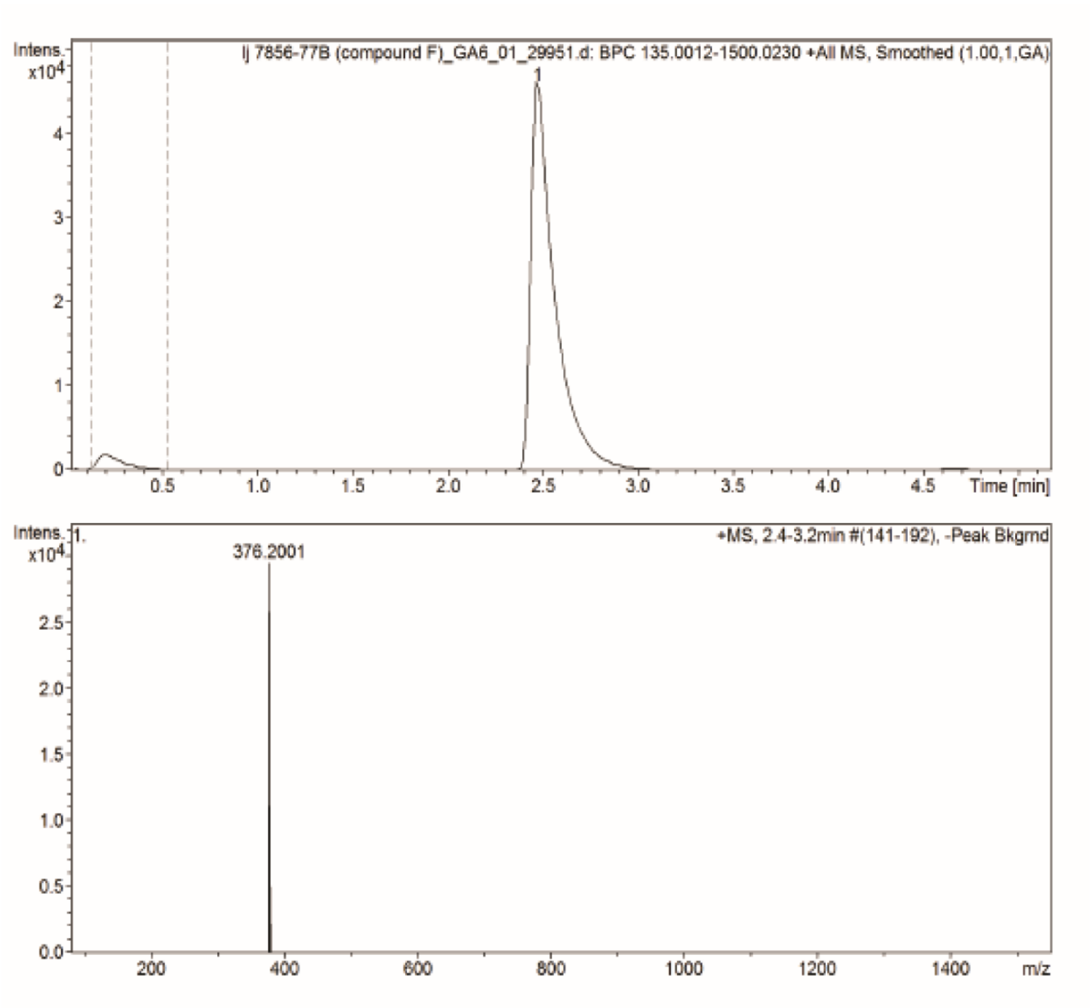

Figure S8. UPLC chromatogram and HRMS (ESI) spectrum of compound **3**.

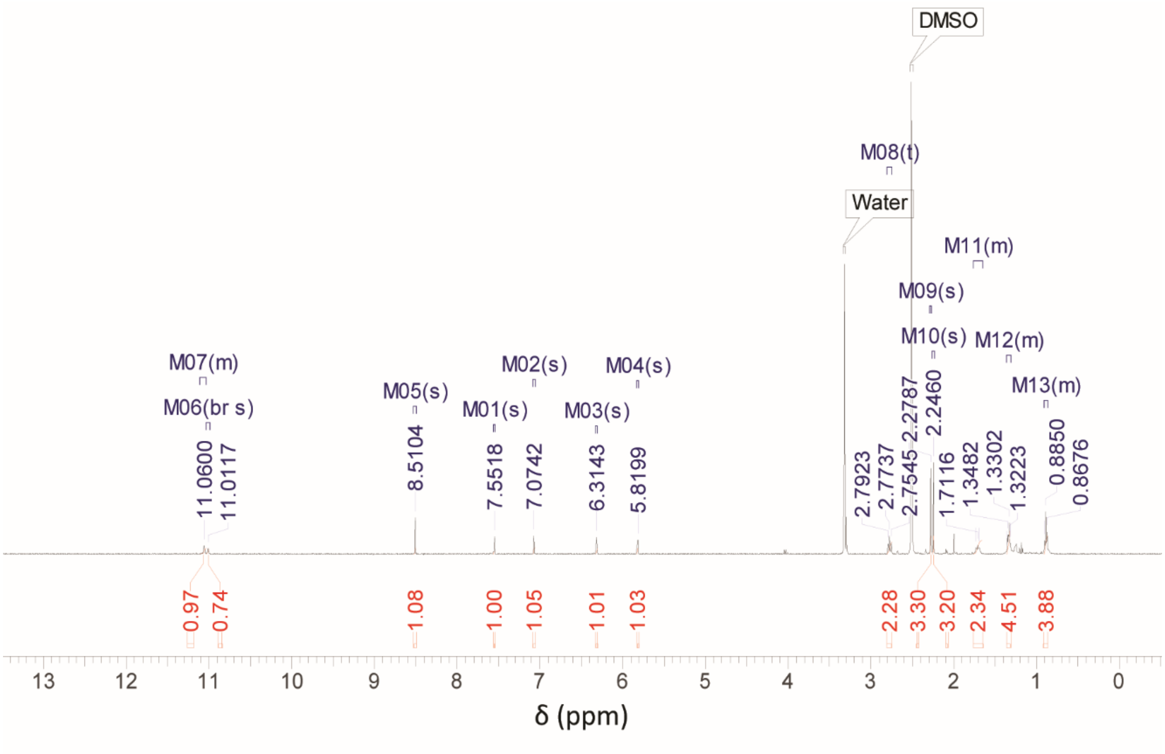

Figure S9. ^{1}H NMR (400 MHz) spectrum of compound **3** in $(CD_3)_2SO$ at 298 K.

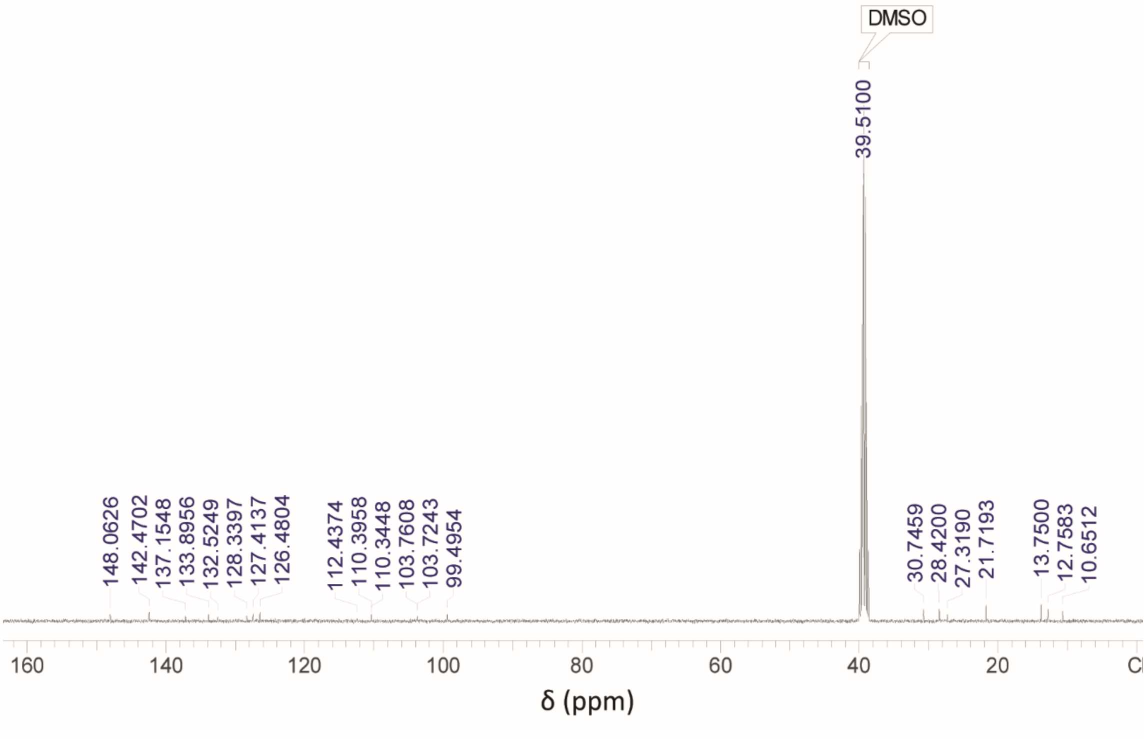

Figure S10. ^{13}C NMR (101 MHz) spectrum of compound **3** in $(CD_3)_2SO$ at 298 K.

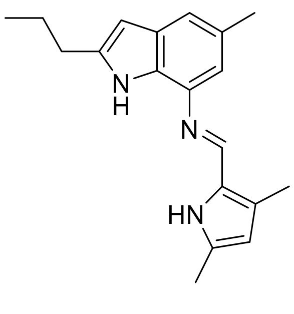

Chemical Formula: $C_{19}H_{23}N_3$
Molecular Weight: 293.41

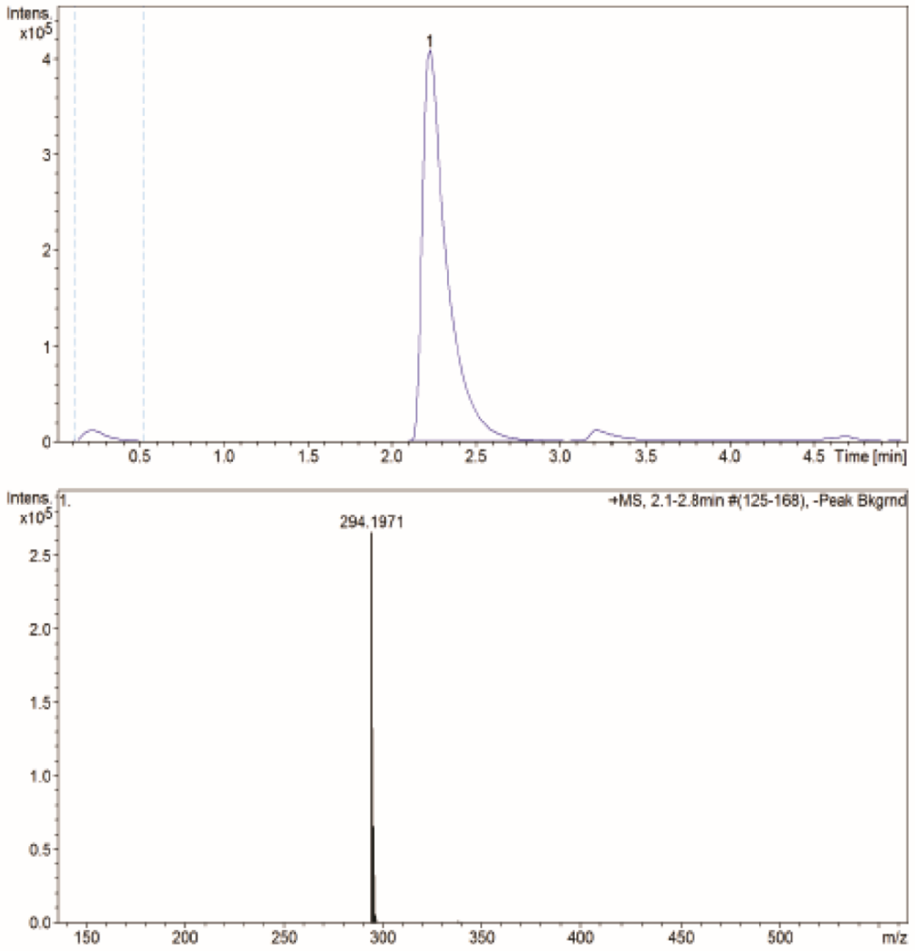

Figure S11. UPLC chromatogram and HRMS (ESI) spectrum of compound **4**.

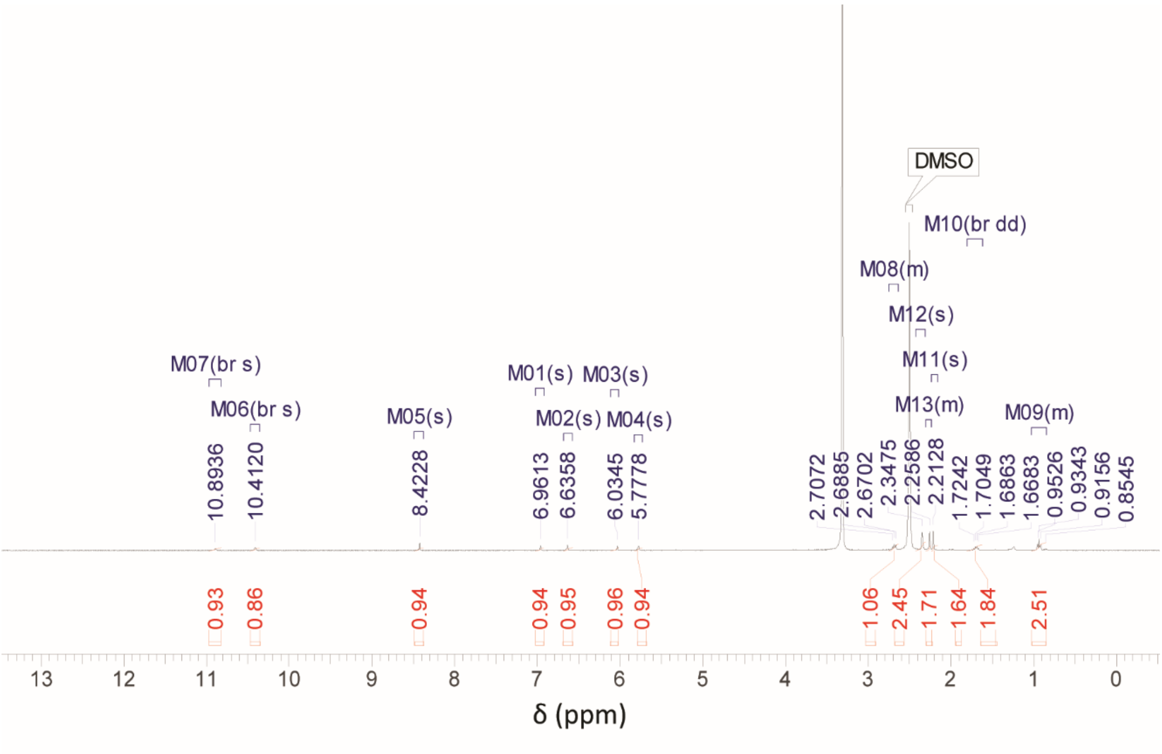

Figure S12. ^{1}H NMR (400 MHz) spectrum of compound **4** in $(CD_3)_2SO$ at 298 K.

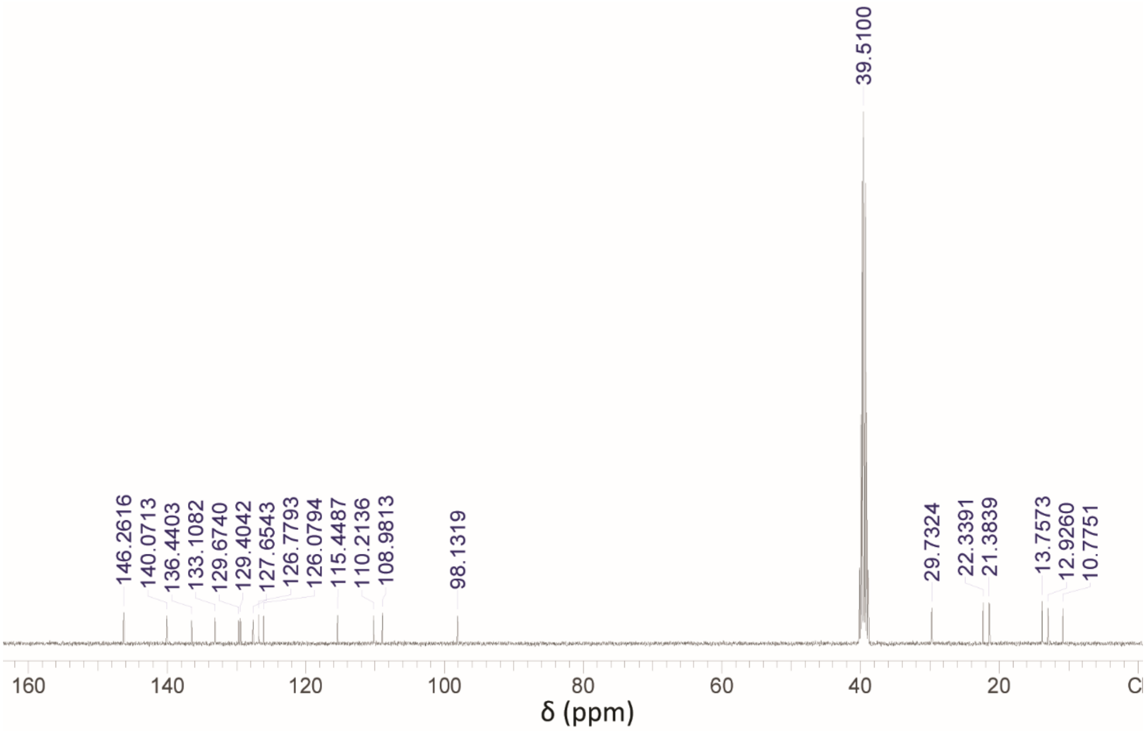

Figure S13. ^{13}C NMR (101 MHz) spectrum of compound **4** in $(CD_3)_2SO$ at 298 K.

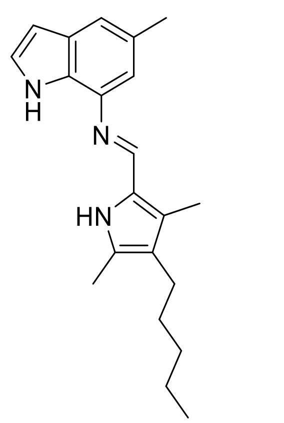

Chemical Formula: $C_{21}H_{27}N_3$
Molecular Weight: 321.47

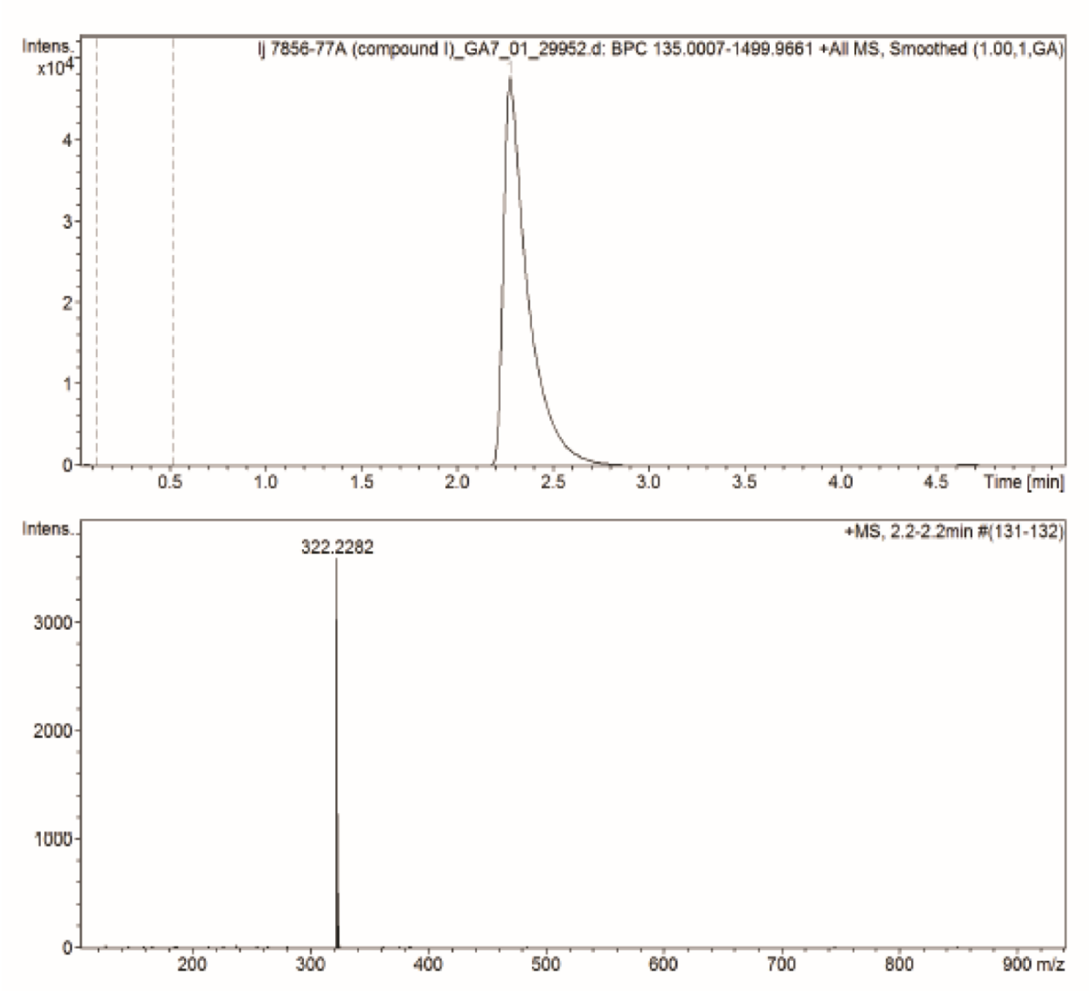

Figure S14. UPLC chromatogram and HRMS (ESI) spectrum of compound **5**.

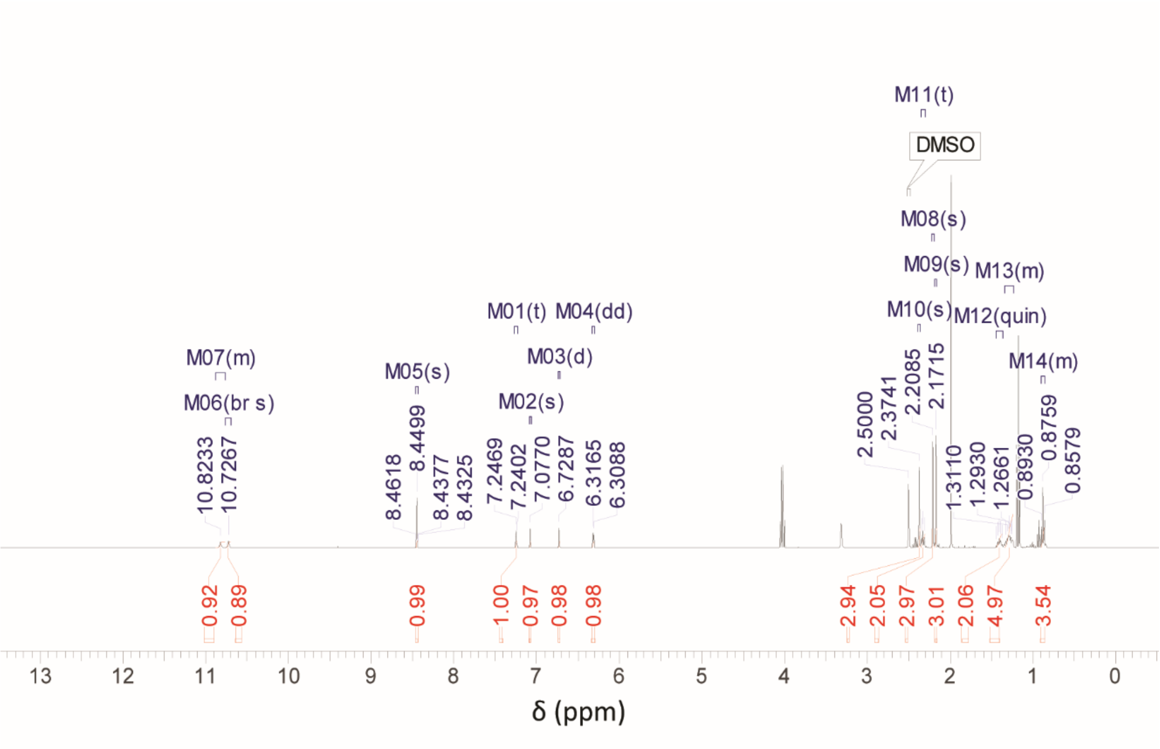

Figure S15. ^{1}H NMR (400 MHz) spectrum of compound **5** in $(CD_3)_2SO$ at 298 K.

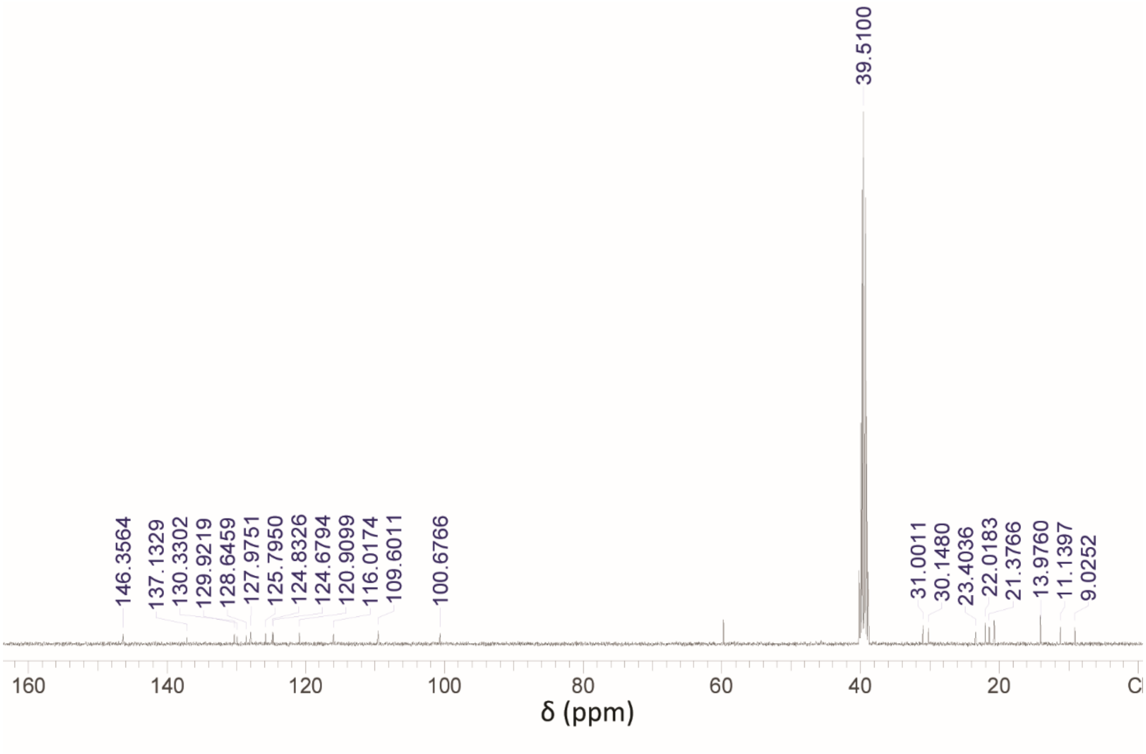

Figure S16. ^{13}C NMR (101 MHz) spectrum of compound **5** in $(CD_3)_2SO$ at 298 K.

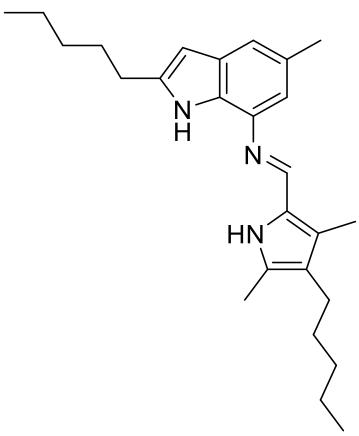

Chemical Formula: $C_{26}H_{37}N_3$
Molecular Weight: 391.60

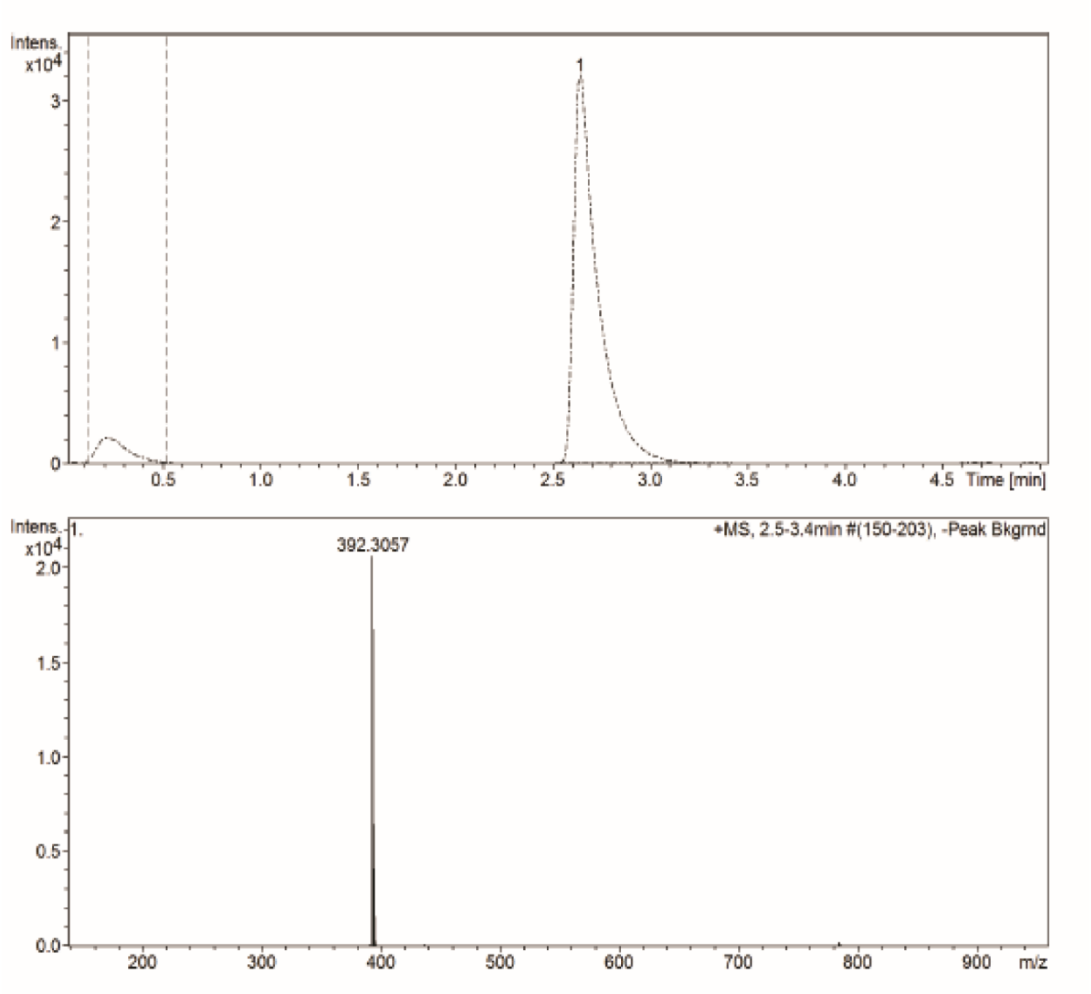

Figure S17. UPLC chromatogram and HRMS (ESI) spectrum of compound **6**.

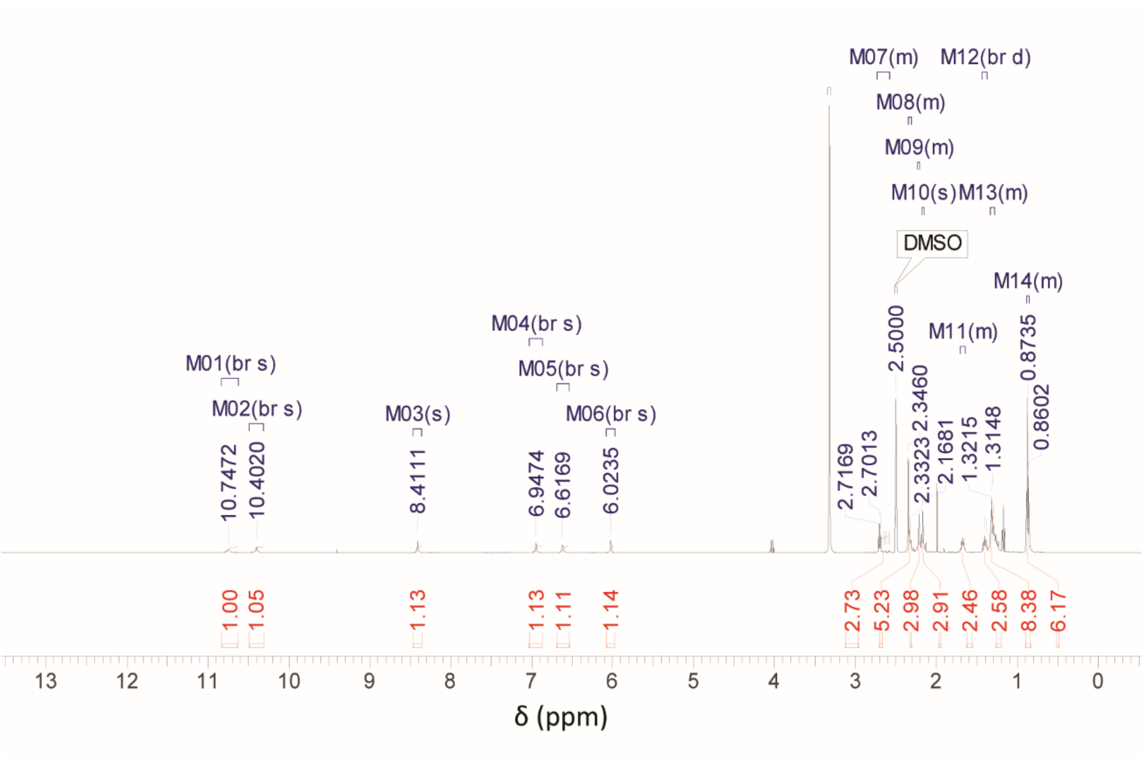

Figure S18. ^{1}H NMR (400 MHz) spectrum of compound **6** in $(CD_3)_2SO$ at 298 K.

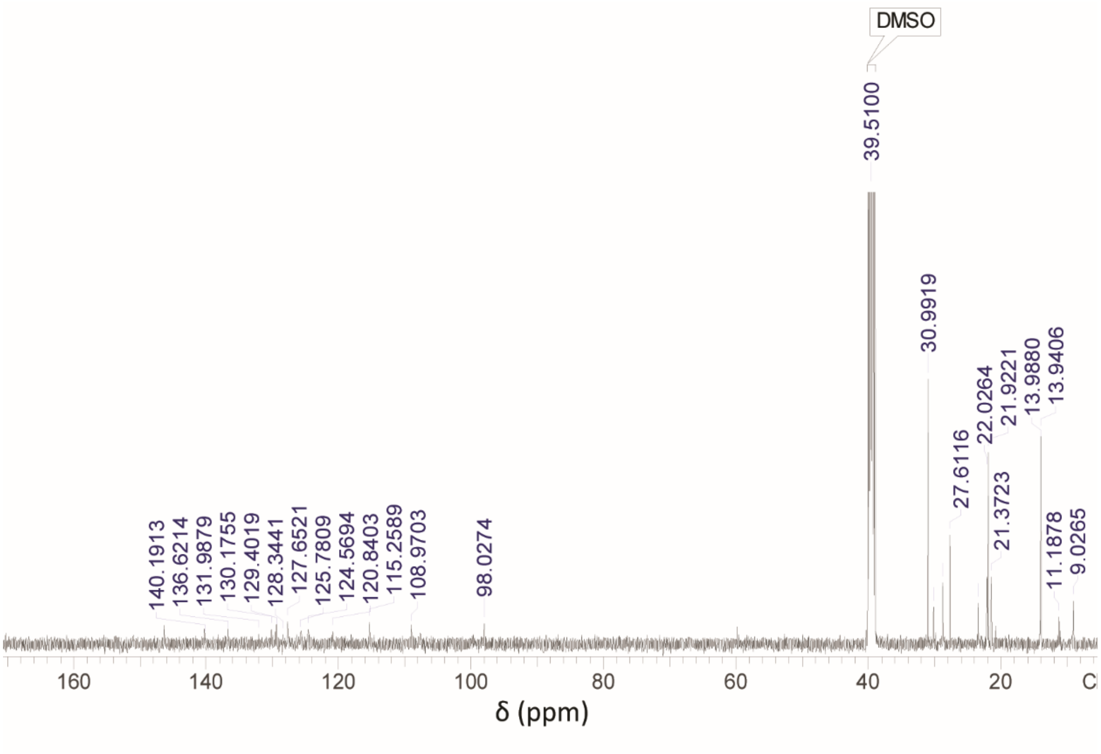

Figure S19. ^{13}C NMR (101 MHz) spectrum of compound **6** in $(CD_3)_2SO$ at 298 K.

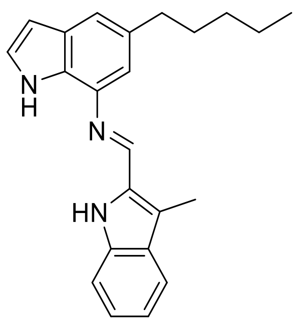

Chemical Formula: $C_{23}H_{25}N_3$
Molecular Weight: 343.47

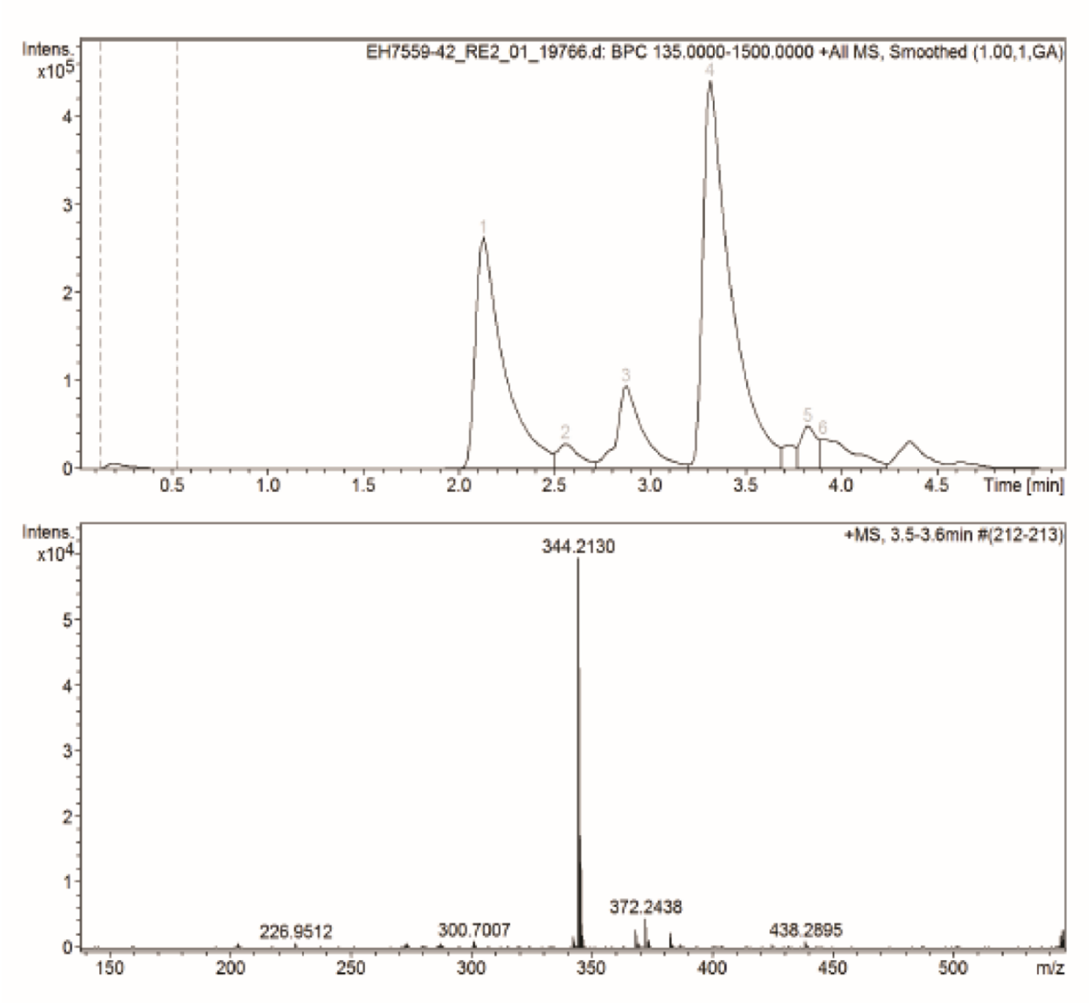

Figure S20. UPLC chromatogram and HRMS (ESI) spectrum of compound **7**.

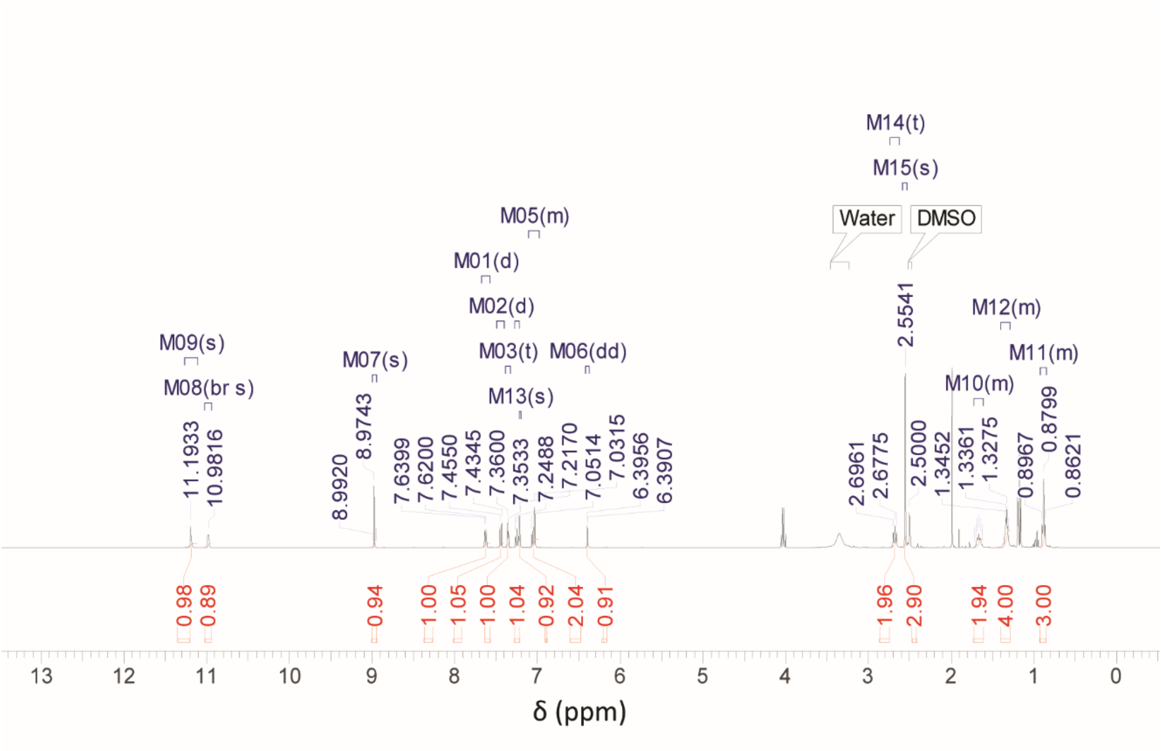

Figure S21. ^{1}H NMR (400 MHz) spectrum of compound **7** in $(CD_3)_2SO$ at 298 K.

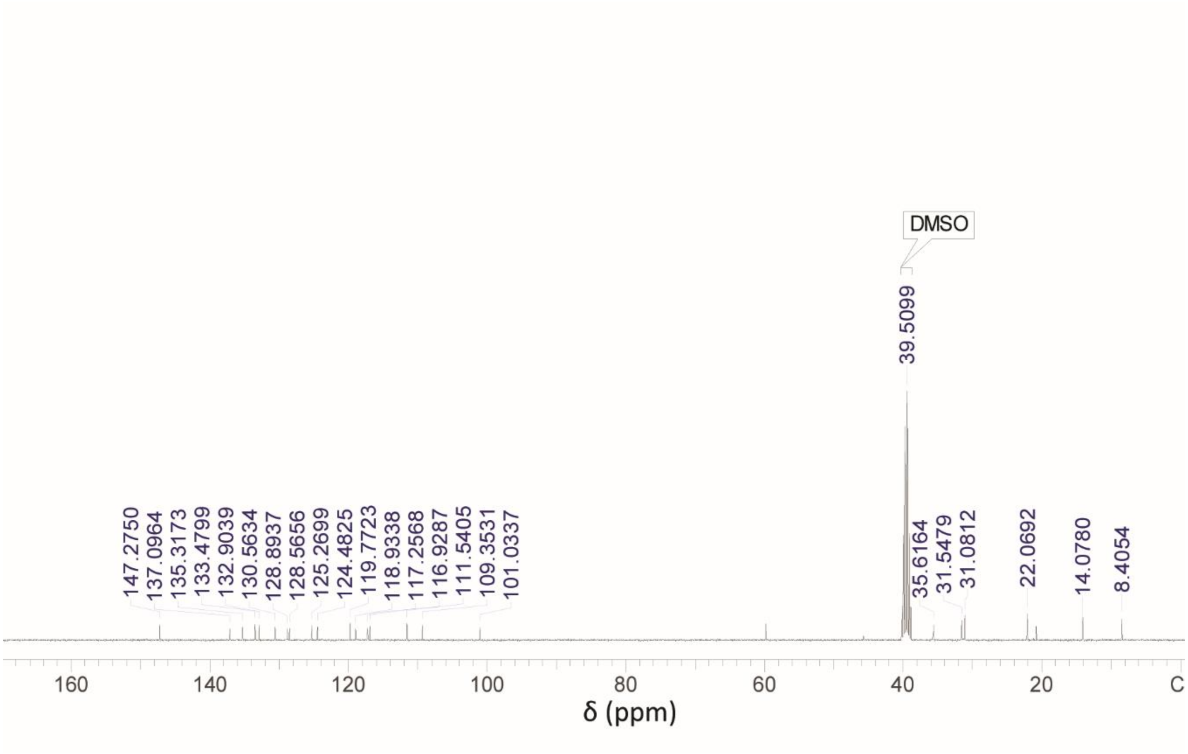

Figure S22. ^{13}C NMR (101 MHz) spectrum of compound **7** in $(CD_3)_2SO$ at 298 K.

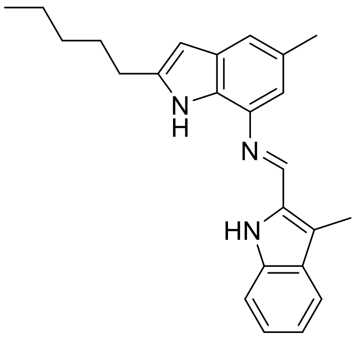

Chemical Formula: $C_{24}H_{27}N_3$
Molecular Weight: 357.50

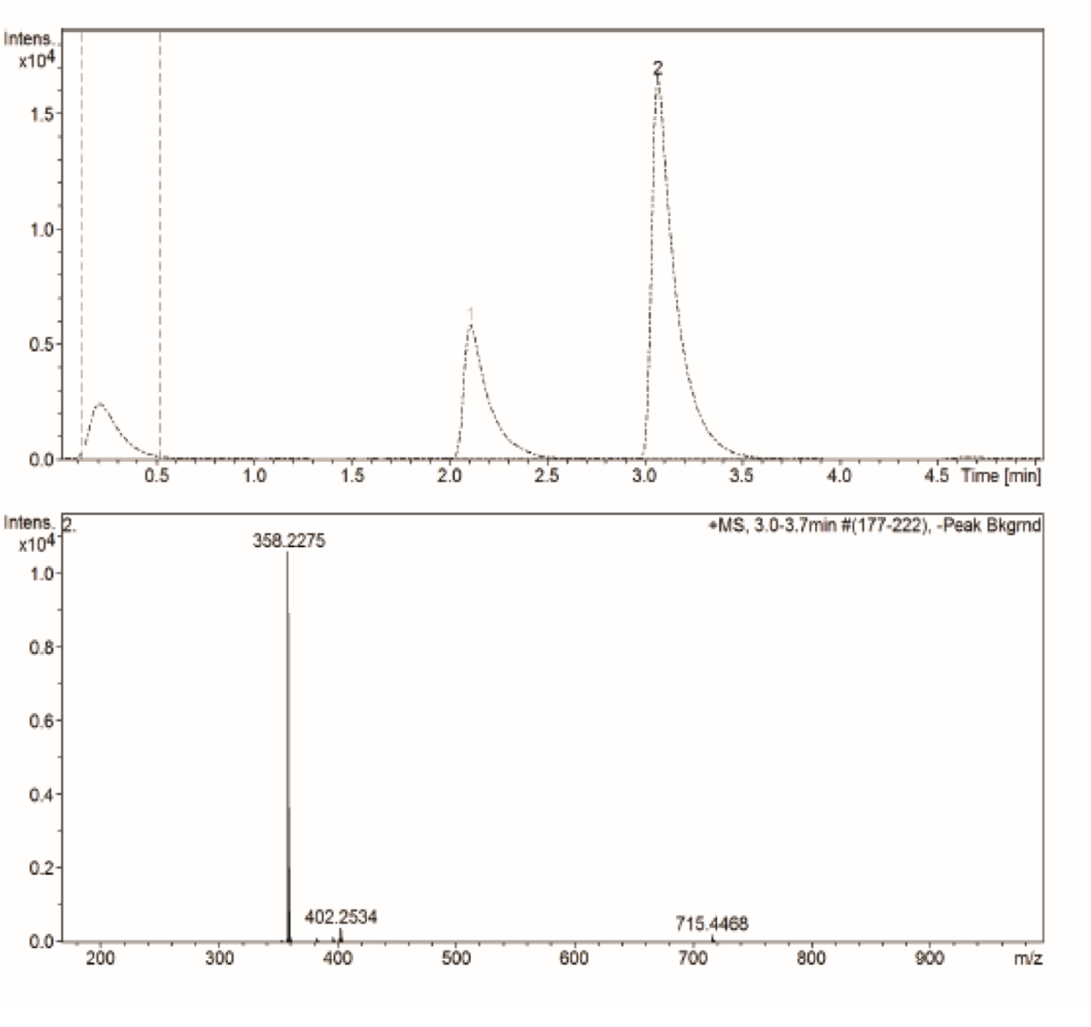

Figure S23. UPLC chromatogram and HRMS (ESI) spectrum of compound **8**.

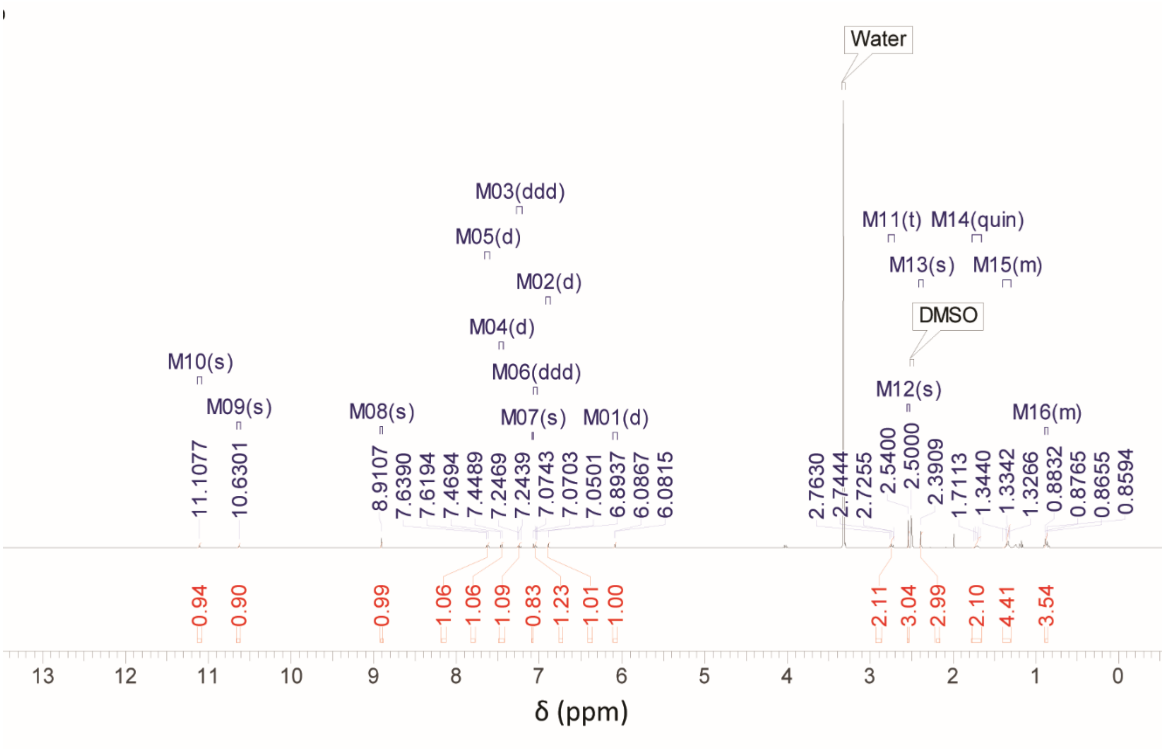

Figure S24. ^{1}H NMR (400 MHz) spectrum of compound **8** in $(CD_3)_2SO$ at 298 K.

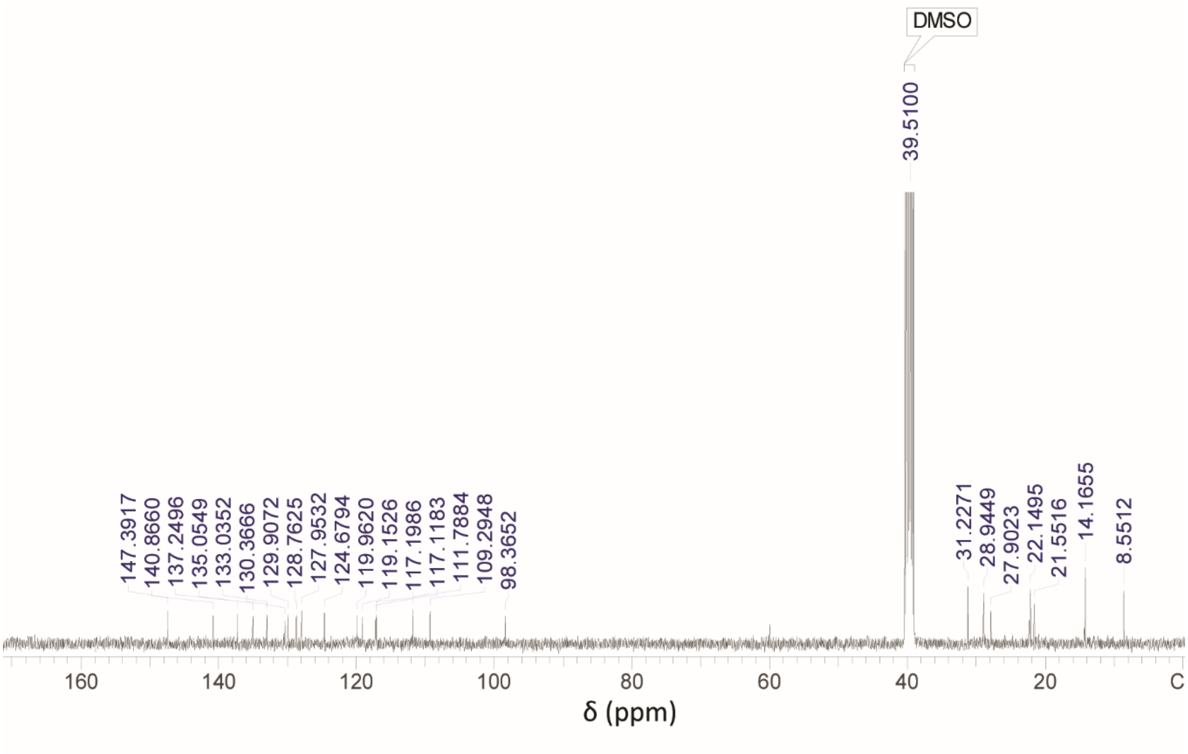

Figure S25. ^{13}C NMR (101 MHz) spectrum of compound **8** in $(CD_3)_2SO$ at 298 K.

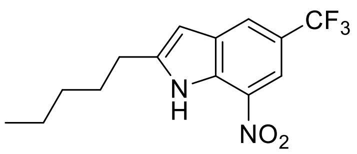

Chemical Formula: $C_{14}H_{15}F_3N_2O_2$
Molecular Weight: 300.28

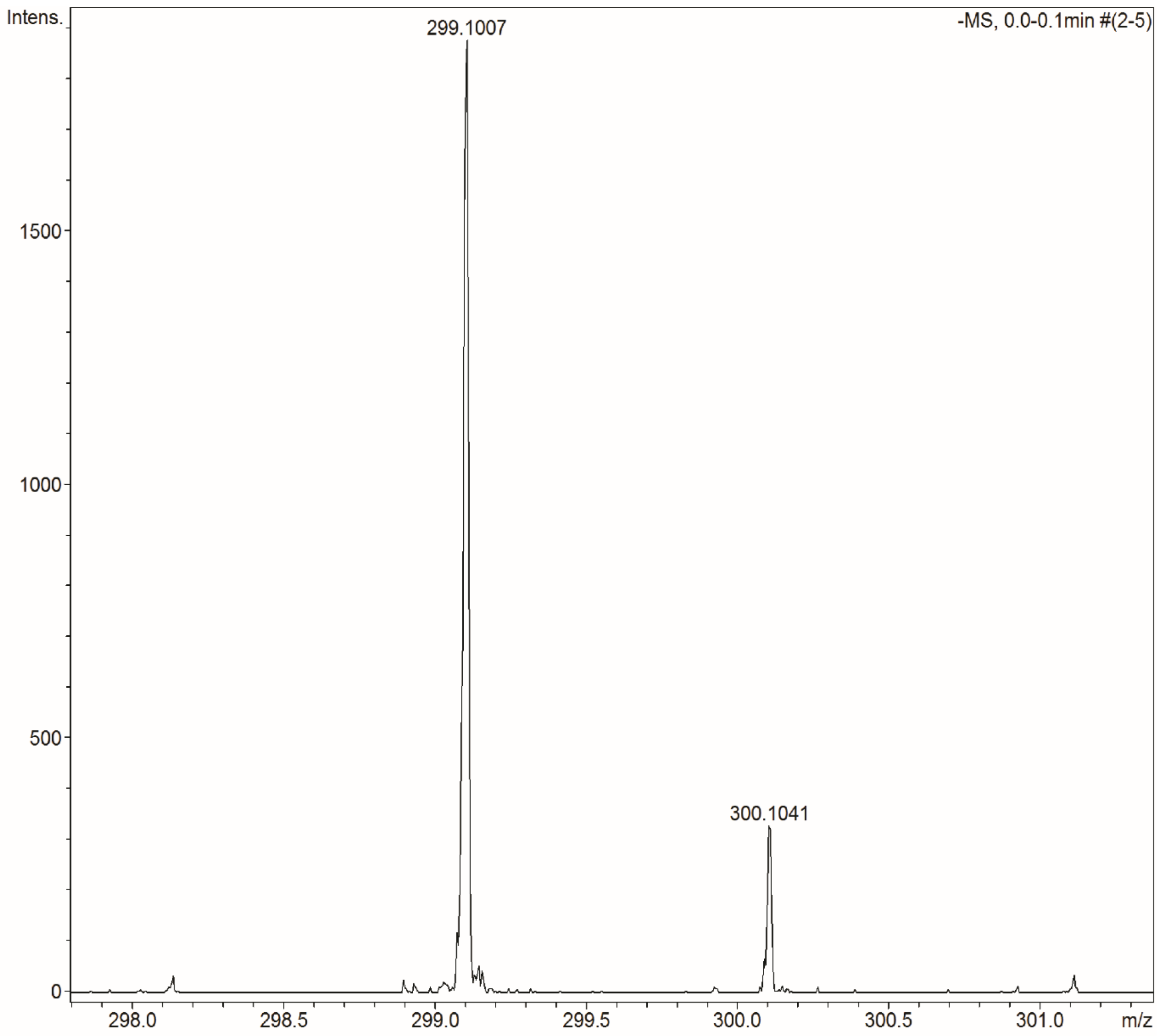

Figure S26. UPLC chromatogram and HRMS (ESI) spectrum of compound **19**.

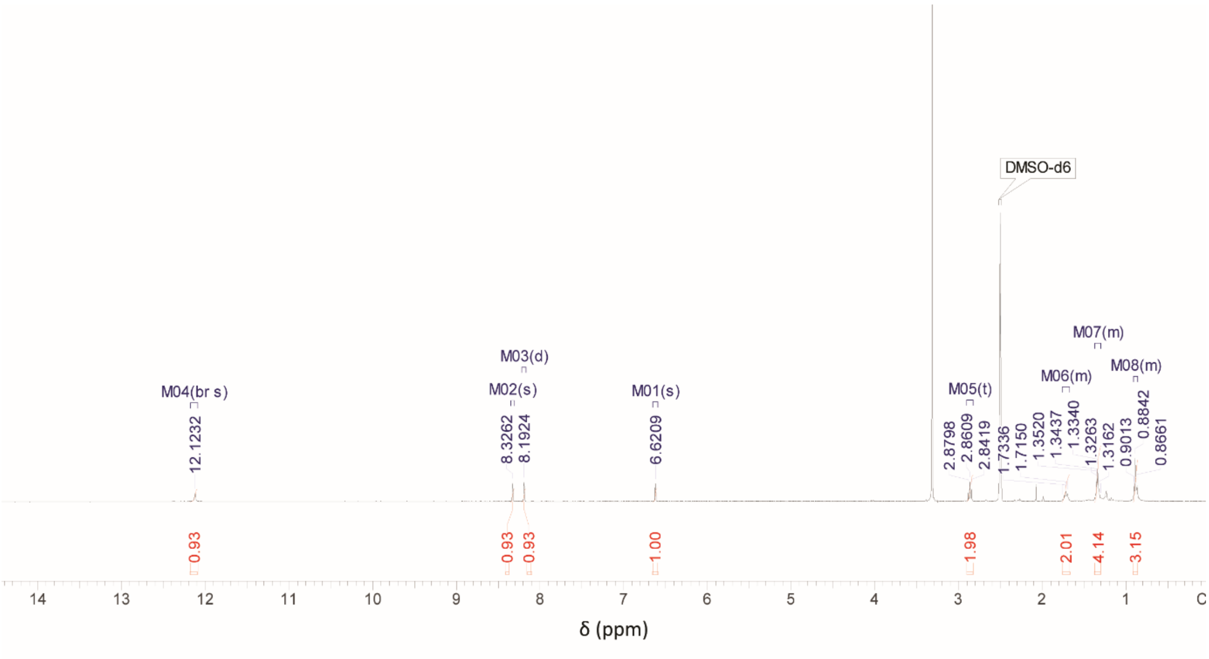

Figure S27. ^{1}H NMR (400 MHz) spectrum of compound **19** in $(CD_3)_2SO$ at 298 K.

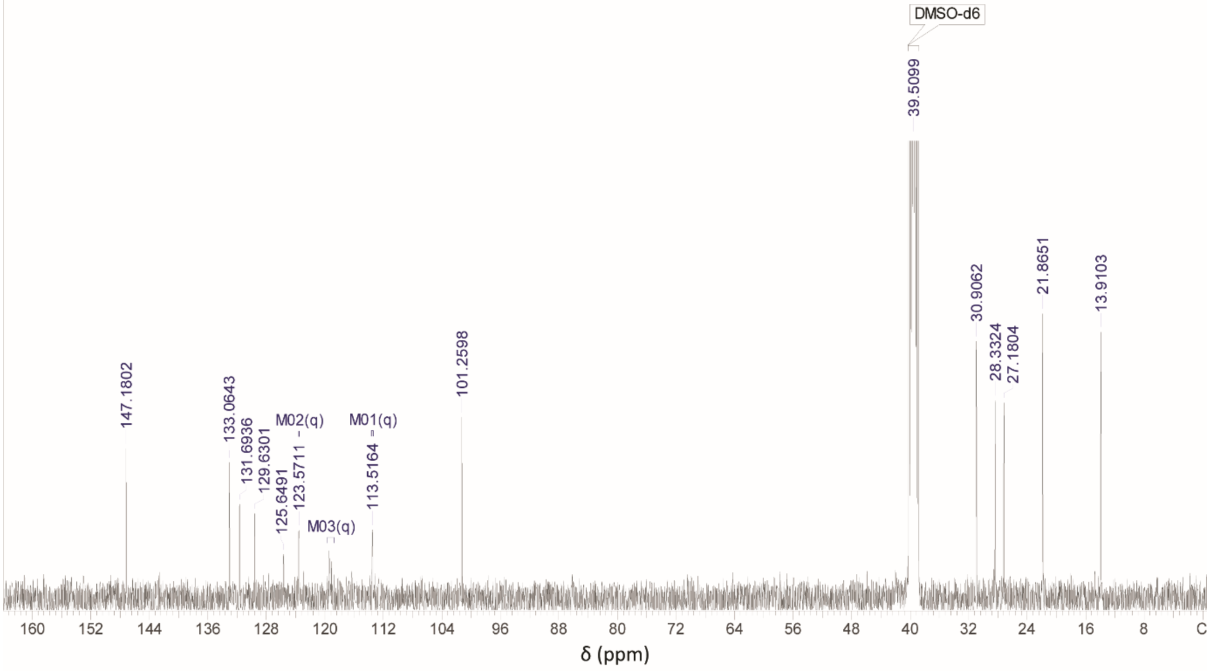

Figure S28. ^{13}C NMR (101 MHz) spectrum of compound **19** in $(CD_3)_2SO$ at 298 K.

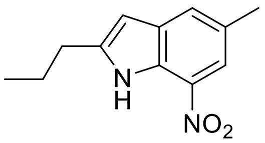

Chemical Formula: $C_{12}H_{14}N_2O_2$
Molecular Weight: 218.26

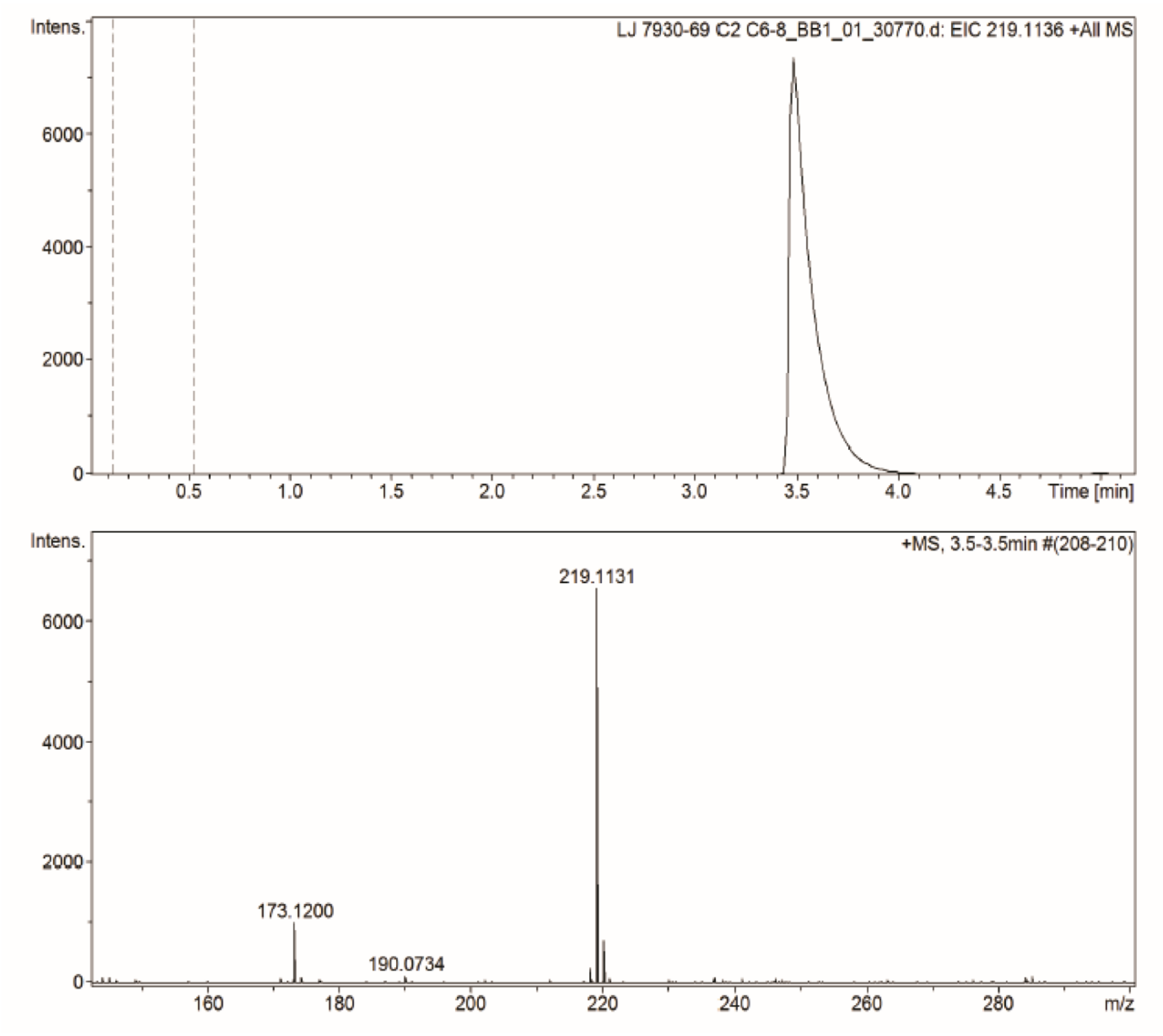

Figure S29. UPLC chromatogram and HRMS (ESI) spectrum of compound **20**.

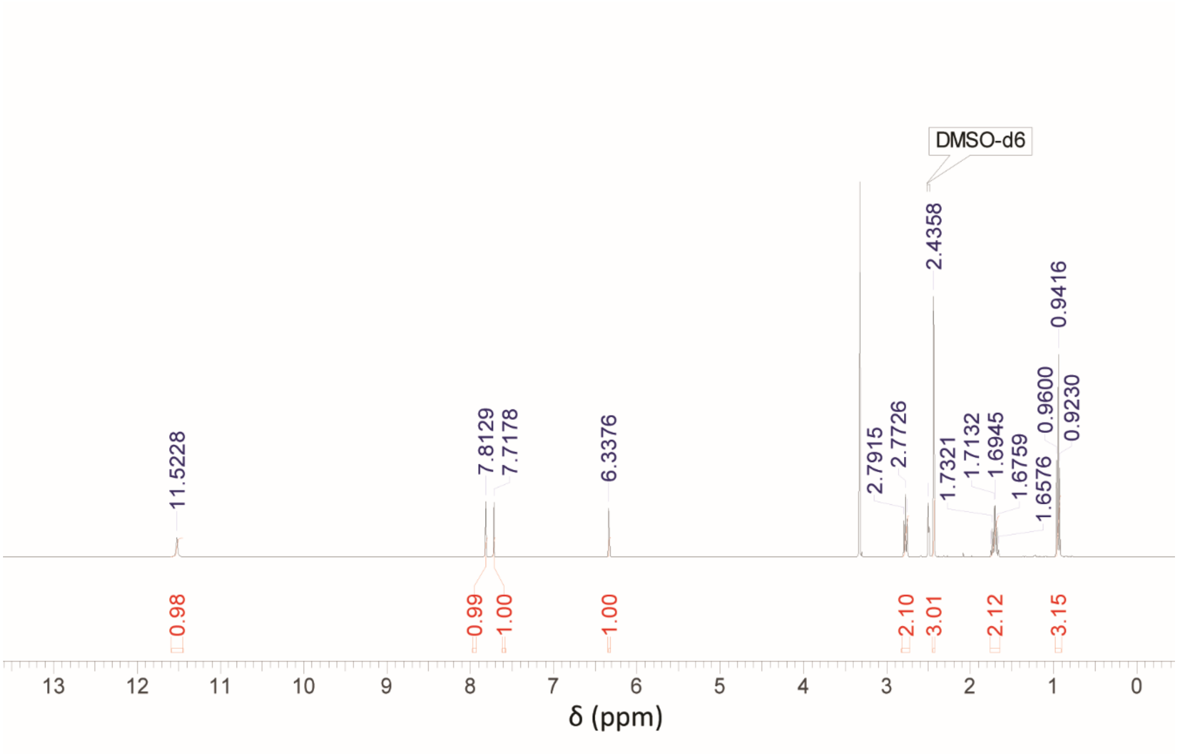

Figure S30. ^{1}H NMR (400 MHz) spectrum of compound **20** in $(CD_3)_2SO$ at 298 K.

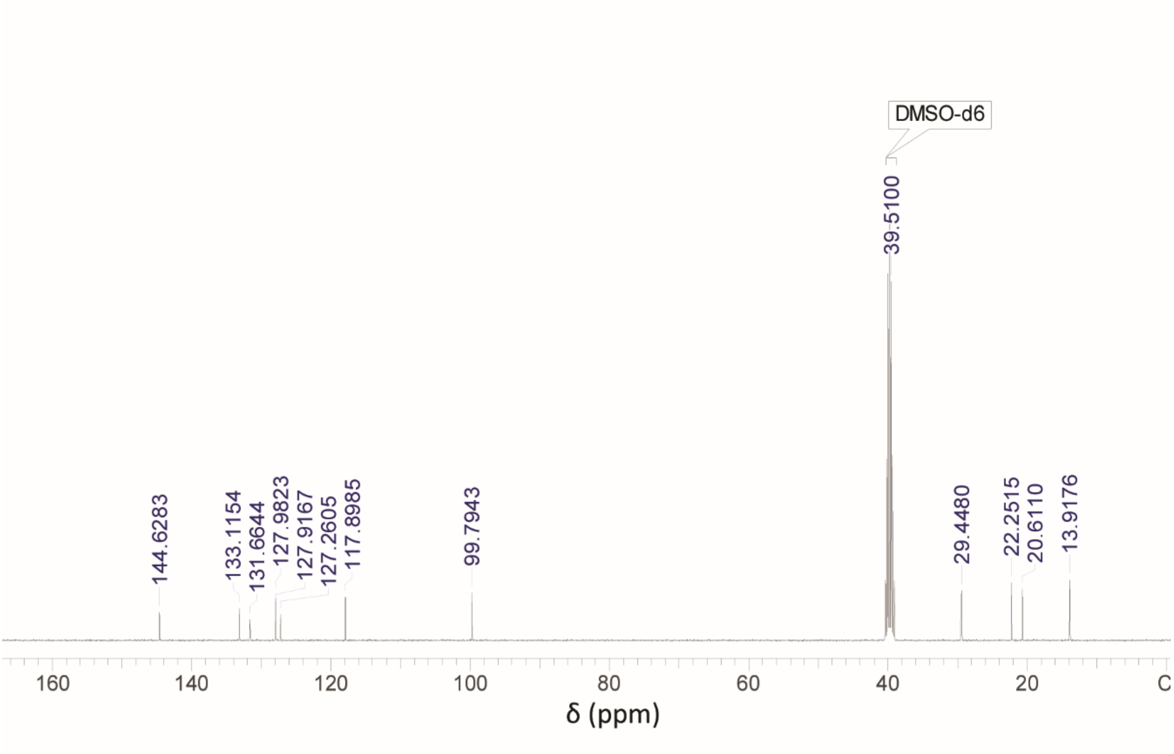

Figure S31. ^{13}C NMR (101 MHz) spectrum of compound **20** in $(CD_3)_2SO$ at 298 K.

S5. X-ray Crystallography

Slow evaporation using a layered solvent system of methanol and hexane gave yellow crystals of compounds **1d**, **2** and **4** which were suitable for single-crystal X-ray diffraction. The single crystals were mounted on a MiTeGen MicroMesh support, and data were collected on various diffractometers (details below) at the X-ray Crystallographic facilities at the University of Southampton. Graphics were generated using Mercury 3.5 (CSDS, 2015), PyMOL Molecular Graphics System (Schrödinger, 2010) and Pov-Ray 3.7 (2013). All crystallographic data have been deposited at the Cambridge Crystallographic Database Centre (CCDC).

The X-ray diffraction data for structures **1d** and **2** were collected on a Rigaku AFC12 goniometer equipped with an enhanced sensitivity (HG) Saturn724+ detector mounted at the window of an FR-E+ SuperBright molybdenum (MoKα, λ=0.71073 Å) rotating anode generator with VHF Varimax optics (70µm focus), using the CrystalClear-SM Expert 3.1 b27 (Rigaku, 2013) software.

The X-ray diffraction data for structure **4** was collected on a Rigaku AFC12 goniometer equipped with an enhanced sensitivity (HG) Saturn724+ detector mounted at the window of an FR-E+ SuperBright molybdenum (MoKα, λ=0.71075 Å) rotating anode generator with HF Varimax optics (100 µm focus), using the CrystalClear-SM Expert 3.1 b27 (Rigaku, 2013) software.

All data set were collected at 100 K using an Oxford Cryostream low temperature device. Data reduction and cell refinement was carried out using CrysAlisPro (Version 1.171.37.31, Agilent Technologies). All structures were solved by direct methods as implemented in SHELXS and refined by full-matrix least-squares refinements using SHELXS to the final *R* value, carried out using the OLEX27 (Version 1.2.7) software. In all cases, non-hydrogen atoms were refined with anisotropic displacement parameters. Most hydrogen atoms were added at calculated positions and refined using a riding model with isotropic displacement parameters based on the equivalent isotropic displacement parameters (U_{eq}) of the parent atom. The hydrogen atoms on the nitrogen atoms were assigned manually from the observed electron density peaks and refined with isotropic displacement parameters.

Crystal data for structure **1d**: $C_{20}H_{25}N_3$, crystal size = 0.25 × 0.1 × 0.02 mm^3, clear yellow needle, triclinic, space group: P_{-1}, a = 14.6610(4) Å, b = 16.2157(5) Å, c = 16.4213(5) Å, α = 92.454 ˚, β = 103.399(3) ˚, γ = 112.518 ˚, V = 3470.4(2) Å^3, Z = 8, ρ_c = 1.177 g cm^{-3}, μ = 0.070 mm^{-1}, T = 293(2) K, ϑ_{max} = 24.999, reflections collected: 45685, independent reflections: 12216 (R_{int} = 0.0470), *GooF* = 1.014, 873 parameters. *R* indices (all data): R_1 = 0.0734, wR_2 = 0.1099, final *R* indices [$I > 2\sigma I$]: R_1 = 0.0452, wR_2 = 0.0992, largest diff. peak and hole = 0.227 and –0.219 e Å^{-3}. CCDC 1546469

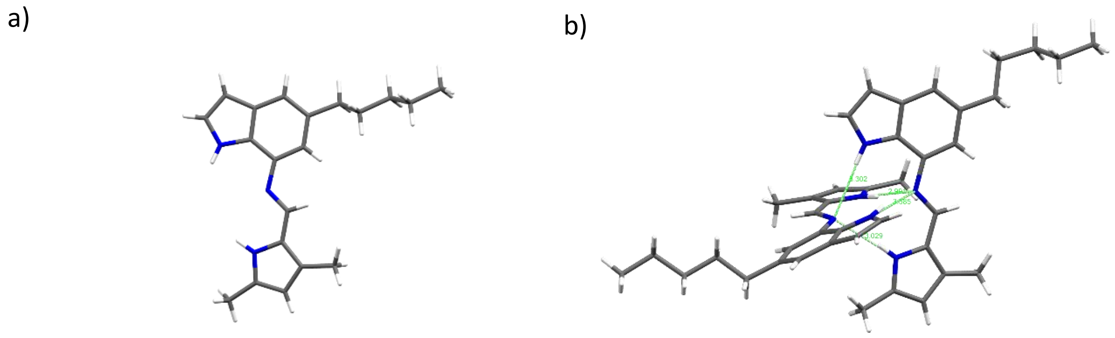

Figure S32. a) Single-crystal X-ray structure of **1d**. b) Single-crystal X-ray structure of **1d** showing the intermolecular H-bonds and lengths in green.

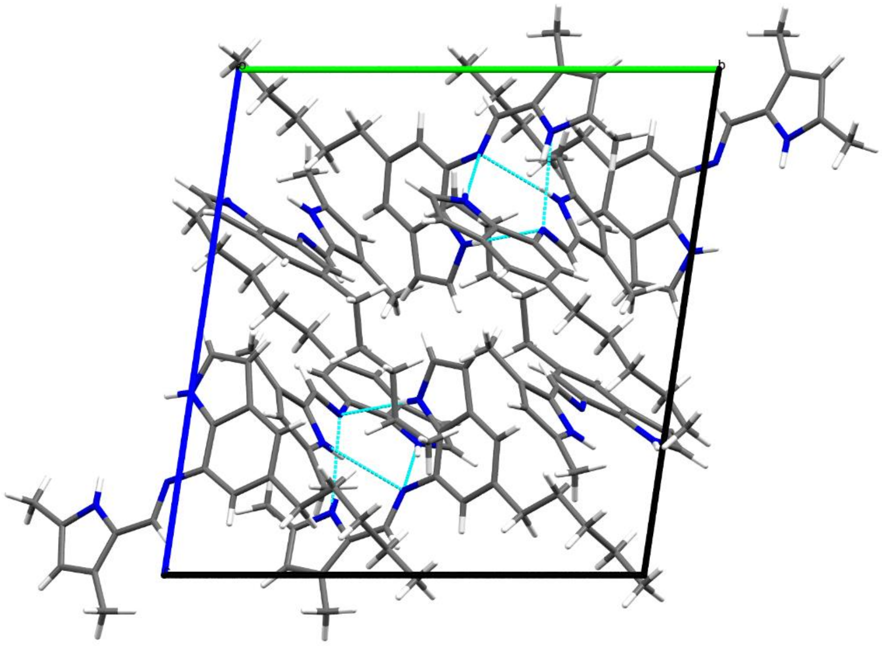

Figure S33. Crystallographic packed diagram of **1d** with the unit cell viewed down the *a* axis. Intermolecular bonds are shown in pale blue.

Crystal data for structure **2**: $C_{21}H_{27}N_3$, crystal size = 0.20 × 0.20 × 0.04 mm^3, clear yellow needle, triclinic, space group: P_{-1}, a = 8.7884(3) Å, b = 12.7773(5) Å, c = 17.3853(7) Å, α = 106.886(3) ˚, β = 92.055(3) ˚, γ = 95.826(3) ˚, V = 1854.01(13) Å^3, Z = 4, ρ_c = 1.152 g cm^{-3}, μ = 0.068 mm^{-1}, T = 100 K, ϑ_{max} = 28.700, reflections collected: 38670, independent reflections: 9548 (R_{int} = 0.0577), *GooF* = 1.018, 441 parameters. *R* indices (all data): R_1 = 0.1070, wR_2 = 0.1480, final *R* indices [$I > 2\sigma I$]: R_1 = 0.0590, wR_2 = 0.1287, largest diff. peak and hole = 0.43 and –0.25 e Å^{-3}. CCDC 1546470

a)

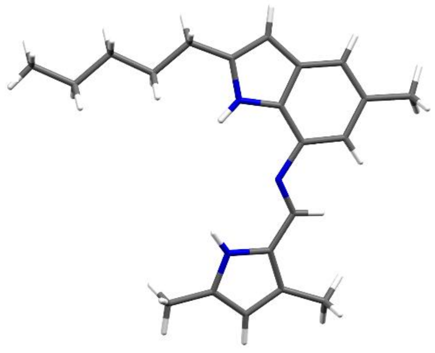

b)

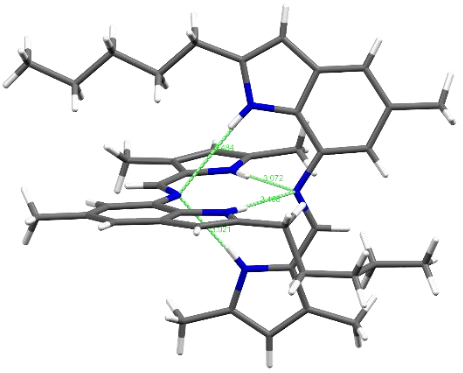

Figure S34. a) Single-crystal X-ray structure of **2**. b) Single-crystal X-ray structure of **2** showing the intermolecular H-bonds and lengths in green.

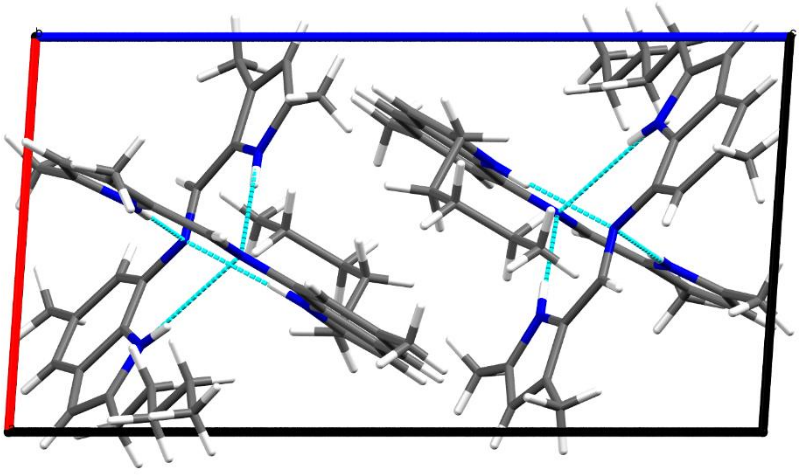

Figure S35. Crystallographic packed diagram of **2** with the unit cell viewed down the *b* axis. Intermolecular bonds are shown in pale blue.

Crystal data for structure **4**: $C_{19}H_{23}N_3$, crystal size = 0.13 × 0.07 × 0.02 mm^3, clear yellow needle, triclinic, space group: P_{-1}, a = 9.9991(4) Å, b = 11.5300(6) Å, c = 15.9917(7) Å, α = 108.455(4) ˚, β = 98.750(4) ˚, γ = 92.295(4) ˚, V = 1720.84(14) $Å^3$, Z = 4, ρ_c = 1.132 g cm^{-3}, μ = 0.068 mm^{-1}, T = 293(2) K, ϑ_{max} = 65.372, reflections collected: 24855, independent reflections: 7033 (R_{int} = 0.0425), *GooF* = 1.012, 421 parameters. R indices (all data): R_1 = 0.0898, wR_2 = 0.1319, final R indices [$I > 2\sigma I$]: R_1 = 0.0550, wR_2 = 0.1170, largest diff. peak and hole = 0.23 and –0.20 e $Å^{-3}$. CCDC 1546471

a) b)

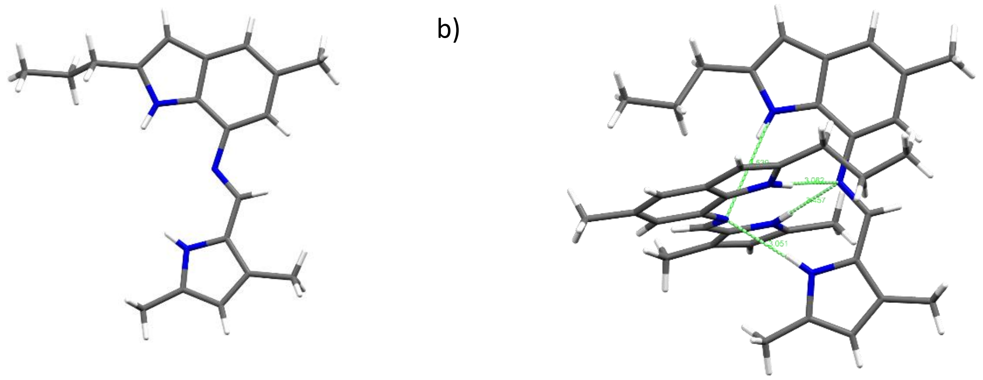

Figure S36. a) Single-crystal X-ray structure of **4**. b) Single-crystal X-ray structure of **4** showing the intermolecular H-bonds and lengths in green.

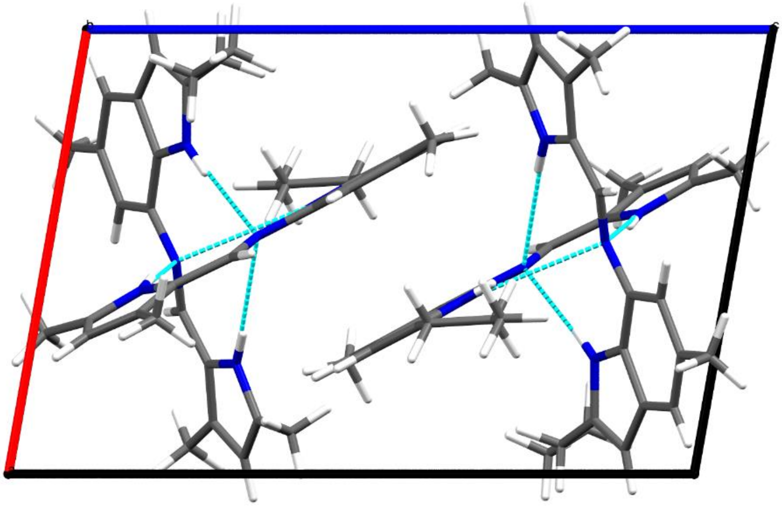

Figure S37. Crystallographic packed diagram of **4** with the unit cell viewed down the *b* axis. Intermolecular bonds are shown in pale blue.

S6. pK_a Studies

S6.1 pK_a Studies in Aqueous Solution

pK_a values for compounds **1d**, **2**, **4**, **5**, **7** and **8** were determined experimentally in aqueous solution through a spectrophotometric procedure using a wavelength that showed the maximum difference in absorbance between the UV-Vis spectra of the protonated and the neutral species. The titration solution was made up with a hexafluorophosphate buffered (100 mM) DMSO:water 50:50 solution (4990 µL) and a transporter solution (10 µL, 25 mM), and the pH of this was recorded. The UV-Vis spectra were recorded on an Agilent Cary 100 UV-Vis spectrophotometer at 25 ˚C with baseline correction, using standard 10 mm quartz glass cells. A baseline absorbance was measured on a hexafluorophosphate buffered (100 mM) DMSO:water 50:50 solution, and then the absorbance of the titration solution was measured. The pH of the solution was decreased stepwise by adding small aliquots of hexafluorophosphoric acid (0.01 mM). After each pH decrease an absorbance measurement was taken. The absorbance at a certain wavelength (noted for each compound below) was plotted against the pH and then fitted using a dose response curve in *OriginPro 9.1*, where the point of inflection corresponds to the apparent pK_a in aqueous solution.

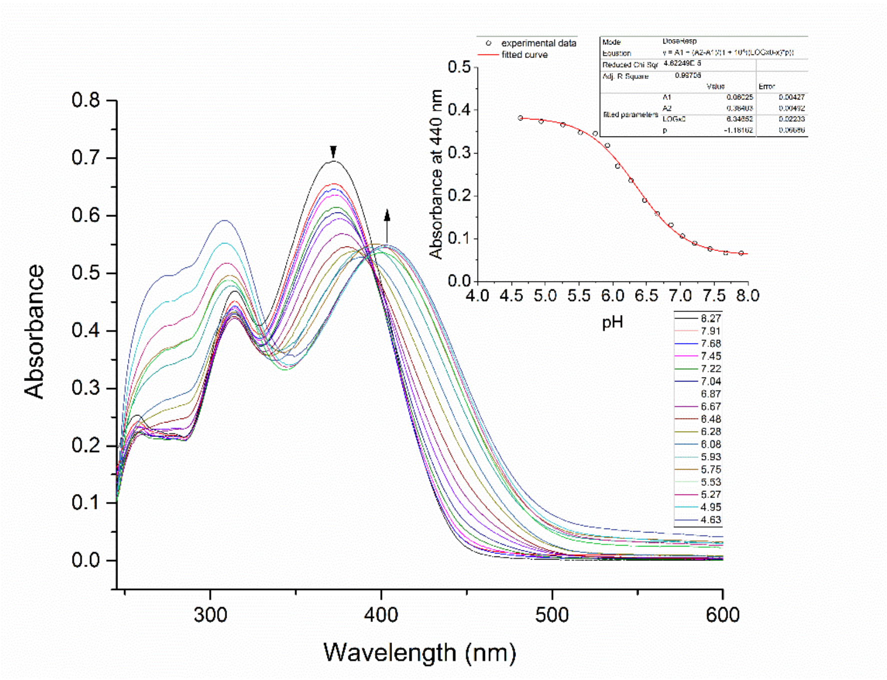

Figure S38. Absorbance spectra taken over the course of a pH-spectrophotometric titration of **1d** (25 mM) in a hexafluorophosphate buffered (100 mM) DMSO:water 50:50 mixture. Inset: Abs vs. pH with dose response curve fit and the point of inflection corresponding to the pK_a value.

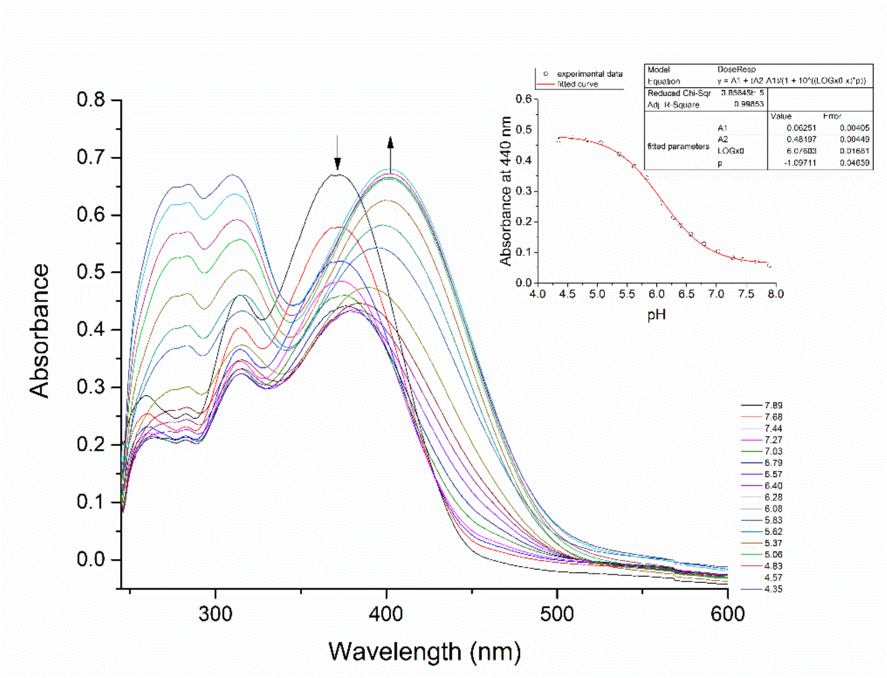

Figure S39. Absorbance spectra taken over the course of a pH-spectrophotometric titration of **2** (25 mM) in a hexafluorophosphate buffered (100 mM) DMSO:water 50:50 mixture. Inset: Abs vs. pH with dose response curve fit and the point of inflection corresponding to the pK_a value.

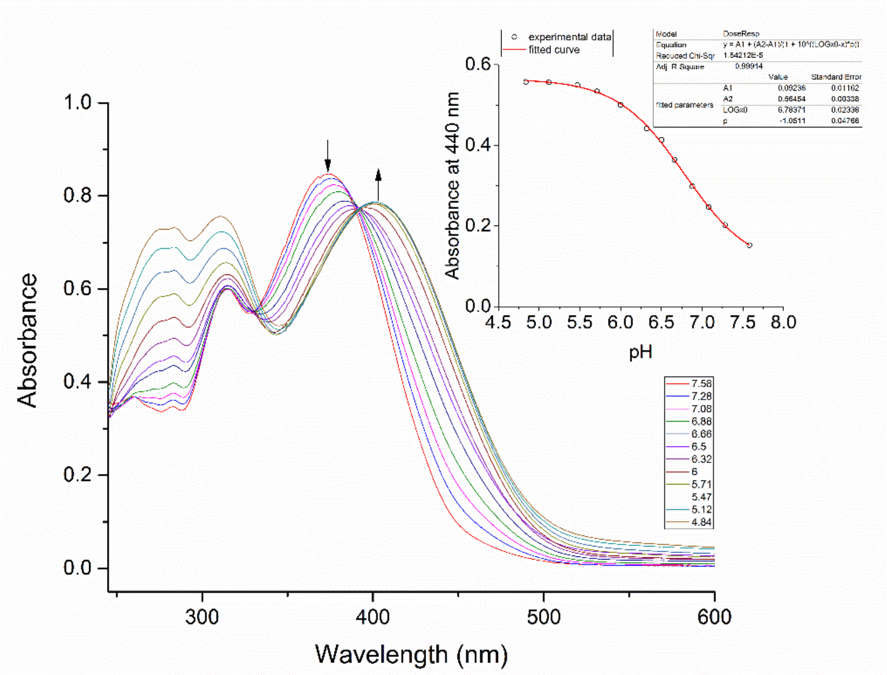

Figure S40. Absorbance spectra taken over the course of a pH-spectrophotometric titration of **4** (25 mM) in a hexafluorophosphate buffered (100 mM) DMSO:water 50:50 mixture. Inset: Abs vs. pH with dose response curve fit and the point of inflection corresponding to the pK_a value.

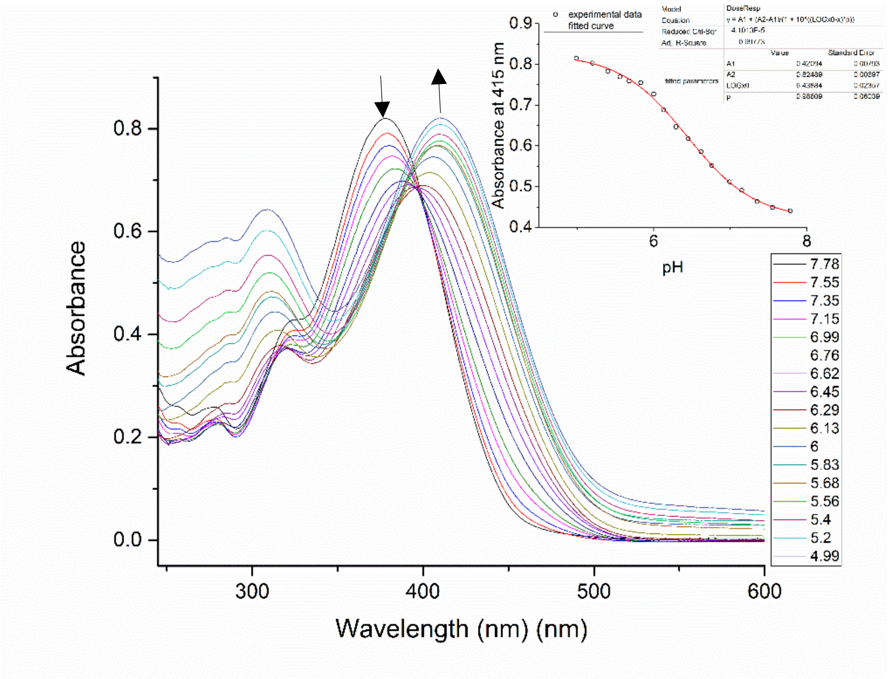

Figure S41. Absorbance spectra taken over the course of a pH-spectrophotometric titration of **5** (25 mM) in a hexafluorophosphate buffered (100 mM) DMSO:water 50:50 mixture. Inset: Abs vs. pH with dose response curve fit and the point of inflection corresponding to the pK_a value.

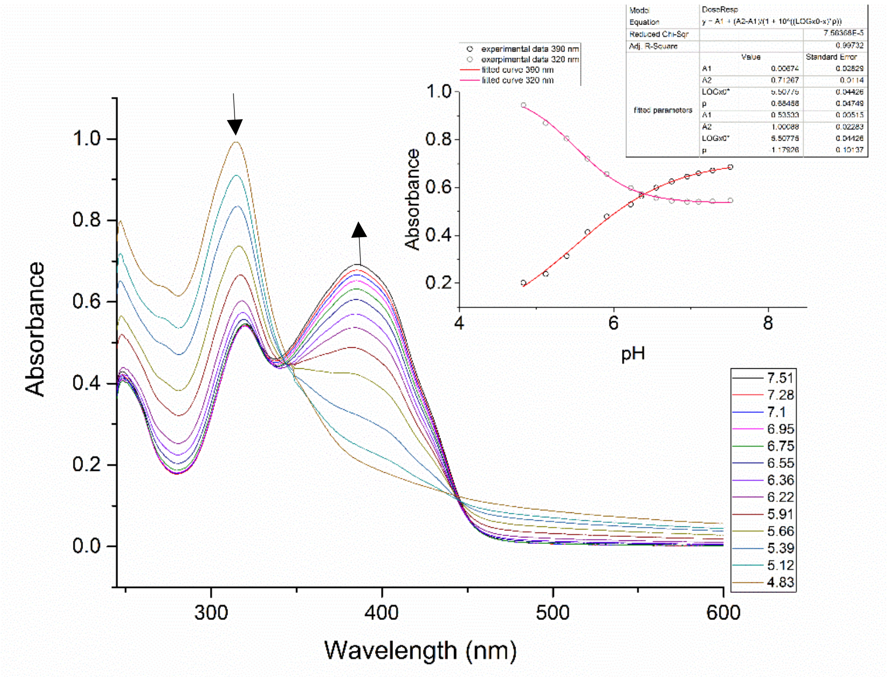

Figure S42. Absorbance spectra taken over the course of a pH-spectrophotometric titration of **7** (25 mM) in a hexafluorophosphate buffered (100 mM) DMSO:water 50:50 mixture. Inset: Abs vs. pH with dose response curve fit and the point of inflection corresponding to the pK_a value.

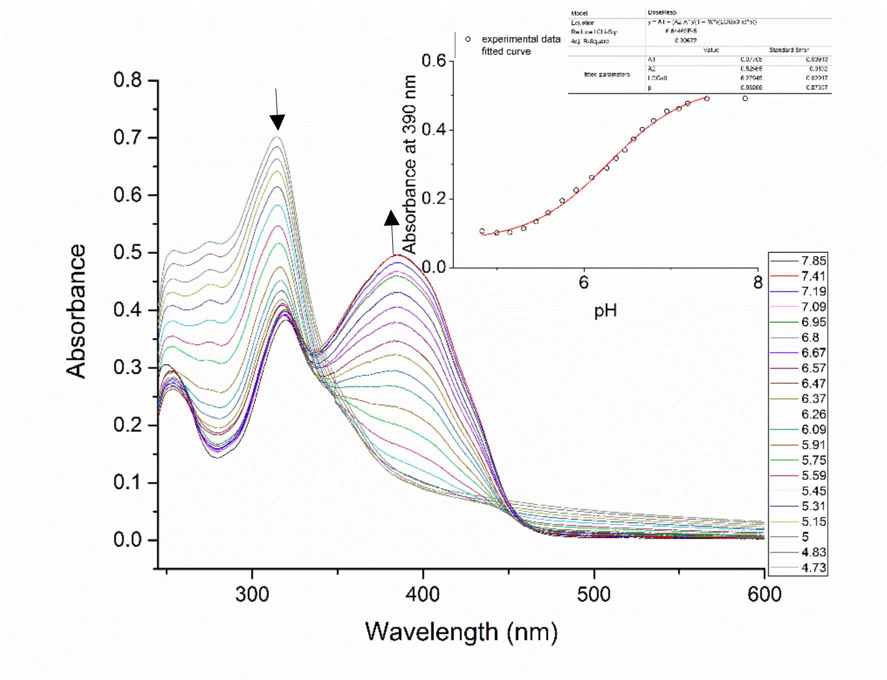

Figure S43. Absorbance spectra taken over the course of a pH-spectrophotometric titration of **8** (25 mM) in a hexafluorophosphate buffered (100 mM) DMSO:water 50:50 mixture. Inset: Abs vs. pH with dose response curve fit and the point of inflection corresponding to the pK_a value.

The pK_a in aqueous solution was determined for all compounds except **3** and **6**; over the course of the titration precipitation was observed for these two compounds because they are not soluble in the solvent system, this led to unreliable titration spectra that could not be fitted to obtain a pK_a value. All spectra obtained have the increasing and decreasing peaks marked with an arrow. Isosbestic points were not observed in the titrations due to break down of the imine in acidic conditions. The breakdown product is shown, for all compounds, as the increasing absorbance peak at ~300 nm.

S6.2 pK_a Studies in Vesicles

pK_a values for compounds **1d**, **2**, **4**, **5**, **7** and **8** were determined experimentally in vesicles through a spectrophotometric procedure using a wavelength that showed the maximum difference in absorbance between the UV-Vis spectra of the protonated and the neutral species. Unilamellar vesicles were prepared using previously reported literature procedures[7-9]. A lipid film of POPC (1-palmitoyl-2-oleoyl-sn-glycero-3-phosphocholine) was prepared from a chloroform solution under reduced pressure and then dried under vacuum for 4 or more hours. The lipid film was rehydrated by vortexing with a solution of phosphate buffered (5 mM) sodium chloride (500 mM). The lipid suspension was then subjected to 9 freeze-thaw cycles and left to rest at room temperature for 30 minutes. After this, the suspension was extruded 25 times through a 400 nm polycarbonate membrane resulting in unilamellar vesicles of a mean diameter of 400 nm. The titration solution was made up to a standard volume (2500 µL, 1 mM) with the lipid (615 µL), phosphate buffered (5 mM) sodium chloride (500 mM) solution (1885 µL) and a transporter solution (10 µL, 25 mM), and the pH of this was noted. The UV-Vis spectra were recorded on an Agilent Cary 100 UV-Vis spectrophotometer at 25 ˚C with baseline correction, using standard 10 mm quartz glass cells. A baseline absorbance was measured using a solution of lipid, phosphate buffered (5 mM) sodium chloride (500 mM) and DMSO, and then the absorbance of the titration solution was measured. The pH of the solution was decreased stepwise by adding small aliquots of hydrochloric acid (0.1 mM).

After each pH decrease an absorbance measurement was taken. The absorbance at a certain wavelength (shown for each compound below) was plotted against the pH and then fitted using a dose response curve in *OriginPro 9.1*, where the point of inflection corresponds to the apparent pK_a in vesicles.

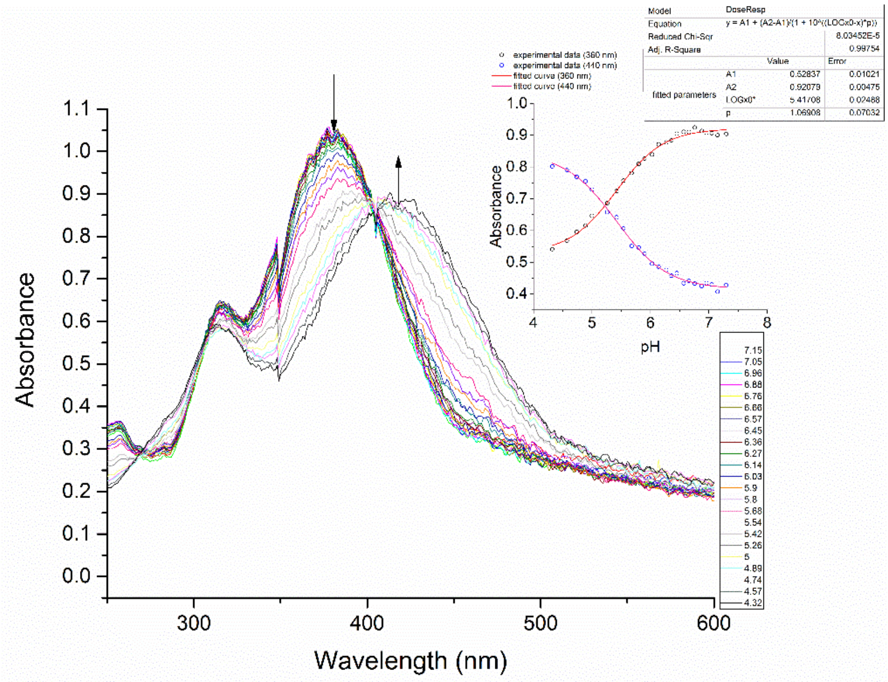

Figure S44. Absorbance spectra taken over the course of a pH-spectrophotometric titration of **1d** (25 mM) in a sodium chloride (500 mM) phosphate buffered (5 mM) lipid solution (1 mM). Inset: Abs vs. pH with dose response curve fit and the point of inflection corresponding to the pK_a value.

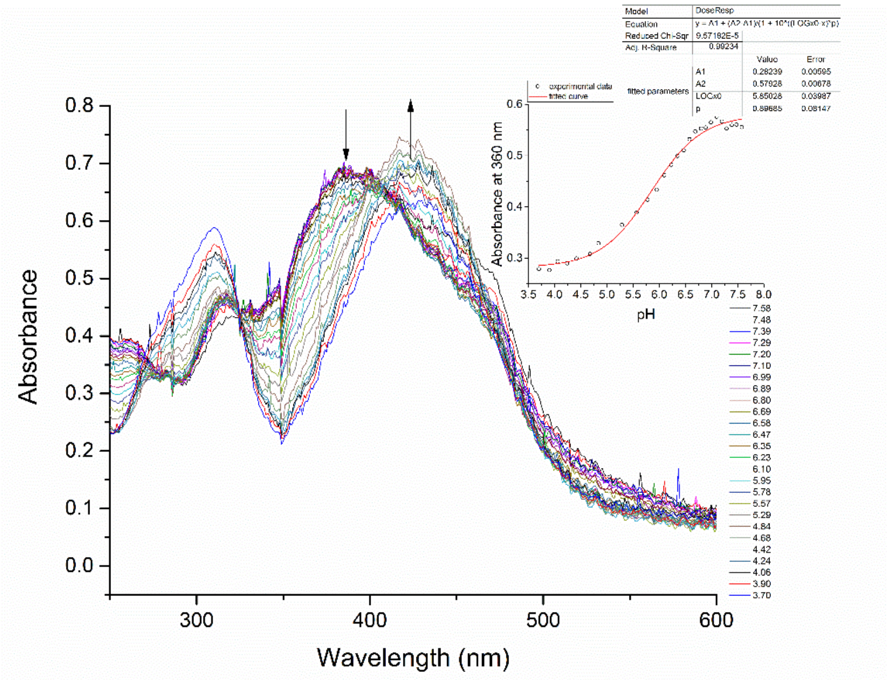

Figure S45. Absorbance spectra taken over the course of a pH-spectrophotometric titration of **2** (25 mM) in a sodium chloride (500 mM) phosphate buffered (5 mM) lipid solution (1 mM). Inset: Abs vs. pH with dose response curve fit with the point of inflection corresponding to the pK_a value.

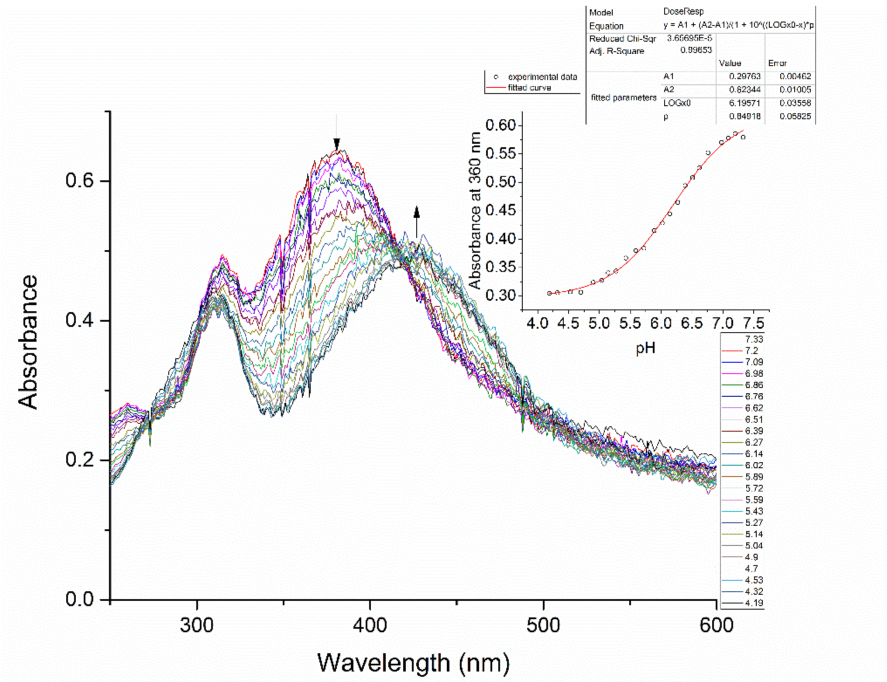

Figure S46. Absorbance spectra taken over the course of a pH-spectrophotometric titration of **4** (25 mM) in a sodium chloride (500 mM) phosphate buffered (5 mM) lipid solution (1 mM). Inset: Abs vs. pH with dose response curve fit and the point of inflection corresponding to the pK_a value.

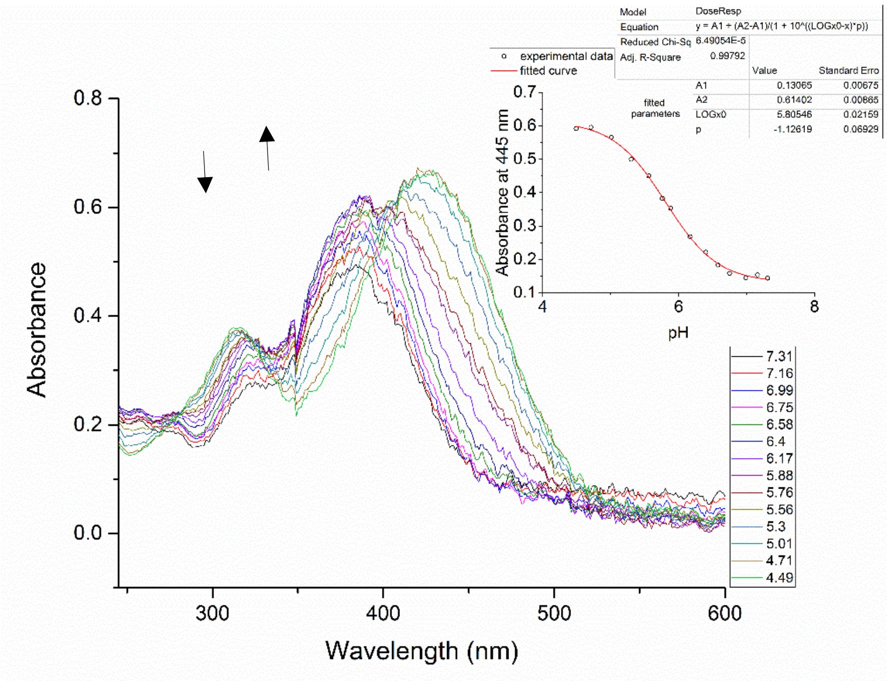

Figure S47. Absorbance spectra taken over the course of a pH-spectrophotometric titration of **5** (25 mM) in a sodium chloride (500 mM) phosphate buffered (5 mM) lipid solution (1 mM). Inset: Abs vs. pH with dose response curve fit and the point of inflection corresponding to the pK_a value.

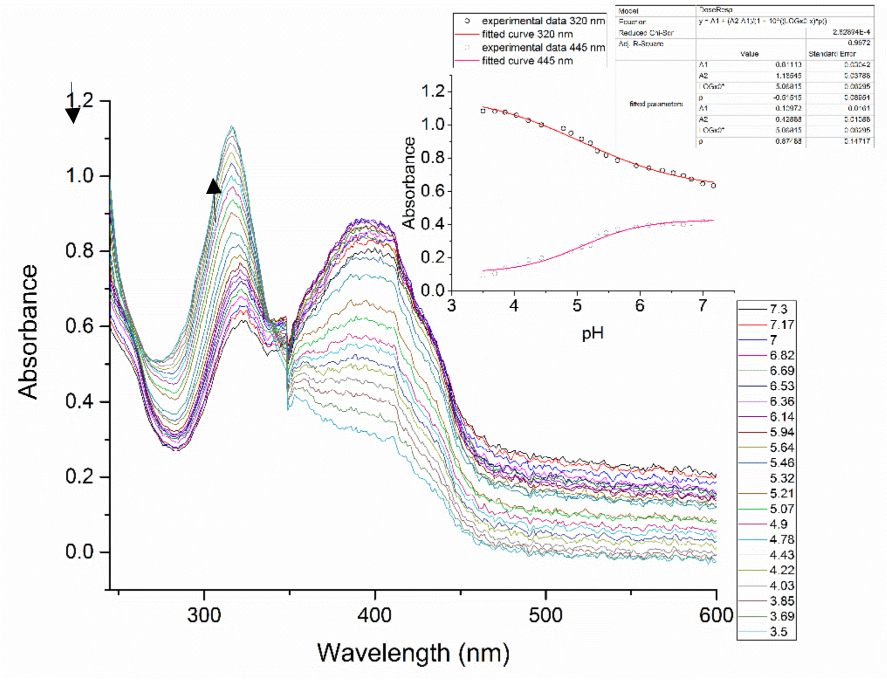

Figure S48. Absorbance spectra taken over the course of a pH-spectrophotometric titration of **7** (25 mM) in a sodium chloride (500 mM) phosphate buffered (5 mM) lipid solution (1 mM). Inset: Abs vs. pH with dose response curve fit and the point of inflection corresponding to the pK_a value.

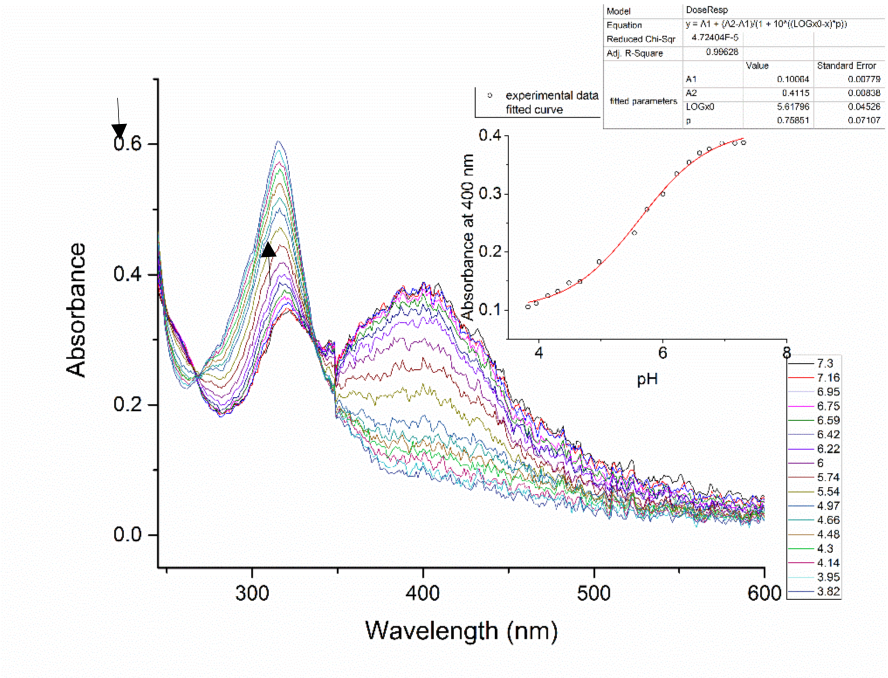

Figure S49. Absorbance spectra taken over the course of a pH-spectrophotometric titration of **8** (25 mM) in a sodium chloride (500 mM) phosphate buffered (5 mM) lipid solution (1 mM). Inset: Abs vs. pH with dose response curve fit and the point of inflection corresponding to the pK_a value.

Discussion:

The pK_a in vesicles was determined for all compounds except **3** and **6**. All spectra obtained have the increasing and decreasing peaks marked with an arrow. Isosbestic points were observed for compounds **7** and **8** because there was a large enough separation between the decreasing peak for the unprotonated species and the increasing peak for the protonated species. Isosbestic points were not observed in the other titrations due to overlap of the decreasing and increasing peaks. Over the course of the titration in vesicles compared to in aqueous solution there is no observable peak at ∼300 nm corresponding to the breakdown product, this is because the compounds are much more stable in the lipid bilayer compared to when they are in aqueous solution.

S7. Stability Studies

S7.1 Stability in Aqueous Solution

Stability studies were performed for the perenosins in aqueous solution using UV-Vis spectroscopy to probe the lifetime of compounds **1d** and **2**-**8** over the course of the transport experiments. The receptors (10 µL, 25 mM) were added to a hexafluorophosphate buffered (100 mM) DMSO:water 50:50 solution (4990 µL) and the pH was adjusted to either 7.2 or 4.6 using hexafluorophosphoric acid. Spectra were collected at 1 minute intervals over a period of 30 minutes.

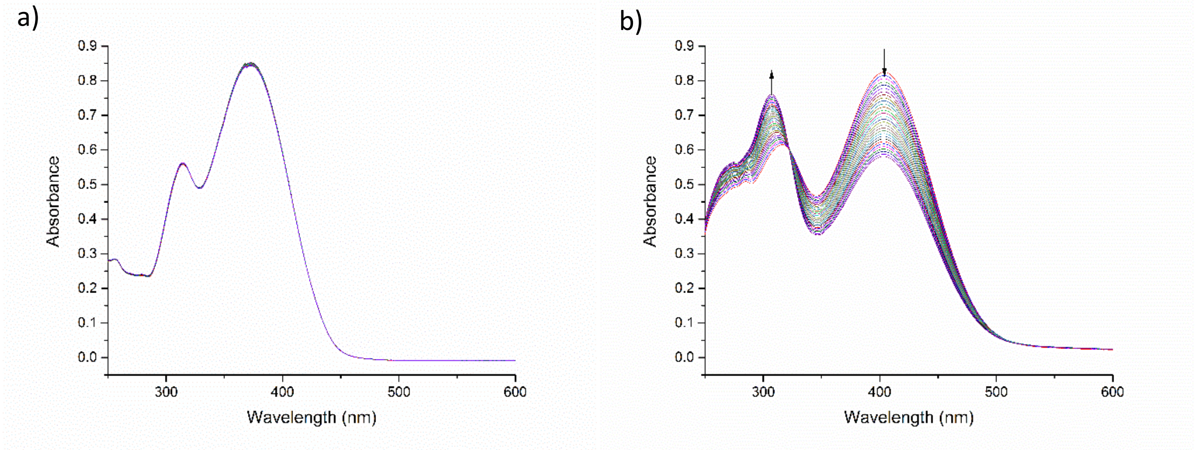

Figure S50. Absorbance spectra taken over 30 minutes of **1d** (25 mM) in a hexafluorophosphate buffered (100 mM) DMSO:water 50:50 mixture. a) pH 7.2 b) pH 4.6

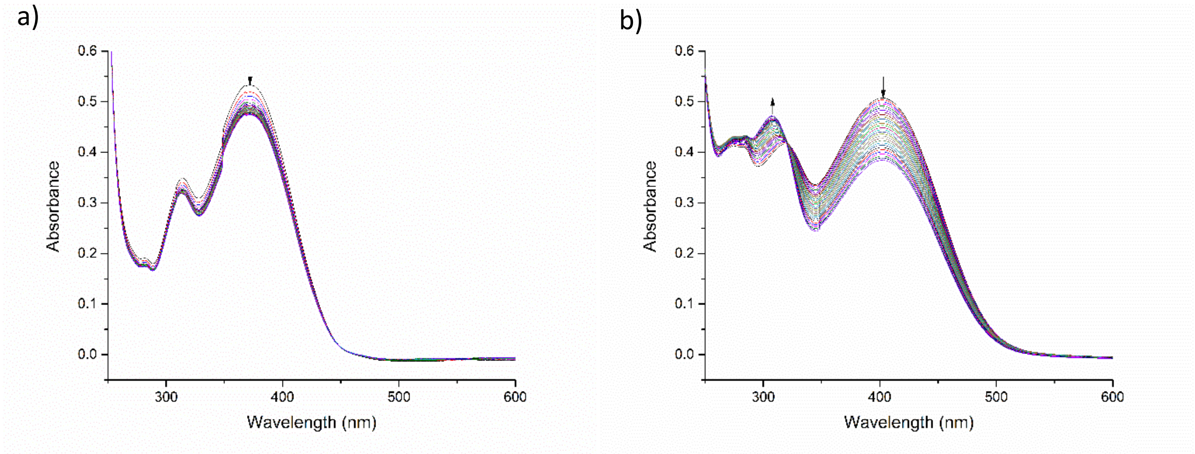

Figure S51. Absorbance spectra taken over 30 minutes of **2** (25 mM) in a hexafluorophosphate buffered (100 mM) DMSO:water 50:50 mixture. a) pH 7.2 b) pH 4.6

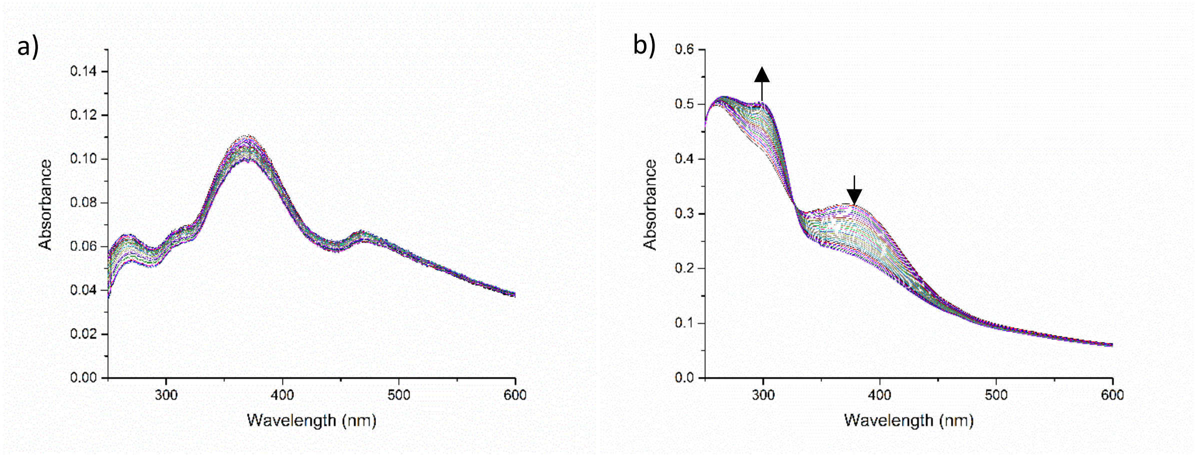

Figure S52. Absorbance spectra taken over 30 minutes of **3** (25 mM) in a hexafluorophosphate buffered (100 mM) DMSO:water 50:50 mixture. a) pH 7.2 b) pH 4.6

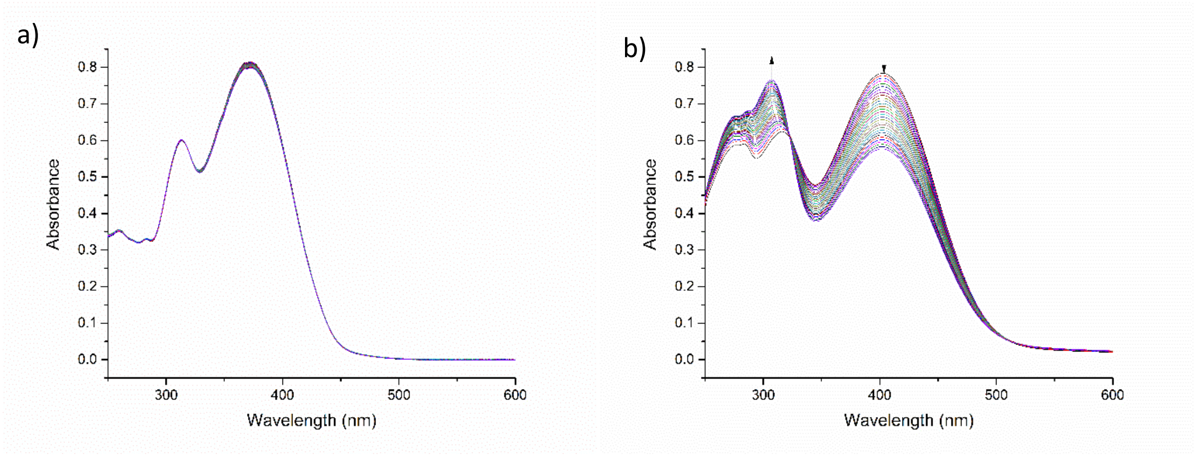

Figure S53. Absorbance spectra taken over 30 minutes of **4** (25 mM) in a hexafluorophosphate buffered (100 mM) DMSO:water 50:50 mixture. a) pH 7.2 b) pH 4.6

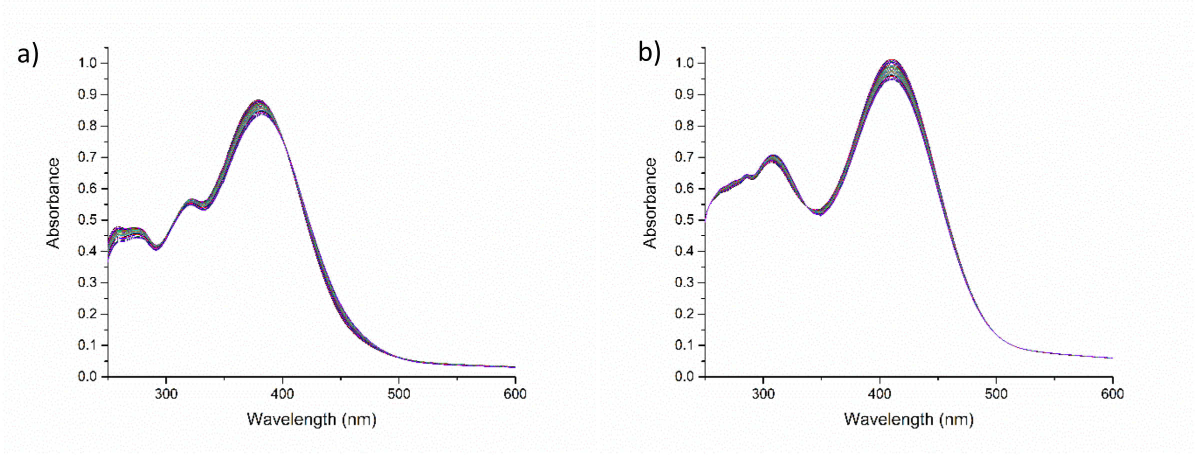

Figure S54. Absorbance spectra taken over 30 minutes of **5** (25 mM) in a hexafluorophosphate buffered (100 mM) DMSO:water 50:50 mixture. a) pH 7.2 b) pH 4.6

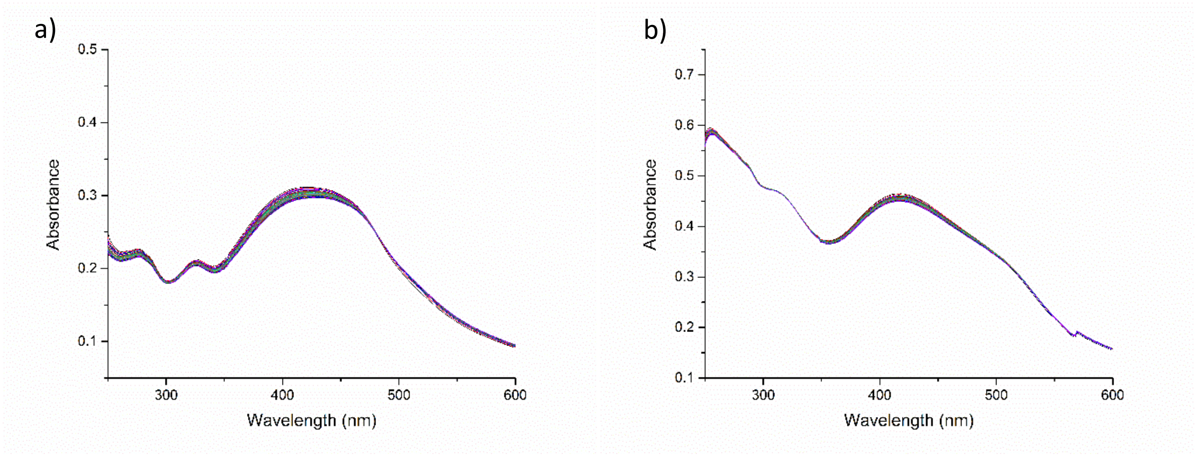

Figure S55. Absorbance spectra taken over 30 minutes of **6** (25 mM) in a hexafluorophosphate buffered (100 mM) DMSO:water 50:50 mixture. a) pH 7.2 b) pH 4.6

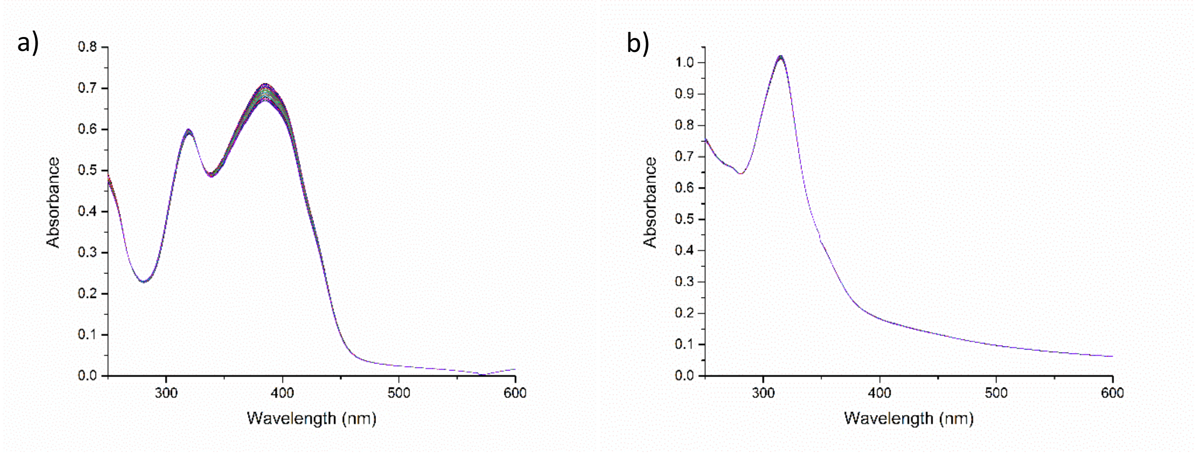

Figure S56. Absorbance spectra taken over 30 minutes of **7** (25 mM) in a hexafluorophosphate buffered (100 mM) DMSO:water 50:50 mixture. a) pH 7.2 b) pH 4.6

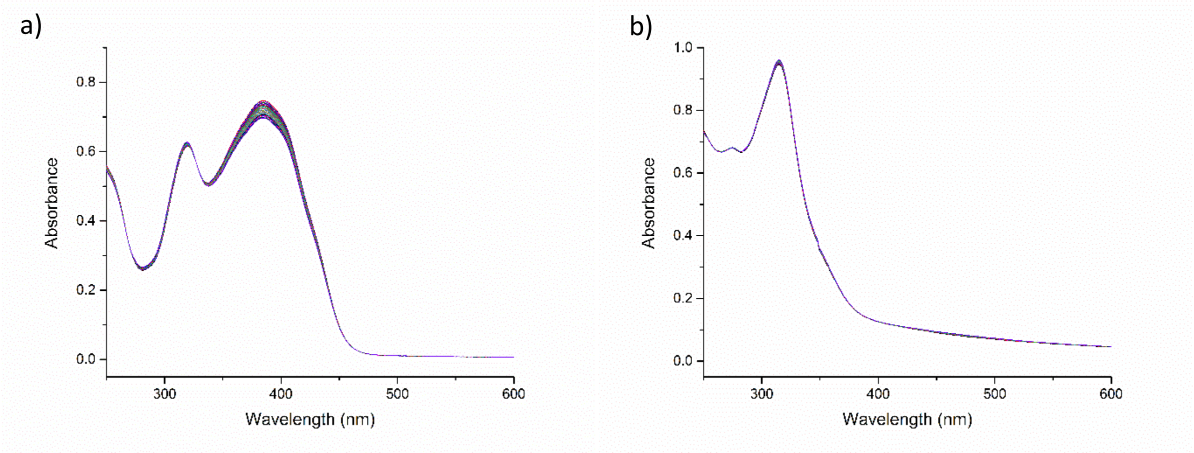

Figure S57. Absorbance spectra taken over 30 minutes of **8** (25 mM) in a hexafluorophosphate buffered (100 mM) DMSO:water 50:50 mixture. a) pH 7.2 b) pH 4.6

Discussion:

The spectra obtained at pH 7.2 for compounds **1d** and **2**-**8** show no notable change in the absorbance over the course of 30 minutes, indicating that the compounds remain stable in these conditions. However, the spectra obtained at pH 4.6 for compounds **1d** and **2**-**4** show a decreasing peak at ~400 nm corresponding to the perenosins, and an increasing peak at ~300 nm corresponding to the breakdown product (both are marked with an arrow). The change in spectra over the 30 minute duration indicates that the compounds are not stable in aqueous solution at pH 4.6. Compounds **5**-**8** show no large change in the absorbance spectra at pH 4.6 suggesting that these compounds are more stable in acidic solutions.

Stability studies were performed for the perenosins in aqueous solution using UV-Vis spectroscopy to probe the lifetime of compounds **1d** and **2**-**8** over the course of the cell studies. The receptors (10 µL, 25 mM) were added to a hexafluorophosphate buffered (100 mM) DMSO:water 50:50 solution (4990 µL) and the pH was adjusted to 7.4 as this was the pH at which the cell tests were done. Spectra were collected periodically over 72 hours to monitor the change in the UV-vis absorbance.

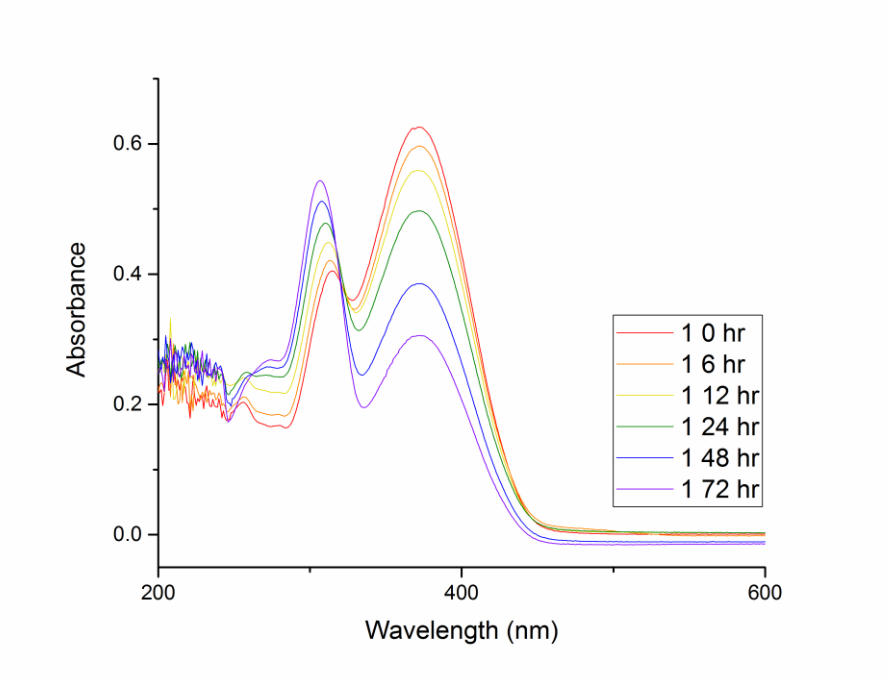

Figure S58. Absorbance spectra taken periodically over 72 hours of **1d** (25 mM) in a hexafluorophosphate buffered (100 mM) DMSO:water 50:50 mixture at pH 7.4

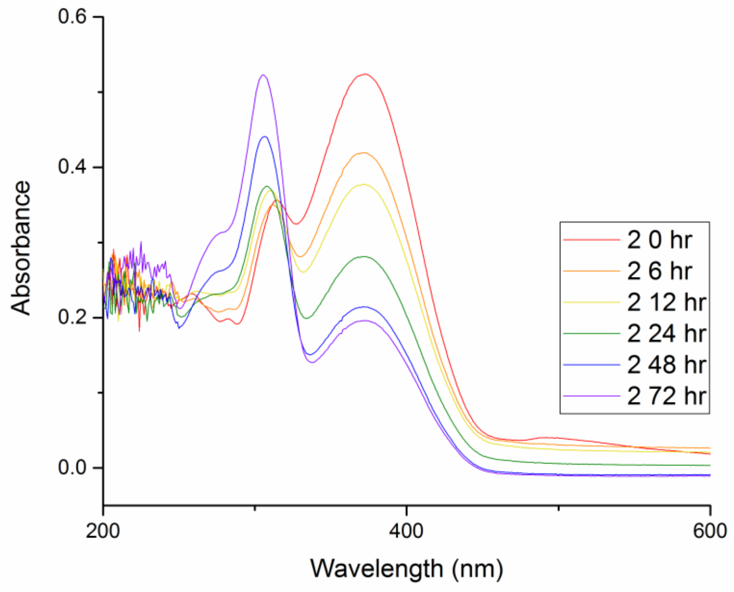

Figure S59. Absorbance spectra taken periodically over 72 hours of **2** (25 mM) in a hexafluorophosphate buffered (100 mM) DMSO:water 50:50 mixture at pH 7.4

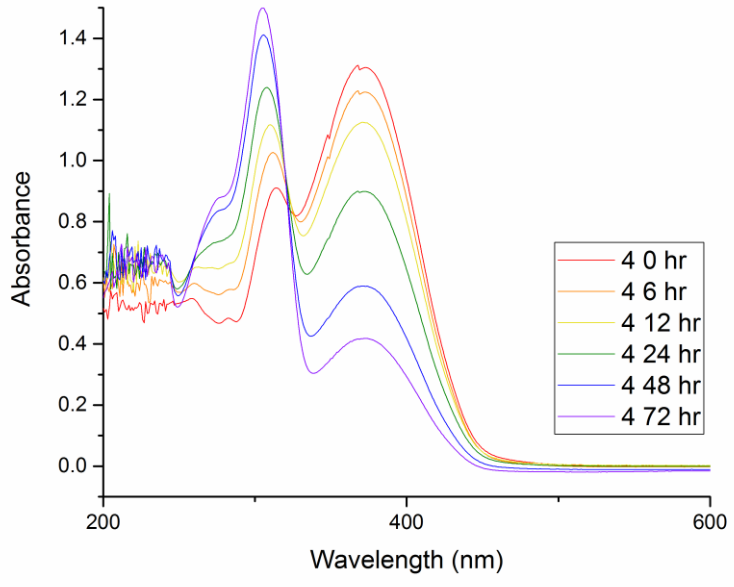

Figure S60. Absorbance spectra taken periodically over 72 hours of **4** (25 mM) in a hexafluorophosphate buffered (100 mM) DMSO:water 50:50 mixture at pH 7.4

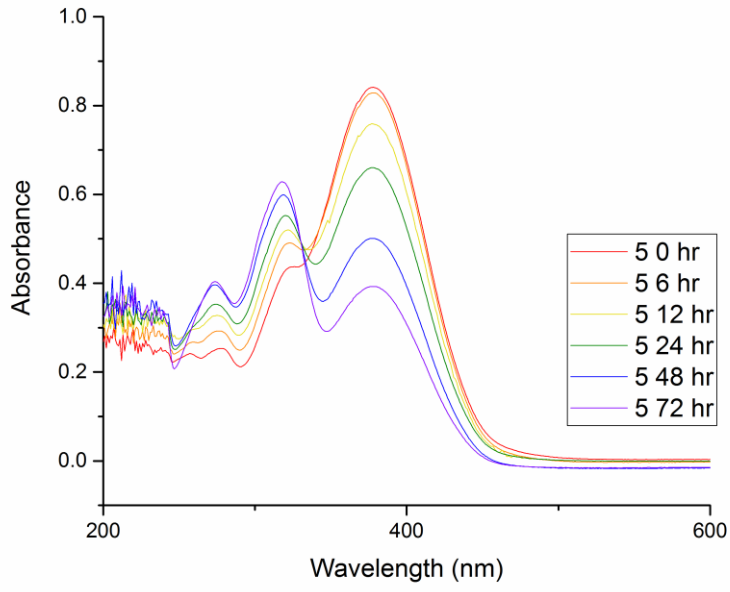

Figure S61. Absorbance spectra taken periodically over 72 hours of **5** (25 mM) in a hexafluorophosphate buffered (100 mM) DMSO:water 50:50 mixture at pH 7.4

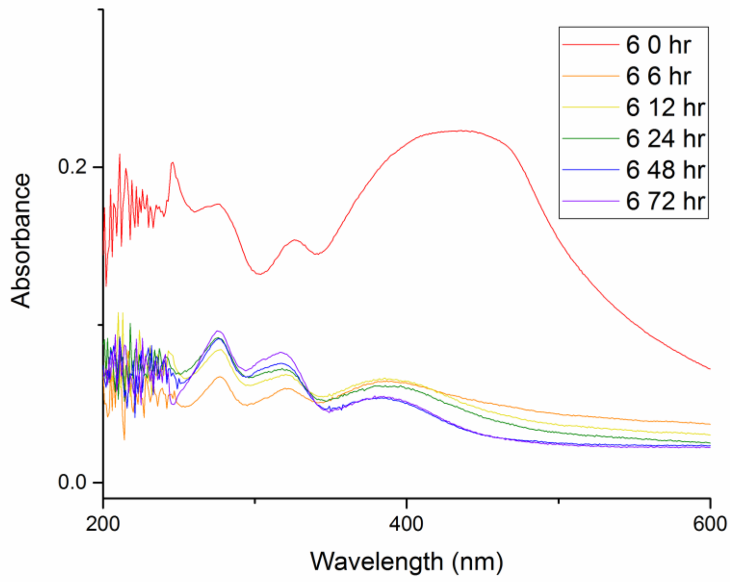

Figure S62. Absorbance spectra taken periodically over 72 hours of **6** (25 mM) in a hexafluorophosphate buffered (100 mM) DMSO:water 50:50 mixture at pH 7.4

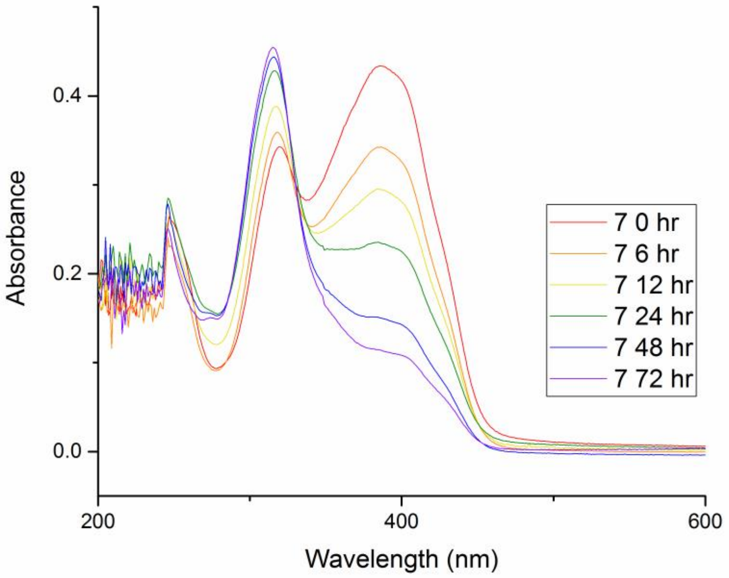

Figure S63. Absorbance spectra taken periodically over 72 hours of **7** (25 mM) in a hexafluorophosphate buffered (100 mM) DMSO:water 50:50 mixture at pH 7.4

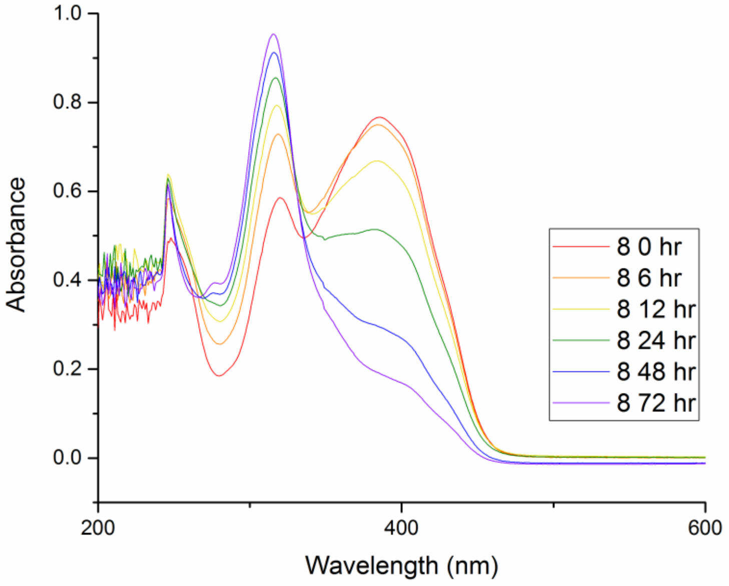

Figure S64. Absorbance spectra taken periodically over 72 hours of **8** (25 mM) in a hexafluorophosphate buffered (100 mM) DMSO:water 50:50 mixture at pH 7.4

Discussion:

The spectra obtained over the course of 72 hours for **1d**, **2** and **4**-**8** show similar trends, **3** was not tested because it was unavailable. All spectra show a decrease in the peak at around 400 nm after 6 hours and an increase in the peak at around 300 nm after 72 hours. These changes in spectra correspond to the breakdown of the compound suggesting that they are unstable over the course of 72 hours. The absorbance for **6** decreases dramatically for the readings after 6 hours because most of the compound had precipitated out of the solution due to poor solubility.

S7.2 Stability in Vesicles

Stability studies were performed for the perenosins in vesicles using UV-Vis spectroscopy to probe the lifetime of compounds **1d** and **2**-**8** at pH 7.2 and pH 4.6. Unilamellar vesicles were prepared using previously reported literature procedures[7-9]. A lipid film of POPC (1-palmitoyl-2-oleoyl-sn-glycero-3-phosphocholine) was prepared from a chloroform solution under reduced pressure and then dried under vacuum for 4 or more hours. The lipid film was rehydrated by vortexing with a solution of phosphate buffered (5 mM) sodium chloride (500 mM). The lipid suspension was then subjected to 9 freeze-thaw cycles and left to rest at room temperature for 30 minutes. After this, the suspension was extruded 25 times through a 400 nm polycarbonate membrane resulting in unilamellar vesicles of a mean diameter of 400 nm. The receptors (10 µL, 25 mM) were added to a solution of lipid (615 µL) in phosphate buffered (5 mM) sodium chloride (500 mM) solution (1885 µL) and the pH was adjusted to 7.2 or 4.6 using hydrochloric acid. Spectra were collected at 1 minute intervals over a period of 2 hours.

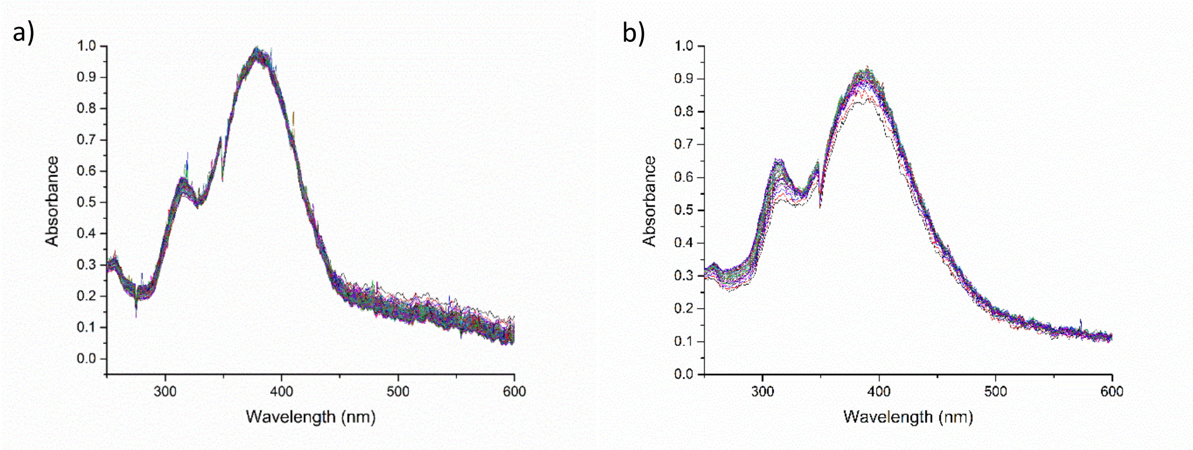

Figure S65. Absorbance spectra taken over 2 hours of **1d** (25 mM) in a sodium chloride (500 mM) phosphate buffered (5 mM) lipid solution. a) pH 7.2 b) pH 4.6

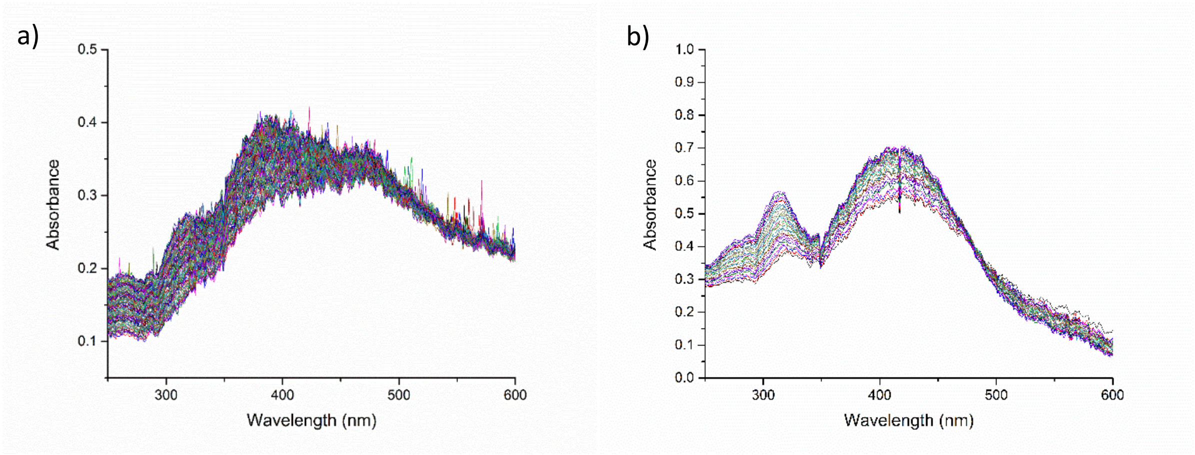

Figure S66. Absorbance spectra taken over 2 hours of **2** (25 mM) in a sodium chloride (500 mM) phosphate buffered (5 mM) lipid solution. a) pH 7.2 b) pH 4.6

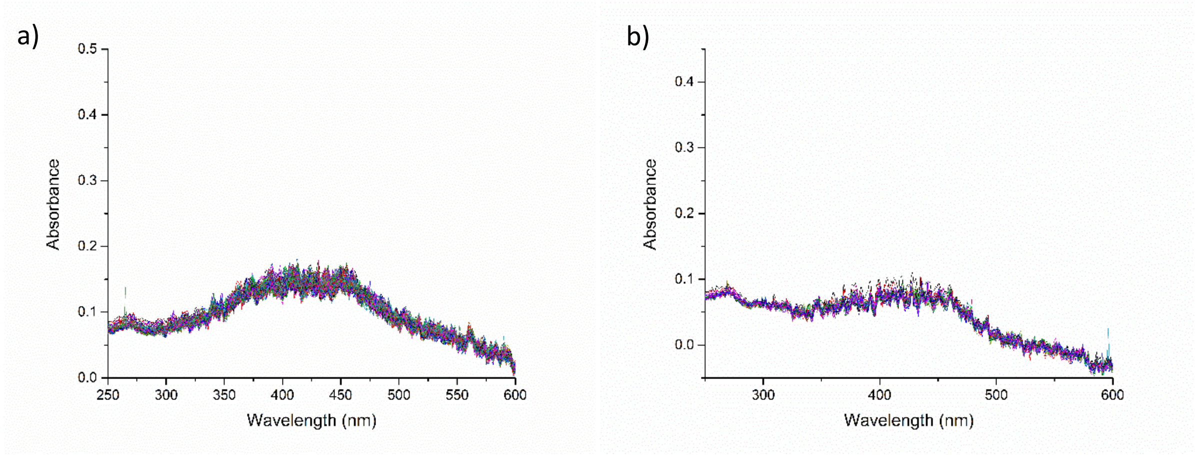

Figure S67. Absorbance spectra taken over 2 hours of **3** (25 mM) in a sodium chloride (500 mM) phosphate buffered (5 mM) lipid solution. a) pH 7.2 b) pH 4.6

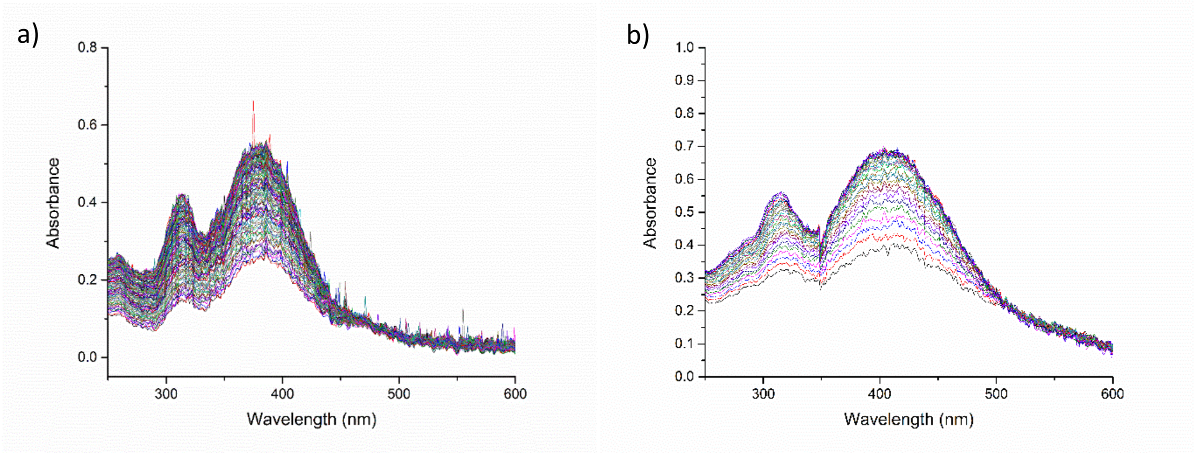

Figure S68. Absorbance spectra taken over 2 hours of **4** (25 mM) in a sodium chloride (500 mM) phosphate buffered (5 mM) lipid solution. a) pH 7.2 b) pH 4.6

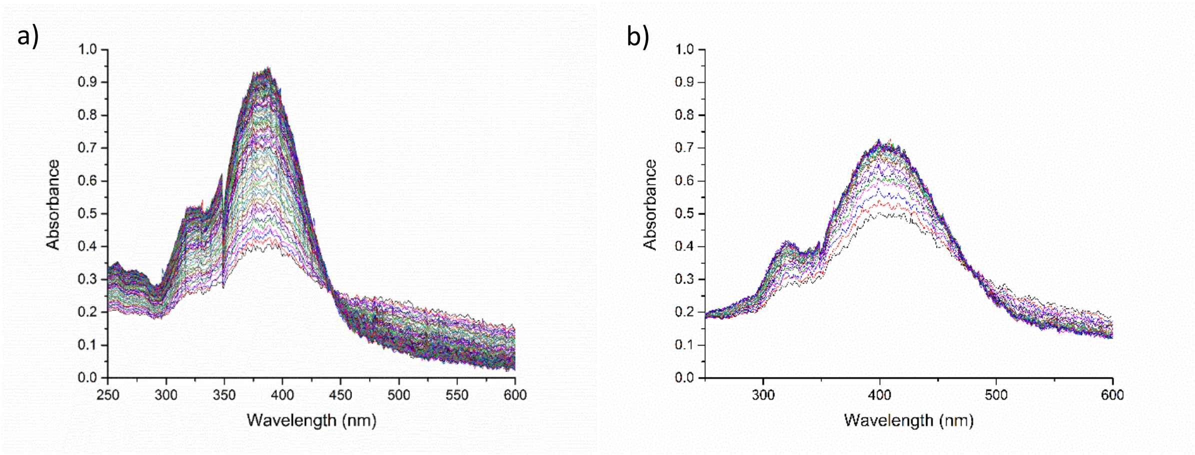

Figure S69. Absorbance spectra taken over 2 hours of **5** (25 mM) in a sodium chloride (500 mM) phosphate buffered (5 mM) lipid solution. a) pH 7.2 b) pH 4.6

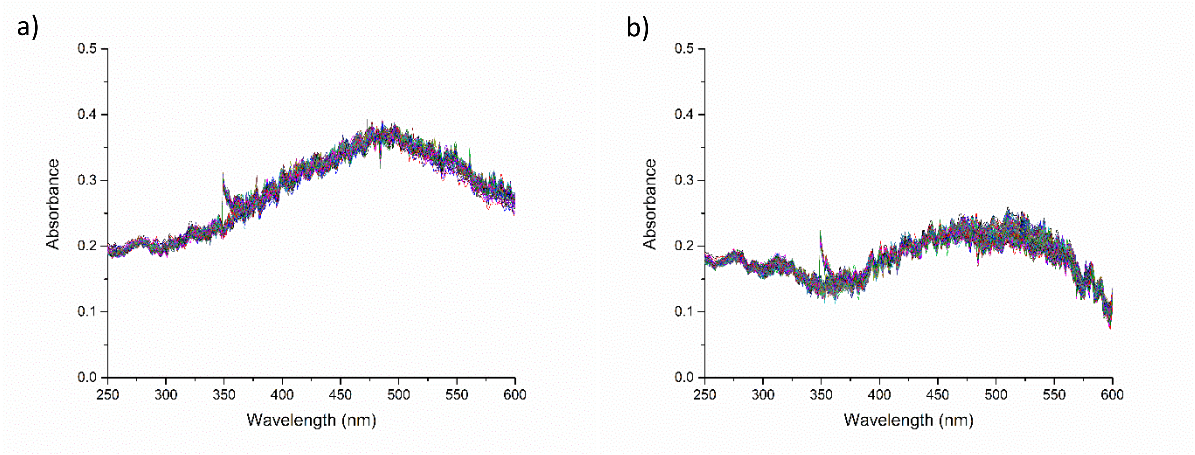

Figure S70. Absorbance spectra taken over 2 hours of **6** (25 mM) in a sodium chloride (500 mM) phosphate buffered (5 mM) lipid solution. a) pH 7.2 b) pH 4.6

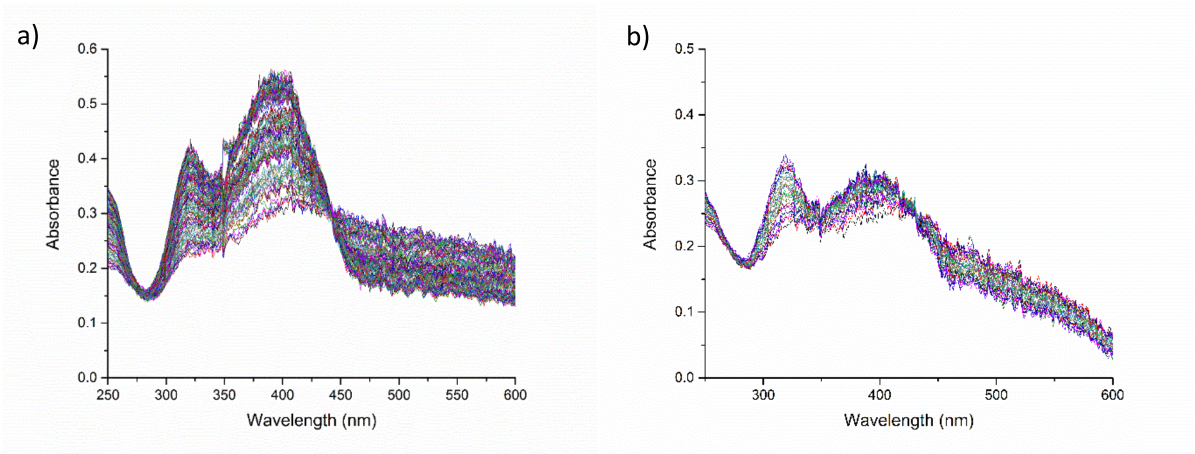

Figure S71. Absorbance spectra taken over 2 hours of **7** (25 mM) in a sodium chloride (500 mM) phosphate buffered (5 mM) lipid solution. a) pH 7.2 b) pH 4.6

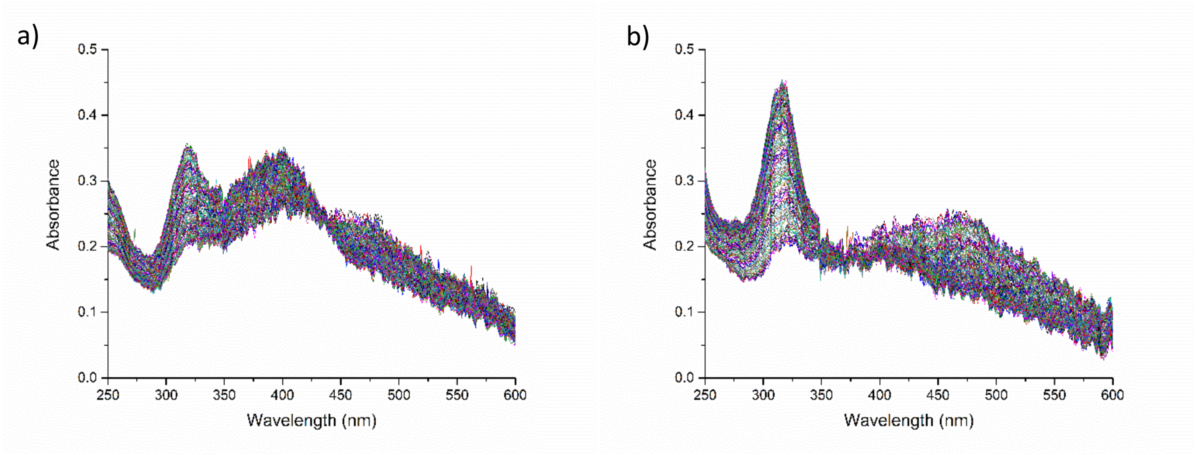

Figure S72. Absorbance spectra taken over 2 hours of **8** (25 mM) in a sodium chloride (500 mM) phosphate buffered (5 mM) lipid solution. a) pH 7.2 b) pH 4.6

Discussion:

The spectra obtained at pH 7.2 for compounds **1d**, **3** and **6** show no notable change in the absorbance over the course of 2 hours. Compound **1d** shows a large absorbance peak and this suggests it remains stable in these conditions, whereas compounds **3** and **6** do not show a large absorbance peak indicating that the absorbance of this compound is not as good in the presence of vesicles. The spectra obtained at pH 7.2 for compounds **2**, **4**, **5**, **7** and **8** show an increase in absorbance over the course of 2 hours which indicates slow diffusion of the transporter into and out of the lipid bilayer. There is no observable increasing peak corresponding to the breakdown products at ~300 nm suggesting that all the compounds tested are stabilised by the lipid bilayer at pH 7.2. At pH 4.6 the same trend is observed, with compounds **1d**, **3**, and **6** showing no significant change in the absorbance spectra and compounds **2**, **4**, **5**, **7** and **8** showing an increase in the absorbance spectra. This corresponds to slow diffusion of the transporters into the lipid bilayer. There is no peak observed at ~300 nm in the absorbance spectra for all compounds at pH 4.6 in vesicles, indicating that the compounds are stable in these conditions for at least 2 hrs.

S8. ^{1}H NMR Titration Binding Studies

S8.1. Equipment and Sample Preparation

^{1}H NMR titrations were performed on a Bruker Avance AVII400 FT-NMR spectrometer with either SEF 1H/D-19F or DUL D/1H-13C, 5 mm z-gradient probes, operating at a frequency of 400 MHz for ^{1}H NMR with the probe temperature maintained at 298 K unless otherwise stated as determined using a variable temperature unit (± 5 K). For all NMR titrations, a constant host concentration was maintained (~ 5.0 mM) by using the host solution to dissolve the guest to make the guest stock solution. Over the course of the titration Hamiltonian Microlitre syringes were used to add aliquots of the guest stock solution to the NMR sample of the host solution.

The anions were added as the tetrabutylammonium (TBA) salts after being dried under high vacuum (< 1.0 mmHg) for 24 h. Stock solutions of the host (~ 5.0 mM) were prepared in a DMSO-d_6/0.5%H_2O mixture. In some titrations, protonation of the host species was achieved through the addition of 1 eq. hexafluorophosphoric acid (HPF_6) to the host stock solution. The host stock solutions, with or without addition of HPF_6, (500 µL) were transferred to an air-tight screw-cap NMR sample tube (5 mm ID) and the same host stock solution was used to prepare the standard guest titrant solution containing approximately 100 mM of the TBA-anion salts. This ensured a constant concentration of the host (~ 5.0 mM) for the duration of the titration experiment.

S8.2. Titration Procedure

Over the course of the titration small aliquots (2-100 µL) of the standard guest solution were added to the host solution (~ 500 µL) in the NMR tube. For each titration 15-20 data points were collected and at the end of the titration approximately 10 equivalents of the guest anion salt were present.

Upon each addition of the standard guest solution the samples were thoroughly shaken in the NMR tube and then allowed to equilibrate for up to 2 minutes inside the NMR probe before the spectra were taken. Throughout each titration experiment all parameters of the NMR spectrometer remained constant.

S8.3. Titration Data Fitting

For each ^{1}H NMR titration, all the proton resonances were monitored for changes in chemical shift. In the case of protonated host species at least one proton resonance was followed and for the neutral host species two or more proton resonances were followed, which allowed several data sets to use in determination of the association constant (K_a). Global fitting takes into account all data sets at the same time and this improves the quality of the nonlinear curve fitting. The supramolecular.org web applet[10] was used to fit the collected titration data and allows the use of different binding models to fit the data in the best way. For compounds **1d** and **2**-**8** firstly the 1:1 binding model was attempted and if it did not give a complete fit the 2:1 binding model, either full cooperative or additive, was used. The quality of fit is determined by the covariance of the fit (cov_{fit}), which is calculated by dividing the (co)variance of the residual (experimental data − calculated data) with the covariance of the experimental data. A covariance of fit factor (F cov_{fit}) can be obtained by dividing the cov_{fir} from the 1:1 binding model by the cov_{fit} from the 2:1 binding model. Due to the higher number of parameters from the 2:1 binding model an improvement in the cov_{fit} (i.e. F cov_{fit}) by a factor greater than 5 indicates that the 2:1 binding model is preferential.[11]

S.8.4 ^{1}HNMR Titration Spectra and Fitted Binding Isotherms

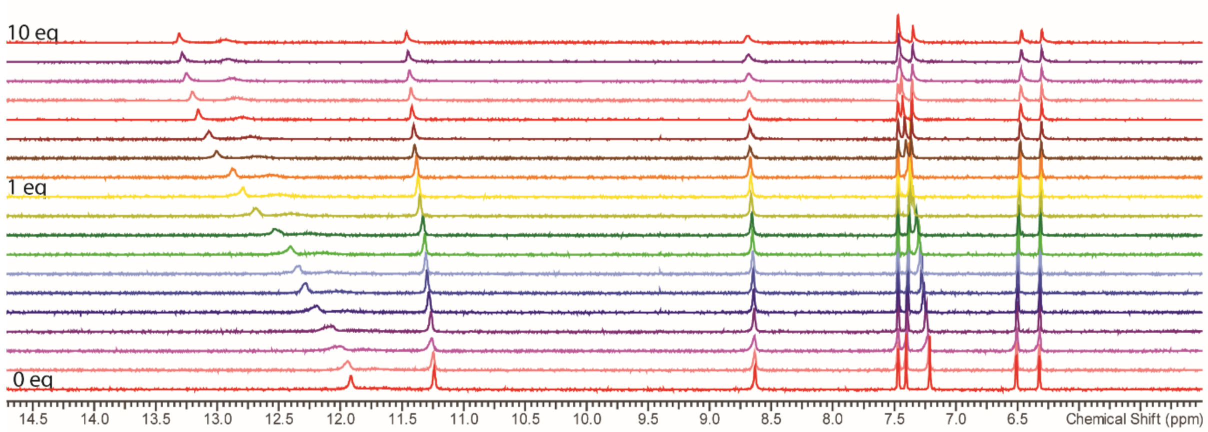

Figure S73. Stack plot of NMR spectra for compound **1d** + HPF_6 + TBACl

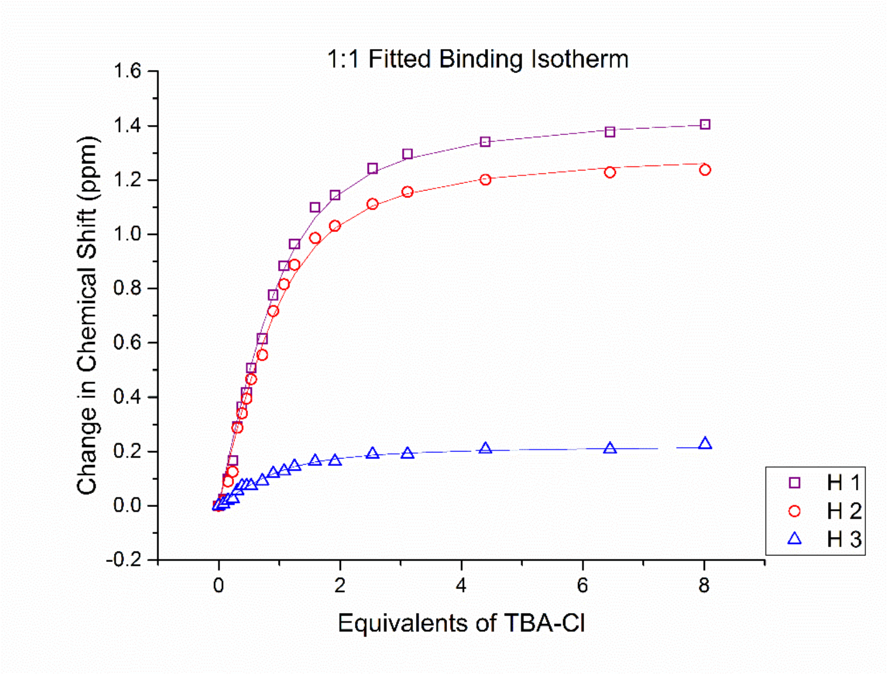

Figure S74. Fitted binding isotherm of compound [**1d** + HPF_6] with TBA-Cl in DMSO-d_6 showing the change in chemical shift of the NH protons fitted to the 1:1 binding model. K_a: 605 M^{-1}; covariance of fit: 3.3 x 10^{-3}.

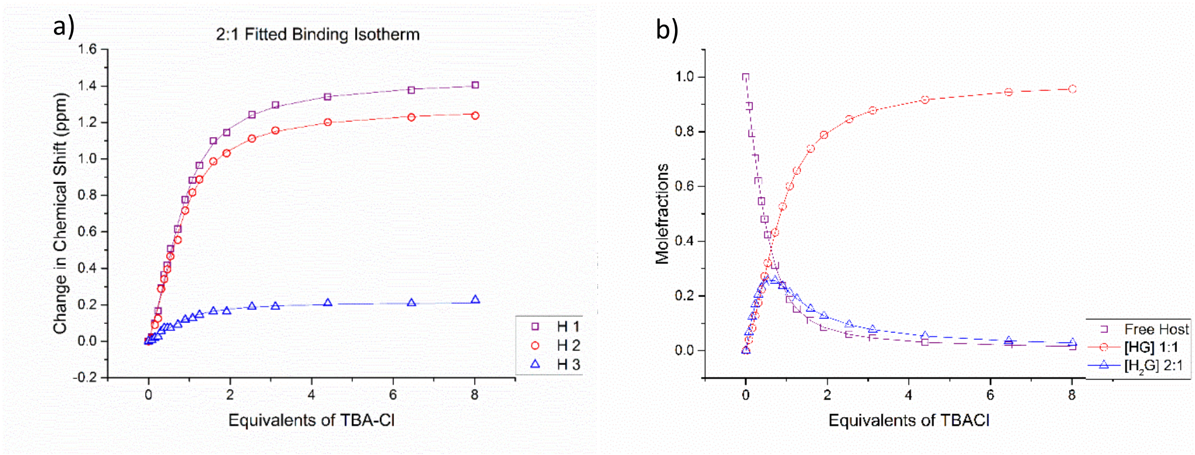

Figure S75. a) Fitted binding isotherm of compound [**1d** + HPF_6] with TBA-Cl in DMSO-d_6 showing the change in chemical shift of the NH protons fitted to the 2:1 full cooperative binding model. K_{11}: 1750 M^{-1}; K_{21}: 190 M^{-1}; covariance of fit: 1.5 x 10^{-3}. b) Molefractions included to show how the composition of the mixture of complexes changes over the course of the titration.

Discussion:

Based on the F cov_{fit} (2.2) and inspection of the binding isotherms, both the 1:1 and 2:1 model can describe this data. Therefore concluding that the derived association constants from both models should be considered.

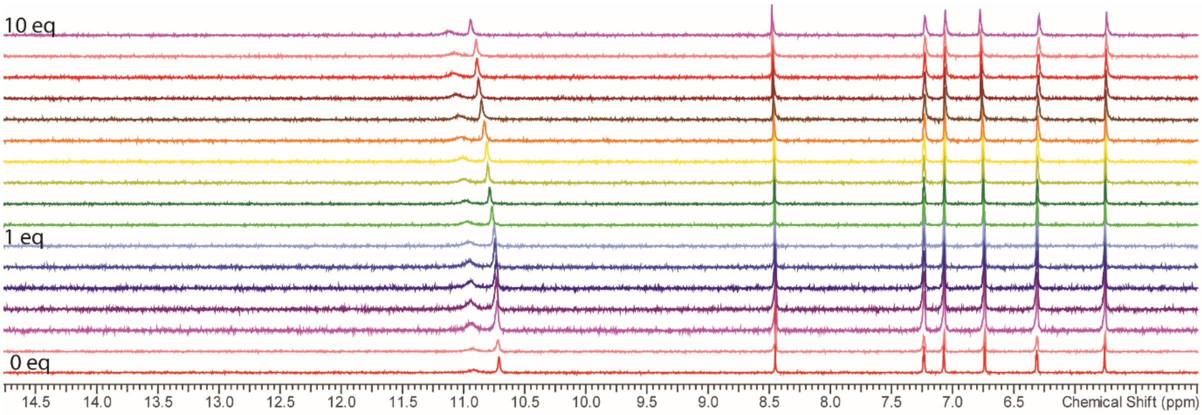

Figure S76. Stack plot of NMR spectra for compound **1d** + TBACl

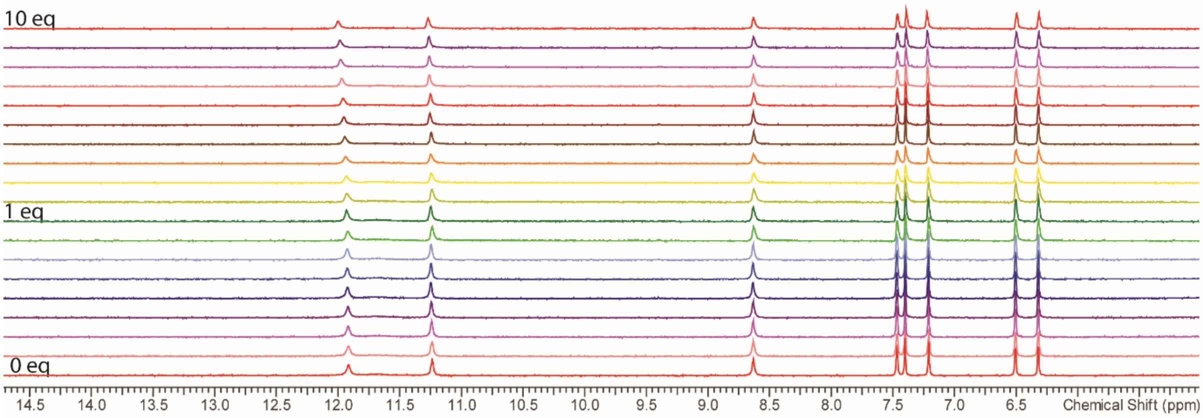

Figure S77. Stack plot of NMR spectra for compound **1d** + HPF_6 + $TBANO_3$

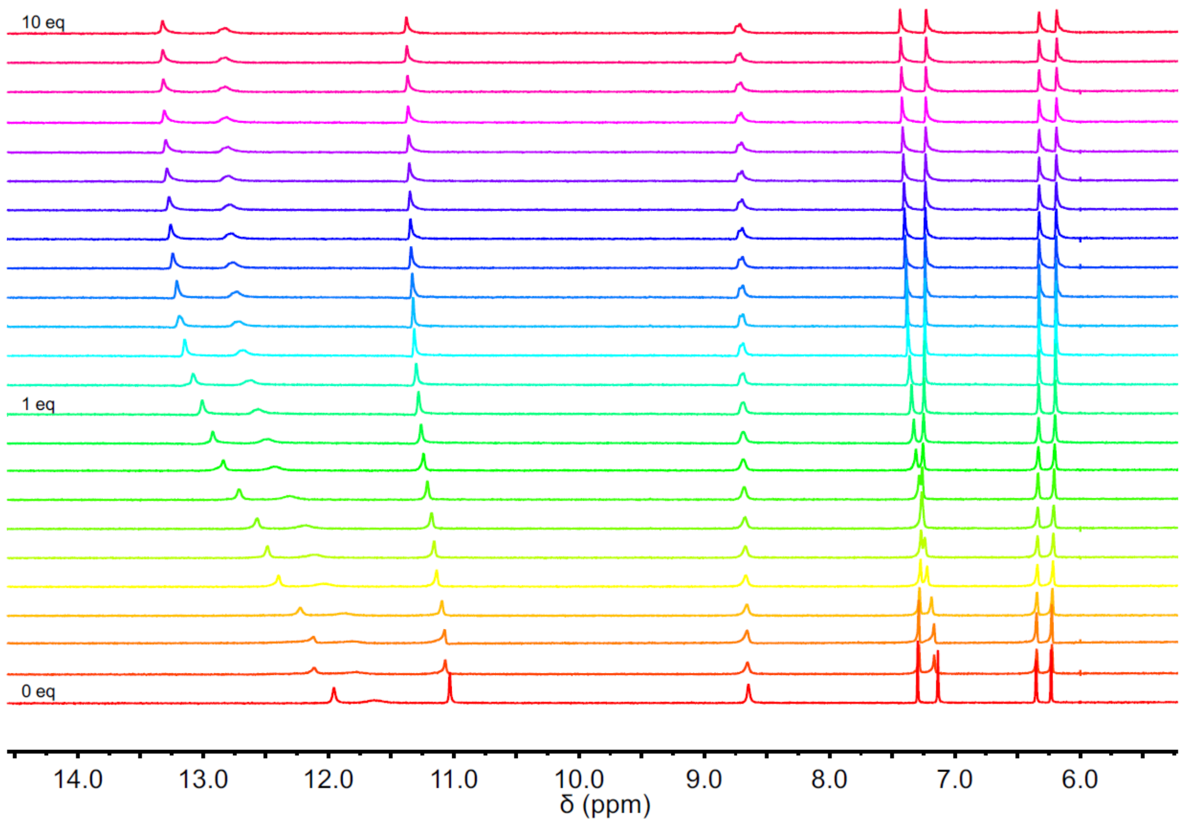

Figure S78. Stack plot of NMR spectra for compound **2** + HPF_6 + TBACl

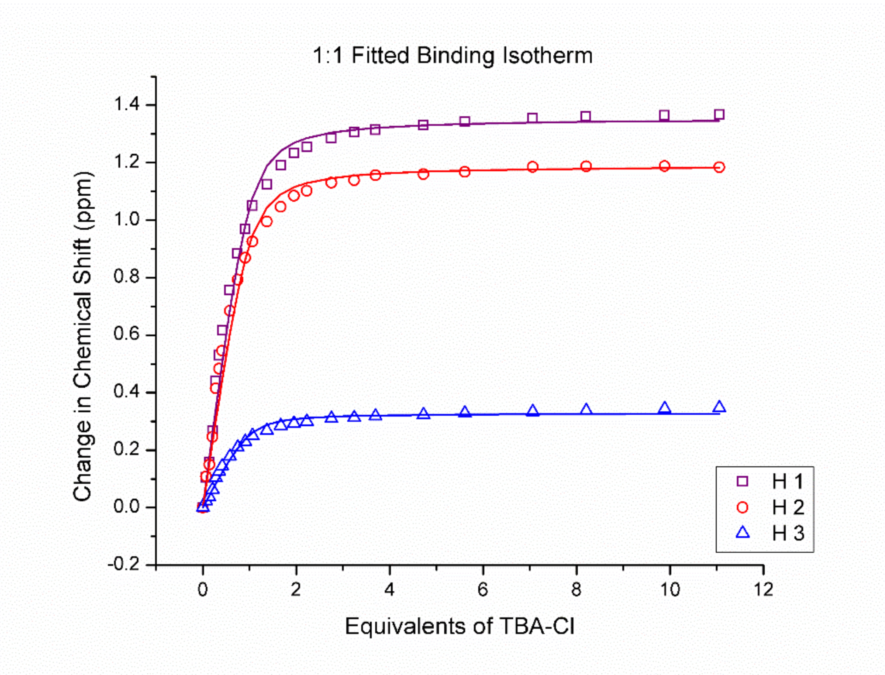

Figure S79. Fitted binding isotherm of compound [**2** + HPF_6] with TBA-Cl in DMSO-d_6 showing the change in chemical shift of the NH protons fitted to the 1:1 binding model. K_a: 2450 M^{-1}; covariance of fit: 6.2 x 10^{-3}.

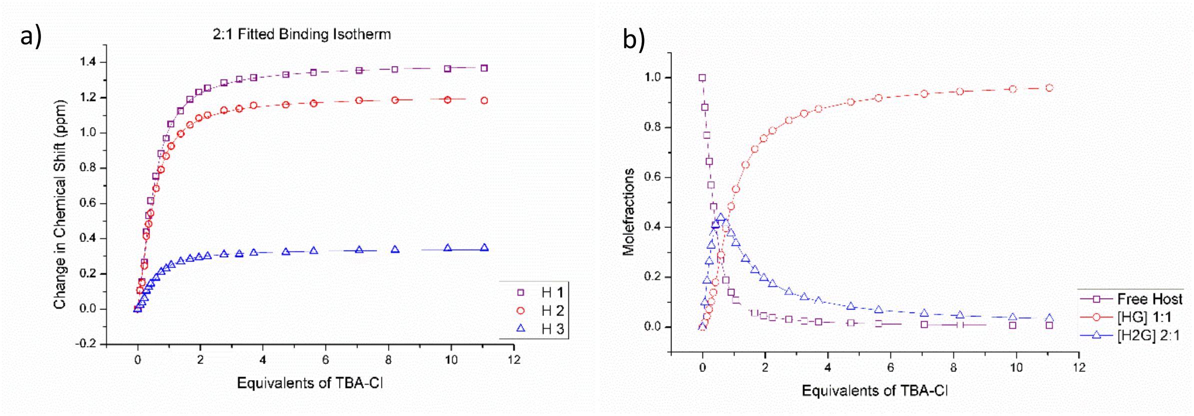

Figure S80. a) Fitted binding isotherm of compound [**2** + HPF_6] with TBA-Cl in DMSO-d_6 showing the change in chemical shift of the NH protons fitted to the 2:1 full cooperative binding model. K_{11}: 2500 M^{-1}; K_{21}: 473 M^{-1}; covariance of fit: 1.0 x 10^{-3}. b) Molefractions included to show how the composition of the mixture of complexes changes over the course of the titration.

Discussion:

Based on the F cov_{fit} (6.2) and inspection of the binding isotherms, the 2:1 model best describes this data. Therefore concluding that the derived association constants from the 2:1 model should be considered.

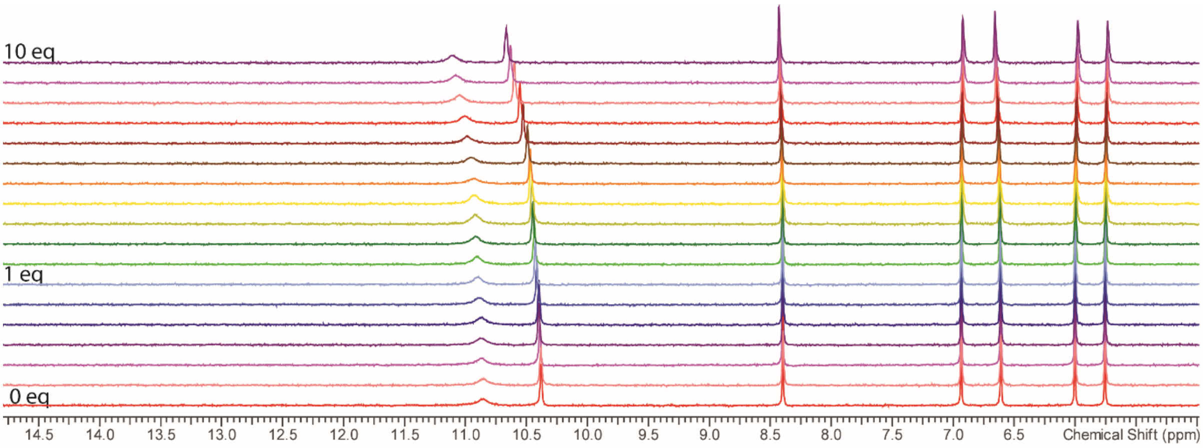

Figure S81. Stack plot of NMR spectra for compound **2** + TBACl

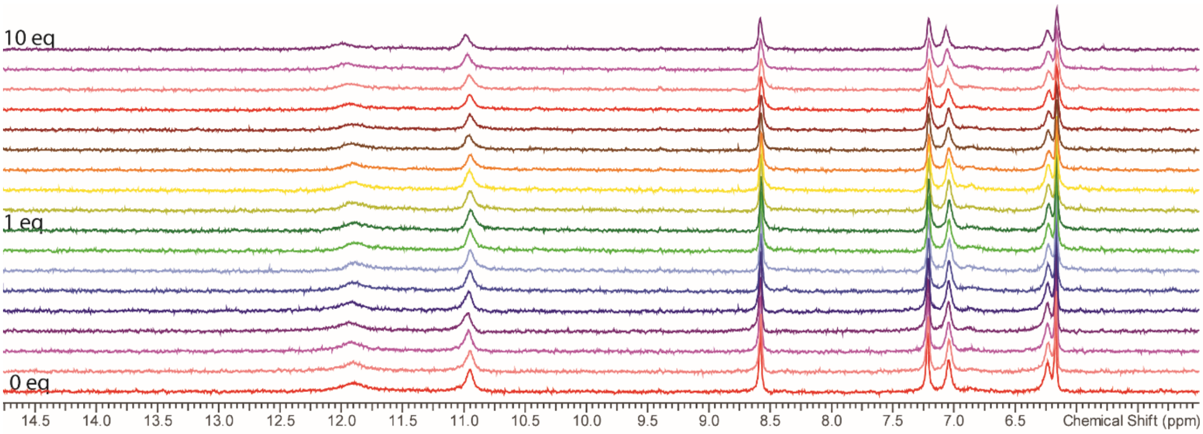

Figure S82. Stack plot of NMR spectra for compound **2** + HPF_6 + $TBANO_3$

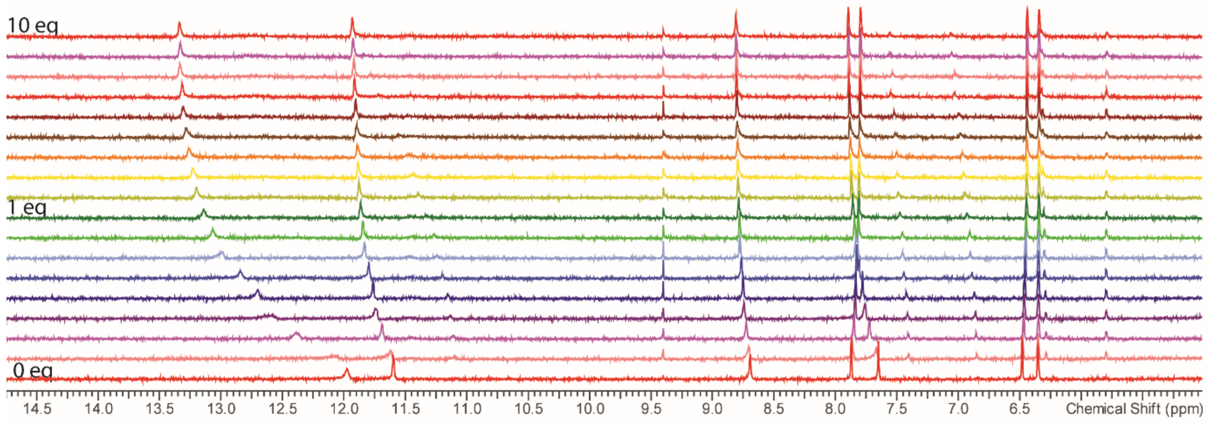

Figure S83. Stack plot of NMR spectra for compound **3** + HPF_6 + TBACl

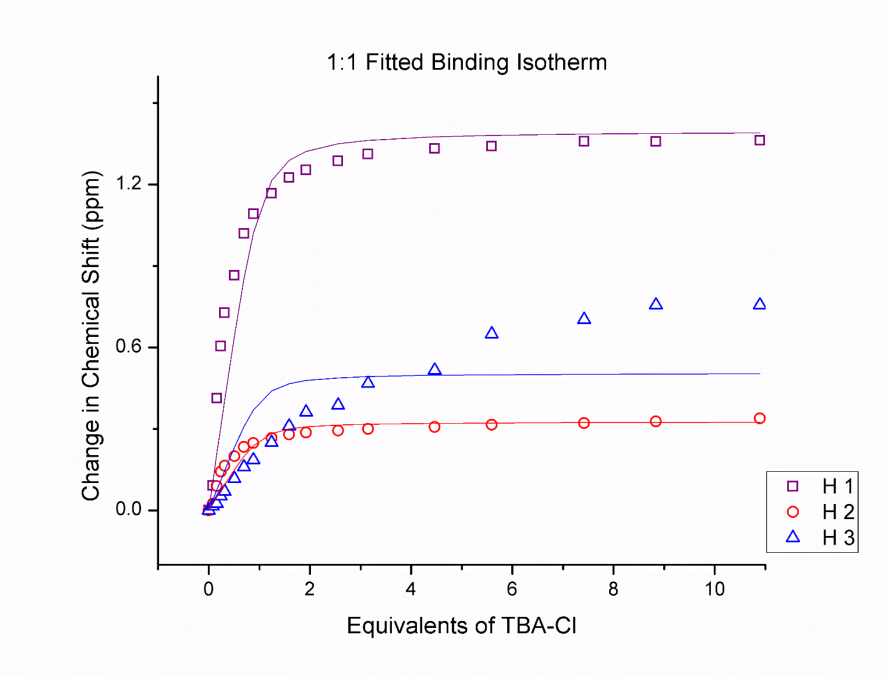

Figure S84. Fitted binding isotherm of compound [**3** + HPF_6] with TBA-Cl in DMSO-d_6 showing the change in chemical shift of the NH protons fitted to the 1:1 binding model. K_a: 3440 M^{-1}; covariance of fit: 6.4 x 10^{-2}.

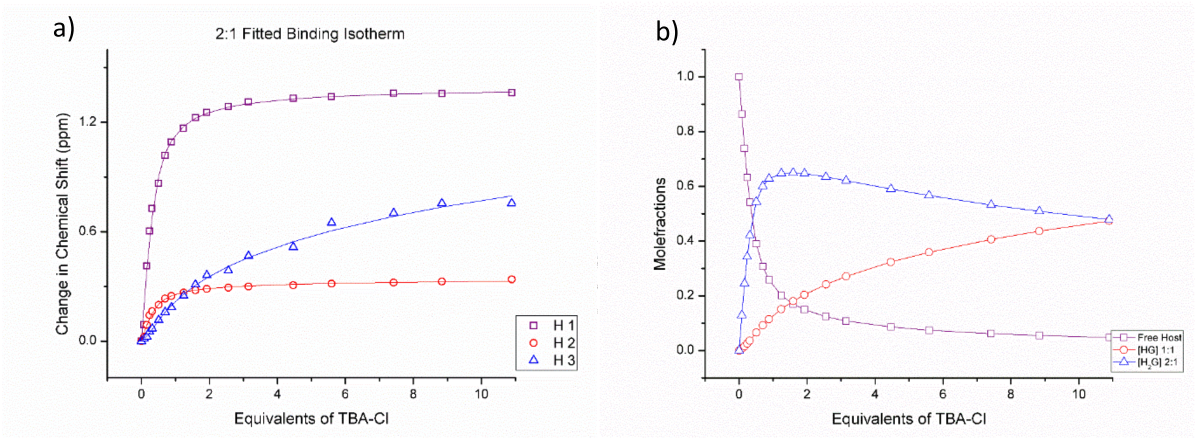

Figure S85. a) Fitted binding isotherm of compound [**3** + HPF_6] with TBA-Cl in DMSO-d_6 showing the change in chemical shift of two NH protons (H 1 and H 3) and one CH proton (H 2) fitted to the 2:1 full cooperative binding model. K_{11}: 190 M^{-1}; K_{21}: 2070 M^{-1}; covariance of fit: 2.6 x 10^{-3}. K_{21} corresponds to [H_2G] which in this case is larger than K_{11} showing that the 2:1 complex is favoured. b) Molefractions included to show how the composition of the mixture of complexes changes over the course of the titration, here about half the sample exists and [HG] and half exists as [H_2G].

Discussion:

Based on the F cov_{fit} (24.6) and inspection of the binding isotherms, the 2:1 model best describes this data. Therefore concluding that the derived association constants from the 2:1 model should be considered.

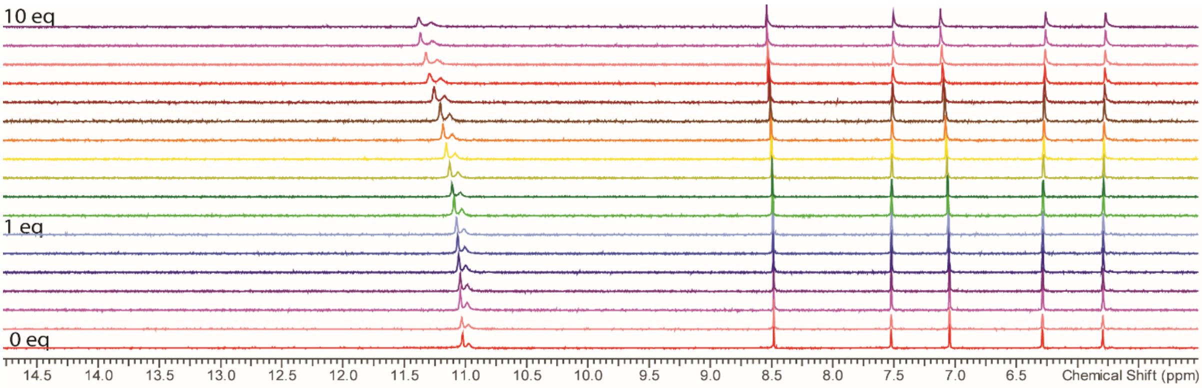

Figure S86. Stack plot of NMR spectra for compound **3** + TBACl

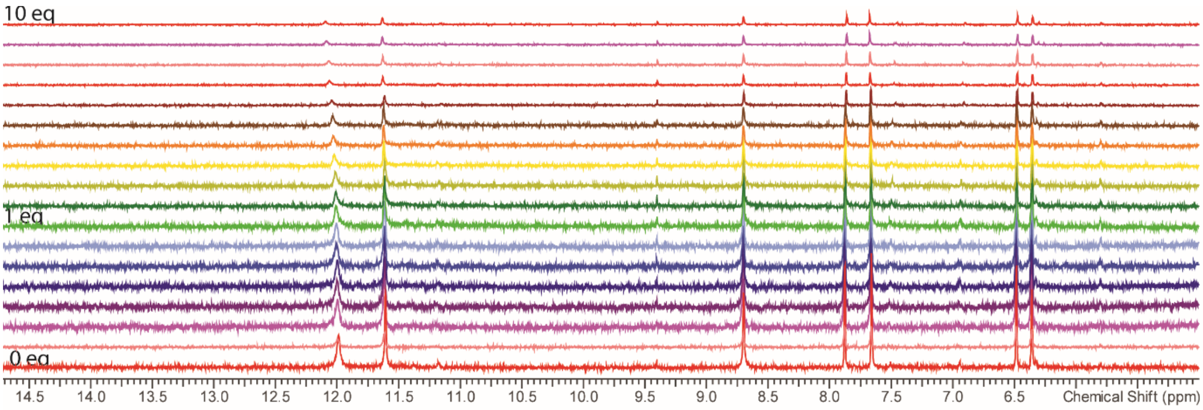

Figure S87. Stack plot of NMR spectra for compound **3** + HPF_6 + $TBANO_3$

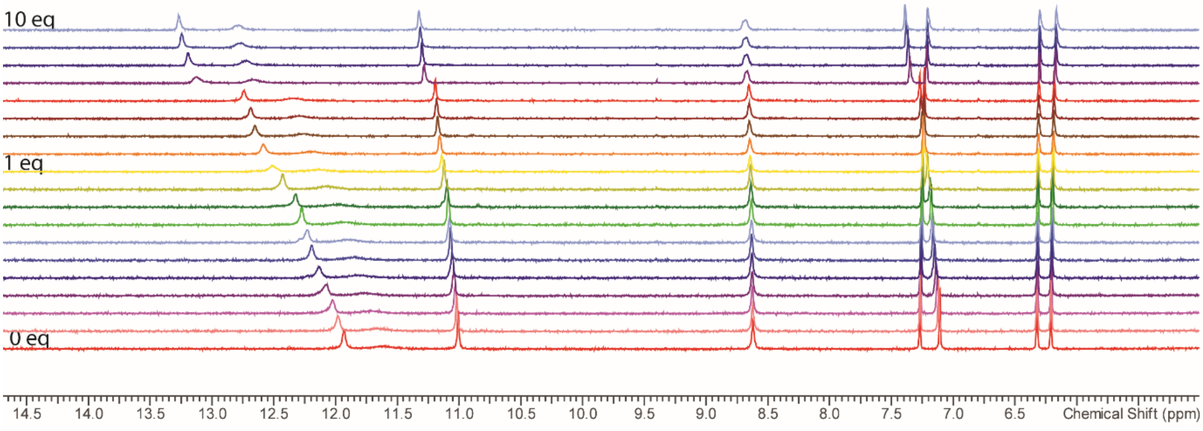

Figure S88. Stack plot of NMR spectra for compound **4** + HPF_6 + TBACl

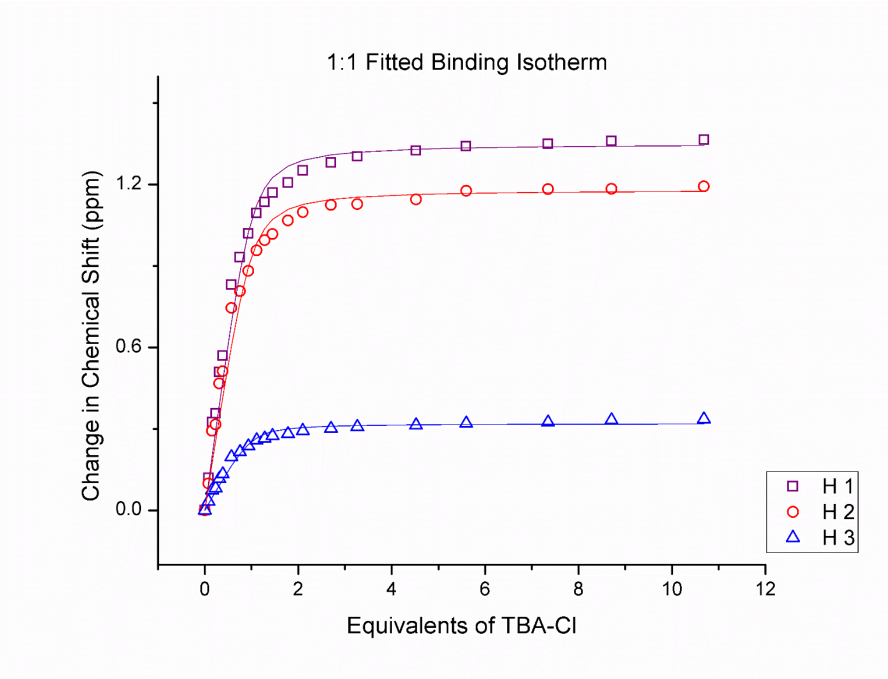

Figure S89. Fitted binding isotherm of compound [**4** + HPF_6] with TBA-Cl in DMSO-d_6 showing the change in chemical shift of the NH protons fitted to the 1:1 binding model. K_a: 3440 M^{-1}; covariance of fit: 1.1 x 10^{-2}.

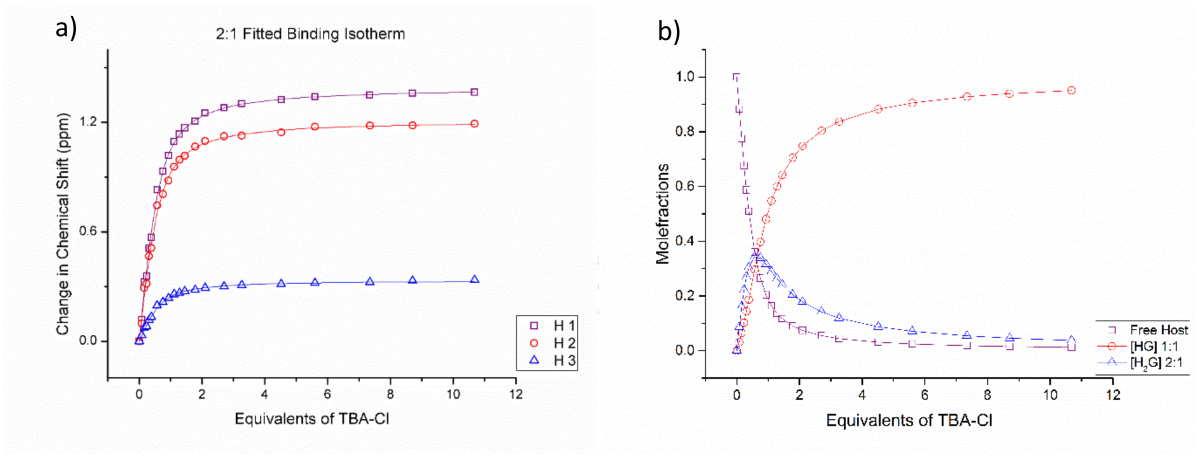

Figure S90. a) Fitted binding isotherm of compound [**4** + HPF_6] with TBA-Cl in DMSO-d_6 showing the change in chemical shift of the NH protons fitted to the 2:1 full cooperative binding model. K_{11}: 1530 M^{-1}; K_{21}: 306 M^{-1}; covariance of fit: 1.2 x 10^{-3} b) Molefractions included to show how the composition of the mixture of complexes changes over the course of the titration.

Discussion:

Based on the F cov_{fit} (9.16) and inspection of the binding isotherms, the 2:1 model best describes this data. Therefore concluding that the derived association constants from the 2:1 model should be considered.

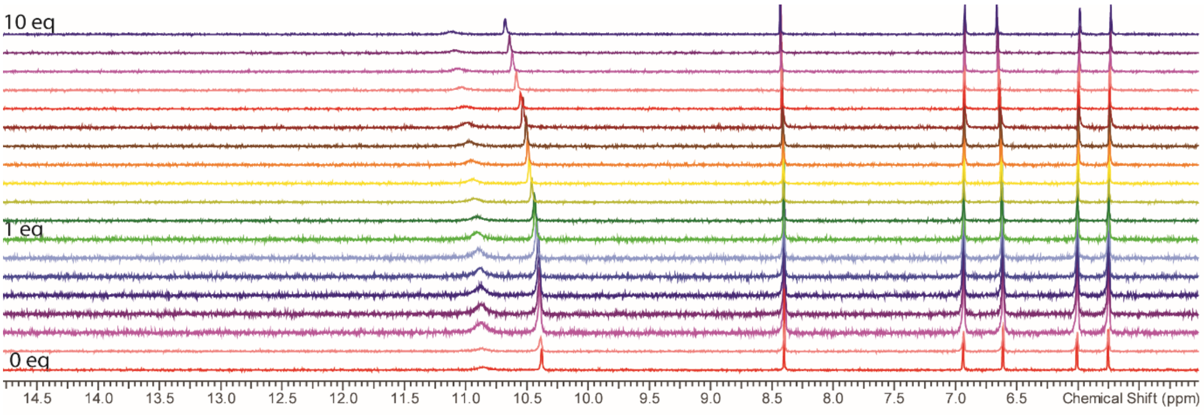

Figure S91. Stack plot of NMR spectra for compound **4** + TBACl

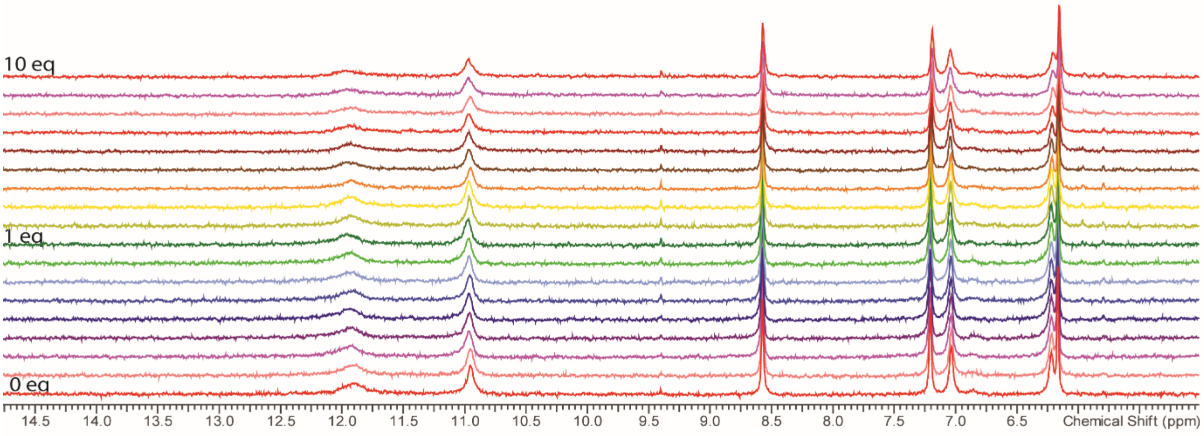

Figure S92. Stack plot of NMR spectra for compound **4** + HPF_6 + $TBANO_3$

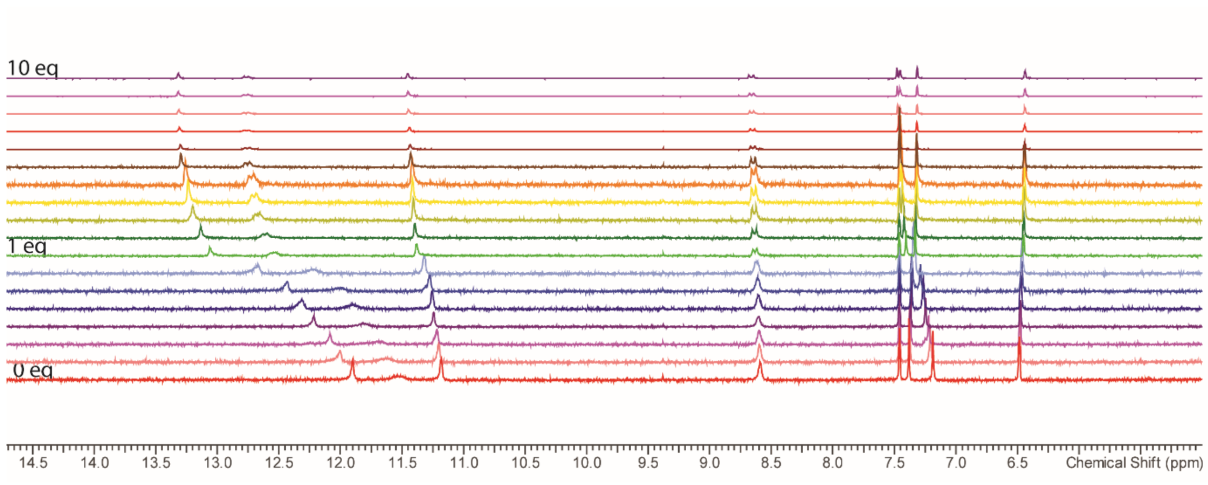

Figure S93. Stack plot of NMR spectra for compound **5** + HPF_6 + TBACl

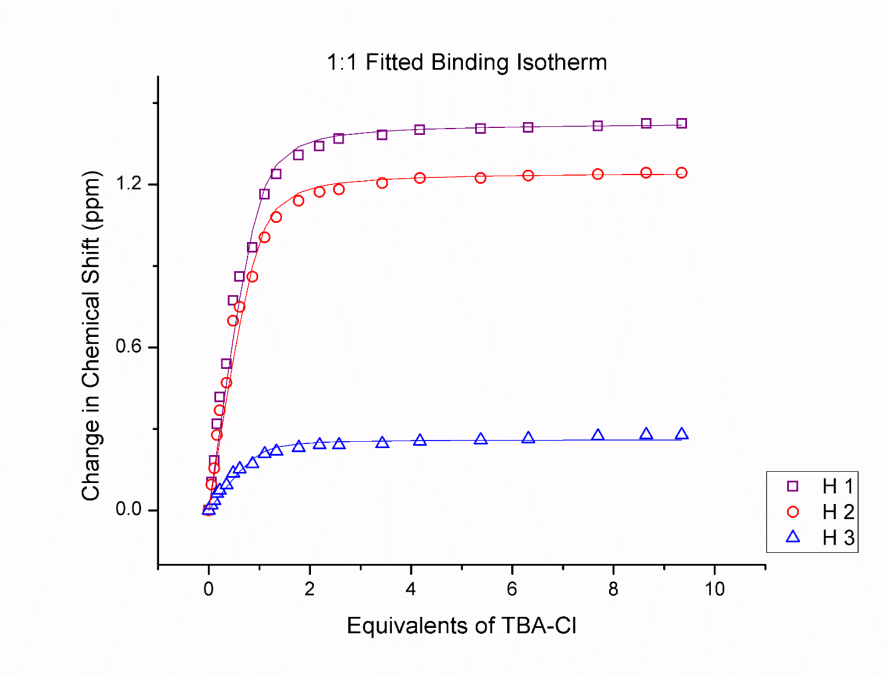

Figure S94. Fitted binding isotherm of compound [**5** + HPF_6] with TBA-Cl in DMSO-d_6 showing the change in chemical shift of the NH protons fitted to the 1:1 binding model. K_a: 2610 M^{-1}; covariance of fit: 5.2 x 10^{-3}.

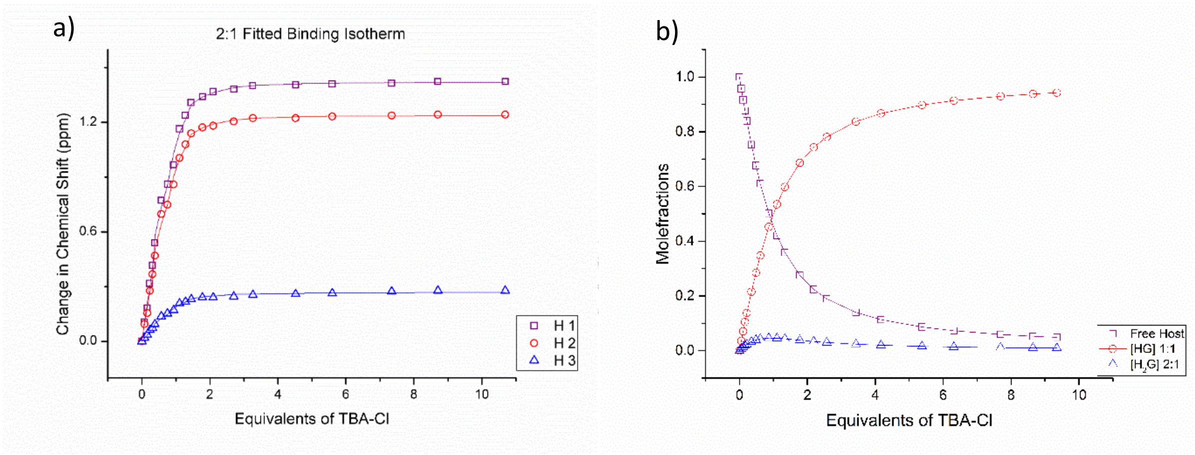

Figure S95. a) Fitted binding isotherm of compound [**5** + HPF_6] with TBA-Cl in DMSO-d_6 showing the change in chemical shift of the NH protons fitted to the 2:1 full cooperative binding model. K_{11}: 331 M^{-1}; K_{21}: 14 M^{-1}; covariance of fit: 8.6 x 10^{-4}. b) Molefractions included to show how the composition of the mixture of complexes changes over the course of the titration.

Discussion:

Based on the F cov_{fit} (6.0) and inspection of the binding isotherms, the 2:1 model best describes this data. Therefore concluding that the derived association constants from the 2:1 model should be considered.

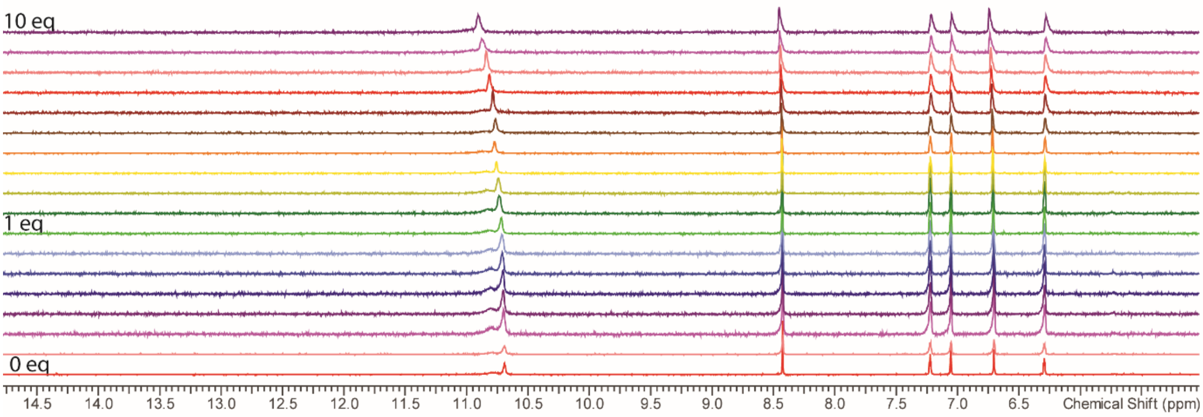

Figure S96. Stack plot of NMR spectra for compound **5** + TBACl

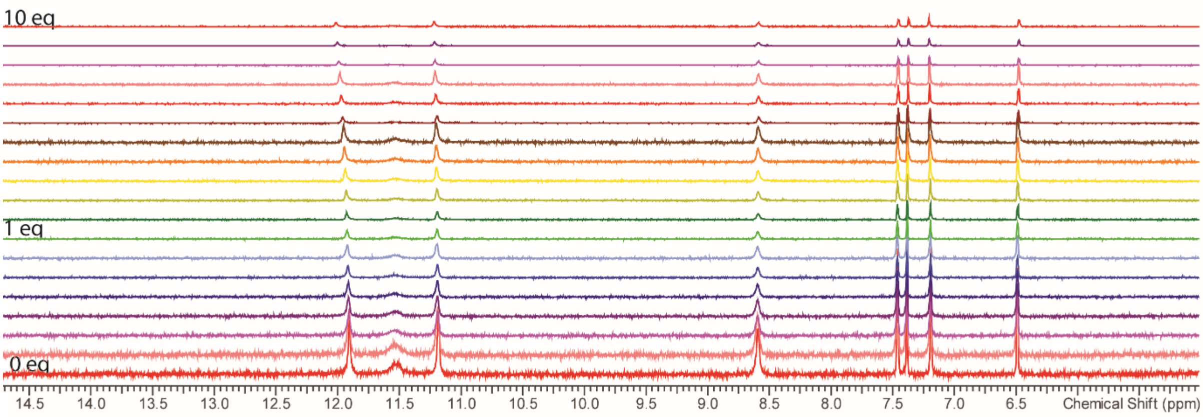

Figure S97. Stack plot of NMR spectra for compound **5** + HPF_6 + $TBANO_3$

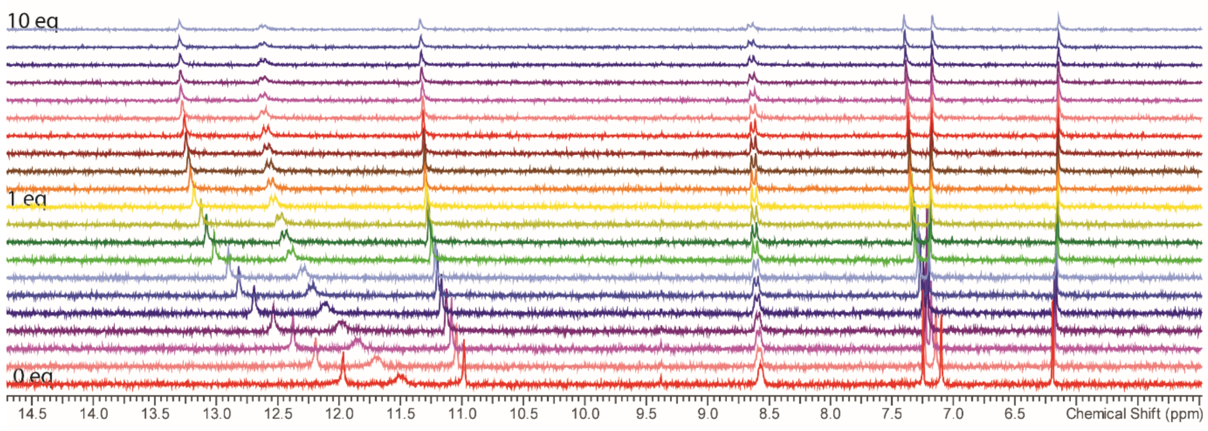

Figure S98. Stack plot of NMR spectra for compound **6** + HPF_6 + TBACl

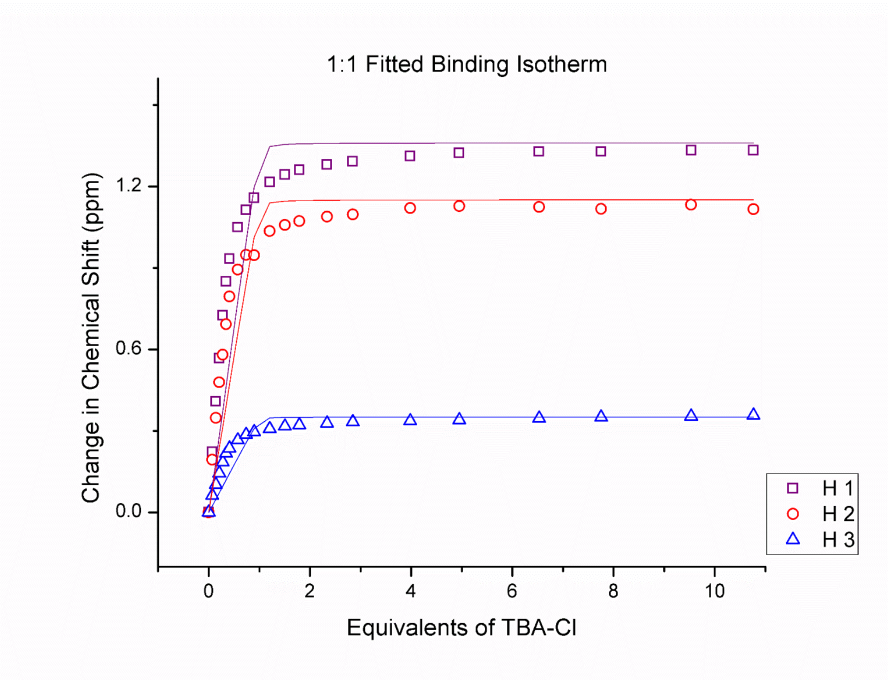

Figure S99. Fitted binding isotherm of compound [**6** + HPF_6] with TBA-Cl in DMSO-d_6 showing the change in chemical shift of the NH protons fitted to the 1:1 binding model. K_a: 78300 M^{-1}; covariance of fit: 8.8 x 10^{-2}.

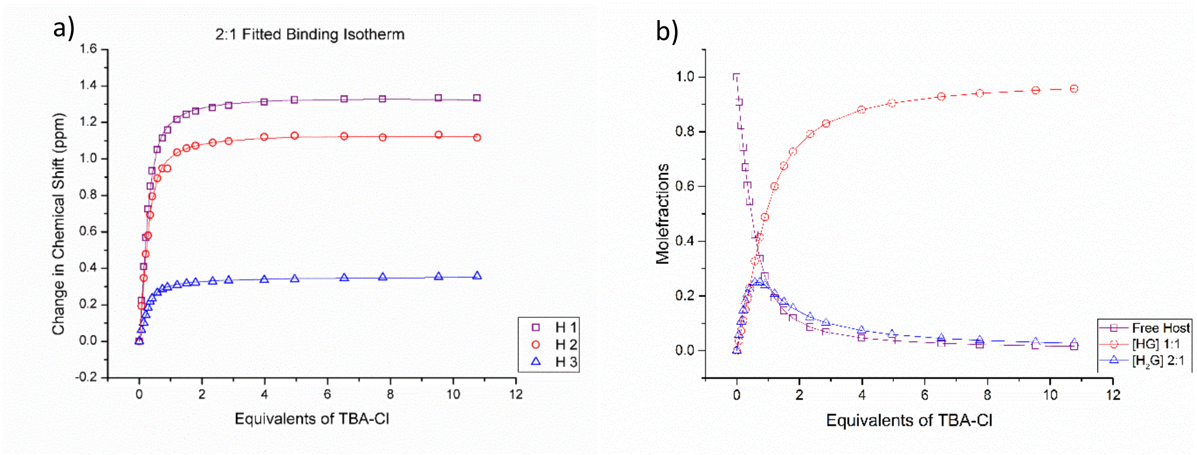

Figure S100. a) Fitted binding isotherm of compound [**6** + HPF_6] with TBA-Cl in DMSO-d_6 showing the change in chemical shift of the NH protons fitted to the 2:1 additive binding model. K_{11}: 1090 M^{-1}; K_{21}: 158 M^{-1}; covariance of fit: 7.5 x 10^{-3}. b) Molefractions included to show how the composition of the mixture of complexes changes over the course of the titration.

Discussion:

Based on the F cov_{fit} (11.7) and inspection of the binding isotherms, the 2:1 model best describes this data. Therefore concluding that the derived association constants from the 2:1 model should be considered.

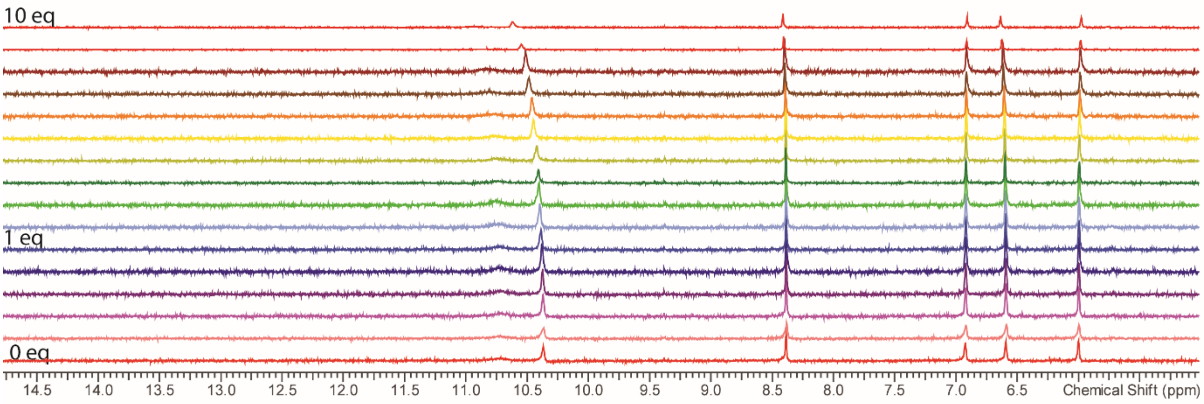

Figure S101. Stack plot of NMR spectra for compound **6** + TBACl

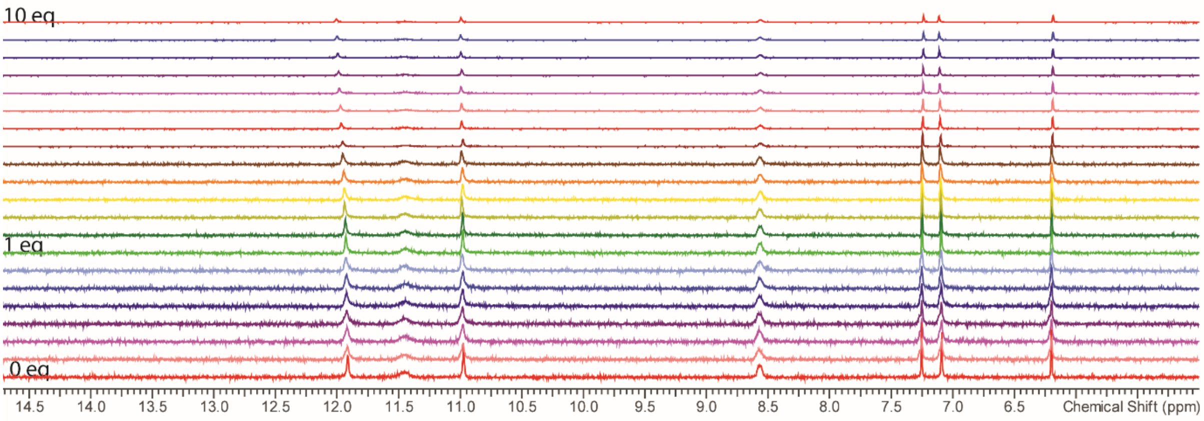

Figure S102. Stack plot of NMR spectra for compound **6** + HPF_6 + $TBANO_3$

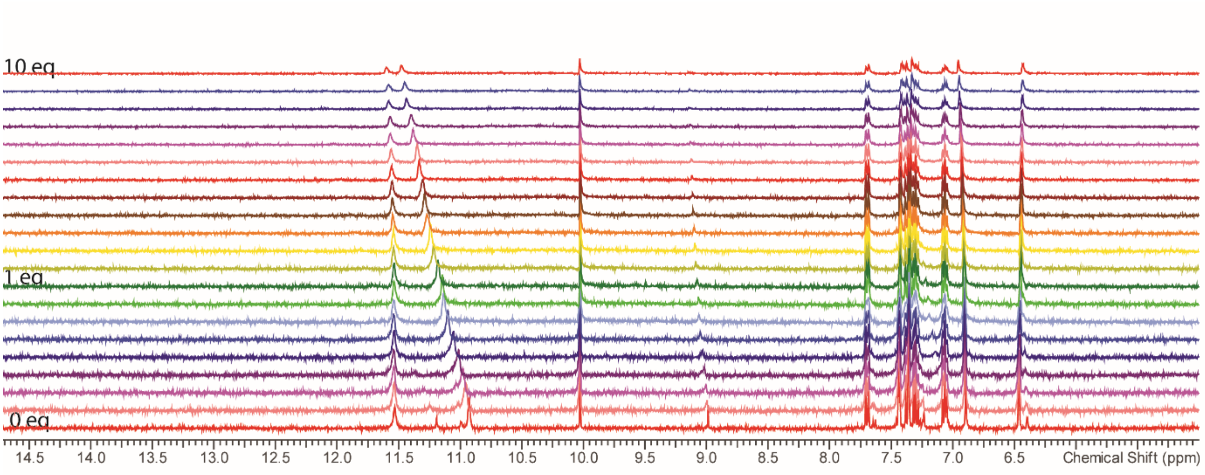

Figure S103. Stack plot of NMR spectra for compound **7** + HPF_6 + TBACl

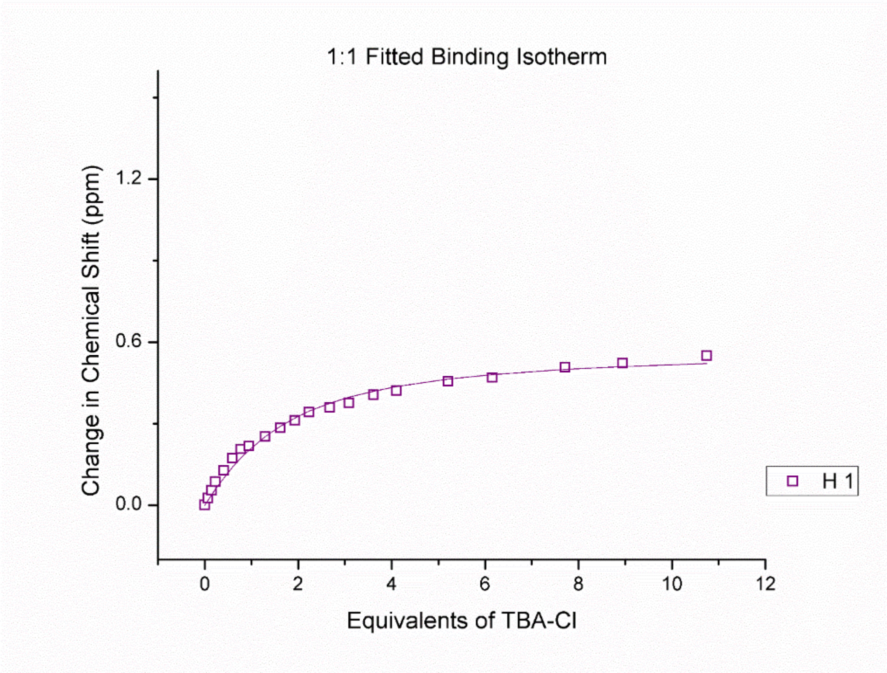

Figure S104. Fitted binding isotherm of compound [**7** + HPF_6] with TBA-Cl in DMSO-d_6 showing the change in chemical shift of the NH proton fitted to the 1:1 binding model. K_a: 157 M^{-1}; covariance of fit: 1.0 x 10^{-2}.

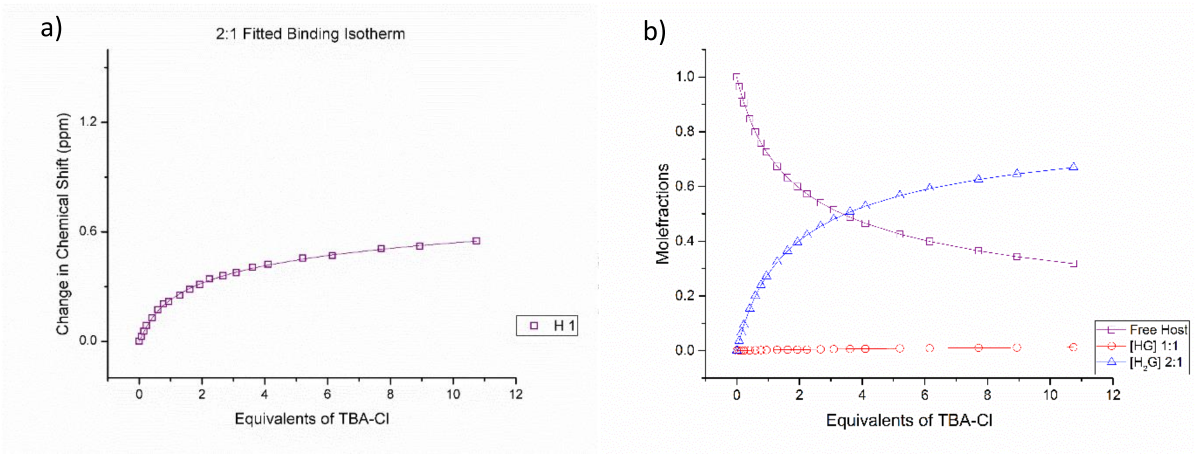

Figure S105. a) Fitted binding isotherm of compound [**7** + HPF_6] with TBA-Cl in DMSO-d_6 showing the change in chemical shift of the NH proton fitted to the 2:1 additive binding model. K_{11}: 72 M^{-1}; K_{21}: 507 M^{-1}; covariance of fit: 8.2 x 10^{-4}. b) Molefractions included to show how the composition of the mixture of complexes changes over the course of the titration.

Discussion:

Based on the F cov_{fit} (12.2) and inspection of the binding isotherms, the 2:1 model best describes this data. However, full protonation does not occur (~ 20 % unprotonated) upon addition of 1 equivalent of HPF_6 acid, therefore the derived association constants from the 2:1 model should be treated with caution.

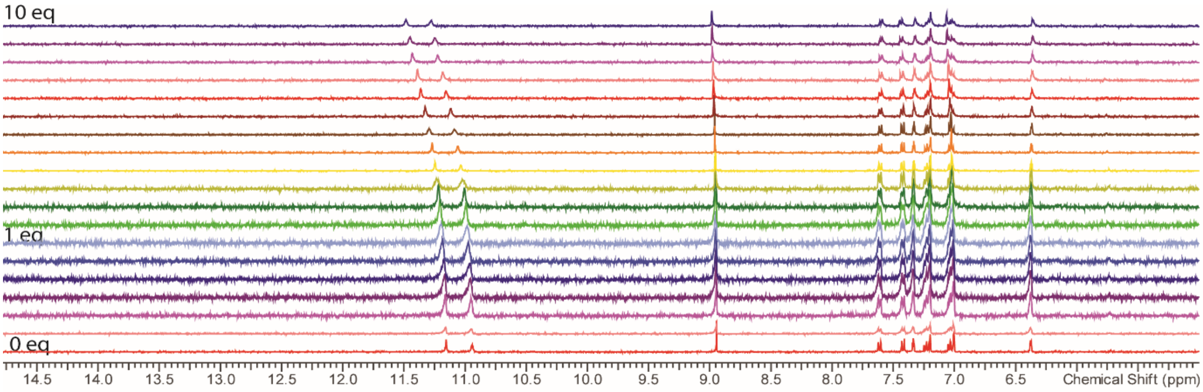

Figure S106. Stack plot of NMR spectra for compound **7** + TBACl

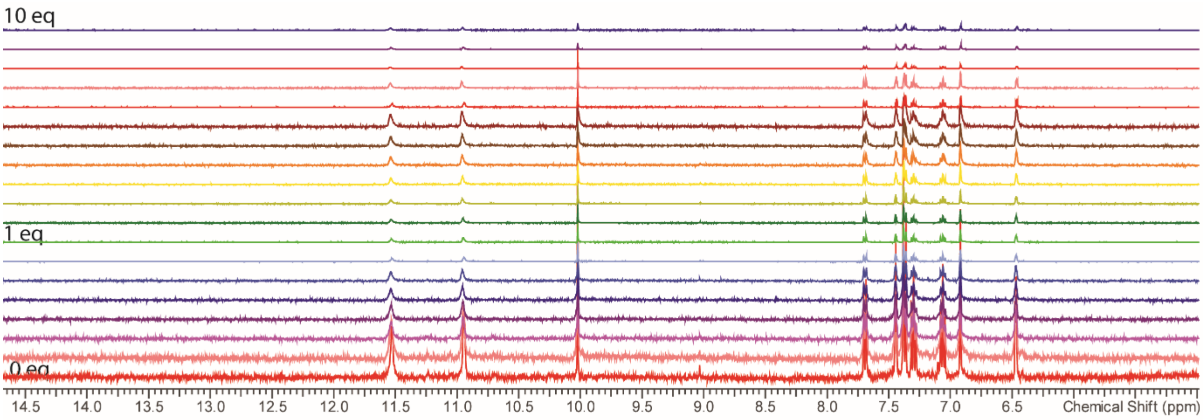

Figure S107. Stack plot of NMR spectra for compound **7** + HPF_6 + $TBANO_3$

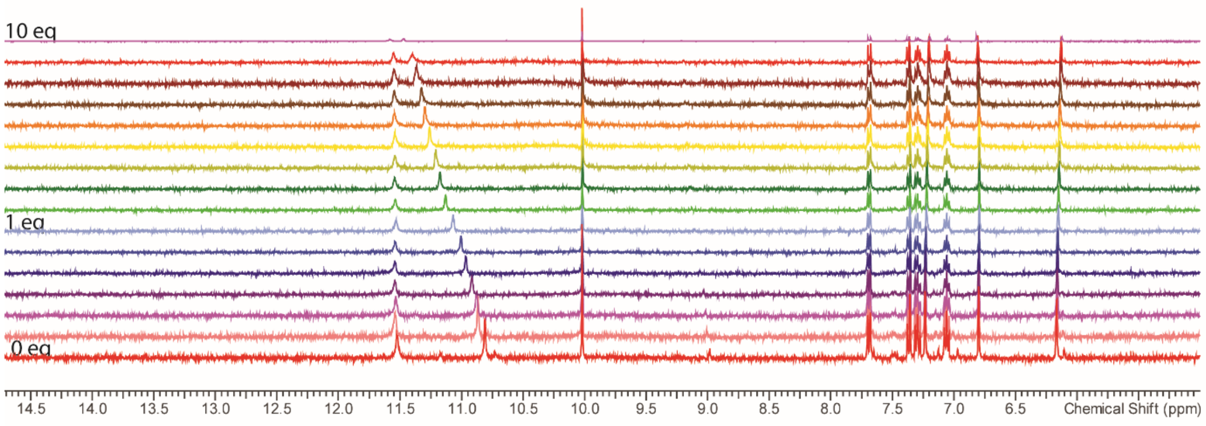

Figure S108. Stack plot of NMR spectra for compound **8** + HPF_6 + TBACl

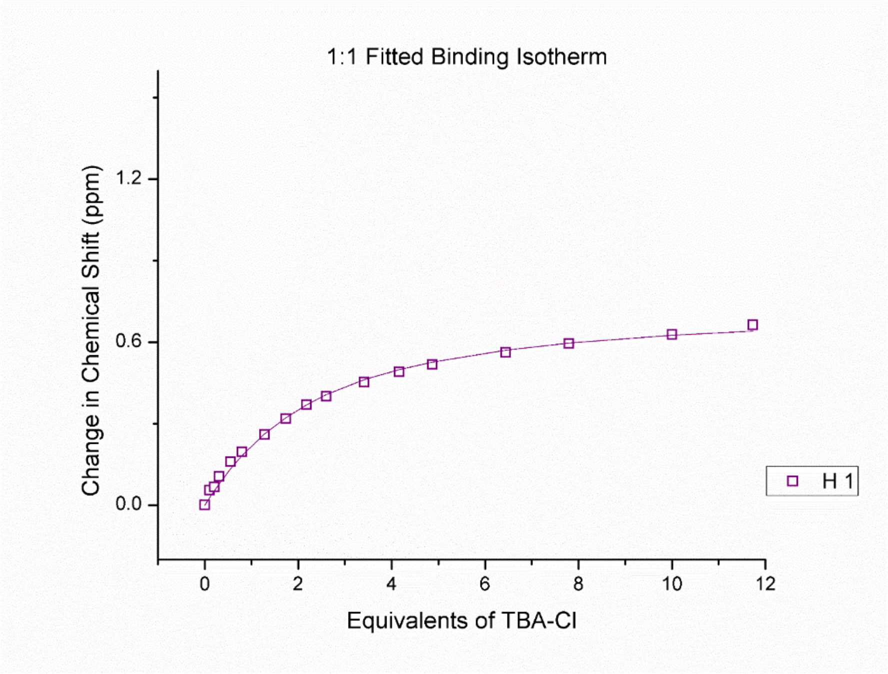

Figure S109. Fitted binding isotherm of compound [**8** + HPF_6] with TBA-Cl in DMSO-d_6 showing the change in chemical shift of the NH proton fitted to the 1:1 binding model. K_a: 142 M^{-1}; covariance of fit: 4.0 x 10^{-3}.

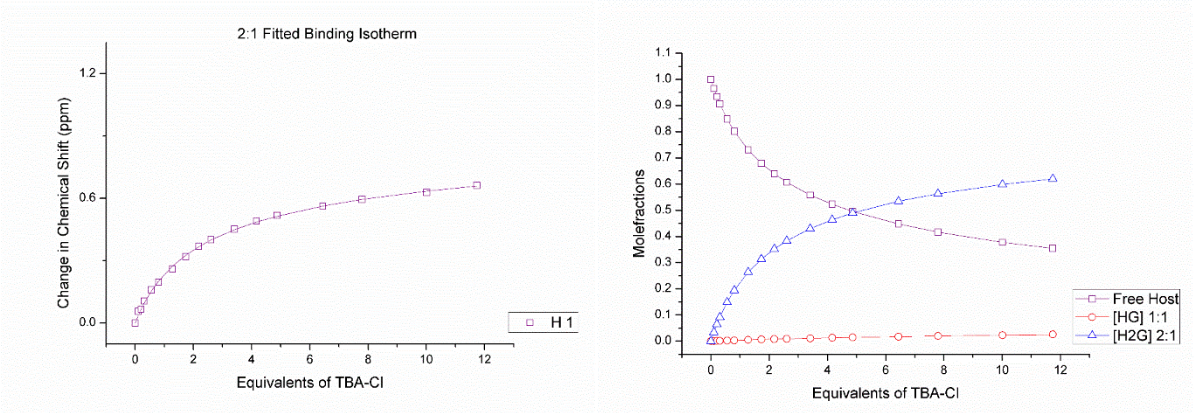

Figure S110. a) Fitted binding isotherm of compound [**8** + HPF_6] with TBA-Cl in DMSO-d_6 showing the change in chemical shift of the NH protons fitted to the 2:1 additive binding model. K_{11}: 184 M^{-1}; K_{21}: 193 M^{-1}; covariance of fit: 1.2 x 10^{-3}. b) Molefractions included to show how the composition of the mixture of complexes changes over the course of the titration, and it is evident that there is no 2:1 complex forming.

Discussion:

Based on the F cov_{fit} (3.3) and inspection of the binding isotherms, the 2:1 model best describes this data. However, full protonation does not occur (~ 10 % unprotonated) upon addition of 1 equivalent of HPF_6 acid, therefore the derived association constants from the 2:1 model should be treated with caution.

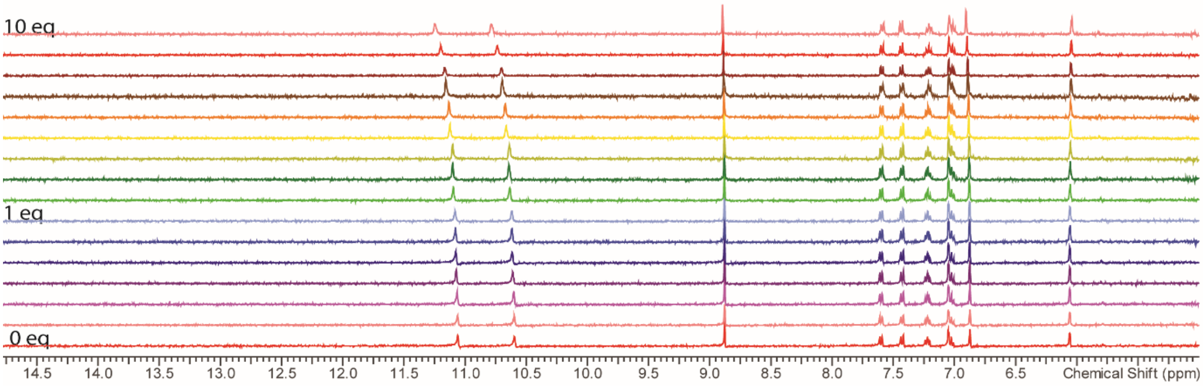

Figure S111. Stack plot of NMR spectra for compound **8** + TBACl

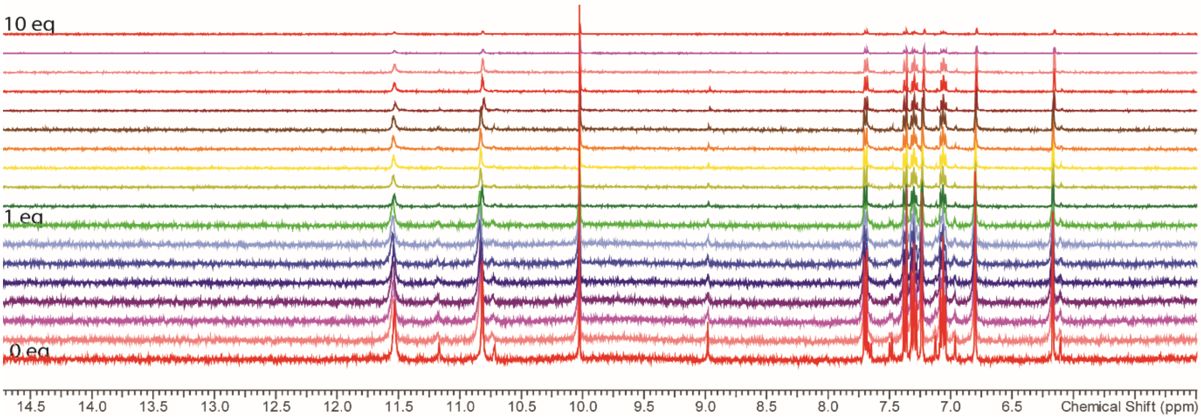

Figure S112. Stack plot of NMR spectra for compound **8** + HPF_6 + $TBANO_3$

S9. Dilution Studies

Dilution studies were performed for compounds **2**, **6** and **8** to give an insight into whether the solid state crystal structure of dimers represented the solution composition, and if this affected the anion binding and transport properties. A stock solution (40 mM) of the receptor was prepared in MeCN-d_3 and this was diluted to give 12-15 samples of different concentrations. A ^{1}H NMR spectrum was recorded for each concentration and the change in chemical shift was noted for any protons that shifted either upfield or downfield. The supramolecular.org web applet[10] was used to fit the collected dilution data to give the equilibrium of aggregation (K_e) and the dimerization constant (K_d).

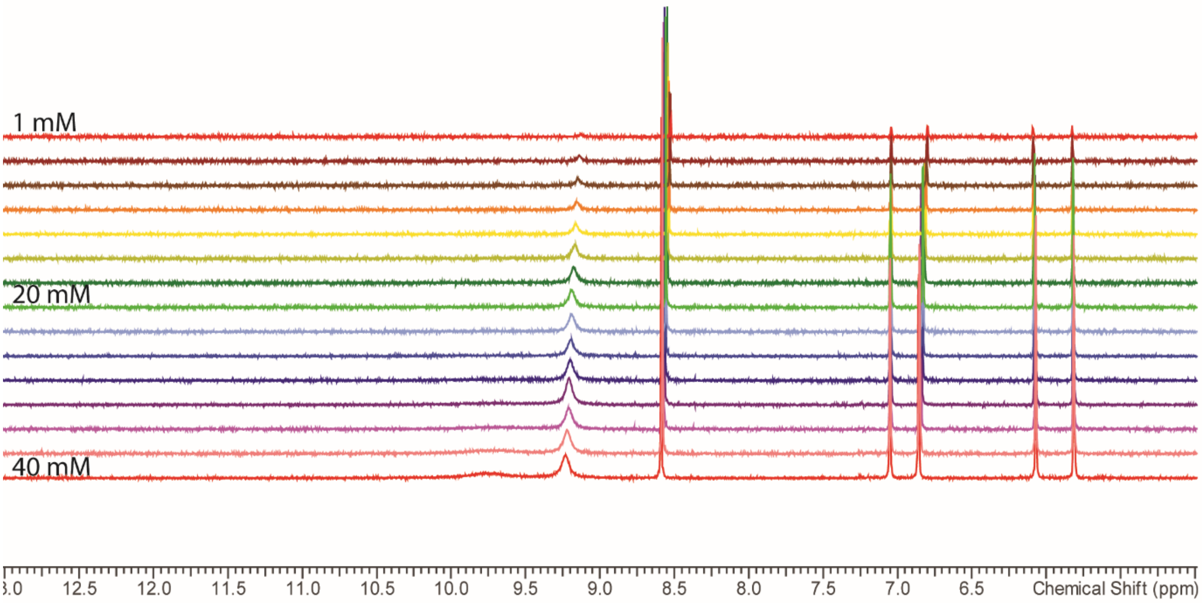

Figure S113. Stack plot of NMR spectra for compound **2** in MeCN-d_3 at various concentrations

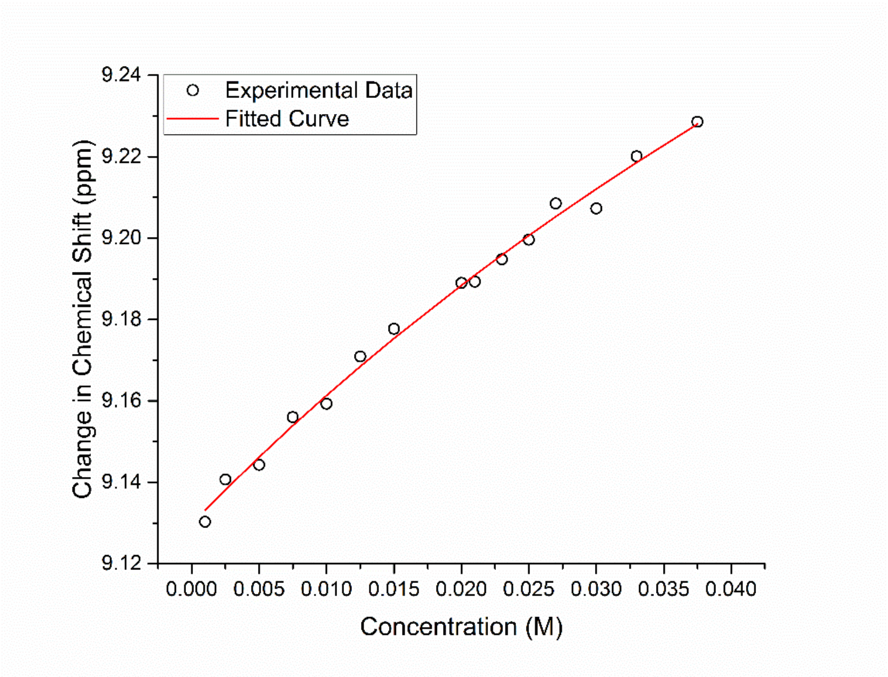

Figure S114. Fitted dilution curve of compound **2** in MeCN-d_3 showing the change in chemical shift of the NH proton fitted using the Nelder-Mead method. K_e: 4.39 M^{-1} and K_d: 2.2 M^{-1} both show weak association.

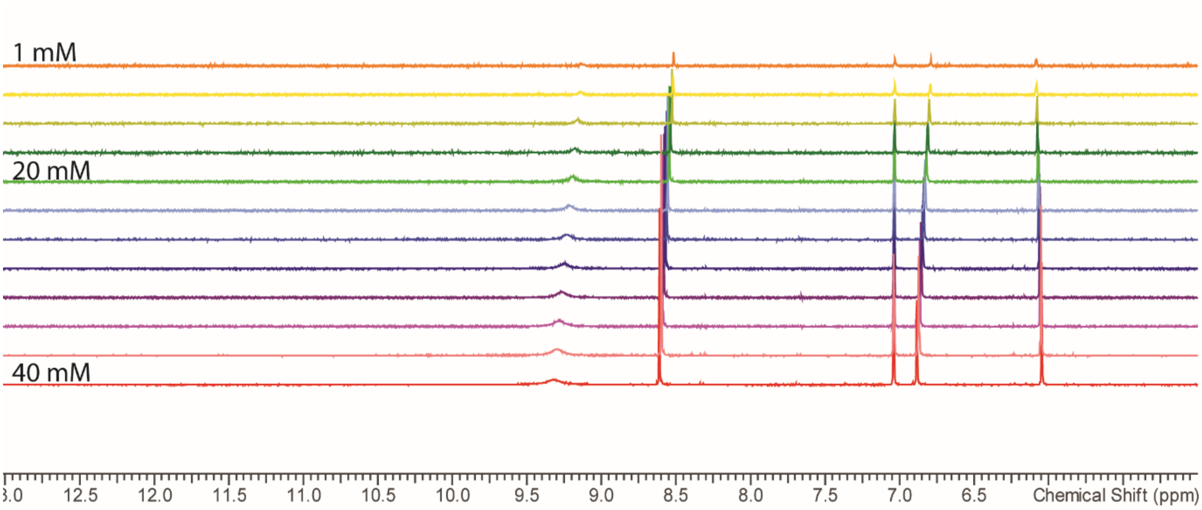

Figure S115. Stack plot of NMR spectra for compound **6** in MeCN-d_3 at various concentrations

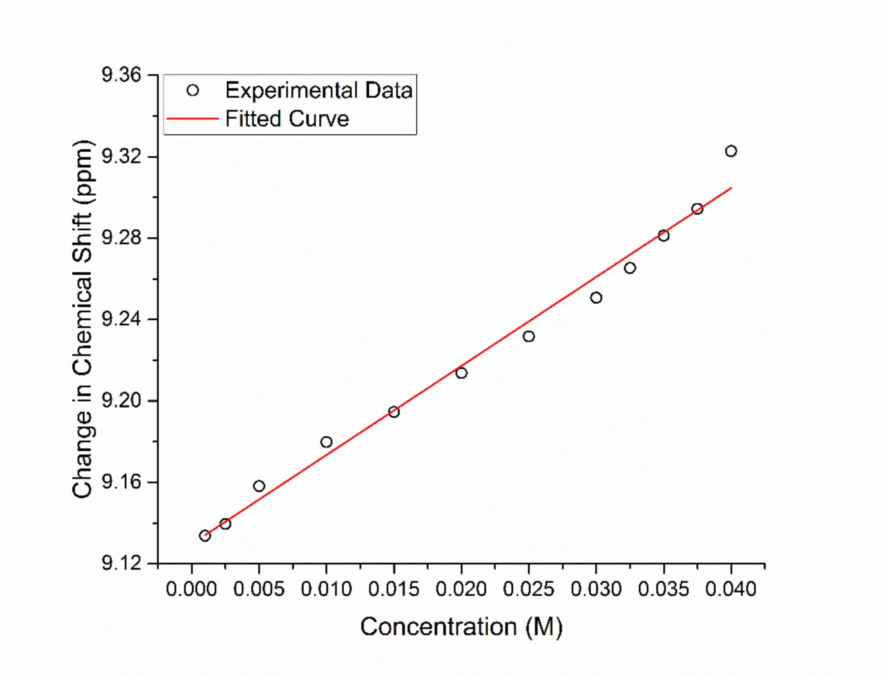

Figure S116. Fitted dilution curve of compound **6** in MeCN-d_3 showing the change in chemical shift of the NH proton fitted using the Nelder-Mead method. K_e: 0.000710 M^{-1} and K_d: 0.000355 M^{-1} both show very weak association.

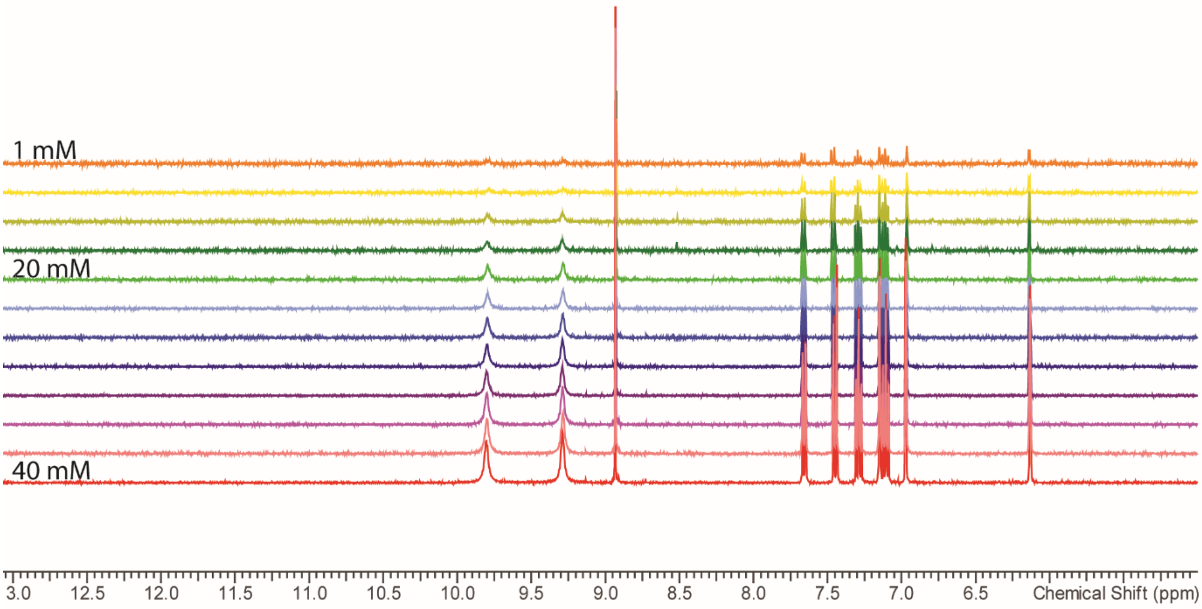

Figure S117. Stack plot of NMR spectra for compound **8** in MeCN-d_3 at various concentrations

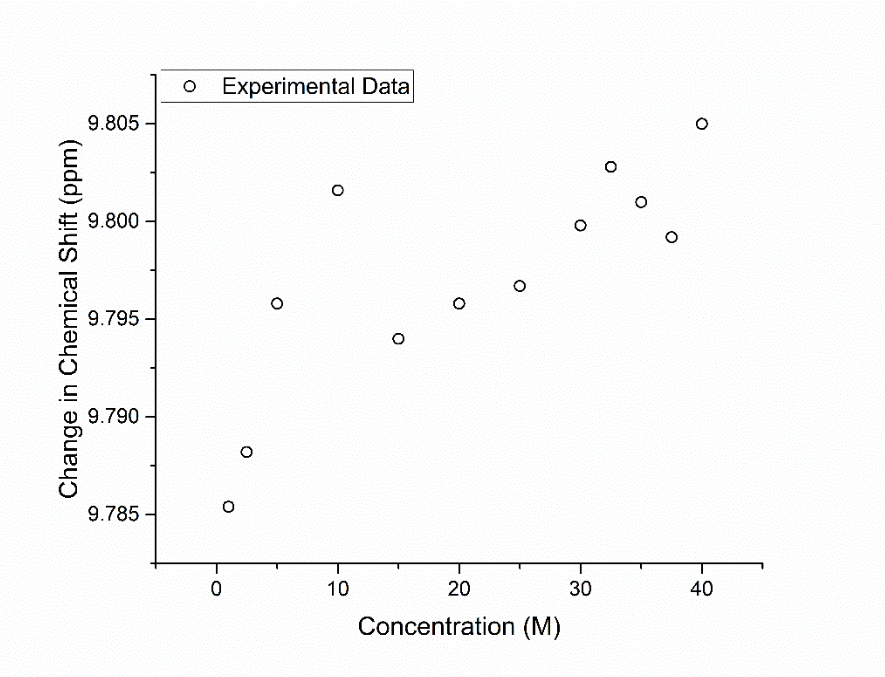

Figure S118. Dilution data for compound **8** in MeCN-d_3 showing the change in chemical shift of the NH proton, could not be fitted using the Nelder-Mead method due to negligible association.

The dilution studies performed showed weak to no association of the compounds in solution meaning that the compounds are very unlikely to exist as dimers in solution like they do in solid state. Therefore this does not affect the anion binding or transport.

S10. Anion Transport Studies

S10.1. General Procedure for the ISE Assay

Unilamellar vesicles were prepared using previously reported literature procedures[7-9]. A lipid film of POPC (1-palmitoyl-2-oleoyl-sn-glycero-3-phosphocholine) or POPC:cholesterol (7:3 molar ratio) was prepared from a chloroform solution under reduced pressure and then dried under vacuum for 4 or more hours. The lipid film was rehydrated by vortexing with an internal solution of buffered metal chloride salt (usually 489 mM MCl). The lipid suspension was then subjected to 9 freeze-thaw cycles and left to rest at room temperature for 30 minutes. After this, the suspension was extruded 25 times through a 200 nm polycarbonate membrane resulting in unilamellar vesicles of a mean diameter of 200 nm. The large unilamellar vesicles were dialysed in the corresponding external solution for a minimum of 2 hours to remove any unencapsulated metal salts. The lipid solution obtained after dialysis was diluted to a standard volume (usually 10 mL) with the external buffered solution to obtain a lipid stock of known concentration.

Internal and external solutions vary for each experiment and are detailed for each. Experiments conducted at different pHs required different buffers (citrate buffer for pH 4.5; phosphate buffer for pH 6.5 and pH 7.2; and ethanolamine buffer for pH 10) however, a constant buffer concentration (5 mM) and total ionic strength (500 mM) was maintained.

The lipid stock was diluted with the external buffer solution to a standard volume (5 mL), to afford a solution with a lipid concentration of 1 mM. The compounds were added as a DMSO solution (10 µL) at the start of the experiment and the resulting chloride efflux was monitored using a chloride selective electrode over 5 minutes. After this time, detergent (50 µL of Triton X-100 (11 w%) in H_2O:DMSO (7:1 v/v)) was added to lyse the vesicles and after 7 minutes a final chloride efflux reading was taken. This value represented 100 % chloride efflux and was used to calibrate the other readings.

S10.2. Cl^-/NO_3^- ISE Assay - pH Tests

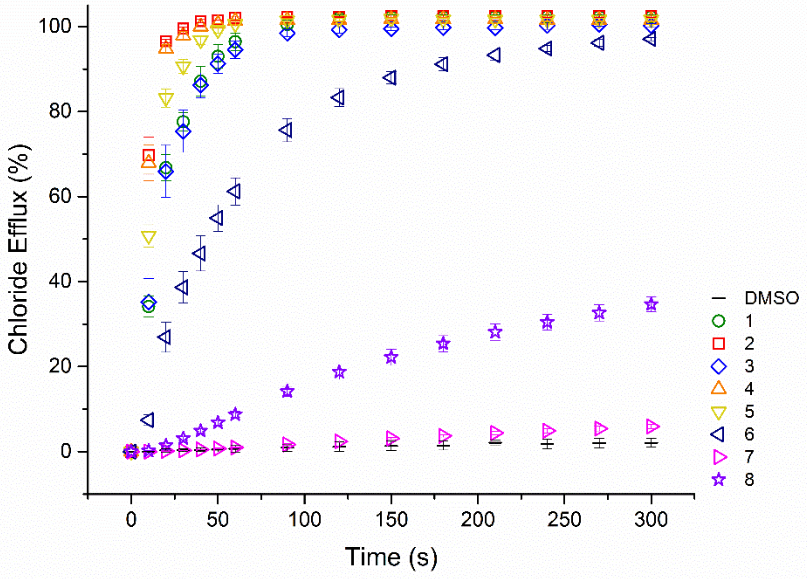

Figure S119. Chloride efflux facilitated by compounds **1d** and **2**-**8** at 0.1 mol% loading (with respect to lipid) from POPC vesicles loaded with NaCl (489 mM) and suspended in $NaNO_3$ (489 mM), buffered to pH 4.5 with sodium citrate salts (5 mM). DMSO was used as a control. Each data point is the average of three repeated measurements with the error bars showing the standard deviation.

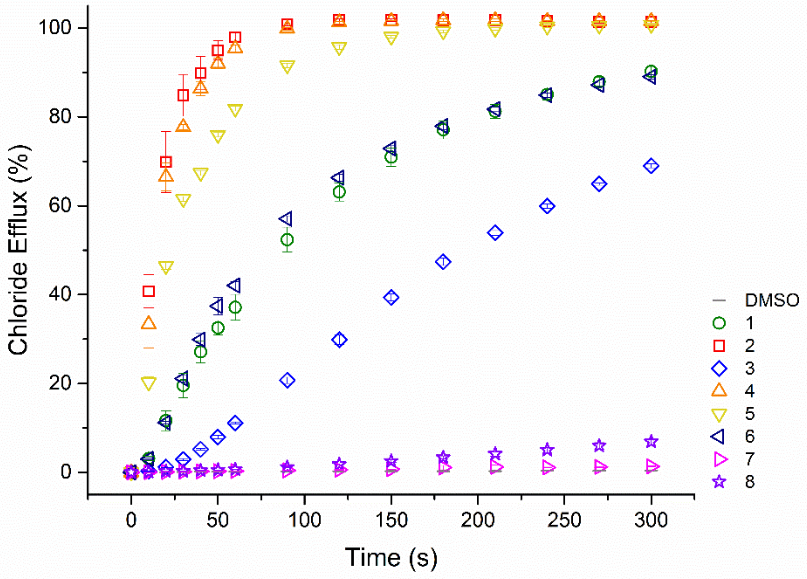

Figure S120. Chloride efflux facilitated by compounds **1d** and **2**-**8** at 0.1 mol% loading (with respect to lipid) from POPC vesicles loaded with NaCl (489 mM) and suspended in $NaNO_3$ (489 mM), buffered to pH 6.5 with sodium phosphate salts (5 mM). DMSO was used as a control. Each data point is the average of three repeated measurements with the error bars showing the standard deviation.

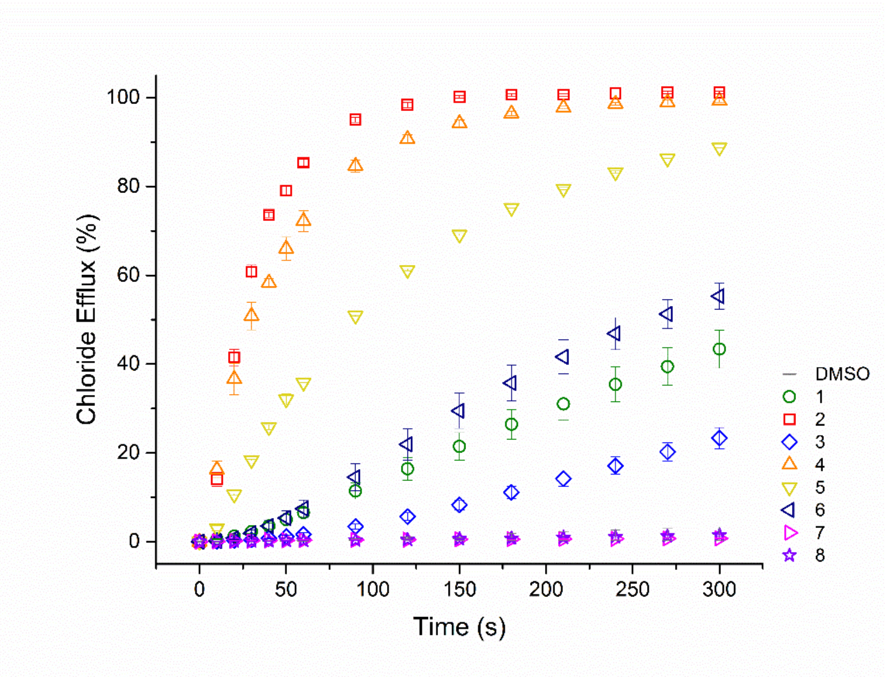

Figure S121. Chloride efflux facilitated by compounds **1d** and **2**-**8** at 0.1 mol% loading (with respect to lipid) from POPC vesicles loaded with NaCl (489 mM) and suspended in $NaNO_3$ (489 mM), buffered to pH 7.2 with sodium phosphate salts (5 mM). DMSO was used as a control. Each data point is the average of three repeated measurements with the error bars showing the standard deviation.

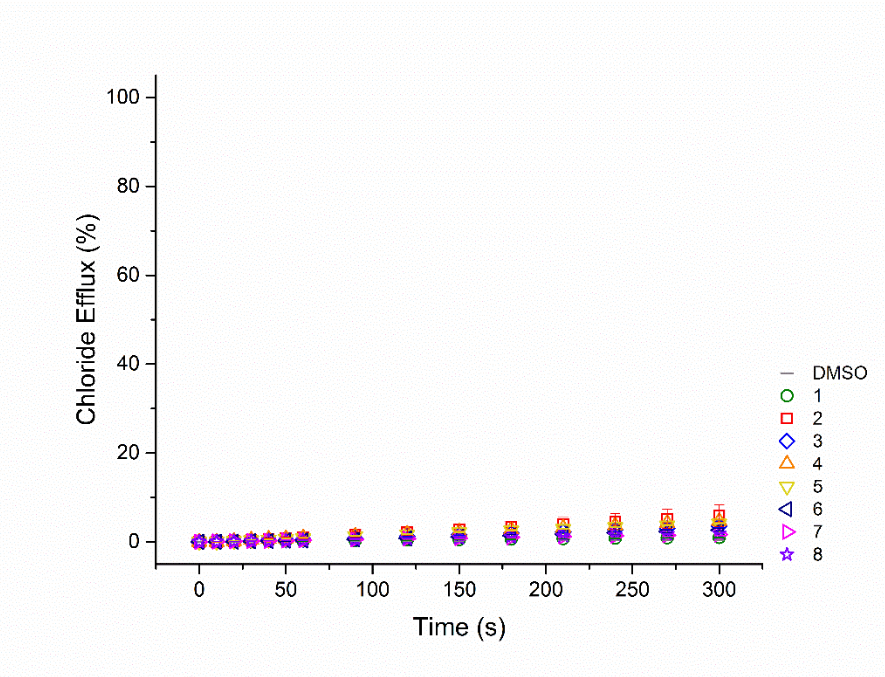

Figure S122. Chloride efflux facilitated by compounds **1d** and **2**-**8** at 0.1 mol% loading (with respect to lipid) from POPC vesicles loaded with NaCl (489 mM) and suspended in $NaNO_3$ (489 mM), buffered to pH 10 with ethanolamine (5 mM). DMSO was used as a control. Each data point is the average of three repeated measurements with the error bars showing the standard deviation.

At pH 4.5 compounds **1d** and **2**-**6** showed very high transport abilities reaching 100 % efflux within 5 minutes. The transport capability of **8** increased to 35 % chloride efflux but **7** remained inactive. Compared to pH 7.2, at pH 6.5 compounds **1d** and **2**-**6** show increased transport ability with **2**, **4** and **5** showing very similar, high transport capabilities and **1d**, **3** and **6** not transporting to as great an extent. **7** and **8** are both inactive at pH 6.5. At pH 10 there was no chloride efflux observed for any of the compounds.

S10.3. Cl^-/NO_3^- ISE Assay - Hill Analysis

The Cl^-/NO_3^- exchange assay was performed as described above testing the compounds at varying concentrations. From these results the chloride efflux (%) at 270 s was plotted as a function of the carrier concentration (mol%, with respect to lipid). The data points were then fitted to the Hill equation using *OriginPro 9.1*:

$$y = V_{max}\frac{x^n}{k + x^n} = 100\%\frac{x^n}{(EC_{50})^n + x^n}$$

where y is the chloride efflux at 270 s (%) and x is the carrier concentration (mol %, with respect to lipid). V_{max}, k and n are the parameters to be fitted. V_{max} is the maximum efflux possible (usually fixed to 100 % as this is the maximum chloride efflux possible), n is the Hill coefficient and k is the carrier concentration needed to reach $V_{max}/2$ (when V_{max} is fixed to 100% k is the EC_{50}). EC_{50} values at 270 s can be obtained directly from the Hill plot.

Hill Analysis was performed for compounds **1d**, **2**, **4**, **5** and **6**, the chloride transport at pH 7.2 for compounds **3**, **7** and **8** was too weak to perform Hill analysis.

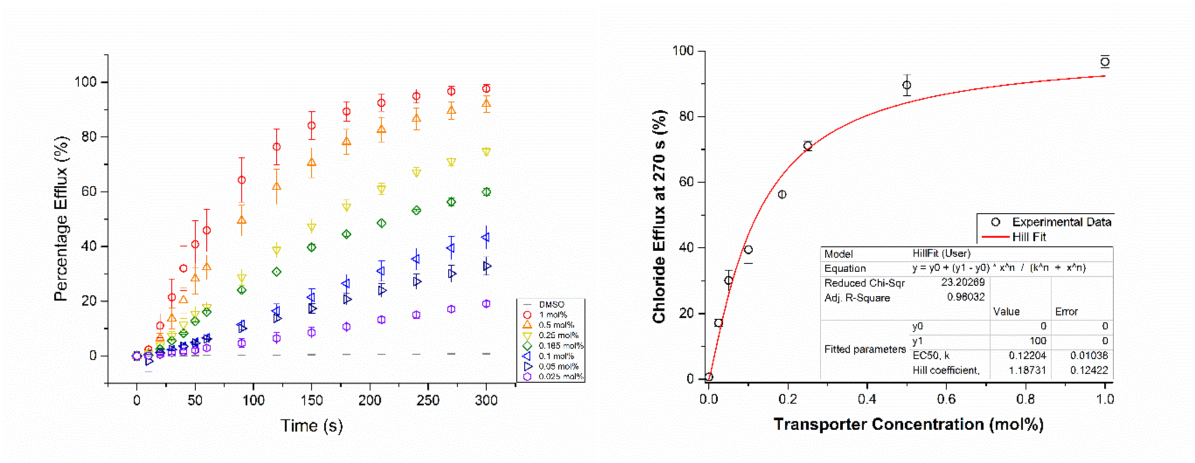

Figure S123. Plots for Hill analysis of chloride efflux facilitated by compound **1d**, from POPC vesicles loaded with NaCl (489 mM) and suspended in $NaNO_3$ (489 mM), buffered to pH 7.2 with sodium phosphate salts (5 mM). DMSO was used as a control. Each data point is the average of three repeated measurements with the error bars showing the standard deviation.

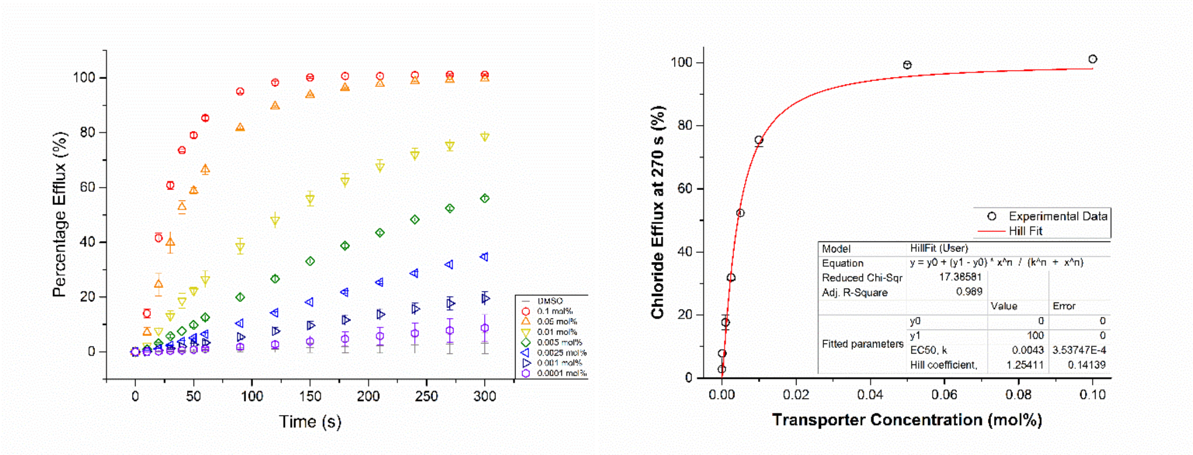

Figure S124. Plots for Hill analysis of chloride efflux facilitated by compound **2**, from POPC vesicles loaded with NaCl (489 mM) and suspended in $NaNO_3$ (489 mM), buffered to pH 7.2 with sodium phosphate salts (5 mM). DMSO was used as a control. Each data point is the average of three repeated measurements with the error bars showing the standard deviation.

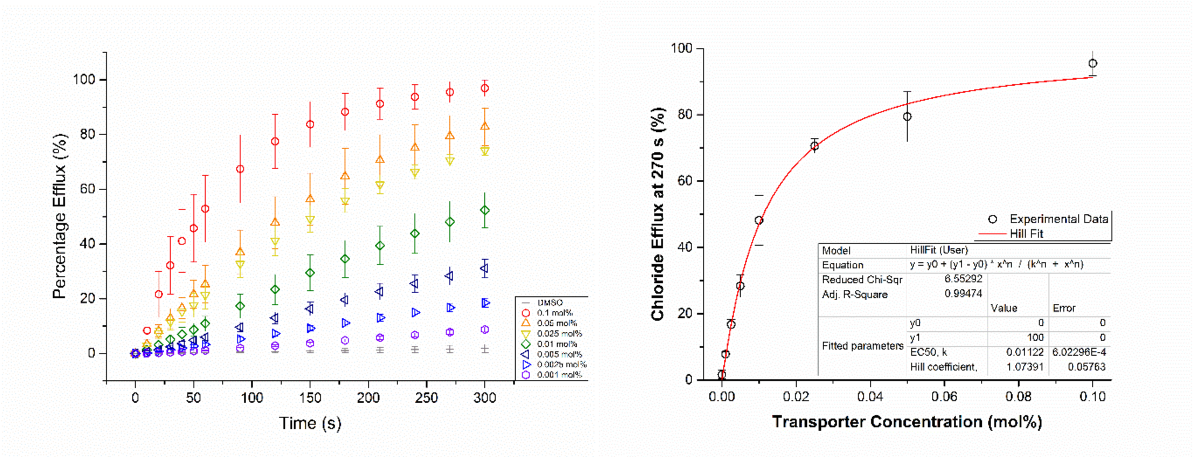

Figure S125. Plots for Hill analysis of chloride efflux facilitated by compound **4**, from POPC vesicles loaded with NaCl (489 mM) and suspended in $NaNO_3$ (489 mM), buffered to pH 7.2 with sodium phosphate salts (5 mM). DMSO was used as a control. Each data point is the average of three repeated measurements with the error bars showing the standard deviation.

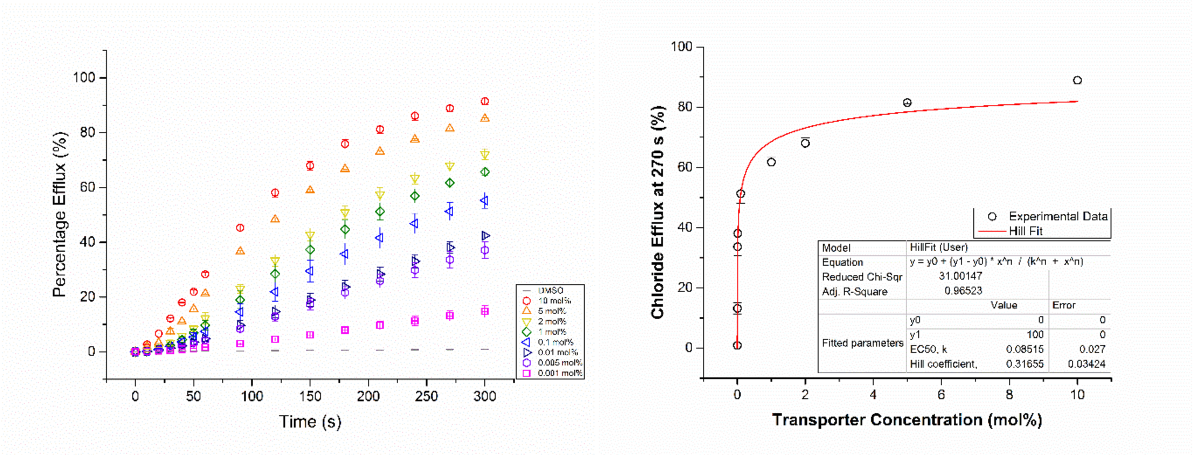

Figure S126. Plots for Hill analysis of chloride efflux facilitated by compound **5**, from POPC vesicles loaded with NaCl (489 mM) and suspended in $NaNO_3$ (489 mM), buffered to pH 7.2 with sodium phosphate salts (5 mM). DMSO was used as a control. Each data point is the average of three repeated measurements with the error bars showing the standard deviation.

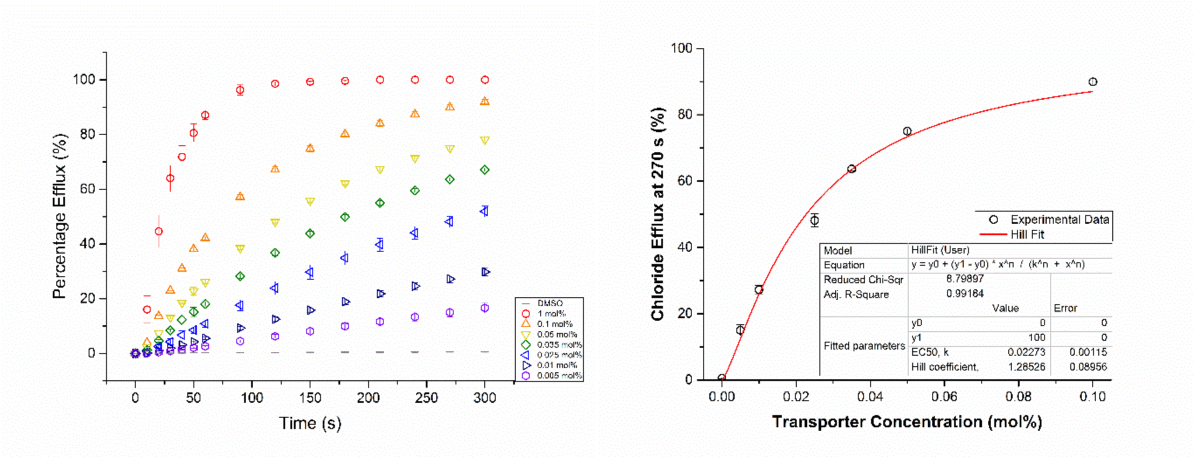

Figure S127. Plots for Hill analysis of chloride efflux facilitated by compound **6**, from POPC vesicles loaded with NaCl (489 mM) and suspended in $NaNO_3$ (489 mM), buffered to pH 7.2 with sodium phosphate salts (5 mM). DMSO was used as a control. Each data point is the average of three repeated measurements with the error bars showing the standard deviation.

S10.4. Cl^-/NO_3^- ISE Assay - POPC:Cholesterol (7:3) Vesicles

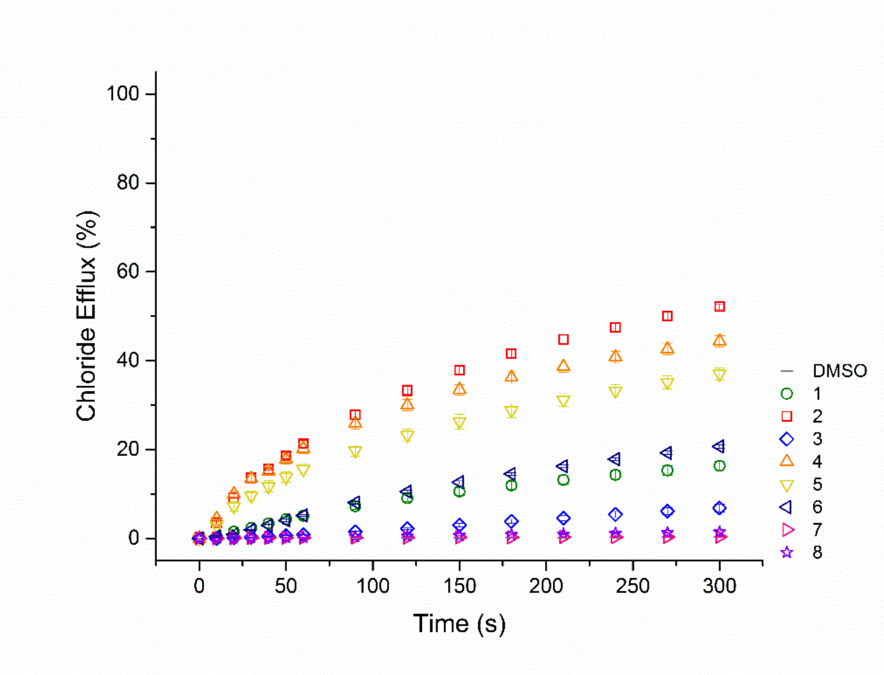

Figure S128. Chloride efflux facilitated by compounds **1d** and **2**-**8** at 0.1 mol% loading (with respect to lipid), from POPC:Cholesterol (7:3) vesicles loaded with NaCl (489 mM) and suspended in $NaNO_3$ (489 mM), buffered to pH 7.2 with sodium phosphate salts (5 mM). DMSO was used as a control. Each data point is the average of three repeated measurements with the error bars showing the standard deviation.

Discussion:

Cholesterol decreases the fluidity and therefore the permeability of vesicle membranes[12,13], so is used in transport studies to probe the effect on anion transport of changing the viscosity of the membrane. Comparing the efflux obtained at 0.1 mol% loading in POPC:Cholesterol (7:3) vesicles (**Figure S128**.) to that obtained at 0.1 mol% loading in POPC vesicles (**Figure S121**.) there appears to be a decrease in the percentage chloride efflux obtained in POPC:Cholesterol (7:3) vesicles. The trend of the receptors is the same, with **2** facilitating the highest chloride efflux, 100% in POPC and 70% in POPC:Cholesterol (7:3), and **7** and **8** showing no chloride efflux. The overall decrease in chloride efflux could be due to the cholesterol reducing the fluidity of the membrane so partitioning of the carrier is more difficult to achieve.

S10.5. Cl^-/SO_4^{2-} ISE Assay

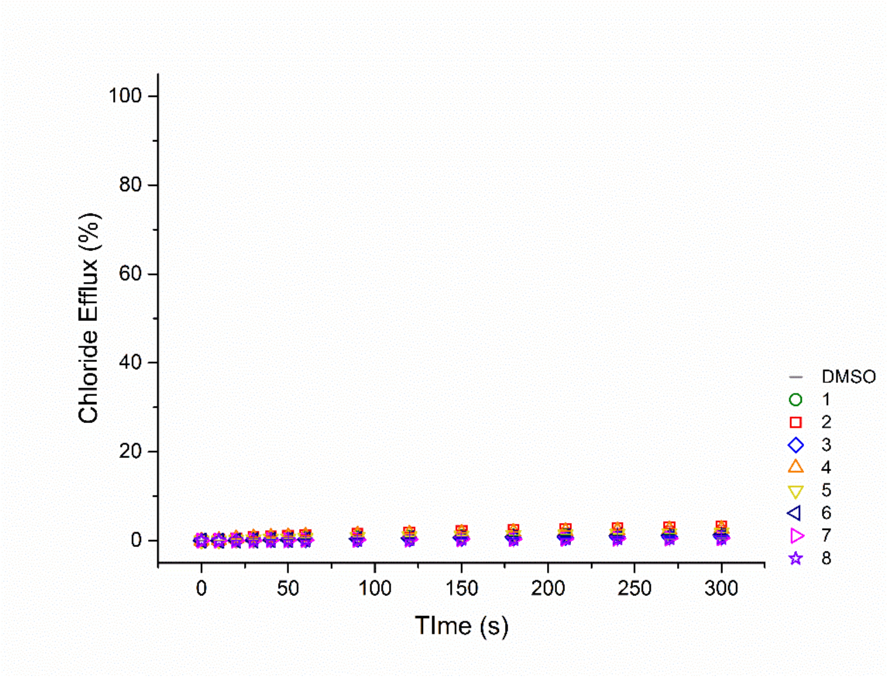

Figure S129. Chloride efflux facilitated by compounds **1d** and **2**-**8** at 0.1 mol% loading (with respect to lipid), from POPC vesicles loaded with NaCl (489 mM) and suspended in Na_2SO_4 (164 mM), buffered to pH 7.2 with sodium phosphate salts (5 mM). DMSO was used as a control. Each data point is the average of three repeated measurements with the error bars showing the standard deviation.

Discussion:

The external solution was changed from $NaNO_3$ to Na_2SO_4 as a control transport experiment. There was no chloride efflux observed for compounds **1d** and **2**-**8** showing that the perenosins cannot transport the hydrophilic sulfate anion.[14]

S10.6. Cl^-/HCO_3^- ISE Assay

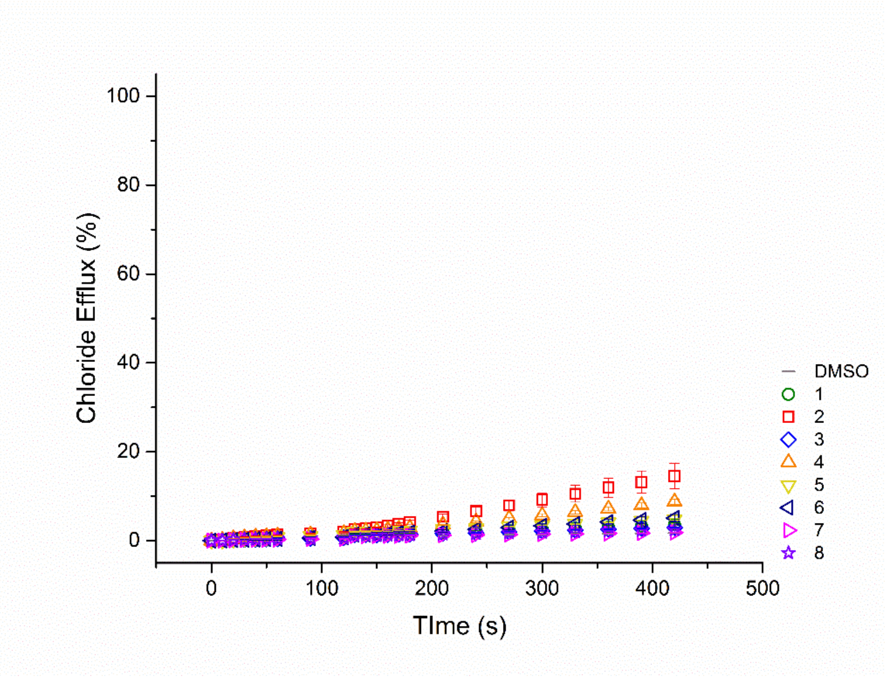

Figure S130. Chloride efflux facilitated by compounds **1d** and **2**-**8** added at 0 s, at 0.1 mol% loading (with respect to lipid), from POPC vesicles loaded with NaCl (489 mM) and suspended in Na_2SO_4 (164 mM), buffered to pH 7.2 with sodium phosphate salts (5 mM). At 120 s a $NaHCO_3$ solution was added to give a 40 mM external concentration, and after 7 mins the vesicles were lysed with detergent. The final chloride efflux reading was taken at 9 mins and was used as the 100% chloride efflux value. DMSO was used as a control. Each data point is the average of three repeated measurements with the error bars showing the standard deviation.

Discussion:

The bicarbonate anion is biologically important in some transporter proteins[9] so small molecules capable of facilitating bicarbonate transport are desirable. Compounds **1d** and **2**-**8** unfortunately do not show any significant bicarbonate transport.

S10.7 KCl/K_2SO_4 ISE Assay - Coupling to Monensin or Valinomycin

Vesicles were prepared according to the general procedure for the ISE assay. Internal and external solutions were the same for each experiment and are detailed in the caption.

The lipid stock solution was diluted with the external buffer solution to a standard volume (5 mL), to afford a solution with a lipid concentration of 1 mM. When using a cationophore (monensin or valinomycin), a DMSO solution (0.1 mol% (with respect to lipid), 10 µL) was added before the receptor to the solution. The compounds were added as a DMSO solution (0.1 mol% (with respect to lipid), 10 µL) to start the experiment and the resulting chloride efflux was monitored using a chloride selective electrode over 5 minutes. After this time, detergent (50 µL of Triron X-100 (11 w%) in H_2O:DMSO (7:1 v/v)) was added to lyse the vesicles and after 7 minutes a final chloride efflux reading was taken. This value represented 100 % chloride efflux and was used to calibrate the other readings.

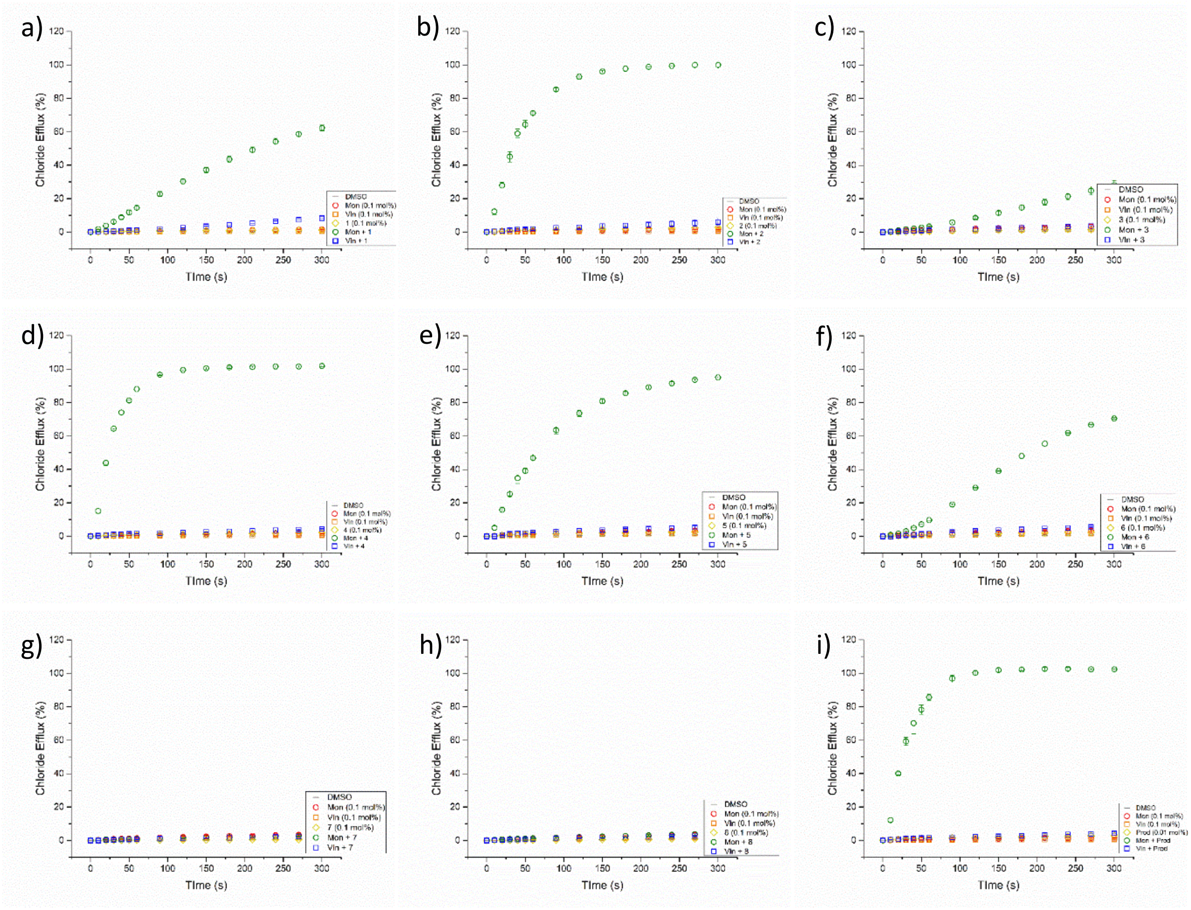

Figure S131. Chloride efflux facilitated by compounds **1d** (a), **2** (b), **3** (c), **4** (d), **5** (e), **6** (f), **7** (g) and **8** (h) at 0.1 mol% loading (with respect to lipid) and prodigiosin (i) at 0.01 mol% loading (with respect to lipid), from POPC vesicles loaded with KCl (300 mM) and suspended in K_2SO_4 (150 mM), buffered to pH 7.2 with potassium phosphate salts (5 mM) in the presence of valinomycin (0.1 mol% loading) or monensin (0.1 mol% loading). DMSO was used as a control. Each data point is the average of three repeated measurements with the error bars showing the standard deviation.

Discussion:

The complementary cationophore coupled assays[15] allow determination of whether an electrogenic or electroneutral transport mechanism is occurring. In electrogenic processes there is a net flow of charge across a membrane, and in an electroneutral process the flow of charge is balanced by antiport (eg. Cl^-/NO_3^- exchange) or symport (eg. Cl^-/H^+). Valinomycin facilitates electrogenic transport of K^+ so if the carrier shows transport in its presence it indicates an electrogenic transport process. Monensin functions as an M^+/H^+ antiporter which, when coupled to anion transport, is an electroneutral process. For prodigiosin and compounds **1d** and **2**-**6** the transport is coupled to monensin which indicates an electroneutral transport mechanism. The transporter facilitates H^+/Cl^- symport and monensin facilitates M^+/H^+ antiport which results in overall KCl efflux, corresponding to the green curve (**Figure S131**.). Compounds **7** and **8** do not facilitate chloride transport in this assay so no change from the DMSO control is seen.

S10.8. General Procedure for the HPTS Assay

Unilamellar vesicles were prepared using previously reported literature procedures[16]. A lipid film of POPC (1-palmitoyl-2-oleoyl-sn-glycero-3-phosphocholine) was prepared from a chloroform solution under reduced pressure and then dried under vacuum for 4 or more hours. The lipid film was rehydrated by vortexing with an internal solution containing HPTS (1 mM). The lipid suspension was then subjected to 9 freeze-thaw cycles and left to rest at room temperature for 30 minutes. After this, the suspension was extruded 25 times through a 200 nm polycarbonate membrane resulting in unilamellar vesicles of a mean diameter of 200 nm. The un-encapsulated HPTS was removed by size exclusion chromatography using a sephadex G-25 column and an external solution eluent that does not contain HPTS. The lipid solution obtained after dialysis was diluted to a standard volume (usually 10 mL) with the external buffered solution to obtain a lipid stock of known concentration.

In each experiment identical internal and external solutions were used with various salts (NMDG-Cl or sodium gluconate) buffered with HEPES (10 mM) to pH 7.0, and are detailed below.

The lipid stock was diluted with the external buffer solution to a standard volume (2.5 mL), to afford a solution with a lipid concentration of 0.1 mM. The compounds were added as a DMSO solution (10 µL) and when an assisting ionophore (gramicidin) or oleic acid (a naturally occurring monosaturated fatty acid) was used this was also added as a DMSO solution (5 µL, 0.1 mol% or 2 mol% respectively (with respect to lipid)). To start the experiment, after the addition of the compounds, a base pulse (25 µL, of NMDG or TBAOH (0.5 M)) was spiked in to generate a pH gradient across the membrane. After 200 s, detergent (50 µL of Triton X-100 (11 w%) in H_2O:DMSO (7:1 v/v)) was added to lyse the vesicles and after 5 mins a final reading was taken. This value represented 100 % and was used for calibration.

The fractional fluorescence intensity (I_f) was calculated using:

$$I_f = \frac{R_t - R_0}{R_d - R_0}$$

where R_t is the fluorescence ratio at time t, R_0 is the fluorescence ratio at time 0 and R_d is the fluorescence ratio at the end of the experiment, after the addition of detergent.

The HPTS exchange assay was performed as described above testing the compounds at varying concentrations. From these results the fluorescence ratio at 200 s was plotted as a function of the transporter concentration (mol%, with respect to lipid). The data points were then fitted to the Hill equation using *OriginPro 9.1*:

$$y = y_0 + (y_{max} - y_0)\frac{x^n}{k + x^n}$$

where *y* is the I_f at 200 s and *x* is the transporter concentration (mol %, with respect to lipid). y_0 is the I_f obtained for the blank DMSO run, y_{max} is the maximum I_f value and *k* and *n* are the parameters to be fitted. *n* is the Hill coefficient and *k* is the EC_{50}.

S10.9. HPTS Assay - NMDG-Cl

Compound **1d** without gramicidin

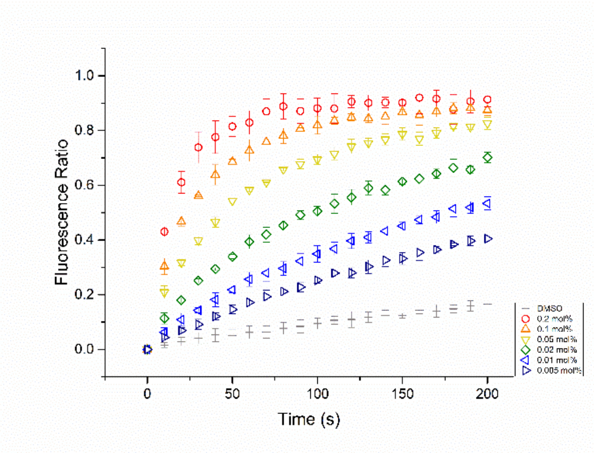

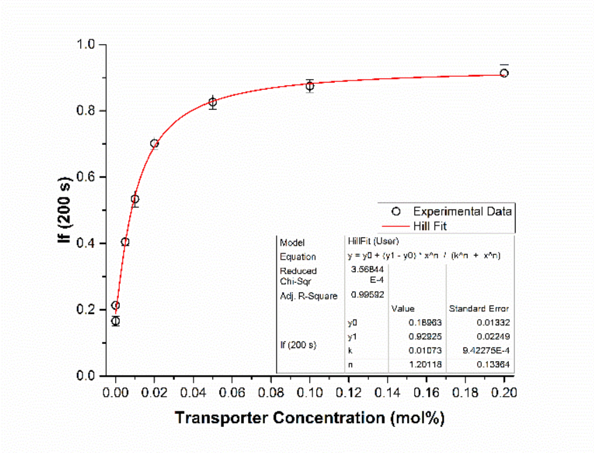

Compound **1d** with gramicidin

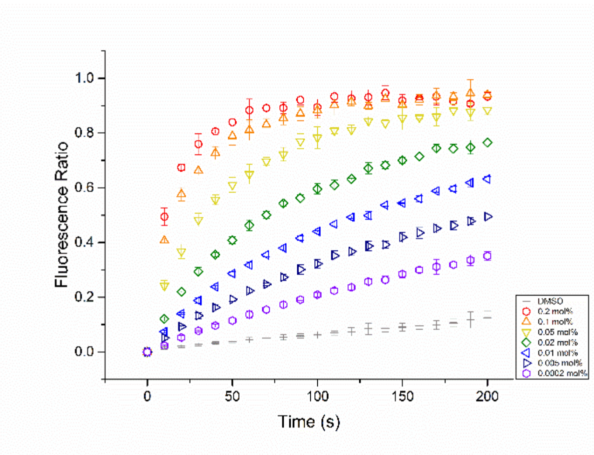

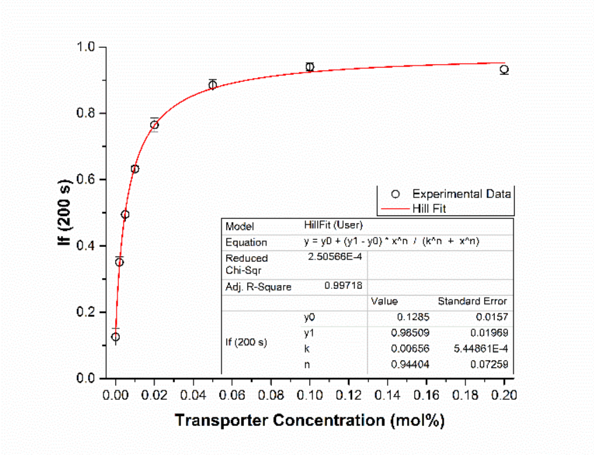

Figure S132. Hill plot analysis of H^+/Cl^- symport or Cl^-/OH^- antiport facilitated by compound **1d**, from POPC vesicles loaded with NMDG-Cl (100 mM), buffered to pH 7.0 with HEPES (10 mM), with and without gramicidin (0.1 mol%) present. DMSO was used as a control. Each data point is the average of three repeated measurements with the error bars showing the standard deviation.

Compound **2** without gramicidin

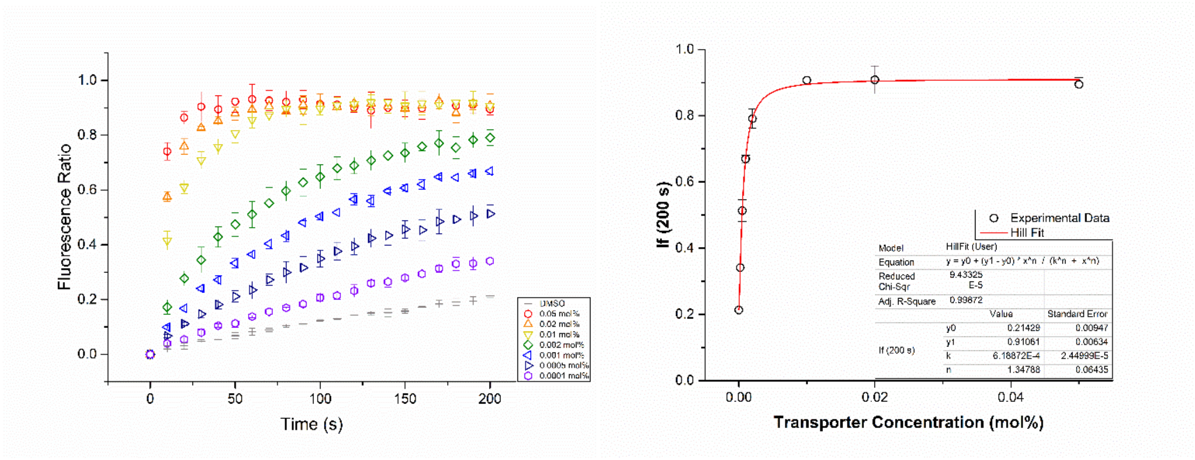

Compound **2** with gramicidin

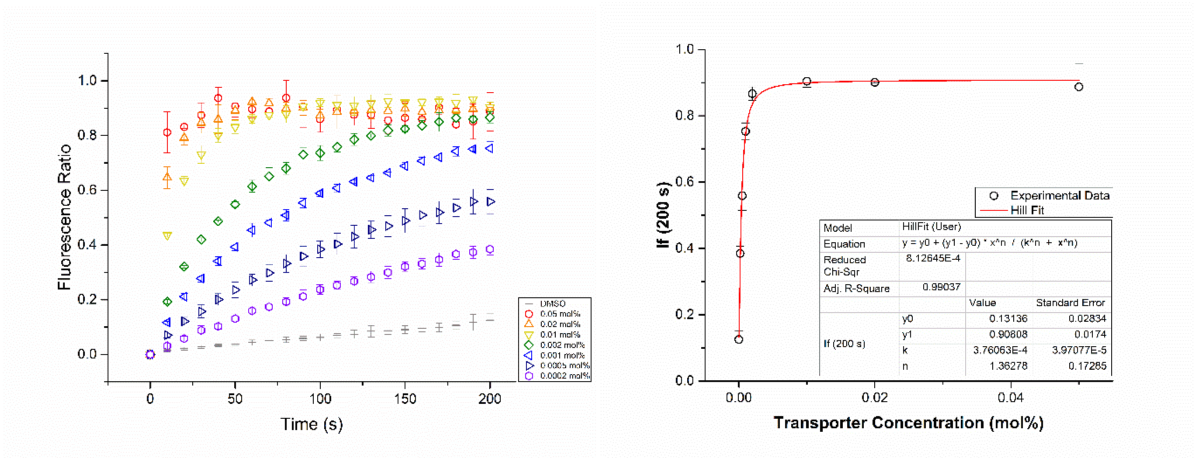

Figure S133. Hill plot analysis of H^+/Cl^- symport or Cl^-/OH^- antiport facilitated by compound **2**, from POPC vesicles loaded with NMDG-Cl (100 mM), buffered to pH 7.0 with HEPES (10 mM), with and without gramicidin (0.1 mol%) present. DMSO was used as a control. Each data point is the average of three repeated measurements with the error bars showing the standard deviation.

Compound **3** without gramicidin

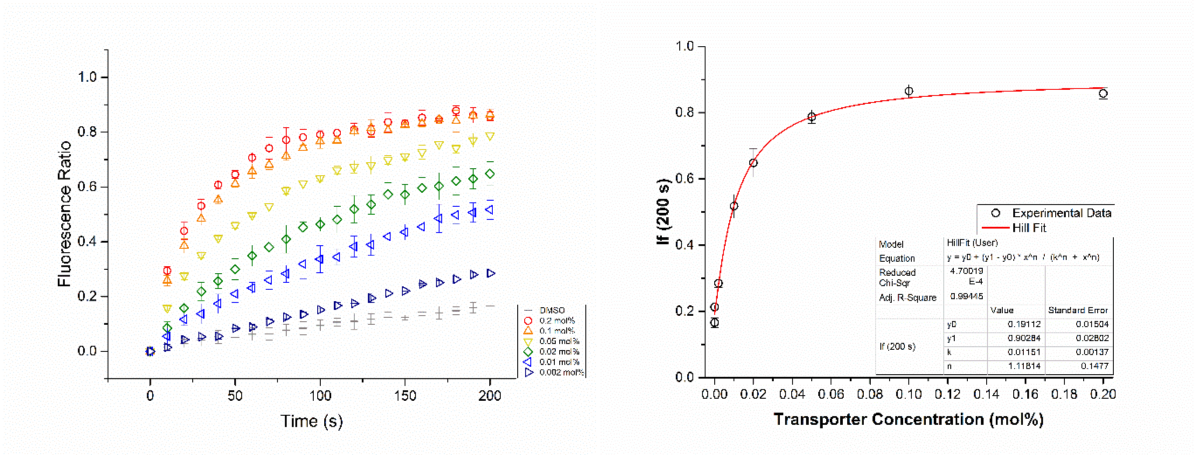

Compound **3** with gramicidin

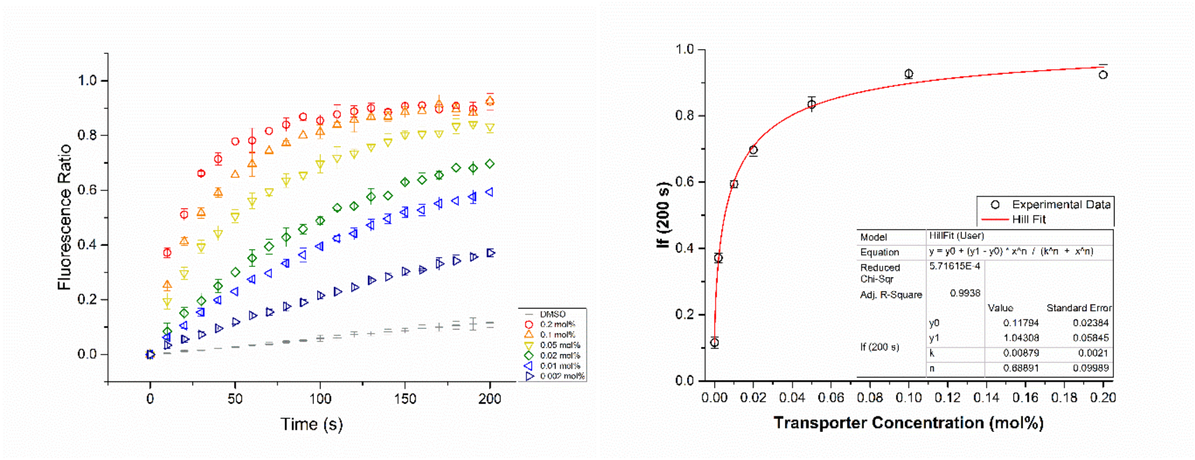

Figure S134. Hill plot analysis of H^+/Cl^- symport or Cl^-/OH^- antiport facilitated by compound **3**, from POPC vesicles loaded with NMDG-Cl (100 mM), buffered to pH 7.0 with HEPES (10 mM), with and without gramicidin (0.1 mol%) present. DMSO was used as a control. Each data point is the average of three repeated measurements with the error bars showing the standard deviation.

Compound **4** without gramicidin

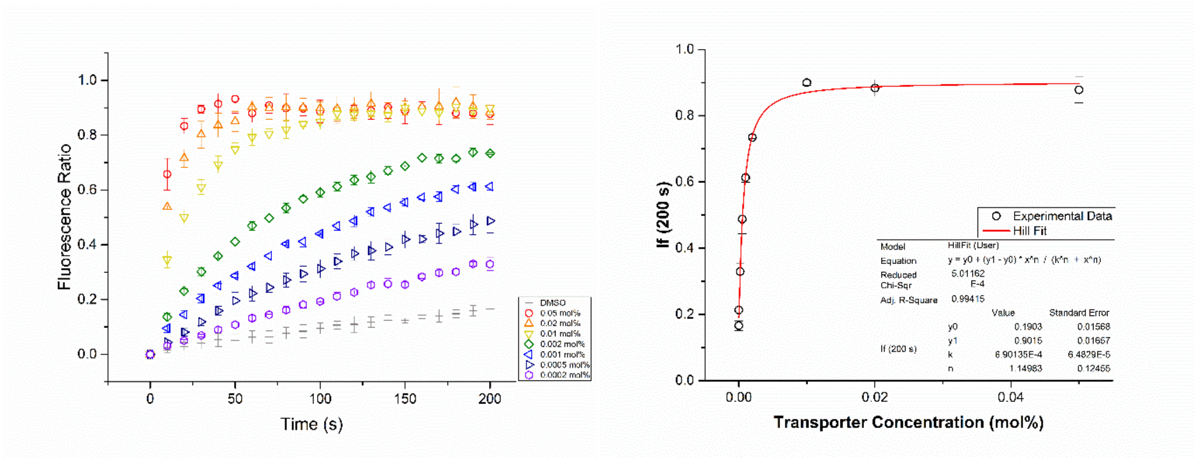

Compound **4** with gramicidin

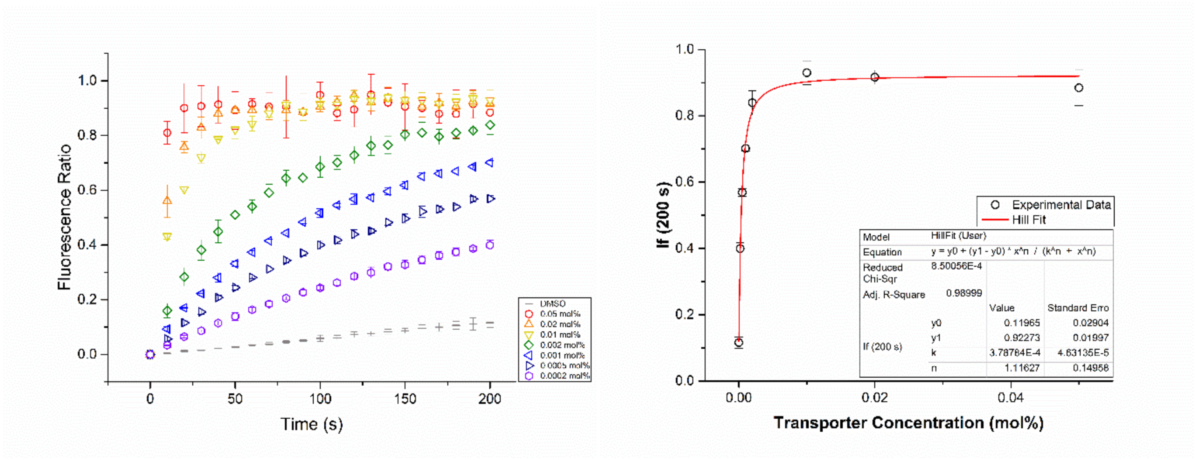

Figure S135. Hill plot analysis of H^+/Cl^- symport or Cl^-/OH^- antiport facilitated by compound **4**, from POPC vesicles loaded with NMDG-Cl (100 mM), buffered to pH 7.0 with HEPES (10 mM), with and without gramicidin (0.1 mol%) present. DMSO was used as a control. Each data point is the average of three repeated measurements with the error bars showing the standard deviation.

Compound **5** without gramicidin

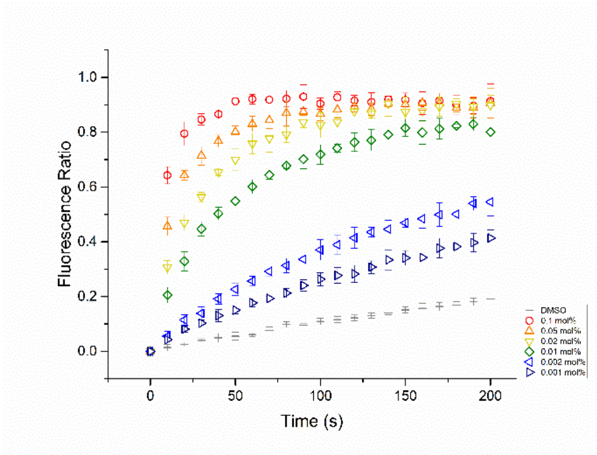

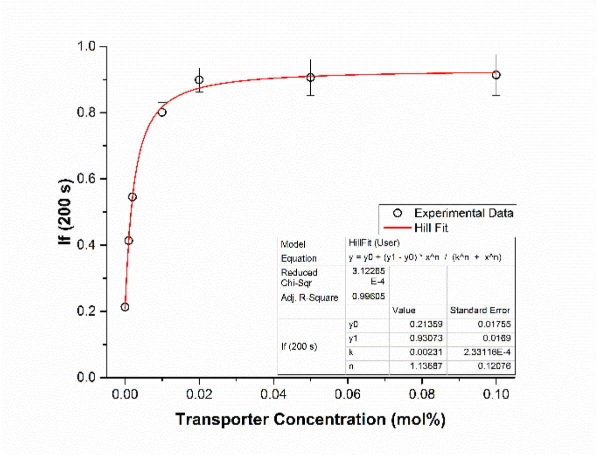

Compound **5** with gramicidin

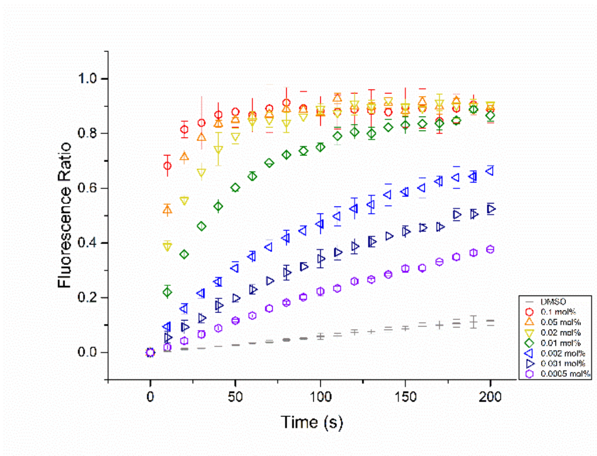

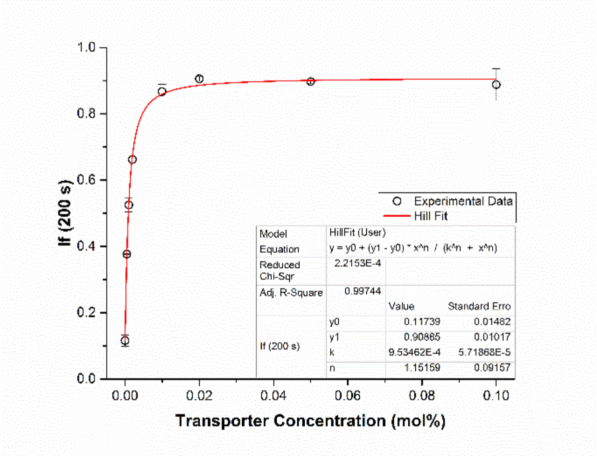

Figure S136. Hill plot analysis of H^+/Cl^- symport or Cl^-/OH^- antiport facilitated by compound **5**, from POPC vesicles loaded with NMDG-Cl (100 mM), buffered to pH 7.0 with HEPES (10 mM), with and without gramicidin (0.1 mol%) present. DMSO was used as a control. Each data point is the average of three repeated measurements with the error bars showing the standard deviation.

Compound **6** without gramicidin

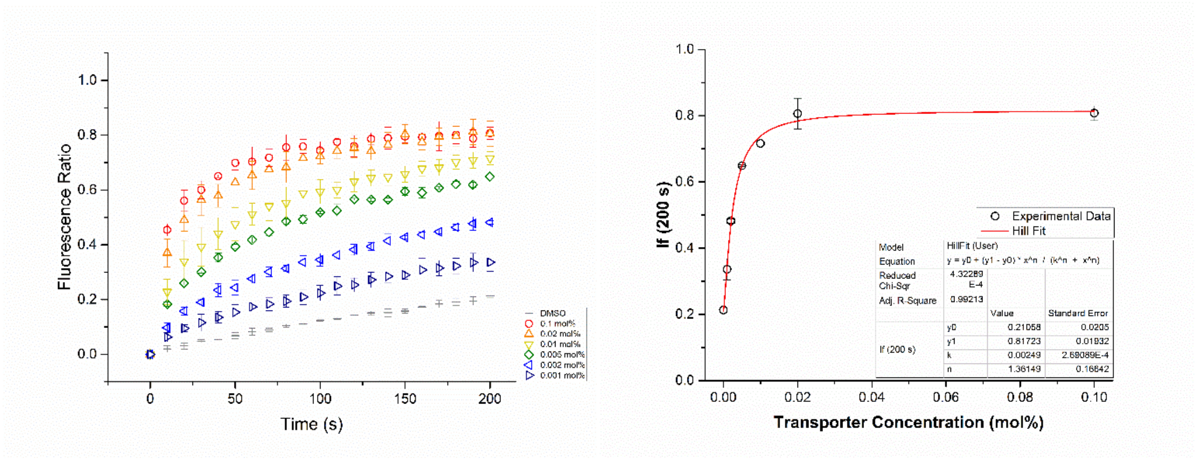

Compound **6** with gramicidin

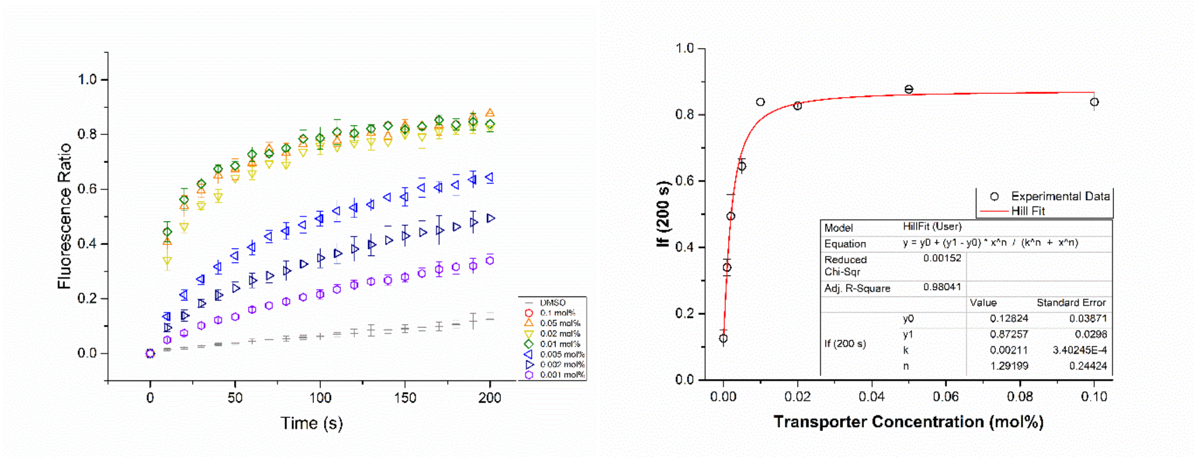

Figure S137. Hill plot analysis of H^+/Cl^- symport or Cl^-/OH^- antiport facilitated by compound **6**, from POPC vesicles loaded with NMDG-Cl (100 mM), buffered to pH 7.0 with HEPES (10 mM), with and without gramicidin (0.1 mol%) present. DMSO was used as a control. Each data point is the average of three repeated measurements with the error bars showing the standard deviation.

Discussion:

Compounds **1d** and **2**-**6** were tested in this assay[16] to gain insight into whether they show any selectivity for the transport of chloride over the transport of protons or hydroxide, as compounds **7** and **8** show minimal to no transport activity in other assays they were omitted. Addition of a base pulse created a pH gradient which can be equilibrated through H^+/Cl^- symport (or Cl^-/OH^- antiport) enabled by the transporter. Gramicidin facilitates electrogenic proton transport which removes the need for H^+/Cl^- symport (or Cl^-/OH^- antiport) by the transporter, therefore in the presence of gramicidin the ability of the compound to selectively transport chloride is tested. EC_{50} values were obtained in the absence and presence of gramicidin and the ratio between them was calculated to give a selectivity (S) value. The perenosins gave an S value of around 1 for all compounds which indicates no selectivity for chloride transport over H^+/OH^- transport.

S10.10. HPTS Assay - TBAOH

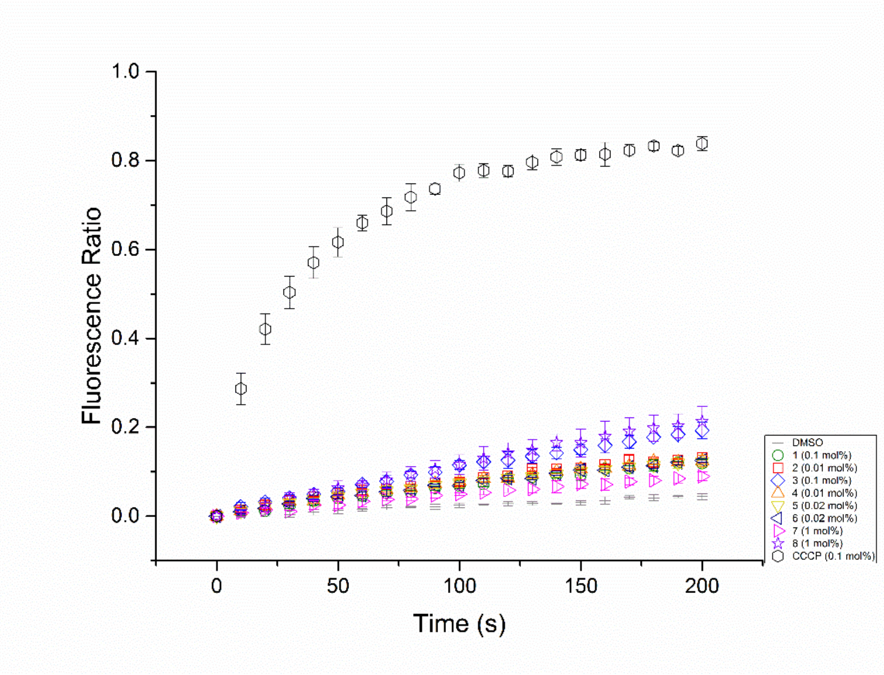

Figure S138. Single point screen of protonophore CCCP and compounds **1d** and **2**-**8** at varying concentrations, of H^+ or OH^- transport from POPC vesicles loaded with sodium gluconate (100 mM) and HPTS (1 mM), buffered to pH 7.0 with HEPES (10 mM). DMSO was used as a control. Each data point is the average of three repeated measurements with the error bars showing the standard deviation.

Discussion:

Electrogenic H^+ or OH^- transport is tested in this assay[16]. The TBAOH (0.5 M) base pulse creates a pH gradient across the membrane. The TBA^+ ion can diffuse across the membrane unaided leaving the pH gradient to be dissipated through the transport of H^+ or OH^-. CCCP is a protonophore and can facilitate the transport of H^+ across the membrane, achieving 100% at a loading of 0.1 mol% (**Figure S138**.). Compounds **1d** and **2**-**8** were tested at a loading that achieved 100% transport in the NMDG-Cl assay and no significant activity was observed for any compounds.

S10.11. HPTS Assay - Fatty Acids

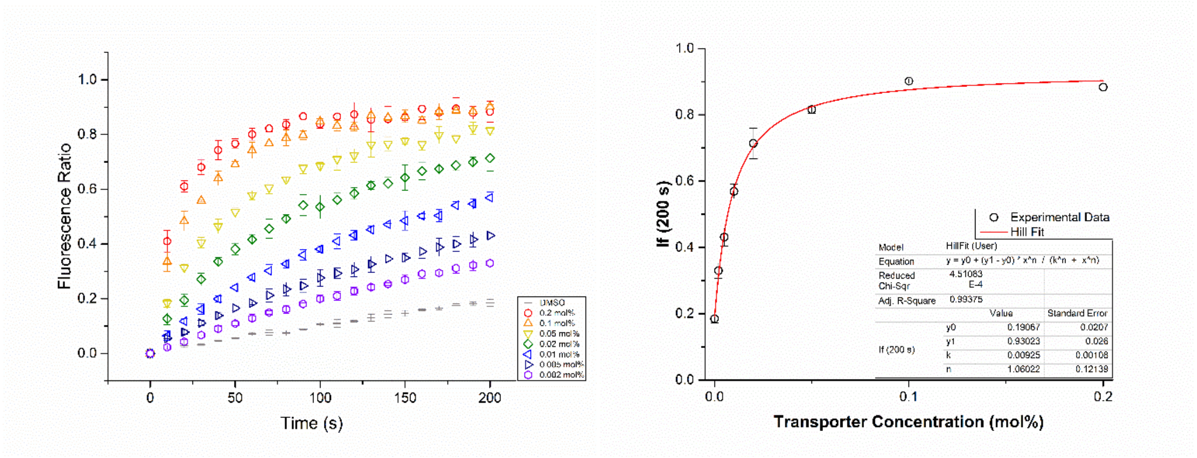

Figure S139. Hill plot analysis of H^+/Cl^- symport or Cl^-/OH^- antiport facilitated by compound **1d**, from POPC vesicles loaded with NMDG-Cl (100 mM), buffered to pH 7.0 with HEPES (10 mM), with oleic acid (2 mol%) present. DMSO was used as a control. Each data point is the average of three repeated measurements with the error bars showing the standard deviation.

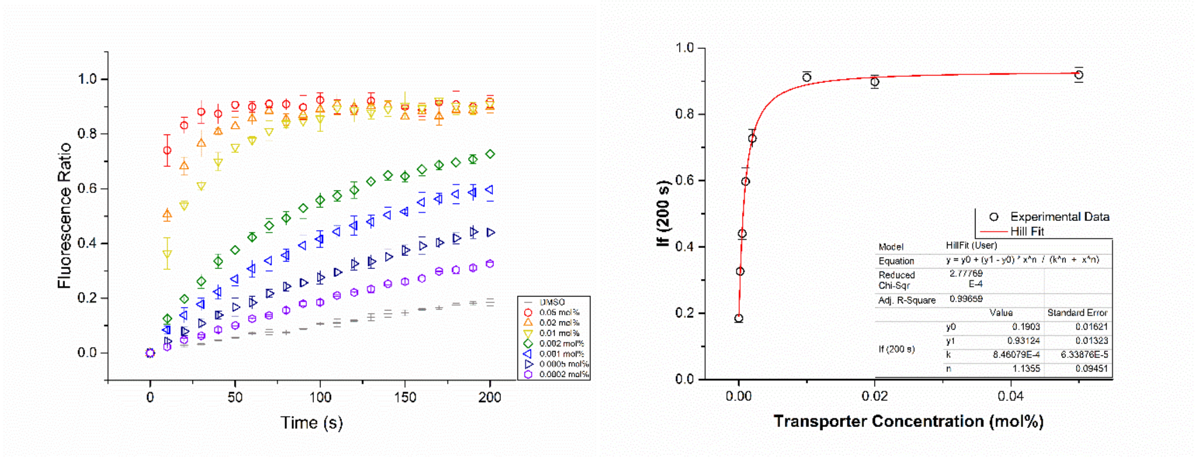

Figure S140. Hill plot analysis of H^+/Cl^- symport or Cl^-/OH^- antiport facilitated by compound **2**, from POPC vesicles loaded with NMDG-Cl (100 mM), buffered to pH 7.0 with HEPES (10 mM), with oleic acid (2 mol%) present. DMSO was used as a control. Each data point is the average of three repeated measurements with the error bars showing the standard deviation.

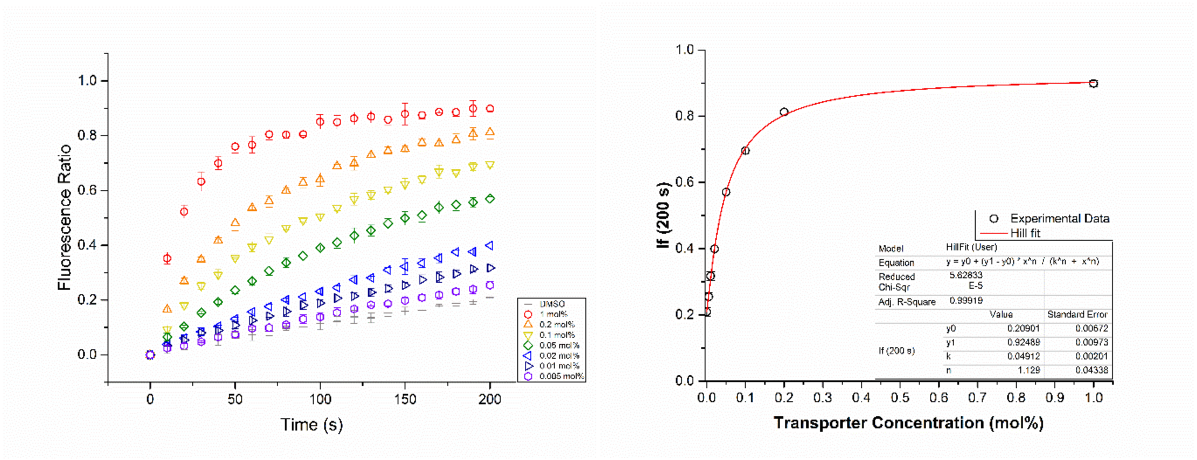

Figure S141. Hill plot analysis of H^+/Cl^- symport or Cl^-/OH^- antiport facilitated by compound **3**, from POPC vesicles loaded with NMDG-Cl (100 mM), buffered to pH 7.0 with HEPES (10 mM), with oleic acid (2 mol%) present. DMSO was used as a control. Each data point is the average of three repeated measurements with the error bars showing the standard deviation.

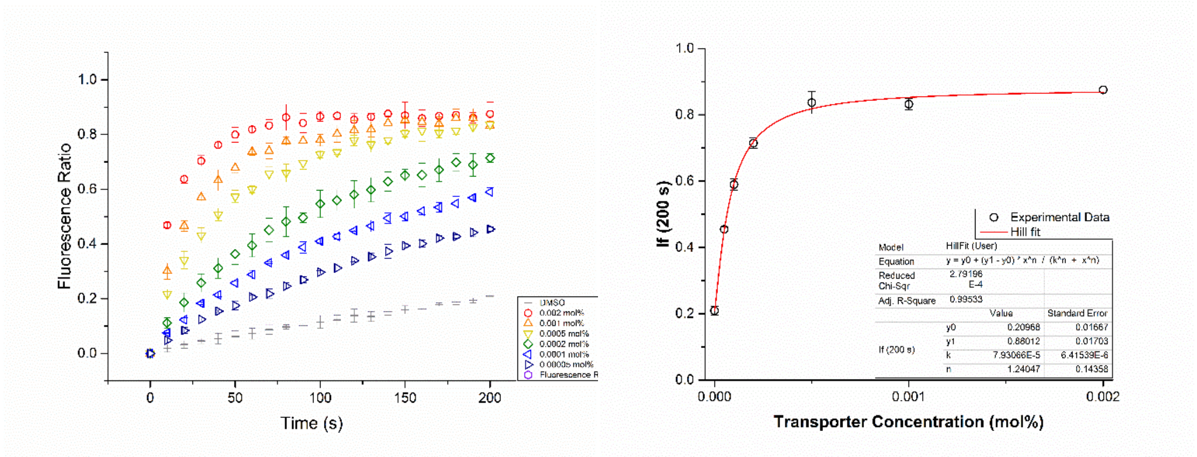

Figure S142. Hill plot analysis of H^+/Cl^- symport or Cl^-/OH^- antiport facilitated by compound **4**, from POPC vesicles loaded with NMDG-Cl (100 mM), buffered to pH 7.0 with HEPES (10 mM), with oleic acid (2 mol%) present. DMSO was used as a control. Each data point is the average of three repeated measurements with the error bars showing the standard deviation.

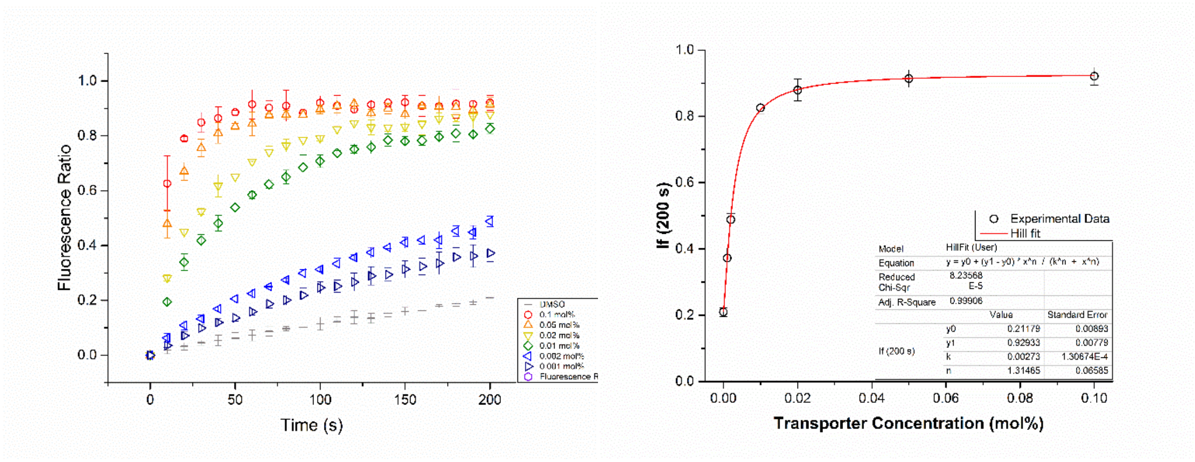

Figure S143. Hill plot analysis of H^+/Cl^- symport or Cl^-/OH^- antiport facilitated by compound **5**, from POPC vesicles loaded with NMDG-Cl (100 mM), buffered to pH 7.0 with HEPES (10 mM), with oleic acid (2 mol%) present. DMSO was used as a control. Each data point is the average of three repeated measurements with the error bars showing the standard deviation.

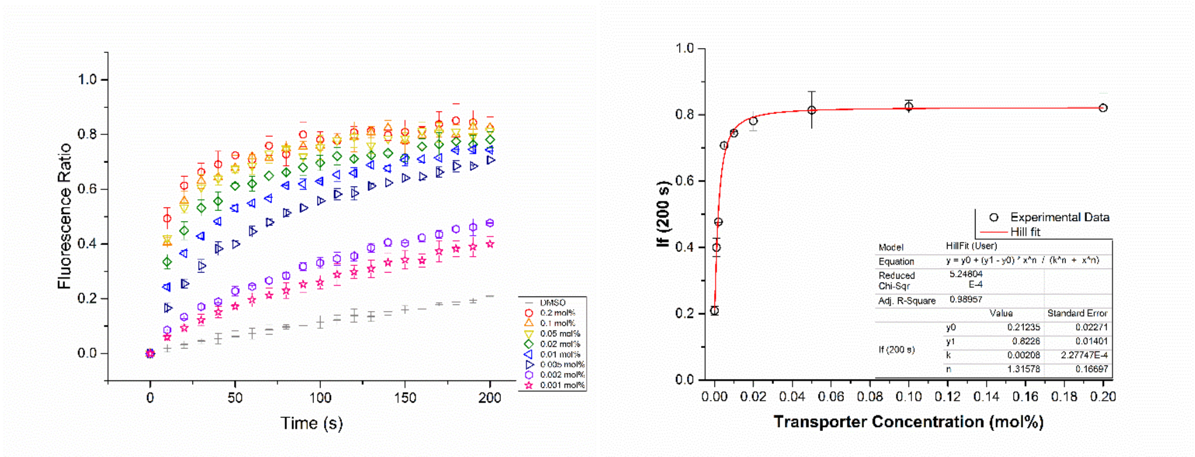

Figure S144. Hill plot analysis of H^+/Cl^- symport or Cl^-/OH^- antiport facilitated by compound **6**, from POPC vesicles loaded with NMDG-Cl (100 mM), buffered to pH 7.0 with HEPES (10 mM), with oleic acid (2 mol%) present. DMSO was used as a control. Each data point is the average of three repeated measurements with the error bars showing the standard deviation.

Discussion:

Compounds **1d** and **2**-**6** were tested in this assay to gain insight into whether they show any selectivity for the transport of chloride over the transport of protons or hydroxide assisted by the naturally occurring fatty acid flip-flop pathway. Compounds **7** and **8** show minimal to no transport activity in other assays therefore they were omitted. Addition of a base pulse created a pH gradient which can be equilibrated through H^+/Cl^- symport (or Cl^-/OH^- antiport) enabled by the transporter. Another pathway for pH dissipation is also available to the transporters. The receptor can bind to the deprotonated head group of a fatty acid and mask the charge, allowing the charged form of the fatty acid to pass across the membrane. This results in an enhancement of the proton shuttling pathway. EC_{50} values were obtained in the presence of oleic acid and the ratio between this and the free NMDG-Cl assay was calculated to give a selectivity (S) value. Perenosins **1d**, **2**, **3**, **5** and **6** gave an S value of around 1 which indicates there was no increase in transport rate in the presence of oleic acid and therefore no selectivity. Compound **4** gave an S value of 9, showing that in the presence of oleic acid the transport rate increased. This suggests that the receptor will bind the anionic oleic acid head group and facilitate the transport of the charged form across the lipid bilayer, therefore enhancing the pH dissipation and overall Cl^- transport.

S10.12. Preincorporated Leaching Assay

Some of the transporters were preincorporated into unilamellar vesicles[17,18]. A lipid film of POPC (1-palmitoyl-2-oleoyl-sn-glycero-3-phosphocholine) was prepared from a chloroform solution under reduced pressure and then dried under vacuum for 4 or more hours. The lipid film was rehydrated with a solution (1 mL) of the desired receptor in chloroform at a concentration that gave 100% chloride efflux in previous assays. This solution was mixed thoroughly and then the solvent was removed under reduced pressure to leave a film of lipid and receptor which was dried under vacuum overnight. The film was hydrated with an internal solution containing HPTS (1 mM), this was sonicated for 30 seconds and then stirred for 1 hour. The lipid suspension was then subjected to 9 freeze-thaw cycles and left to rest at room temperature for 30 minutes. After this, the suspension was extruded 25 times through a 200 nm polycarbonate membrane resulting in unilamellar vesicles of a mean diameter of 200 nm. The un-encapsulated HPTS was removed by size exclusion chromatography using a sephadex G-25 column and an external solution eluent that does not contain HPTS. The lipid solution obtained after dialysis was diluted to a standard volume (15 mL) with the external buffered solution to obtain a lipid stock of known concentration.

The lipid stock was diluted with the external buffer solution to a standard volume (2.5 mL), to afford a solution with a lipid concentration of 0.2 mM. To start the experiment a base pulse (25 µL, of NMDG, 0.5 M) was spiked in to generate a pH gradient across the membrane. After 400 s, detergent (50 µL of Triton X-100 (11 w%) in H_2O:DMSO (7:1 v/v)) was added to lyse the vesicles and after 8 mins a final reading was taken. This value represented 100 % and was used for calibration.

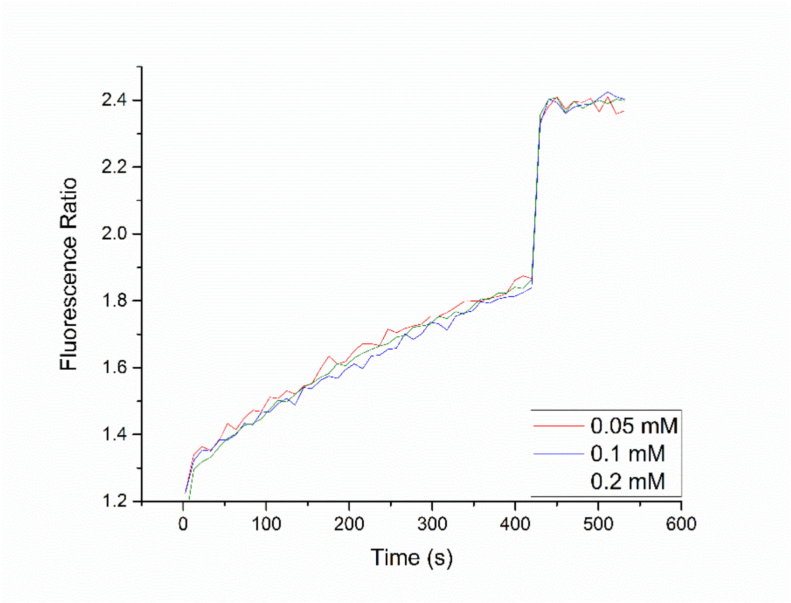

Figure S145. Chloride transport achieved by compound **1d** after preincorporation into POPC vesicles (0.05 mol% loading with respect to lipid) loaded with NMDG-Cl (100 mM) and HPTS (1 mM), buffered to pH 7.0 with HEPES (10 mM). A base pulse (NMDG, 0.5 M, 25 µL) was added at T=0 s to start the experiment. Three different lipid concentrations were tested and each fluorescence trace is the average of three repeated measurements.

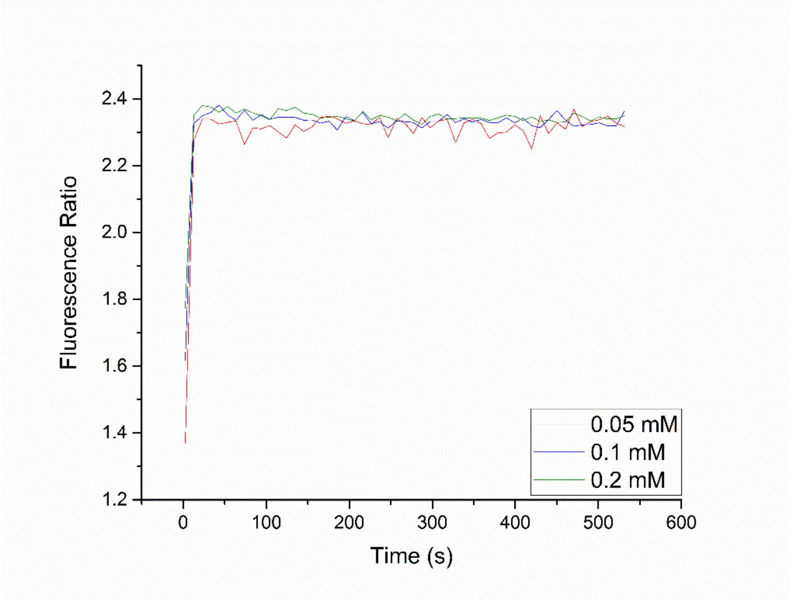

Figure S146. Chloride transport achieved by compound **2** after preincorporation into POPC vesicles (0.01 mol% loading with respect to lipid) loaded with NMDG-Cl (100 mM) and HPTS (1 mM), buffered to pH 7.0 with HEPES (10 mM). A base pulse (NMDG, 0.5 M, 25 µL) was added at T=0 s to start the experiment. Three different lipid concentrations were tested and each fluorescence trace is the average of three repeated measurements.

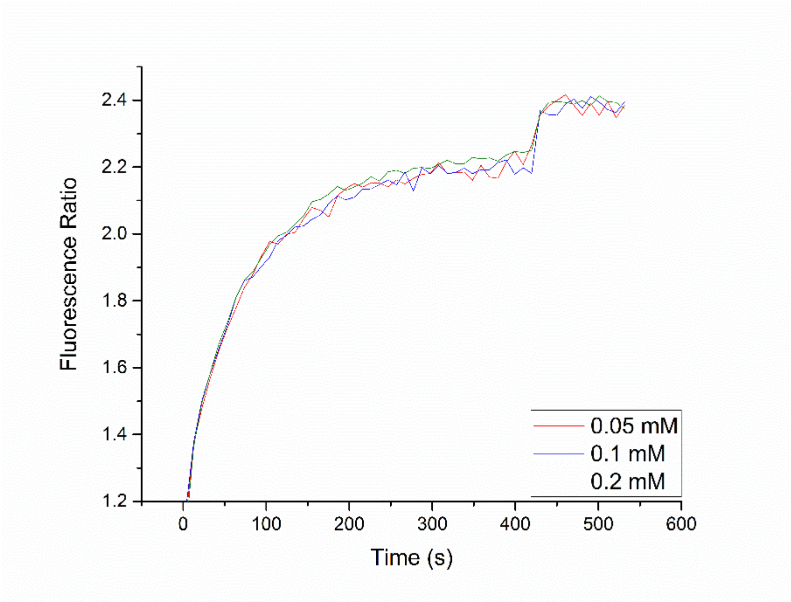

Figure S147. Chloride transport achieved by compound **3** after preincorporation into POPC vesicles (0.1 mol% loading with respect to lipid) loaded with NMDG-Cl (100 mM) and HPTS (1 mM), buffered to pH 7.0 with HEPES (10 mM). A base pulse (NMDG, 0.5 M, 25 µL) was added at T=0 s to start the experiment. Three different lipid concentrations were tested and each fluorescence trace is the average of three repeated measurements.

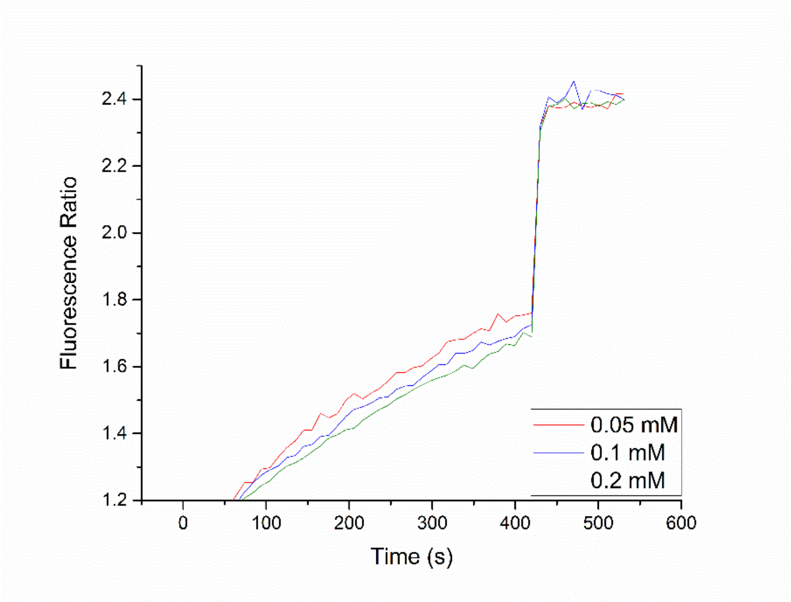

Figure S148. Chloride transport achieved by compound **4** after preincorporation into POPC vesicles (0.01 mol% loading with respect to lipid) loaded with NMDG-Cl (100 mM) and HPTS (1 mM), buffered to pH 7.0 with HEPES (10 mM). A base pulse (NMDG, 0.5 M, 25 µL) was added at T=0 s to start the experiment. Three different lipid concentrations were tested and each fluorescence trace is the average of three repeated measurements.

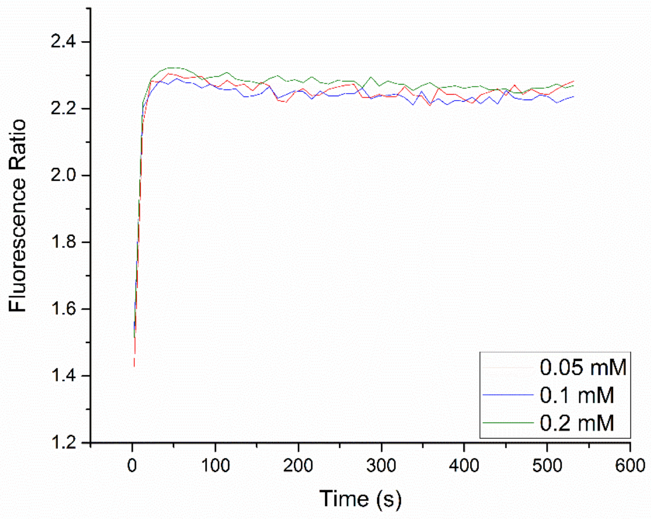

Figure S149. Chloride transport achieved by compound **5** after preincorporation into POPC vesicles (0.02 mol% loading with respect to lipid) loaded with NMDG-Cl (100 mM) and HPTS (1 mM), buffered to pH 7.0 with HEPES (10 mM). A base pulse (NMDG, 0.5 M, 25 µL) was added at T=0 s to start the experiment. Three different lipid concentrations were tested and each fluorescence trace is the average of three repeated measurements.

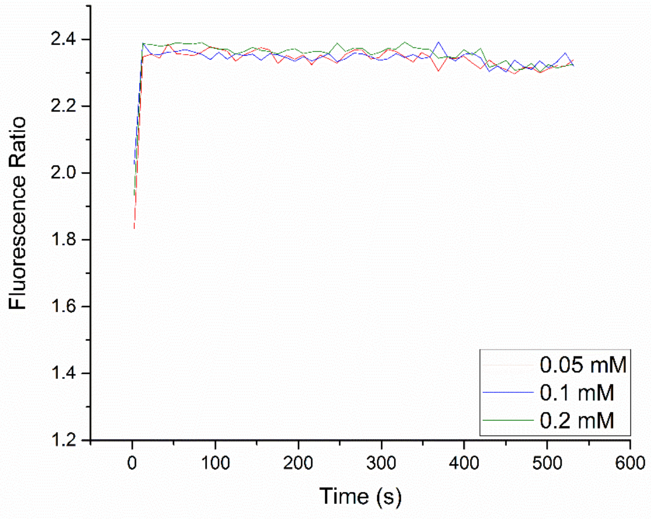

Figure S150. Chloride transport achieved by compound **6** after preincorporation into POPC vesicles (0.02 mol% loading with respect to lipid) loaded with NMDG-Cl (100 mM) and HPTS (1 mM), buffered to pH 7.0 with HEPES (10 mM). A base pulse (NMDG, 0.5 M, 25 µL) was added at T=0 s to start the experiment. Three different lipid concentrations were tested and each fluorescence trace is the average of three repeated measurements.

Discussion:

This assay was performed to monitor whether the compounds remain in the lipid bilayer or partition into the aqueous phase over the course of the experiments. Upon dilution of the lipid solution, if the transporter remains in the membrane, the traces at different concentrations should be superimposable. Whereas if the transporter partitions into the aqueous phase, there is a reduced concentration of it in the bilayer and so the transport rate decreases and the trace would be different. For the compounds tested, there is no notable change in the fluorescence trace (**Figure S145.** to **Figure S150.**) suggesting that the compounds remain in the bilayer over the course of the experiment. Another thing to note is that preincorporation enhances transport activity dramatically. This is because it removes the rate determining step of diffusion of the transporter into the lipid membrane.

S11. Cell Studies

S11.1 Cell Lines and Culture Conditions

The human cell lines were obtained from the American Type Culture Collection (ATCC, Manassas VA). Breast adenocarcinoma MDA-MB-231 and MCF-7 cells were maintained in DMEM:F12 media (1:1; Biological Industries, Beit Haemek, Israel), and 10 µg/ml insulin and 50 µM sodium pyruvate from Sigma-Aldrich Chemical Co. (St. Louis, MO) were added to MCF-7 cells. Lung adenocarcinoma A549 and colorectal adenocarcinoma SW620 cells were cultured in DMEM media. All were supplemented with 10% foetal bovine serum (FBS; Invitrogen-Life Technologies, Carlsbad, CA) and 100 U/mL penicillin, 100 µg/mL streptomycin and 2 mM L-glutamine, all from Biological Industries. Mammary epithelial MCF-10A cells were maintained in DMEM:F12 media supplemented with 5% horse serum (Life Technologies), 20 ng/ml EGF, 0.5 µg/ml Hydrocortisone, 100 ng/ml Cholera toxin, 10 µg/ml insulin all from Sigma-Aldrich and 100 U/ml penicillin, 100 µg/ml streptomycin, and 2 mM L- glutamine (Biological Industries). Cells were grown at 37ºC under a 5% CO_2 atmosphere.

S11.2 Cell Viability Assays

Cell viability was evaluated using the methylthiazoletetrazolium (MTT) colorimetric assay. Cancer cells (MCF-7, MDA-MB-231, A549 and SW620) or non-cancerous human cells MCF-10A cells were harvested (10^4 cells/well) in 96-well plates in a final volume of 100 µl and allowed to grow overnight. After 24 h, vehicle solution (DMSO) or experimental compounds were added at 10 µM (single point experiment) or at different ranging concentrations (0.8-100 µM) to the assay plate and were incubated for 24 or 72 h. Then, 10 µl of MTT (5 mg/ml; Sigma-Aldrich) were added and the plates were incubated for 4 h at 37°C. 100 µl of DMSO (Sigma-Aldrich) were added to dissolve crystals and the reading was taken spectrophotometrically at 570 nm using a multi-well plate reader (Multiskan FC, Thermo Scientific, Waltham, MA, USA). Cell viability and inhibitory concentration (IC) values were obtained using GraphPad Prism v5.0 (Graphpad Software, San Diego, CA, USA). All data are shown as the mean value ± standard deviation (S.D.) of three independent experiments.

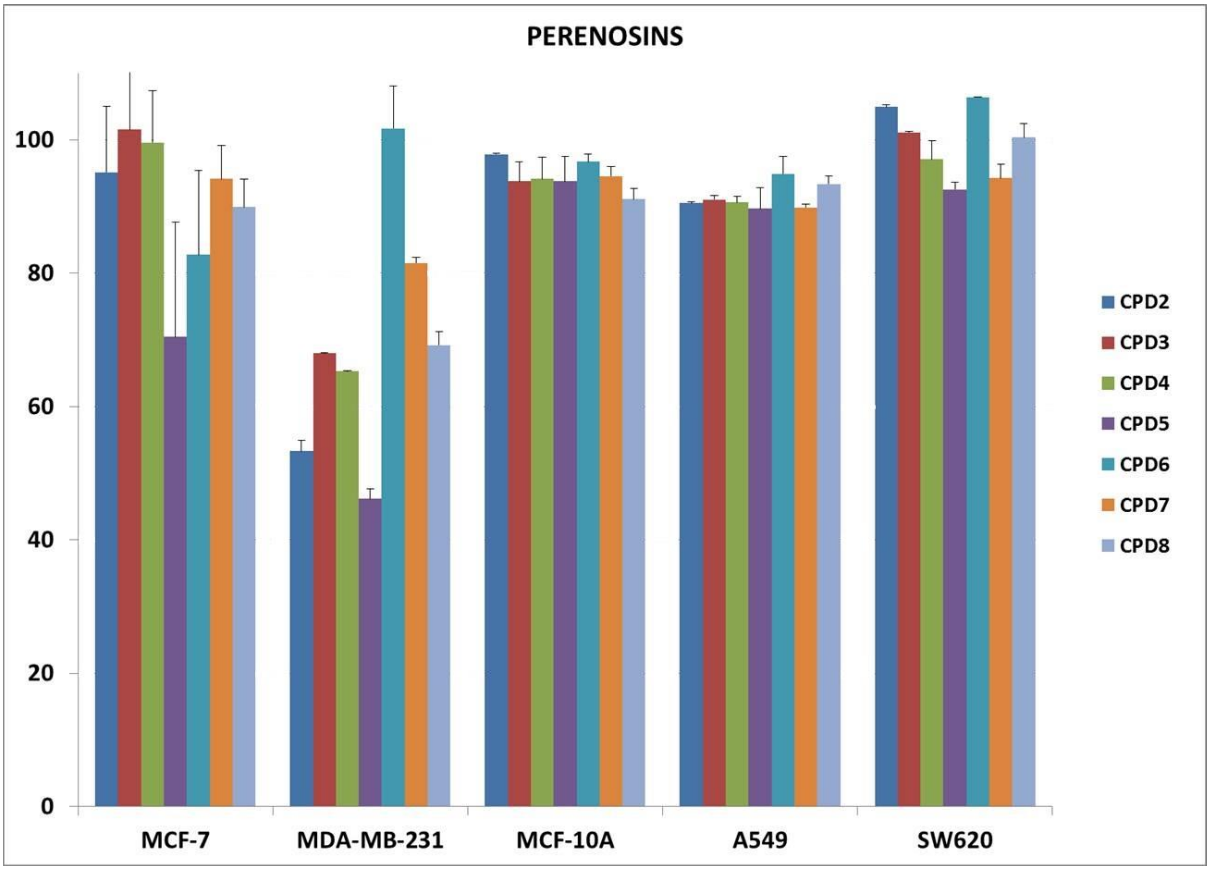

Figure S151. Single point MTT assay experiments for five cell lines performed in triplicate with error bars showing the standard deviation. A reduce in viability of MDA-MB-231 cells was observed for the perenosins after 24 hours.

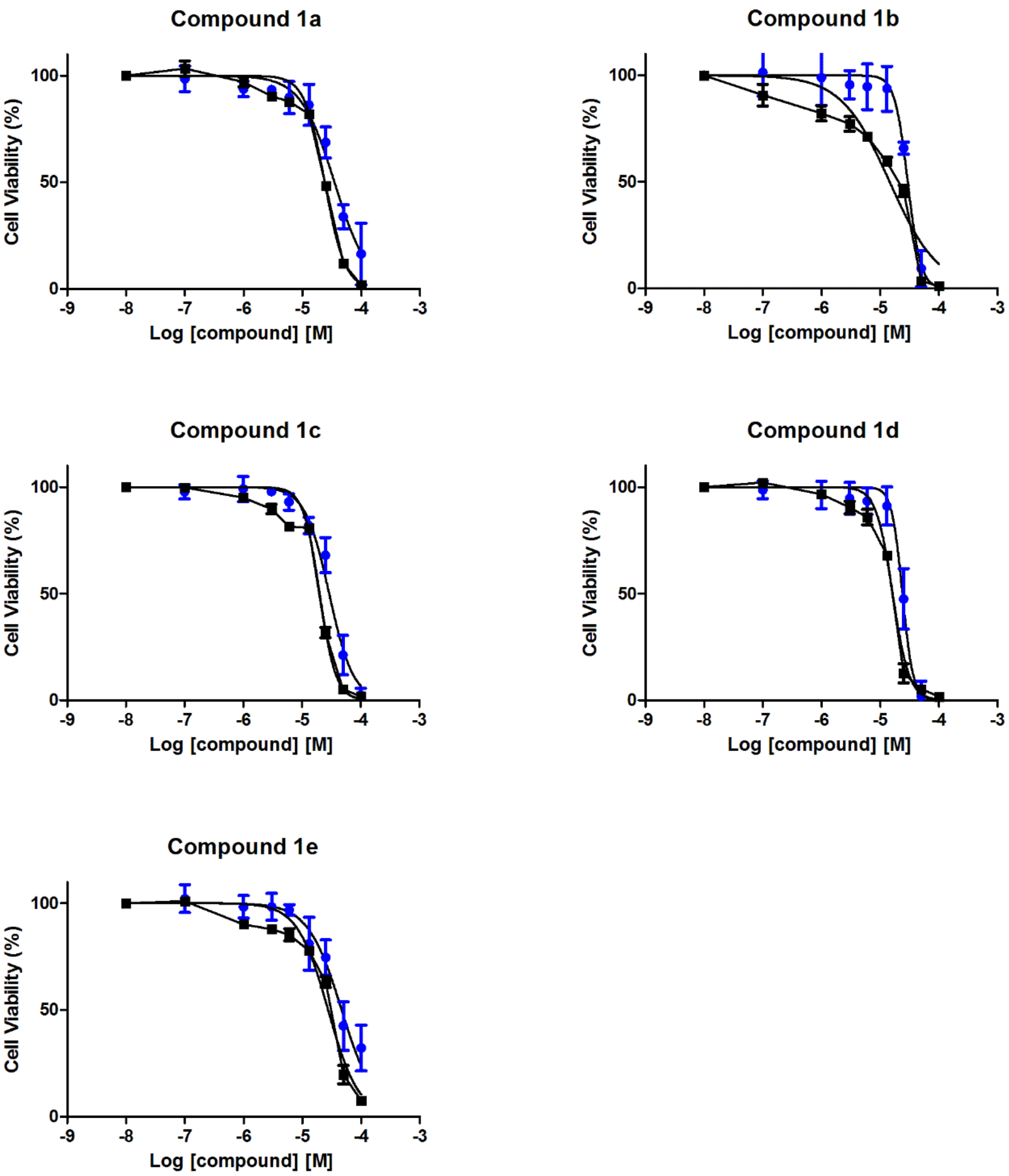

Figure S152. Dose-response cell viability curves from MDA-MB-231 cells treated with varying doses of compounds **1a**-**1e** for 24 h (blue) and 72 h (black), measured by the MTT assay.

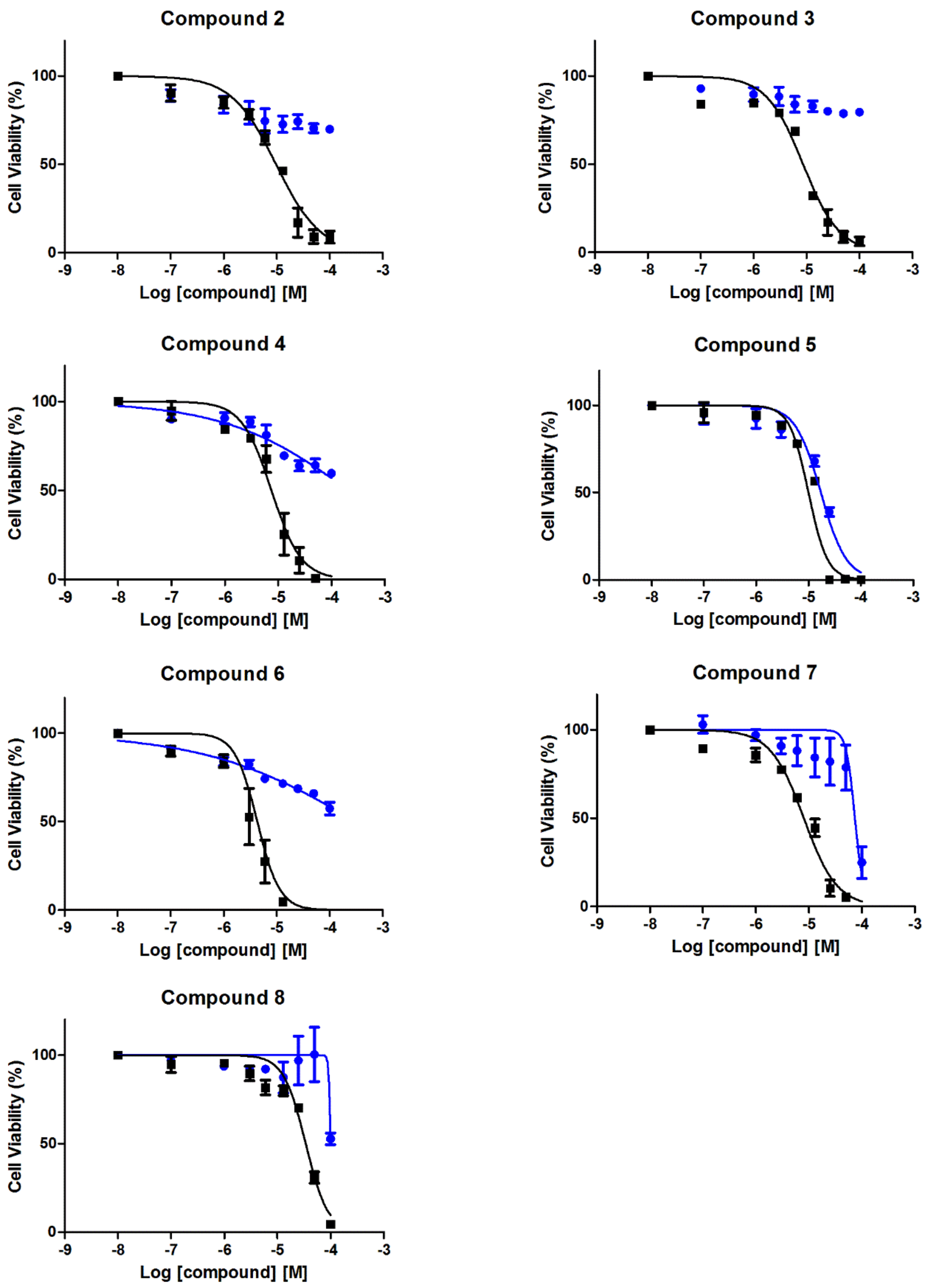

Figure S153. Dose-response cell viability curves from MDA-MB-231 cells treated with varying doses of compounds **2**-**8** for 24 h (blue) and 72 h (black), measured by the MTT assay.

S11.3 Apoptosis Evaluation through Annexin-V Assay

MDA-MB-231 cells ($1x10^5$ cells) were seeded in 6-well plates and, after 24 h, they were treated with IC_{50} values of compounds **A** and **B** for 48 and 72 h. Afterwards, cells were detached, centrifuged at 300 g for 5 min and resuspended in 1 ml of 1X PBS-1% FBS. Then, 100 µl of cell suspension were mixed with Annexin-V kit buffer (1:1; Muse Annexin V & Dead Cell Assay, Merck Millipore, Merck KGaA, Darmstadt, Germany). After 20 min incubation at room temperature, cells were examined on the Muse™ Cell Analyzer (Merck Millipore). All the conditions were assessed in three independent experiments. Results are shown as the mean value ± S.D. and representative results are shown in the selected histograms.

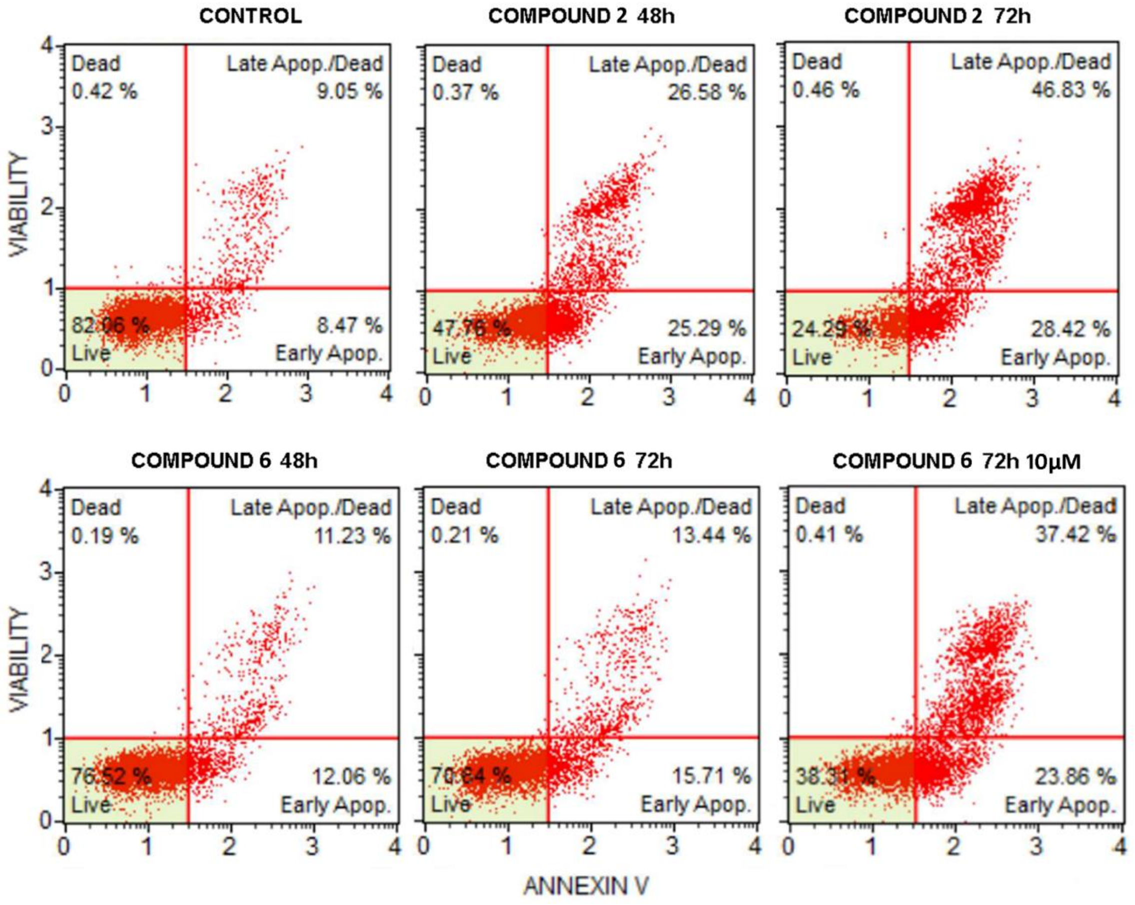

Figure S154. Flow cytometry of MDA-MB-231 cells treated with IC_{50} concentrations of compound **2** and **6** for 48 and 72 hours plus 10 µM of compound **6** for 72 h. Axes show Annexin-V binding versus cell viability.

References

1 Van Rossom, W., Asby, D. J., Tavassoli, A. & Gale, P. A. Perenosins: a new class of anion transporter with anti-cancer activity. *Org. Biomol. Chem.* **14**, 2645-2650, (2016).

2 Koradin, C., Dohle, W., Rodriguez, A. L., Schmid, B. & Knochel, P. Synthesis of polyfunctional indoles and related heterocycles mediated by cesium and potassium bases. *Tetrahedron* **59**, 1571-1587, (2003).

3 Sanz, R., Guilarte, V. & Perez, A. Straightforward selective preparation of nitro- or amino-indoles from 2-halonitroanilines and alkynes. First synthesis of 7-amino-5-nitroindoles. *Tetrahedron Lett.* **50**, 4423-4426, (2009).

4 Mikhalitsyna, E. A. *et al.* High-Yielding Synthesis of β-Octaalkyl-meso-(bromophenyl)-Substituted Porphyrins and X-ray Study of Axial Complexes of Their Zinc Complexes with THF and 1,4-Dioxane. *Eur. J. Inorg. Chem.* **2012**, 5979-5990, (2012).

5 Brown, D., Griffiths, D., Rider, M. E. & Smith, R. C. Synthesis of N-substituted prodigiosenes. *J. Chem. Soc., Perkin Trans. 1*, 455-463, (1986).

6 Rastogi, S. *et al.* Synthetic prodigiosenes and the influence of C-ring substitution on DNA cleavage, transmembrane chloride transport and basicity. *Org. Biomol. Chem.* **11**, 3834-3845, (2013).

7 MacDonald, R. C. *et al.* Small-volume extrusion apparatus for preparation of large, unilamellar vesicles. *Biochim. Biophys. Acta, Biomembr.* **1061**, 297-303, (1991).

8 Koulov, A. V. *et al.* Chloride Transport Across Vesicle and Cell Membranes by Steroid-Based Receptors. *Angew. Chem. Int. Ed.* **42**, 4931-4933, (2003).

9 Moore, S. J. *et al.* Towards "drug-like" indole-based transmembrane anion transporters. *Chem. Sci.* **3**, 2501-2509, (2012).

10 *Bindfit* http://supramolecular.org (accessed July 2017)

11 Thordarson, P. Determining association constants from titration experiments in supramolecular chemistry. *Chem. Soc. Rev.* **40**, 1305-1323, (2011).

12 Róg, T., Pasenkiewicz-Gierula, M., Vattulainen, I. & Karttunen, M. Ordering effects of cholesterol and its analogues. *Biochim. Biophys. Acta, Biomembr.* **1788**, 97-121, (2009).

13 Spooner, M. J. & Gale, P. A. Anion transport across varying lipid membranes - the effect of lipophilicity. *Chem. Commun.* **51**, 4883-4886, (2015).

14 Busschaert, N. *et al.* Synthetic transporters for sulfate: a new method for the direct detection of lipid bilayer sulfate transport. *Chem. Sci.* **5**, 1118-1127, (2014).

15 Howe, E. N. W. *et al.* pH-Regulated Nonelectrogenic Anion Transport by Phenylthiosemicarbazones. *J. Am. Chem. Soc.* **138**, 8301-8308, (2016).

16 Wu, X. *et al.* Nonprotonophoric Electrogenic Cl- Transport Mediated by Valinomycin-like Carriers. *Chem* **1**, 127-146, (2016).

17 Hussain, S., Brotherhood, P. R., Judd, L. W. & Davis, A. P. Diaxial Diureido Decalins as Compact, Efficient, and Tunable Anion Transporters. *J. Am. Chem. Soc.* **133**, 1614-1617, (2011).

18 Valkenier, H., Haynes, C. J. E., Herniman, J., Gale, P. A. & Davis, A. P. Lipophilic balance - a new design principle for transmembrane anion carriers. *Chem. Sci.* **5**, 1128-1134, (2014).
